# Global economic burden of unmet surgical need for appendicitis

**DOI:** 10.1093/bjs/znac195

**Published:** 2022-07-26

**Authors:** Anna Reuter, Lisa Rogge, Mark Monahan, Mwayi Kachapila, Dion G Morton, Justine Davies, Sebastian Vollmer, NIHR Global Surgery Collaboration, AA Essam, Abd Elkhalek Sallam, Abd Elrahman Elshafay, Abd El-Rahman Hegazy Khedr, Abdalla Gamal Saad, Abdalla Gharib, Abdalla Kenibar, Abdallah Salah Elsherbiny, Abdalrahman Adel, Abdelaziz Abdelaal, Abdelaziz Osman Abdelaziz Elhendawy, Abdelfatah Hussein, Abdelkader Belkouchi, Abdelmalek Hrora, Abdelrahman Adelshone, Abdelrahman Alkammash, Abdelrahman Assal, Abdelrahman Geuoshy, Abdelrahman Haroun, Abdelrahman Mohammed, Abdelrahman Sayed, Abdelrahman Soliman, Abdelrhman Essam Elnemr, Abdelrhman KZ Darwish, Abdelrhman Osama Elsebaaye, Abdul Khalique, Abdul Rehman Alvi, Abdul Wahid Anwar, Abdulaziz Altwijri, Abdullah Al-Mallah, Abdullah Almoflihi, Abdullah Altamimi, Abdullah Daqeeq, Abdullah Dwydar, Abdullah Gouda, Abdullah Hashim, Abdulmalik Altaf, Abdulmalik Huwait, Abdulrahman Abdel-Aty, Abdulrahman M. Altwigry, Abdulrahman Sheshe, Abdulrasheed A Nasir, AbdulRazzaq Oluwagbemiga Lawal, Abdulshafi Khaled Abdrabou, Abdurrahaman Sheshe, Abdussemiu Abdurrazzaaq, Abebe Bekele Zerihun, Abeer Al-shammari, Abeer El Gendy, Abeer Esam, Abeer Marey, Abhishek Mittal, Abiboye Yifieyeh, Abid Bin Mahamood, Abidemi Adesuyi, Abouelatta Khairy Aly, Abrar Nawawi, Adam Gyedu, Ade Waterman, Adedapo Osinowo, Adedeji Fatuga, Adel Albiety, Adel B Hassanein, Adel Denewar, Adeleke Adekoya, Ademola Adebanjo, Ademola Adeyeye, Ademola Popoola, Adesina Adedeji, Adesoji O Ademuyiwa, Adesoji Tade, Adewale Adeniyi, Adewale O Adisa, Adham Tarek, Adomas Ladukas, Adrian F. Palma, Afifatun Hasanah, Afizah Salleh, Afnan Abdelfatah, Afnan Altamimi, Afnan Altamini, Agazi Fitsum, Agboola Taiwo, Ahamed Hassan, Ahed Ghaben, Ahmad Abdel Fattah, Ahmad Abdel Razaq Al Rafati, Ahmad Aboelkassem Ibrahem, Ahmad Aldalaq, Ahmad Ali, Ahmad Almallah, Ahmad Alrifaie, Ahmad Ashour, Ahmad Bakr, Ahmad Bani-Sadar, Ahmad Bin Adnan, Ahmad Elbatahgy, Ahmad Faraz, Ahmad Gudal, Ahmad Hasan, Ahmad Khaled Sabe, Ahmad Khoja, Ahmad Nashaat, Ahmad Qaissieh, Ahmad Sabe, Ahmad Saber Sleem, Ahmad Sakr, Ahmad Shalabi, Ahmad Uzair Qureshi, Ahmed Aamer, Ahmed Abd El Galeel, Ahmed Abd Elmoen Elhusseiny, Ahmed Abd Elsameea, Ahmed Abdelkareem, Ahmed Abdelmotaleb Ghazy, Ahmed Abo El Magd, Ahmed Abo Elazayem, Ahmed Adamu, Ahmed Adel, Ahmed Afandy, Ahmed Ahmed, Ahmed Alghamdi, Ahmed Ali, Ahmed Al-khatib, Ahmed Altibi, Ahmed Alzahrani, Ahmed Ata, Ahmed Badr, Ahmed Dahy, Ahmed Diab, Ahmed El Kashash, Ahmed El Kholy, Ahmed Elgaili Khalid Musa, Ahmed Elgebaly, Ahmed Elkelany, Ahmed Elkholy, Ahmed El-Sehily, Ahmed Essam, Ahmed Fahiem, Ahmed Farag, Ahmed Fawzy, Ahmed Fouad, Ahmed Gad, Ahmed Ghanem, Ahmed Gheith, Ahmed Gomaa, Ahmed Hafez El-Badri Kotb, Ahmed Hammad, Ahmed Hassan, Ahmed Hossam Eldin Fouad Rida, Ahmed Ismail, Ahmed Karim, Ahmed Khyrallh, Ahmed Lasheen, Ahmed M. Rashed, Ahmed Magdy, Ahmed Mahmoud Abdelraouf, Ahmed Menshawy, Ahmed Meshref, Ahmed Mohamed Afifi, Ahmed mohamed Ibrahim, Ahmed Mohameden, Ahmed Mohammed, Ahmed Mokhtar, Ahmed Mosad, Ahmed Moustafa, Ahmed Moustafa Saeed, Ahmed Negida, Ahmed Rabeih Mohammed, Ahmed Rabie Mohamed, Ahmed Ragab Nayel, Ahmed Ragab Soliman, Ahmed Raslan, Ahmed Raza, Ahmed Refaat, Ahmed Rslan, Ahmed Sabry, Ahmed Sabry El-Hamouly, Ahmed Safwan Marey, Ahmed Saidbadr, Ahmed Sakr, Ahmed Samir, Ahmed Shahine, Ahmed Sheta, Ahmed Soliman, Ahmed Tammam, Ahmed Tarek Abdelbaset Hassan, Ahmed W. Shamsedine, Ahmed Zaki, Ahmed Zaki Eldeeb, Ahmed Zohair, Ahmedali M Kandil, Ahmedglal Elnagar, Ahsan Zil-E-Ali, Aijaz Jabbar, Ailsa Claire Snaith, Ainhoa Costas-Chavarri, Aiste Austraite, Ajayesh Mistry, Akin Olaolorun, Akinlabi E Ajao, Al Faifi Jubran, Ala Shamasneh, Alaa Abouelnasr, Alaa Al-Buhaisi, Alaa Bowabsak, Alaa El Jamassi, Alaa Elazab, Alaa Elhadad, Alaa Fergany, Alaa Habeebullah, Alaa Hassan, Alaa Shabkah, Alaa Shaheen, Alaba Adesina, Alan Baird, Alan Grant, Alasdair Ball, Alban Cacurri, Albert Mohale Mphatsoe, Alberto Realis Luc, Alejandro Matheu, Alejandro Munera, Alemayehu Ginbo Bedada, Alessandro Favero, Alessio Maniscalco, Alexander Canta Calua, Alexander J Fowler, Alexandra Gerosa, Alexandre Horobjowsky, Alexandre Venancio De Sousa, Alexia Farrugia, Alexis Pierre Arnaud, Alfio Alessandro Russo, Alfredo Gulielmi, Ali Ababneh, Ali Abo El Dahab, Ali Amin Ahmed Ata, Ali Khan Niazi, Ali Kiasat, Ali Mohamed Hammad, Ali Zardab, Ali Zeynel Abidin Balkan, Aliaa Gamal Toeema, Aliaa Sadek, Aliaksandr Filatau, Aliang Latif, Alibeth Andres Baquero Suarez, Alice Faure, Alice Niragire, Alina Robledo-Rabanal, Aline Broch, Alireza Hasheminia, Alisdair Macdonald, Aliyu Ndajiwo, Allan Novak, Alphonse Zeta Mutabazi, Alvaro Enrique Mendoza Beleño, Alvin Ee Zhiun Cheah, Aly Abd Elrazek, Aly Nasr, Aly Sanad, Alyaa Halim Elgendy, Alyne Daltri Lazzarini Cury, Amal Ibrahim, Amandine Martin, Amani Althwainy, Amany Abouzahra, Amany Eldosouky Mohammed, Amar Kourdouli, Amel Hashish, Amerdip Birring, Amgad Al Meligy, Amina Abdelhamid, Aminah Hanum Haji Abdul Majid, Aminu Mohammad, Amir Ait Kaci, Amira Atef Omar, Amira Elsawy, Amira Hassan Bekhet, Amira Reda, Amjad Abu Qumbos, Amjad Elmashala, Ammar Gado, Amna Mamdouh Mohamed, Amna Mohamed, Amoudtha Rasendran, Amr Ahmed Saleh, Amr Fadel, Amr Hasan, Amr Hassaan, Amr Hossameldin, Amr Muhammad Elkorashy, Amr Tarek Hafez, Amreen Faruq, Amro Aglan, Ana Cecilia Manchego Bautista, Ana Lucia Contreras-Vergara, Ana Maria Sandoval Barrantes, Ana Vega Carreiro De Freitas, Ana Vega Freitas, Anam Rashid, Anan Rady Abdelazeam, Anand Kirishnan, Anass Majbar, Anastasia Bamicha, Anastasios Stefanopoulos, Anders Thorell, Andre Das, Andre Dubois, Andre L Mihaljevic, Andre Navarro, Andrea Allegri, Andrea Armellini, Andrea Belli, Andrea Bondurri, Andrea Echevarria Rosas Moran, Andrea Natili, Andrea Ruzzenente, Andrea Simioni, Andreass Haloho, Andrei Tanase, Andrej Kolosov, Andrejus Subocius, Andrew G N Robertson, Andrew Kirby, Andrew Mcguigan, Andrew Spina, Andrey Litvin, Andrius Burmistrovas, Andrius Strazdas, Andy Arenas, Aneel Bhangu, Anele Rudzenskaite, Angel David Pérez Rojas, Angela Dell, Angelica Genoveva Vergara Mejia, Angeline Charles, Angelo Antoniozzi, Angelo Benevento, Angelos Tselos, Angham Solaiman El-Ma'doul, Anjana Sreedharan, Ankur Bhatnagar, Ann Kjellin, Anna Lasek, Anna Maffioli, Anna Powell, Anna Rinaldi, Anna Watts, Annalisa lo Conte, Annamaria Bigaran, Annelisse Ashton, Annisa Dewi Fitriana Mukin, Antanas Gulbinas, Antanas Zadoroznas, Anthonius Santoso Rulie, Anthony Ajiboye, Anthony Avoka, Anthony Chuk-Him Lai, Anthony Davor, Anthony Sander, Antje Oosterkamp, Antoinette Bediako-Bowan, Antonella La Brocca, Antônio Leal, Antonio Nocito, Antonio Ramos-De La Medina, Antonio Taddei, Anwar Atiyeh, Anyomih Theophilus Teddy Kojo, Aoife Driscoll, Apar Shah, Apostolos Vlachogiorgos, April Camilla Roslani, Aram Abdelhaq, Arazzelly del Pilar Paucar, Arcangelo Picciariello, Areej Tarek, Arezo Kanani, Ari Leppäniemi, Arianna Birindelli, Arij Ibrahim, Arjun Nesaratnam, Arlindawati Suyadi, Armando José Román Velásquez, Arnaud Bonnard, Arnav Agarwal, Aroub Alkaaki, Arturas Vaicius, Arvin Khamajeet, Arvo Reinsoo, Arwa Abouzaid, Arwa Elfarargy, Arwa Ibrahim, Arwa Mohamed, Asaf Kedar, Asdaq Ahmed, Ased Ali, Aseel Alnusairat, Aseel Hamarshi, Aseel Musleh, Ash Prabhudesai, Ashraf A. Maghrabi, Ashraf Morsi, Ashrarur Rahman Mitul, Asmaa Abdelgelil, Asmaa Abdel-Rahman Al-Aarag, Asmaa Rezq, Asmaa Salah, Aspasia Papailia, Assmaa Badwy, Astrid Leusink, Ata Khan, Ataa Ahmed, Athanasia Bamicha, Athar Eysa, Athirah Zulkifli, Atif Mahdi, Attia Attia, Attia Mohamed Attia, Audrey Clarissa, Audrius Dulskas, Audrius Parseliunas, Augusto Zani, Aung Kyaw Tun, Aurel Mironescu, Aurel Sandu Mironescu, Aurelien Scalabre, Aurora Mariani, Aurore Haffreingue, Aurore Thollot, Ausrine Usaityte, Austė Skardžiukaitė, Awais Raza, Aya Abdel Fatah Ibraheem, Aya Aboarab, Aya Adel Elsharkawy, Aya El-Sawy, Aya Elwaey, Aya Firwana, Aya Hagar, Aya Hammad, Aya Mohamed Fathy, Aya Reda, Aya Yehia Ata, Ayah Hamdan, Ayat Hassaan, Ayman And Taher, Ayman Elwan, Ayman Nabawi, Ayman Salman, Ayman Shwky, Ayokunle Ogunyemi, Azher Herebat, Azmina Verjee, Babajide Adenekan, Babatunde Odeyemi, Badr Eldin Adel, Badreldin Adel Tawfik, Bahar Busra Ozkan, Bakeer Mohamed, Bakhtiar Nighat, Bandar Albeladi, Bárbara Málaga, Barbara Mijuskovic, Barbara Pereira Silvestre, Basant Kumar, Basem Sieda, Bashir Bello, Basim Alghamdi, Basma Magdy, Basma Mahmoud, Basmah Alhassan, Bassant Mowafy, Beatrice Brunoni, Beatrix Weber, Belen Sanchez, Ben Thompson, Benoît Parmentier, Bernard Limoges, Bernard Van Duren, Bernhard Wolf, Bertrand Dousset, Besmir Grizhja, Bettina Lieske, Betty Maillot, Billal Mansour, Bjorn Frisk, Bogdan Diaconescu, Bogdan-Valeriu Martian, Boris Marinkovic, Brendan Skelly, Brian Cameron, Britta Dedekind, Bruno Noukpozounkou, Bryon Frankie Hon Khi Chong, Bylapudi Seshu Kumar, Caio Vinícius Barroso de Lima, Calogero Iacono, Cameron Fairfield, Camila Sanchez Samaniego, Camilla Cona, Camilo Lopez-Arevalo, Caoimhe Normile, Caranj Venugopal, Carla Cecilia Ramã­rez Cabrera, Carla Pierina García Torres, Carlo Corbellini, Carlos Alejandro Arroyo Basto, Carlos Iván Pérez Velásquez, Carlos Morales, Carlos Nsengiyumva, Carlos Paz Galvez, Carmen Capito, Carmen Fernández, Carmina Diaz-Zorrilla, Carolina Guzmán Dueñas, Carolina Oliveira Felipe, Caroline Clifford, Caroluce K Musyoka, Catherine A Shaw, Cathy Magee, Cecile Muller, Cecilia Costa, Cecilia Tolg, Cecilia Wredberg, Celeste Del Basso, Céline Grosos, Cesar Augusto Azmitia Mendizabal, Cesar Miranda, Cesar Razuri, Cezar Ciubotaru, Chali Chibuye, Challine Alexandre, Charing Cheuk Ling Szeto, Charles Dally, Charlotte Jane Mcintyre, Chaymae Benyaiche, Chean Leung Chong, Chee Siong Wong, Cheewei Gan, Chelise Currow, Chelsea Deane, Cheng Chun Goh, Cherry Koh, Cheryl Ou Yong, Chetan Khatri, Chi Chung Foo, Chi Ying Jacquelyn Fok, Chia Kong, Chiara Ceriani, Chimwemwe Kwatiwani, Chingwan Yip, Chintya Tedjaatmadja, Choon Seng Chong, Chouikh Taieb, Choy Ling Tan, Chris Bode, Chris Lee, Christel Leanne Almanon, Christian Hinojosa, Christian Lari Coompson, Christin Schoewe, Christina Neophytou, Christina P Major, Christina Panteli, Christine Mizzi, Christoforos Ferousis, Christopher Bode, Christos Agalianos, Christos Anthoulakis, Christos Barkolias, Christos Dervenis, Chu-Ann Chai, Chui Yee Wong, Ciara Fahy, Cicilia Viany Evajelista, Cirugia De Emergencia, Ciskje Zarb, Citra Dewi Mohd Ali, Claire Sharpin, Clara Milagros Herrera Puma, Clare M Rees, Clare Morgan, Claudia Reali, Claudio Arcudi, Claudio Fermani, Claudio Gabriel Fermani, Clemens Nawara, Clement Onuoha, Clodagh Mangan, Colleen Sampson, Collins Nwokoro, Colombani Jean-Francois, Constantinos Marinos, Cornelius Mukuzunga, Corrado Bottini, Craig Gouldthorpe, Crislee Elizabeth Lopez, Cristina Fernandes, Crystal Yern Nee Chow, Cutting Edge Manipal, Dale Vimalachandran, Dalia Alkhabbaz, Dalia Hemeda, Damien Brown, Damir Ljuhar, Dan L Deckelbaum, Dana Jaradat, Danelo Du Plessis, Daniel Ardian Soeselo, Daniel Cox, Daniel Dabessa, Daniel Estuardo Marroquín Rodríguez, Daniel Hamill, Daniel Nel, Daniel Youssef, Daniela Magri, Daniele Angelieri, Daniele Gui, Danilo Herrera Cruz, Danjuma Sale, Dansou Gaspard Gbessi, Dario Andreotti, Darius Kazanavicius, Darragh McCullagh, David Mcnish, David Merlini, David Monterroso Cohen, Davide De Boni, Davide Rossi, Dayang Nita Abdul Aziz, DC Grobler, Debora Schivo, Deborah Chiesa, Deimante Mikuckyte, Deividas Dragatas, Demi Gray, Diaa Eldin Abdelazeem Amin Elsorogy, Diaa Moustafa Elbendary Elsawahly, Diaaaldin Zahran, Diana Duarte Cadogan, Diana Sanchez, Dickson Bandoh, Diego Alonso Romani Pozo, Diego Antezana, Diego Coletta, Diego Romani, Diego Sasia, Dietmar Öfner, Dieudonne Duhoranenayo, Dimitri Aristotle Raptis, Dimitrios Balalis, Dimitrios K Manatakis, Dimitrios Karousos, Dimitrios Korkolis, Dimitrios Kyziridis, Dimitrios Lytras, Dimitrios Papageorgiou, Dimitrios Sfougaris, Dimitris-Christos Zachariades, Dina Al-Marakby, Dina Faizatur Rahmah, Dina Gamal, Dina Tarek, Dineshwary Periasammy, Diogo Vinicius dos Santos, Dion Morton, Diya Mirghani, Djifid Morel Seto, DM Cocker, Dmitri A Raptis, Dmitri Nepogodiev, Dmitri Raptis, Doaa Emadeldin, Doaa Gamil, Doaa Hasan, Doaa Hasanain, Doaa Maher Abdelrouf, Domenica Pata, Domingos Mapasse, Dominic Charles Marshall, Donal B O’Connor, Donatas Danys, Donatas Venskutonis, Dorota Radkowiak, Doug Bowley, Dovilè Majauskyté, Dulan Irusha Samaraweera, Durvesh Lacthman Jethwani, Dush Iyer, Dushyant Iyer, Dzianis Khokha, Dzmitry Paulouski, Ebenezer Takyi Atkins, Echaieb Anis, Edgar Domini, Edilberto Temoche, Eduardo Huaman, Edvard Grisin, Edvinas Dainius, Efeson Thomas, Egle Preckailaite, Ehab Alnawam, Ehab Mamdouh, Eirik Kjus Aahlin, Eirini Kefalidi, Ekow Mensah, Elaine Borg, Eldaa Prisca Refianti Sutanto, Eleanor Marks, Elena Goldin, Elena Muzio, Elena Vendramin, Elena Zdanyte Sruogiene, Eleonora Ciccioli, Elio Jovine, Elisa Francone, Elisabeth Jensen, Elissa Rifhan Mohd Basir, Elizabeth Snyder, Ella Teasdale, Elliot Akoto, Elodie Gaignard, Elodie Haraux, Elsa Robert, Elsayed Ali, Elsayed Gamaly, Emad Abdallah, Emad Al-Dakka, Emad Ali Ahmed, Emad Aljohani, Emad Mohamed Saeed Taha, Eman Abd Al Raouf, Eman Abdelmageed, Eman Abuqwaider, Eman Adel Sayma, Eman Elwy, Eman Emara, Eman Hashad, Eman Ibrahim, Eman Magdy, Eman Magdy Hegazy, Eman Mahmoud Abdulhakeem, Eman Mohamed Ibrahim, Eman Mohamed Morshedy, Eman Nofal, Eman Rashad, Eman Yahya Mansor, Emanuel Barrios, Emanuele Rausa, Emeka Nwabuoku, Emilia De Luca, Emilie Eyssartier, Emilio Dijan, Emma Blower, Emma Jurdell, Emma Upchurch, Emmanuel Acquah, Emmanuel Akatibo, Emmanuel Barrios, Emmy Runigamugabo, Enas Alaloul, Enas Alqahtani, Enoch Dagoe, Enoch Tackie, Eriberto Farinella, Eric Ackom, Eric Kofi Appiah, Erick Samuel Florez Farfan, Erik Hervieux, Erik Schadde, Erika Vicario, Erikas Laugzemys, Ernest Yemalin Stephane Ahounou, Eslam Elbanby, Eslam Ezzat, Esraa Abd Elkhalek, Esraa Abdalmageed Kasem, Esraa Alm Eldeen, Esraa El-Gizawy, Esraa Elhalawany, Esraa El-Taher, Esraa Gamal, Esraa Ghanem, Esraa Kasem, Esraa Samir Elbanby, Esraay Zakaria, Ethar Hany, Etienne Courboin, Eu Xian Lee, Euan Macdonald, Eugene Niyirera, Eugenio Grasset, Eugenio Morandi, Eugenio Panieri, Eva Borin, Evangelos Voulgaris, Evelina Slapelyte, Evelina Woin, Ewan Macdermid, Ewen M Harrison, Eyad Khalifah, Ezio Veronese, Fabian Deichsel, Fabrizio Aquilino, Fahd Abdel Sabour, Faisal Idris, Faith Qi Hui Leong, Fanjandrainy Rasoaherinomenjanahary, Farah Mahmoud Ali, Farhana Iftekhar, Farrag Sayed, Fatai Balogun, Fatema Al Bastawis, Fatema Asi, Fathee Nada, Fathi Elzowawi, Fathia Abd El-Salam, Fathy Sroor, Fatima Baluch, Fatimah I Elgendy, Fatma Elkady, Faustin Ntirenganya, Fawzia Abdellatif Elsherif, Fawzy Mohamed, Fayez Elian Al Barrawi, Fazlin Noor, Federica Bianco, Federica Falaschi, Federico Coccolini, Fei Zheng, Felipe Zapata, Felix Alakaloko, Felix Lee, Feng Yih Chai, Ferdy Iskandar, Fernanda Altoe, Fernanda Frade, Fernande Djivoh, Fernando Espinoza, Fernando Fernandez-Bueno, Fernando Tale, Ferry Fitriya Ayu Andika, Fidelis Jacklyn Adella, Filippo Di Franco, Finaritra Casimir Fleur Prudence Rahantasoa, Fitjerald Henry, Fitriana Nur Rahmawati, Florence Dedey, Florian Primavesi, Florin-Mihail Iordache, Fong Yee Lam, Foteini Koumpa, Francesca Steccanella, Francesco Pata, Francesco Riente, Francesco Ruben Giardino, Francesco Selvaggi, Francis Abantanga, Francis Dossou, Francisco Fujii, Francisco Regalado, Francois-Coridon Helene, Françoise Schmitt, Frank Enoch Gyamfi, Frank Owusu, Fred Alexander Naranjo Aristizã¡bal, Fred Hodonou, Frederick Du Toit, Frederique Sauvat, Fredrik Wogensen, Frehun Ayele Asele, Fridiz Saravia, Gabriel Pardo, Gabriela Elisa Nita, Gaetano Gallo, Gaetano Luglio, Gaetano Tessera, Galaleldin Abdelazim, Gamal Shimy, Gandau Naa Barnabas, Garba Samson, Gareth Irwin, Gehad El Ashal, Gehad Samir El Sayed, Gehad Tawfik, Gemma Humm, Genoveffa Balducci, George Christian Manrique Sila, George Ihediwa, George Manrique Sila, Georges Azzie, Georgette Marie Camilleri, Georgios Gemenetzis, Georgios Gkiokas, Georgios Karabelias, Georgios Kyrou, Georgios Tzikos, Gerardo Perrotta, Gerfried Teufelberger, Germain Ahlonsou, German Minguez, Geta Maharaj, Gezim Galiqi, Ghada Elhoseny, Ghada Saied Nagy, Ghiath Al Saied, Ghina Shamim Shamsi, Giacomo Nastri, Giacomo Pata, Gianfranco Cocorullo, Gianluca Curletti, Gianluca Pagano, Gianluca Pellino, Gianmaria Confalonieri, Gianpiero Gravante, Giedrius Lauzikas, Giles Dawnay, Gintaras Simutis, Giorgio Vasquez, Giovanni Landolfo, Giovanni Lazzari, Giovanni Luca Lamanna, Giovanni Pascale, Giovanni Pesenti, Giovanni Sgroi, Giridhar H Devadasar, Gisele Moreira, Giuliano Borda-Luque, Giuseppe Clerico, Giuseppe Rotunno, Giuseppe Salamone, Giuseppe Sammarco, Gokhan Lap, Greg Padmore, Gregorio Tugnoli, Gregory Kouraklis, Greta Mclachlan, Greta Wood, Greta Žiubrytė, Guillaume Podevin, Guillermo Sanchez Rosenberg, Guo Liang Yong, Gurdeep Singh Mannu, Gurpreet Singh Banipal, Gustavo Miguel Machain Vega, Gustavo Peixoto Soares Miguel, Gustavo Pereira Fraga, Gustavo Recinos, Gustavo Rodolfo Pertersen Servin, Haaris A. Shiwani, Hadeel Al-farram, Hafiz Hakim, Hagar Zidan, Hager Abdul Aziz Amin, Hager Abdulaziz, Hager Ahmed El-badawy, Hager Elwakil, Hager Tolba, Hagir Zain Elabdin, Haidar Hajeh, Hala Ahmed, Hala Saad, Halima Aliyu, Hamdi Ebdewi, Hamza Asumah, Hamza Waleed, Hanan Adel Saad, Haney Youssef, Hani Natalie, Hanna Royson, Hannah Anderson-Knight, Hannah Burns, Hannah S Thomas, Hans-Ivar Pahlsson, Harish Neelamraju Lakshmi, Harriet Jordan, Hasan Ismael Ibraheem, Hasan Ismael Ibraheem Al-Hameedi, Hasbi Maulana Arsyad, Hasnain Abbas Dharamshi, Hassan Ali Mostafa, Hatem El-Sheemy, Haya Tahboub, Hayam Ahmed, Hayden Kretzmann, Hayssam Rashwan, Haytham Abudeeb, Hazem Khaled, Hazmi Dwinanda Nurqistan, Heather Bougard, Heba Baraka, Heba Gamal, Heba Shaker, Hector Shibao Miyasato, Helen Mohan, Helen Woodward, Helena Franco, Helene Francois-Coridon, Helmut Alfredo Segovia Lohse, Hend Adel Gawad Shakshouk, Hend Kandil, Hend Mahmoud, Henri Kotobi, Henry Mendel, Henry Nnaj, Herlin Karismaningtyas, Herman Cruz, Hesham Magdy, Hesham Mohammed Bakry, Hian Ee Heng, Hildur Thorarinsdottir, Hisham Safa, Hisham Samih, Hogea Mircea, Hong Kong SAR, Hong Yee Wong, Hoong-Yin Chong, Hope Edem Kofi Kordorwu, Hope Glover-Addy, Horacio Paredes Decoud, Hosni Khairy Salem, Hossam Dawoud, Hossam Elfeki, Hossam Emadeldin, Houda Bachri, Hunain Shiwani, Hussein Ali, Hussein El-Kashef, Hussein Mohammed, Hussien Ahmed, Iason-Antonios Papaskarlatos, Ibrahem Abdelmotaleb, Ibrahim AbdelFattah, Ibrahim Alhabli, Ibrahim Al-Slaibi, Ibrahim AlYoussef, Ibrahim Elzayat, Ibrahim Elzayyat, Ibrahim N. Alomar, Ibrahim Rakha, Ibrahim Raza, Ida Björklund, Idelso Vasquez, Ignas Rakita, Ihab Hassan, Ihdaa Adawi, Iloba Njokanma, Iman Elkadsh, Immacolata Iannone, Ingemar Havemann, Ioannis Kyriazanos, Ioannis Patoulias, Ioannis Valioulis, Ionasc Dan, Ionut Negoi, Ionut-Bogdan Diaconescu, Irene Montes, Irene Ortega-Vazquez, Isaac Amole, Isaac Bertuello, Isaac Hanley, Isam Bsisu, Islam Magdy El Sayed, Ismael Isaac Zelada Alvarez, Ismail Lawani, Israa Abdullah Aziz Al-Azraqi, Israa Adel, Israa Awad, Israa Qawasmi, Ivan Mendoza Restrepo, J Edward Fitzgerald, Jack Almy, Jacqueline Sheehan, Jaime Andres Montoya Botero, Jaime Herrera-Matta, Jakeline Restrepo, Jakov Mihanovic, James Adeniran, James Brown, James Davies, James Giles, James Glasbey, James Olivier, James Pape, James Richards, James Wheeler, James Yang, Jamie Shah, Janet Pagnozzi, Jannin Salcedo, Jasim Amin, Jason Brown, Javier Pastora, Javier Rosales, Jazmin Coronel, Jean Bréaud, Jean De La Croix Allen Ingabire, Jean-Baptiste Marret, Jean-Francois Colombani, Jean-François Lecompte, Jeffrey Dalli, Jehad Hassan Youssif, Jehad Meqbil, Jemina Onimowo, Jen Cornick, Jenifa Jeyakumar, Jennifer Nowers, Jennifer Rickard, Jennifer Skehan, Jerry Makama, Jesse Ron Swire Ting, Jessica Juliana Tan, Jessica Patricia Gonzales Stuva, Jessica Roth, Jessica Souza Luiz, Jia Hao Law, Jia Yng Siaw, Jian Er Saw, Jibran Abbasy, Jiheon Song, Jimy Harold Jara Quezada, Joachim Amoako, Joachim Wiborg, Joanna Swann, Jo-Anne Carreira, Joanne Edwards, Joe Vincent, Joel Kin Tan, Joe-Nat Clegg-Lamptey, Johanna Joosten, Johanna Nyberg, Johannes Kurt Schultz, Johannes Wiik Larsen, John Bondin, John F. Camilleri-Brennan, John Jemuel V. Mora, John Lee Y Allen, John Whitaker, Jolanta Gribauskaite, Jon Arne Søreide, Jon Kristian Narvestad, Jonathan Ajah, Jonathan Dakubo, Jonathan Heath, Jonathan R L Wild, Jonny Setiawan, Jorge Armando Chungui Bravo, Jorge Torres Cardozo, Jose Aguilar-Jimenez, Jose Andres Garcia-Marin, Jose Antonio Cabala Chiong, Jose Costa-Maia, José Hamasaki, José Luis Hamasaki Hamaguchi, Jose Luis Rodicio, Jose María Vergara Celis, José René Arévalo Azmitia, Joselyn Ye, Joseph Awuku-Asabre, Josephine Psaila, Joshua Luck, Joshua Michael Clements, Joyeta Razzaque, Juan Camilo Correa, Juan Carpio, Juan Gouws, Juan Jaime Herrera Matta, Juan Manuel Carmona, Juan Marcelo Delgado, Juana Kabba, Jubran J Al-Faifi, Julia Guasti Pinto Vianna, Julian Camilleri-Brennan, Juliana Menegussi, Julien Leroux, Julien Rod, Juliette Hascoet, Julio Jimenez, Junyeong Oh, Juozas Kutkevicius, Justas Kuliavas, Justas Žilinskas, Justin Chak Yiu Lam, Justus Lando, Ka Hin Gabriel Li, Ka Wai Leung, Kai Yin Lee, Kalangu Kabongo, Kalitha Pinnagoda, Kalon Hewage, Kamau Kinandu, Kamran Faisal Bhopal, Kandasami Palayan, Kareem Dabbour, Kareem Elshaer, Karen Bailey, Karim Hilal, Karl Bonavia, Karolis Lagunavicius, Karolis Varkalys, Kate Cross, Kate Yu-Ching Chang, Katharina Beate Reinisch, Katharine Whitehurst, Katherine Gash, Kathryn Chu, Kathryn Lee, Katie Connor, Katrin Gudlaugsdottir, Kaustuv Das, Kazeem Atobatele, KC Janardha, Kean Leong Koay, Keat-Seong Poh, Keiran David Clement, Keith Sammut, Keith Say Kwang Tan, Kenneth Aaniana, Kenneth Johnson, Kenneth Mealy, Kenneth Thorsen, Kenny Turpo Espinoza, Kent Pluke, Kestutis Strupas, Kevin C. Conlon, Kevin Turpo Espinoza, Khaled Abozeid, Khaled Alhady, Khaled Aljboor, Khaled Dawood, Khaled Hesham Elbisomy, Khaled Ibrahim, Khaled Khattab, Khaled Naser El Deen, Khalid Mahmud, Khalid Qurie, Khalid Salah El-Dien, Khalil Abdul Bassit, Khaoula Boukhal, Khlood Ashour, Kholod Tarek Lasheen, Kholoud Abdelbadeai, Khurram Khan, Khuzaimah Zahid Syibrah, Kieran Atkinson, Kieran Ka Kei Li, Kirsten Lafferty, Kjetil Søreide, Knut Magne Augestad, Kolonia Konstantina, Konstantinos Farmakis, Konstantinos Gasteratos, Kornelija Maceviciute, Kpèmahouton René Keke, Kresimir Zamarin, Kristian Styles, Kristijonas Jasaitis, Kristijonas Jokubonis, Kristina Cassar, Kuet Jun Chung, Kuhaendran Gunaseelan, Kuok Chung Lee, Kurt Carabott, Kwabena Agbedinu, Kwaku Boakye-Yiadom, Kwame Maison, Kwasi Asare-Bediako, Kwasi Kusi, Kyaw Phyo Aung, Kylie Joan-yi Szeto, Kyriakos Psarianos, Laimonas Uščinas, Lalith Asanka Jayasooriya Jayasooriya Arachchige, Lana Abusalem, Larissa Ines Páez Lopez, Lau Wen Liang Joel, Laura Gavagna, Laura Koskenvuo, Laura Lorenzon, Laura Luque, Laurent Fourcade, Lawal Abdullahi, Lawani Ismaïl, Lawrence Bongani Khulu, Layza-Alejandra Mercado Rodriguez, Lee Shi Yeo, Leif Israelsson, Lemuel Davies Bray, Lenin Peña, Leo Licari, Leonardo Solaini, Li Jing Yeang, Liam Henderson, Liam Richardson, Liana Roodt, Lillian Reza, Linas Urbanavicius, Linas Venclauskas, Linda Alvi Madrid Barrientos, Linda Andersson, Ling Wilson, Linn Nymo, Linnea Mauro, Liviu Iuliu Muntean, Liviu Muntean, Ljiljana Jeremic, Lofty-John Anyanwu, Lopna Ahmed Mohamed Ahmed, Lorena Fuentes-Rivera, Lorena Rodriguez, Lorena Solar García, Lorraine Sproule, Lotfy Eldamaty, Luai Jamal, Luana Ayres Da Silva, Lubna Sabeeh, Luc Hervé Samison, Luca Ansaloni, Luca Bortolasi, Luca Turati, Lucia Duinhouwer, Lucian Corneliu Vida, Lucile Fievet, Lucio Selvaggi, Ludwing Alexander Zeta Solis, Luen Shaun Chew, Luigi Bonavina, Luigi Bucci, Luigi Maria Cloro, Luis Alberto Valente Laufer, Luis Barneo, Luis Joaquín García Florez, Luis M. Helguero-Santin, Luis Miguel Alvarez Barreda, Luis Tale, Luisa Giavarini, Luiz Carlos Barros De Castro Segundo, Luiza Sarmento Tatagiba, Lukas Eisner, Lusi Padma Sulistianingsih Mata, Maarten Vermaas, Mabel Amoako-Boateng, Maciej Walędziak, Madan Jha, Madelaine Gimzewska, Mads Gran, Maeve O'neill, Magdalini Mitroudi, Magnus Boijsen, Maha Al-faqawi, Maha Elmasry, Maha Gamal Mohamad Hamad, Maha Nasr, Mahadevan Deva Tata, Mahitab Essam, Mahitab Morsy Farahat, Mahmoud A. Elnajjar, Mahmoud Abdelshafy, Mahmoud Abdelshakour, Mahmoud Abdulgawad, Mahmoud Ahmed Fathi Abozyed, Mahmoud Alrahawy, Mahmoud Amreia, Mahmoud Badawy, Mahmoud Eldafrawy, Mahmoud Elfiky, Mahmoud Elkhadragy Maher, Mahmoud Elkhadrawi, Mahmoud Elsayed Moghazy, Mahmoud Gomah, Mahmoud M. Saad, Mahmoud Mohamed Metwally, Mahmoud Morsi, Mahmoud Saad, Mahmoud Saami, Mahmoud Salama, Mahmoud Salma, Mahmoud Shalaby, Mahmoud Warda, Mahmoud Zakaria, Mahmut Arif Yuksek, Mahnoor Javaid, Mahnuma Mahfuz Estee, Mai Ebidy, Mai Mohamed Ebidy, Mai Salama, Maíra Cassa Careta, Maja Marcus, Majd Dabboor, Majed Aboelella, Makafui Dayie, Makki Elsayed, Malcolm Falzon, Maleeha Hassan, Malin Sund, Man Fung Leung, Man Hon Andrew Yeung, Manar Abd-Elmawla, Manar Saeed, Mantas Drungilas, Mantas Jokubauskas, Mantas Vilčinskas, Manuel Francisco Roxas, Manuel Hache-Marliere, Manuel Lopez, Manuel Rodriguez Castro, Manuela Mendez, Manzoor Dar, Maram Abu-toyour, Maram Salah, Marcelo O´Higgins Roche, Marco Catani, Marco Maria Pascale, Marco Migliore, Mardelangel Zapata Ponze De Leon, Margaret O'Shea, Margarita Montrimaite, Margherita Notarnicola, Margub Hussain, Maria Clara Mendoza Arango, Maria Giovanna Grella, Maria Hjertberg, Maria Isabel Villegas Lanau, Maria Jesusa B. Maño, Maria Lorena Aguilera, Maria Marta Modolo, Maria Mayasari, Maria Novella Ringressi, Maria Soledad Gonzales Montejo, Maria Soledad Merlo, Maria Utter, María Valcarcel-Saldaña, Maria-Lorena Aguilera-Arevalo, Mariam Darweesh, Mariam O. Gad, Mariam Saad Aboul-Naga, Mariano Cesare Giglio, Mariastella Malavenda, Marie Carmela Lapitan, Marie Dione Parreno-Sacdalan, Marie Paul, Mariette Renaux-Petel, Marija Agius, Marilia Del Carmen Escalante Salas, Marilla Dickfos, Marina Luiza Pimenta, Mario Contreras Urquizu, Mario Corbellino, Mário Jacobe, Mario Lopez, Mario Pasini, Mario Trompetto, Marisa Leal, Marisol Manriquez-Reyes, Mariuca Popa, Mark Ian Hampton, Mark Sykes, Mark Wagener, Markus Zuber, Marte Bliksøen, Martha Glynn, Martin Jarmin, Martin Kyereh, Martina Perino, Martina Yusuf Shawky, Martinique Vella-Baldacchino, Marvin Vargas, Marwa Altarayra, Marwa Elashmawy, Marwa Elshobary, Marwa Hamdan, Marwa Sayed, Marwan Abubakr, Marwan Fahim, Marwan Shawki, Maryam Ali Khan, Maryna Shubianok, Mashael Al-Mousa, Masood Alghamdi, Masood Jawaid, Massiell Machaca, Massimiliano Dal Canto, Massimo Coletti, Matas Pažuskis, Matei Bratu, Matei Razvan Bratu, Mateusz Rubinkiewicz, Matteo Papandrea, Matteo Ripa, Mattew Ekow, Matthew Baldacchino, Matthew Billy, Matthew Young-Han Kim, Matthieu Peycelon, Matti Tolonen, Maureen Bezzina, Maurizio Foco, Mawaddah Alrajraji, Max Dénakpo, Max Rath, Mayaba Maimbo, Mazed Mohamed, Mazen Hassanain, Megan Turner, Mehmet Ali Yavuz, Mehmet Gumar, Mehmet Uluşahin, Melanie Castro Mollo, Melanie Zapata Ponze De Leon, Menatalla Salem, Mengistu Worku, Menna Tallah Ramadan, Mennaallah Hafez, Mennat-Allah Mustafa, Menold Archee P. Redota, Meran Allam, Meric Mericliler, Merna Mostafa, Meryem Abbouch, Metwally Aboraya, Michael Amoah, Michael Cox, Michael Edye, Michael Gillespie, Michael Hanrahan, Michael Livingston, Michael Puttick, Michael Stoddart, Michael Van Niekerk, Michael Walsh, Michael Wilson, Michail Kontos, Michail Margaritis, Michał Janik, Micheal Ohene-Yeboah, Michela Monteleone, Michele Carlucci, Michele Sacco, Michelle Mccarthy, Midhun Mohan, Miguel Angel Paludi, Miguel Siguantay, Mihael Radic, Mihaela Vartic, Miklosh Bala, Milaksh Kumar Nirumal, Milan Radojkovic, Milica Nestorovic, Millika Ghetia, Mindaugas Kiudelis, Mircea Beuran, Mircea Hogea, Mirko Mangiapane, Mitchelle Solange De Fã Tima Linares Delgado, Moayad Othman, Mobolaji Oludara, Modise Zacharia Koto, Mohamad Baheeg, Mohamad Bakhaidar, Mohamad Jeffrey Bin Ismail, Mohamed A Abdelaziz, Mohamed A Amer, Mohamed A Baky Fahmy, Mohamed Abbas, Mohamed Abd El Slam, Mohamed Abdelaty, Mohamed Abdelaty Mohamed, Mohamed Abdelkhalek, Mohamed Abdelraheim, Mohamed Abozaid, Mohamed Abozed Abdullah, Mohamed Abuseif, Mohamed Adel Badenjki, Mohamed Ali Ghonaim, Mohamed Ali Mahmoud, Mohamed Ameen, Mohamed Ammar, Mohamed Asal, Mohamed Awad Elkarim Hamad Mohamed, Mohamed Dablouk, Mohamed El Halawany, Mohamed Elazoul, Mohamed Elbermawy, Mohamed Elfil, Mohamed Elsehimy, Mohamed Elzayat, Mohamed Etman, Mohamed F Zalabia, Mohamed Fares, Mohamed Fawzy Mahrous Badr, Mohamed Fouad Hamed, Mohamed Gadelkarim, Mohamed Ghoneem, Mohamed Gulamhussein, Mohamed Hafez, Mohamed Hashish, Mohamed Hassab Alnaby, Mohamed Husseini, Mohamed Ibrahim, Mohamed Ismail, Mohamed Karkeet, Mohamed Kelany, Mohamed Mabrouk, Mohamed Magdy, Mohamed Mahmoud, Mohamed Moamen Mohamed, Mohamed Moaty, Mohamed Mostafa, Mohamed Mustafa, Mohamed Nashat, Mohamed Nazir, Mohamed Reda loaloa, Mohamed Rezal Abdul Aziz, Mohamed Sabry Ammar, Mohamed Salah, Mohamed Salah Elhelbawy, Mohamed Seisa, Mohamed Shaalan, Mohamed Sleem, Mohamed Sobhi Jabal, Mohamed Youssef, Mohamed Zidan, Mohamedraed Elshami, Mohammad Abdulkhalek Habeeb, Mohammad Aboraya, Mohammad Adawi, Mohammad Alherz, Mohammad Aliyu, Mohammad Elsayed Omar, Mohammad Ghannam, Mohammad Ghassan Alwafai, Mohammad Mohsin Arshad, Mohammad Rashid, Mohammadasim Amjad, Mohammed Alamoudi, Mohammed Alhendy, Mohammed AlRowais, Mohammed Alsaggaf, Mohammed Alzahrani, Mohammed Bukari, Mohammed Deputy, Mohammed Elgheriany, Mohammed Elsayed, Mohammed Elshaar, Mohammed Elsiddig, Mohammed Firdouse, Mohammed G. Azizeldine, Mohammed Hanafy, Mohammed Ismail, Mohammed Kamal Ismail, Mohammed Mousa, Mohammed Mousa Salem, Mohammed Mustafa Hassan Mohammed, Mohammed Mustafa Mohammed, Mohammed Najjar, Mohammed Nasr, Mohammed Osman, Mohammed Osman Dablouk, Mohammed Saeed, Mohammed Saleh A. Alghamdi, Mohammed Ubaid Alsaggaf, Mohammed Yahia Mohamed Aly, Mohannad Aledrisy, Mojolaoluwa Olugbemi, Mona Hamdy Madkor, Mona Hosh, Mona Rashad, Monica Bassem, Monique Moron Munhoz, Monty Khajanchi, Morgan Haines, Morvarid Ashtari, Mostada Samy, Mostafa Abdelkader, Mostafa Ahmed Bahaa Eldin, Mostafa Allam, Mostafa Gemeah, Mostafa Mahmoud Eid, Mostafa Qenawy, Mostafa Samy, Mostafa Seif, Mostafa Shalaby, Mousa Mustafa, Moustafa Ibrahim Mahmoud, Moustafa R. Aboelsoud, Msafiri Kimaro, Muayad Ahmed Alfarsi, Muhamed M H Farhan-Alanie, Muhammad Adil, Muhammad Alkelani, Muhammad Amsyar Auni Lokman, Muhammad Bin Hasnan, Muhammad Daniyan, Muhammad El-Saied Ahmad Muhammad Gohar, Muhammad Fathi Waleed Omar, Muhammad Habib Ibrahim, Muhammad Mohsin Furqan, Muhammad Rashid Minhas Qadir, Muhammad Saqlain, Muhammad Shawqi, Muhammad Talha Butt, Muhammad Taqiyuddin Yahaya, Muhammad Waqar, Muhammed Masood Riaz, Muhammed Talaat, Muhtarima Haque, Muna Rommaneh, Murad Aljiffry, Murat Karakahya, Musah Yakubu, Muslimat Alada, Mustafa Farhad, Mustafa Mohammed Taher, Muthukumaran Rangarajan, Muwaffaq Mezeil Telfah, Myint Tun, Myranda Attard, Nada Ahmed Reda Elsayed, Nada El-Sagheer, Nada Elzahed, Nada Mohamed Bekhet, Nader Abd El Hamid, Nadia Khalid Abd El-Latif, Nadia Ortiz, Nadin Elsayed, Nadya Johanna, Nahilia Carrasco, Najwa Nadeem, Naomi J Wright, Napoleon Mendez, Narimantas E. Samalavicius, Nashat Ghandora, Nasir Bustangi, Natale Di Martino, Natalie Blencowe, Natalie Redgrave, Nathalie Botto, Nathania Sutandi, Nawal Sadig, Nazmie Kariem, Nebil Behar, Nebiyou Seyoum Abebe, Nebyou Seyoum, Nebyou Seyoum Abebe, Neel Gobin, Neel Limaye, Neerav Aruldas, Nehal Yosri Elsayed Abdel-Wahab, Neil Smart, Nelson Manuel Urbina Rojas, Nelson Msiska, Nerijus Kaselis, Nermeen Soubhy El-Shahat, Nermin M Badwi, Nermin Mohamed Badwi, Nesma Elfouly, Nicholas Phillips, Nichole Starr, Nicola Chetta, Nicola Zanini, Nicolas Henric, Nicole D'aguzan, Nicole Grech, Nicoleta Panait, Nicoletta Leone, Nicolò Falco, Nidhi Gyanchandani, Nigel J Hall, Nihaal Shaikh, Niiarmah Adu-Aryee, Nik Azim Nik Abdullah, Nik Ritza Kosai, Nikica Pezelj, Nikki Green, Nikolaos Gouvas, Nikolaos Ivros, Nikolaos Mitroudis, Nikolaos Nikoloudis, Nikolaos Zampitis, Nithya Niranjan, Niveshni Maistry, Noha Abdullah, Noha Abdullah Soliman, Noha Maraie, Noha Wael, Nohad Osman, Noman Shahzad, Nora Abdul Aziz, Norah Al Subaie, Noran Abdel-Hameed, Noran Halim El Gendy, Norbert Uzabumwana, Norberto Herrera, Norma Depalma, Nosisa Sishuba, Nouf Akeel, Noura A. Attallah, Nourhan Adam, Nourhan Anwar, Nourhan Elsabbagh, Nourhan Medhat Elhadary, Nourhan Mesbah, Nourhan Semeda, Nourhan Soliman, Novia Adhitama, Nowrin F. Aman, Nuno Muralha, Nur Zulaika Riswan, Nurlaila Ayu Purwaningsih, Nyawira Ngayu, Octavio Garaycochea, Oday Halhouli, Ogechukwu Taiwo, Ola Sherief Abd El Hameed, Olabisi Osagie, Olabode Oshodi, Olajide Abiola, Olalekan Ajai, Oliver Warren, Oliver Ziff, Olivier Abbo, Olivier Azzis, Olivier Rosello, Olubukola Faturoti, Olufemi Habeeb, Olumide Elebute, Oluseyi Ogunsua, Oluwaseyi Adebola, Oluwatomi Odutola, Omar Abdelkader, Omar Abdulbagi, Omar Aguilera, Omar Alahmady, Omar Arafa, Omar Ghoneim, Omar Hesham, Omar Mattar, Omar Moussa, Omar Osman, Omar Salah, Omar Saleh, Omnia Aboelmagd, Omnia Mosalum, Omobolaji O Ayandipo, Omolara Faboya, Omolara Williams, Opeoluwa Adesanya, Orestis Ioannidis, Osaid H. Alser, Osama Algohary, Osama Mohamed, Osama Mohamed Salah, Osama Mokhtar Mohamed Hassan, Osama Saadeldeen Ebrahim, Osama Seifelnasr, Osman Imoro, Ossama Al-Obaedi, Otto Coyoy-Gaitan, Ourdia Bouali, Owusu Emmanuel Abem, Oyediran Kehinde Timothy, Oyindamola Oshati, Pablo Ramazzini, Pål Aksel Næss, Pamphile A Assouto, Panchali Sarmah, Pandi Eduard, Panu Mentula, Paola Salusso, Paola Violi, Paolino De Marco, Paolo Aonzo, Paolo Silvani, Paolo Ubiali, Patrizio Mao, Paul Kielty, Paul Sutton, Paul Ugalde, Paul Witherspoon, Paul Wondoh, Pauline Gastaldi, Paulius Karumnas, Paulius Kondrotas, Paulo Alves Bezerra Morais, Pedro Angel Toribio Orbegozo, Peep Talving, Pei Ying Koh, Per Weber, Per-Olof Lundgren, Peter Deutschmann, Peter Labib, Peter Wiel Monrad-Hansen, Petras Višinskas, Phebe Anggita Gultom, Philip Alexander, Philip Choi, Philip Mshelbwala, Philip Taah Amoako, Philippe Buisson, Phoebe De Bono, Phumudzo Ndwambi, Pier Paolo Grandinetti, Piergiorgio Danelli, Pierpaolo Sileri, Pietra Ligure, Pietro Mingrone, Pigeneswaren Yoganathan, Piotr Major, Poddevin Francois, Povilas Ignatavicius, Povilas Mazrimas, Prasad Pitigala Arachchi, Pratik Jain, Prince Kwakyeafriyie, Prisca A.L. Har, Pui Xin Chin, Puneet Malik, Puyearashid Nashidengo, Qinyang Liu, Quentin Alimi, Quentin Ballouhey, Quinn Ellison, R. Goh Ern Tze, Rachel King, Rachel Moore, Radhian Amandito, Radin Mohd Nurrahman Radin Dorani, Rafael Araujo, Rafael Soley, Rafał Roszkowski, Raffaele Galleano, Ragavan Narayanan, Ragnar Herikstad, Rahma Kamil, Rajeev Satoskar, Rakan Kabariti, Ralph F Staerkle, Ram Nataraja, Ramadan Oumer, Ramadan Shaker, Ramdan Shaker, Ramesh Jonnalagadda, Ramon Alvarado Jaramillo, Ramón Augusto Melo Cardozo, Rana Mamdouh, Rana Saadeh, Raquel Rodríguez-Uría, Raquillet Claire, Rasha Abdelhamed, Razvan-Matei Bratu, Reda Žilinskienė, Redouane Mammar Bennai, Reem Alyahya, Reem Fakher, Reem Husseiny, Reem Khreishi, Reem Mohammed Hassan Balila, Rehab Elashry, Reham Alaa El-Din, Reham Alshareef, Reham Saad, Renato Melo, Reuban D'cruz, Reuben Goh Ern Tze, Reynu Rajan, Rezaul Karim, Ricardo Velasquez, Richard Gilbert, Richard Lilford, Richard Opoku-Agyeman, Richard Spence, Richard William Gilbert, Richmond Hagan, Rifan Alyami, Riinu Ots, Ritauras Rakauskas, Roaa Khan, Robert George, Robert Karlo, Robert Kerley, Robert Mcintyre, Robert Morton, Robert Parker, Robert Tyler, Roberta Bugeja, Roberta Tutino, Roberta Villa, Robertas Baltrunas, Robertas Pranevicius, Roberto Cautiero, Roberto Cirocchi, Roberto Faccincani, Roberto Klappenbach, Roberto Macchiavello, Roberto Peltrini, Roberto Schiavone, Robinson Mas, Roel Matos-Puig, Rofida Elsemelawy, Roger Lawther, Roger Schmid, Rohan Ardley, Rohi Shah, Rokas Rackauskas, Rokayah Julaihi, Rokia Sakr, Roland Osuoji, Romeo Guevara, Romeo Lages Simoes, Romualdas Riauka, Ronald Coasaca Huaraya, Ronald Renato Barrionuevo Ojeda, Ronan Cahill, Rony Camacho, Rory Callan, Rosario Sacco, Rose Khreishi, Rosie Mcdonald, Ross Bowe, Ross Coomber, Rowida Elmelegy, Roxanne Chenn, Roy Quek, Rubén Balmaceda, Rubén Darío Arias Pacheco, Ruben Rivas, Ruben Santiago Restrepo Giraldo, Rudy Gunawan, Rula Zaa'treh, Ruqaya Kadhim Mohammed Jawad Al-Hasani, Ruta Mazelyte, Ruth Blanco, Ruth Gratton, Ruth Scicluna, Ryan Adams, Ryan Choon Kiat Tan, Ryan Mcintosh, S.V. Kinnera, Saad Al Awwad, Sabbir Karim, Sabine Irtan, Sabrina Asturias, Sabrina Dardenne, Sabry Mohy Eldeen Mahmoud, Safia Ali, Safwat Al-Nahrawi, Saged Elsherbiney, Sahar Abdoun Ishag Idris, Sahar Jaber, Sahlu Wondimu, Saiba Abdul-Latif, Said Alyacoubi, Sakhaa Hanoun, Saleem El-Rabaa, Saleh A. Alnuqaydan, Saleh Alqahtani, Salim Anderson Khouri Ferreira, Sally Elshanwany, Sally Hallam, Salma Magdy, Salma Mansour, Salma Said Elkolaly, Salman Aldhafeeri, Salomone Di Saverio, Salwa Khallaf, Sam Arman, Sam Debrah, Sam Seisay, Samaa Mahmoud Al Attar, Samah Afana, Samantha Corro-Diaz Gonzalez, Samar Abdelhady, Samar Adel Ismail, Samar Saad, Samar Soliman, Sameer Kushwaha, Sameh Emile, Sameh Sarsik, Sami Martin Sundstrom, Samson Olori, Samuel Essoun, Samuel Nigo, Samuel Osei-Nketiah, Samuel S. Y. Sii, Samuel Sani Ali, Sandip Kumar, Sandra Ahlqvist, Sandrine Kwizera, Sandro Pasquali, Sani Ali Samuel, Sanju Sobnach, Santiago Villalobos, Sara Abd Elmageed Barakat, Sara Ahmed, Sara Al-saqqa, Sara Amr Mohamed Farouk, Sara Arafa, Sara Ayad, Sara Elhamouly, Sara Etienne, Sara Ghanem, Sara Kharsa, Sara Mahmoud Abdel-Kader, Sara Mamdouh Matter, Sara María Contreras Mérida, Sara Mehrez, Sara Mohammed, Sara W Al-Saqqa, Sarah Abdelghany, Sarah Antar, Sarah Benammi, Sarah Braungart, Sarah Hafez, Sarah Rayne, Sarah Sahel, Sarah Samy, Saraibrahim Ahmed, Saskia Highcock, Saud Aljohani, Saulius Bradulskis, Saulius Mikalauskas, Savino Occhionorelli, Savni Satoskar, Sawsan Adel Awad, Sayed Sarwary, Sayeda Nazmum Nahar, Sayeeda Aktar Tori, Sayinthen Vivekanantham, Scott K D'amours, Sean Mizzi, Sebastian Bernardo Shu Yip, Sebastian King, Sebastian Shu, Sebastian Sierra, Sebastien Gaujoux, Sebestian Shu, Sefeldin Mahdi, Selina Chiu, Selina Man Yeng Chiu, Semay Desta, Serena Manfreda, Serge Kapenda Tshisola, Sergio Estupinian, Sergio Ribaldi, Sergio Zegarra, Servio Tulio Torres Rodriguez, Shadid Al Amin, Shadid Alamin, Shady Elhadry, Shady Hussein, Shady Mahmoud, Shagorika Talukder, Shahadatul Shaharuddin, Shahinaz Alaa El-Din, Shaimaa Aql, Shalon Guevara Torres, Shamsudeen Aliyu, Sharad Karandikar, Sharon Koh, Shaza Rabie Mohamed, Shereen Elsheikh, Sherif Shehata, Sherif Tariq, Shimaa Gamal, Shimaa Said Elkholy, Shireen Gaafar, Shirish Tewari, Shiva Dindyal, Shivanee Tharmalingam, Shorouk El Mesery, Shpetim Ymeri, Shravan Nadkarni, Shruti Ayyar, Shu Ning Kong, Shuang Yi Teo, Shyam Gokani, Shyang Yee Lim, Silje Holte, Silvia Basilicò, Silvia Boni, Silvia De Franciscis, Simon George Gosling, Simon Gosling, Simon Ng, Simon Stock, Simona Juciute, Simona Kasputyte, Simone Conci, Simone Sandler, Simone Targa, Sir Young Yam, Siti Mohd Desa Asilah, Siti Nur Alia Kamarulzamil, Sivasuriya Sivaganesh, Siyaka Itopa Suleiman, Siyi Chung, Soaad Elsobky, Sofia Mouttalib, Soha Abushamleh, Sohaila Elmihy, Soliman Magdy Ahmed, Sondos Turkustani, Sophian Hmila, South Africa, Srinivas Pai, Sriram Bhat, SS Prasad, Stassen Paul, Stavros Parasyris, Stefan Botes, Stefan Breitenstein, Stefan Zammit, Stefano Berti, Stefano Cucumazzo, Stefano M.M Basso, Stefano Roncali, Stella Binna Kim, Sten Saar, Stephanie Hiu-wai Kwok, Stephanie Van Straten, Stephen Dias, Stephen J Chapman, Stephen Kache, Stephen Mcaleer, Stephen R Knight, Stephen Tabiri, Steponas Petrikenas, Stuart J Fergusson, Styliani Parpoudi, Stylianos Germanos, Sudipta Roy, Sukrit Suresh, Sule Burger, Suleiman Baba, Sultan Almuallem, Sung-Hee Kim, Sunil Kumar, Suparna Das, Suraya Bahar, Susan Aviles, Susan Limache, Susan Wndy Mathew, Susana Yrma Aranzabal Durand, Svetlana Doris Brincat, Swantje Kruspi, Swapnil Roy, Syed Abdul Wahhab Eusoffee Wan Ali, Syed Altaf Naqvi, Syed Asaat ul Razi, Sylvia Batista Lemaire, Sylvie Mochet, Syrine Rekhis, T Ariani Widiastini, Tagang Ebogo Ngwa, Taha Yusufali, Taher Al-taher, Tahir Muhammad Yaseen, Tahir Yaseen, Tahira Naqvi, Taiwo Akeem Lawal, Taiwo Lawal, Tan Arulampalam, Tanzeela Gala, Tapan Kumar, Tara Grima, Tarek Ezzat, Tarek Razek, Tasneem Idress, Tasnia Hamid Kanta, Tatsiana Shachykava, Taufiq Khan, Tebian Hassanein Ahmed Ali, Tessa Fautz, Tewodros Worku, Thamer Nouh, Thays Brunelli Pugliesi, Thea Dimech, Thelma Tembo, Thelma Xerri, Theodore Pezas, Theodosios Theodosopoulos, Thiago Fernandes Giuriato, Thierry Alihonou, Thomas Feidantsis, Thomas Fozard, Thomas G Weiser, Thomas M Drake, Thomas Olagboyega Olajide, Thomas Pinkney, Thomas Prudhomme, Thomas Sherman, Thomas Tetens Moe, Thuraya Alzayat, Thusitha Sampath Hettiarachchi, Tien Seng Bryan Lee, Timothy White, Tina Gaarder, Tobias Schuetz, Todisoa Emmanuella Christina Tolotra, Tolg Cecilia, Tom AM Malik, Tom Arthur, Tom Falconer Hall, Tomas Abaliksta, Tomas Jankus, Tomas Poškus, Tommaso Bocchetti, Tommaso Campagnaro, Tommaso Fontana, Tony Mak, Toqa Khafagy, Torhild Veen, Trude Beate Wold, Tsz-Yan Katie Chan, Tuan Nur'Azmah Tuan Mat, Tunde Sholadoye, TWC Mak, Tyler Rouse, Tzu-Ling Chen, Uday Muddebihal, Ufuk Karabacak, Ulf Gunnarsson, Ulf Gustafsson, Umar Muktar, Umberto Tedeschi, Umme Salma, Usama Hantour, Uthman Alamoudi, Valdemaras Jotautas, Valentine Parent, Vanessa Dina Palomino Castillo, Vanessa Msosa, Vania Guglielmo, Vania Silvestri, Vasileios Despotidis, Vasileios Kalles, Vasiliki Soulou, Vassilis Kalles, Veereanna Shatkar, Venerand Barendegere, Veronica Grassi, Veronica Lazzari, Vicky Jennings, Victor Dassah, Victor Etwire, Victor Kong, Victor Manuel Quintero Riaza, Victor Nwinee, Victoria K Proctor, Vijaid Upadhyaya, Vijay Gadhvi, Viktorija Ambrozeviciute, Viktorija Nevieraite, Ville Sallinen, Vimalakanthan Thanusan, Vincas Jonas Banaitis, Virgilijus Beisa, Viviana Sollazzo, Vivien Graffieille, Vizir Jean Paul Nsengimana, Vladimir Khokha, Vu Thanh Hien Le, Vytautas Gaižauskas, Vytautas Lipnickas, Wahid Anwer, Wai Cheong Soon, Wai Him Lam, Wairimu Ndegwa, Waleed Thabet, Walid Adham, Walter Forno, Walter Ruiz Panez, Wan Nurul ‘Ain Wan Mohd Nasir, Wanigasekara Senanayake Mudiyanselage Kithsiri Janakantha Senanayake, Ward Hamsho, Wasim Dar, Wedyan Alhazmi, Wei Guo, Weiguang Ho, Weihei Dao, Wendy Leslie Messa Aguilar, Wennweoi Goh, Wifanto Saditya Jeo, Wilfredo Pino, William Appeadu-Mensah, William Beasley, William Bonney, William Hutch, William J. Lossius, William Milligan, Willy Alcca Ticona, Wing Sum Li, Witold Chachulski, Xavier Delforge, Xianelle Rodriguez, Xinwei Low, Xue Wei Chan, Ya Theng Neo, Yacoubou Imorou Souaibou, Yahaya Ukwenya, Yahya Salama, Yaseen Rajjoub, Yasmein Ibrahim, Yasmin Abd-Elrasoul, Yasmin Elfouly, Yasmin Hegazy, Yasmin Soliman, Yasser Abd El Salam, Yee Wen Tan, Yehia Zakaria, Yella Reddy, Yi Koon Tan, Yi Ting Mok, Yih Jeng Cheong, Yiing Yee Gan, Yishan Der, Yogendra Praveen Mogan, Yomna Allam, Yomna Hosny Asar, Yong Yong Tew, Yousef Abuowda, Yousra El Shoura, Ysabel Esthefany Alejos Bermúdez, Yücel Cengiz, Yuk Hong Eric Cheung, Yuksel Altinel, Yung Kok Ng, Yuri Macchitella, Yves Aigrain, Zaher Mikwar, Zahra Jaffry, Zain Ali Khan, Zainab Iftikhar, Zaynab M Elsayed, Zhongtao Zhang, Zi Hao Sam, Zigmantas Urniežius, Zilvinas Dambrauskas, Zineb Bentounsi, Zygimantas Tverskis

**Affiliations:** Department of Economics & Centre for Modern Indian Studies, University of Goettingen, Göttingen, Germany; Heidelberg Institute of Global Health, Heidelberg University, Heidelberg, Germany; Department of Economics & Centre for Modern Indian Studies, University of Goettingen, Göttingen, Germany; Institute of Economics, Department of Health Economics, Friedrich-Alexander-Universität Erlangen-Nürnberg, Germany; Institute of Cancer and Genomic Sciences, University of Birmingham, Birmingham, UK; Institute of Cancer and Genomic Sciences, University of Birmingham, Birmingham, UK; Institute of Cancer and Genomic Sciences, University of Birmingham, Birmingham, UK; Institute of Applied Health Research, University of Birmingham, Birmingham, UK; Centre for Global Surgery, Department of Global Health, Stellenbosch University, Cape Town, South Africa; Medical Research Council/Wits University Rural Public Health and Health Transitions Research Unit, Faculty of Health Sciences, School of Public Health, University of the Witwatersrand, Johannesburg, South Africa; Department of Economics & Centre for Modern Indian Studies, University of Goettingen, Göttingen, Germany

## Abstract

**Background:**

There is a substantial gap in provision of adequate surgical care in many low- and middle-income countries. This study aimed to identify the economic burden of unmet surgical need for the common condition of appendicitis.

**Methods:**

Data on the incidence of appendicitis from 170 countries and two different approaches were used to estimate numbers of patients who do not receive surgery: as a fixed proportion of the total unmet surgical need per country (approach 1); and based on country income status (approach 2). Indirect costs with current levels of access and local quality, and those if quality were at the standards of high-income countries, were estimated. A human capital approach was applied, focusing on the economic burden resulting from premature death and absenteeism.

**Results:**

Excess mortality was 4185 per 100 000 cases of appendicitis using approach 1 and 3448 per 100 000 using approach 2. The economic burden of continuing current levels of access and local quality was US $92 492 million using approach 1 and $73 141 million using approach 2. The economic burden of not providing surgical care to the standards of high-income countries was $95 004 million using approach 1 and $75 666 million using approach 2. The largest share of these costs resulted from premature death (97.7 per cent) and lack of access (97.0 per cent) in contrast to lack of quality.

**Conclusion:**

For a comparatively non-complex emergency condition such as appendicitis, increasing access to care should be prioritized. Although improving quality of care should not be neglected, increasing provision of care at current standards could reduce societal costs substantially.

## Teaser

The economic burden due to loss of income from deaths and days off sick of not meeting need for appendicitis surgery. This is estimated by considering the losses from not providing care to the “local standard” or not providing care to the “highest standard” (based on high income country standards). Improving access to care whilst keeping the local standard (or quality) of care would overcome most of the economic losses; improving quality to the highest standard has marginal additional benefits.

## Background

It has been estimated that, each year, 143 million additional surgical procedures need to be done in low- and middle-income countries to prevent disability and reduce mortality^1^. The associated loss of economic productivity has been estimated at $12.3 trillion for the interval 2015–2030^1^. In addition to insufficient access to surgery, it has been recognized that outcomes of surgery can be suboptimal for many patients in low- and middle-income countries. This is reflected in the higher rate of perioperative mortality and surgical-site infections experienced by patients undergoing surgery in low- and middle-income countries compared with those in high-income countries^2,3^. This high-level evidence has been insufficient to prompt large-scale policy change and substantial investment in surgery. The cost to a given society of not providing adequate surgical care for specific conditions might provide direct evidence that more targeted investment in surgical services could be cost-effective.

Surgery is a treatment for many diverse conditions^4^. Although the magnitude of lack of access to quality surgical care has been estimated, developing a health service to provide such holistic surgical care for all conditions may not currently be attainable. It should, however, be within the reach of many countries to provide services to treat some conditions that are otherwise fatal and require a fairly simple procedure^5^.

Appendicitis is a common condition, with an incidence of around 17 700 000 in 2019^6^. Although it leads to death or disability if not treated, timely surgical treatment results in a rapid return to normal function. It disproportionally affects younger populations, who are generally economically productive. Hence, lack of access to surgical treatment for appendicitis is likely to have substantial economic consequences for individuals and societies.

Effective treatment of appendicitis requires appropriate and timely surgery, necessitating ready access to acute services^7^, unlike planned procedures that can be referred to a tertiary centre. Appendicitis can vary in severity from self-limiting infection to life-threatening peritonitis, depending on the development of irreversible, but unpredictable, gangrene. The safest treatment is, therefore, early surgery. Effective surgery for appendicitis is also reflective of local and district surgical services^8^, and improved delivery is likely to have additional benefits for other common surgical conditions.

Estimates are available for the global incidence of appendicitis^6^, the unmet need for surgery^1^, and of harm from lack of access to quality surgery, including procedures for appendicitis^2^. However, there has been no previous estimation of the global economic burden associated with failure to provide access to quality care for appendicitis. Given that access to surgical treatment for appendicitis reflects local surgical care provision, such information is needed to inform discussions on the investment case for provision of surgical care at country, regional, and international levels^1,9^.

This study assessed the economic burden (from loss of income) associated with unmet or delayed or substandard surgical care in low- and middle-income countries. The study focused on two types of indirect cost: lost income owing to premature death and lost income due to absenteeism and/or sick days.

## Methods

This study calculated the economic burden in two scenarios: that resulting from not providing surgery at local standards in low- and middle-income countries; and that resulting from not providing surgical care in these countries at the standard available in high-income countries, which, based on the literature^3^, was assumed to reflect optimal (high-quality) care. The economic burden attributed to scenario 1 could be avoided by increasing the coverage of operations to all who require them while keeping the standard of care in each country the same as it is currently. Scenario 2 involves increasing the coverage of operations to all who require them while increasing the standard of care in each country to be equivalent to that seen in high-income countries. Additionally, the study estimated the total economic burden experienced owing to the current state of care across countries, that is the consequences of providing surgical care of local standard to the proportions of people who currently receive care, and providing no surgical care to those who do not. *Supplementary material S.2* provides a detailed description of the method, and details of the data sources and indicator construction can be found in *supplementary material S.3*. The main analysis was conducted at country level, although some data inputs were available only at the level of country-income groups. The results are presented aggregated to WHO region in the main text. The estimates are presented for the year 2015 as this was the latest year for which data from most sources were available.

### Unmet surgical need

All scenarios required information on the number of individuals with appendicitis who do not receive any surgical care. For this, estimates of the incidence of appendicitis for a given country and 5-year age group in 2015 from the Global Burden of Disease project were used^6^. Data on numbers of appendicectomies performed (or the shortage of appendicectomies) were not available for most countries. Thus, the unmet need was estimated using the following two approaches. The first approach assumed a need for surgical volume of 5000 operations per 100 000 people, for all conditions that should be treated surgically, following the Lancet Commission on Global Surgery^1^. Using data on a country’s total surgical volume delivered from Holmer *et al.*^10^, the gap between surgical need and volume was defined as the unmet need. It was assumed that the proportion of unmet need for appendicitis is equivalent to that for all conditions requiring surgery.

The second approach assumed that appendicectomies comprise a certain share of surgical volume, with that share varying depending on characteristics of the country. This approach was taken based on the knowledge that lower-income countries perform a larger volume of emergency and gastrointestinal procedures (such as appendicectomy), relative to total surgical volume, than high-income countries^11^. Given that data on volume of appendicectomies to surgical volume were not available for most countries, several steps were taken to derive these estimates. First, information on the ratio of appendicectomies to gastrointestinal surgery from 116 countries was taken from the COVIDSurg Collaborative, GlobalSurg Collaborative database^12^. Second, information on the share of gastrointestinal surgery relative to total surgical volume in low- and middle-income countries was taken from multiple publications identified in a systematic search of the literature, as listed in the *supplementary material Table S2*. Data from England’s Hospital Episode Statistics were used as a proxy for data from high-income countries. Data from low-, middle-, and high-income countries are shown in the *Supplementary material* to illustrate how the ratio of gastrointestinal surgery to total surgery varies between World Bank income groups. Although these data were used to calculate the predicted number of appendicectomies for high-income countries, it was assumed that there is no unmet need in such countries; thus, the resulting figures were not included in the estimation. Third, a combination of these sources was used to predict the number of appendicectomies for each country based on its surgical volume from Holmer *et al.*^10^. The gap between a country’s incidence of appendicitis and the predicted number of appendicectomies was then defined as the unmet need.

For both approaches, it was assumed that all patients with appendicitis in high-income countries receive surgical treatment. Organisation for Economic Co-operation and Development (OECD) data for 25 high-income countries in 2015 showed that, for most countries, the number of cases of appendicitis was very close to the number of appendicectomies performed^13,14^.

### Economic burden estimation

**Fig. 1:**
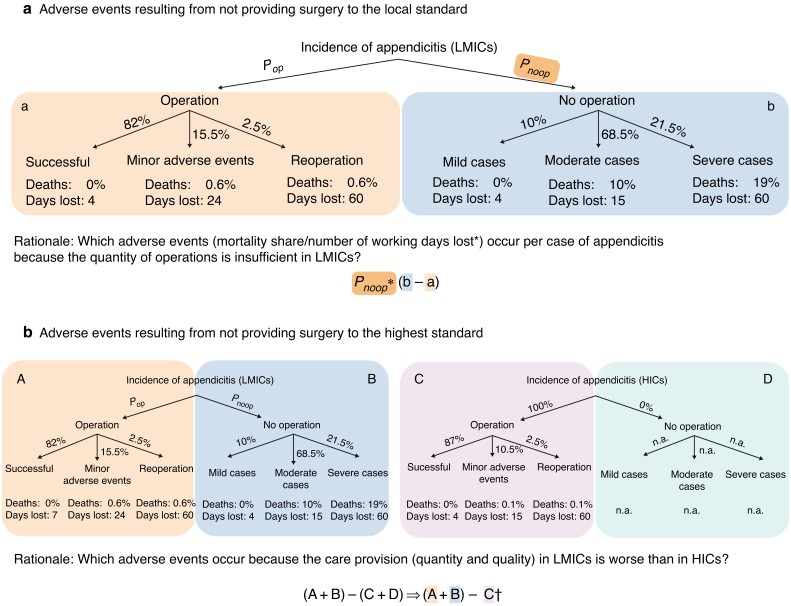
Calculation of expected mortality risk and number of absent days **a** Resulting from not providing surgery to the local standard, and **b** resulting from not providing surgery to the highest standard. *Non-fatal cases. †Assuming that all patients with appendicitis undergo surgery in high-income countries (HICs), such that D = 0. LMICs, low- and middle-income countries; n.a., not applicable.

This study focused on two types of income loss: that resulting from early death and that associated with absenteeism. In the first step, the mortality and absenteeism outcomes of surgical treatment in low- and middle-income countries were estimated using surgical treatment at a standard received in low- and middle-income countries and that in high-income countries (as highest available standard), and outcomes of no surgical treatment at all (described in detail in *the supplementary material Section S2*). Estimates of the probabilities and mortality or absenteeism (days lost) outcomes were based on adverse events of surgically treated appendicitis from the GlobalSurg Collaborative database^3,15^ (exact definitions are available in *supplementary material Table S2*). The outcomes of not surgically treating appendicitis in low- and middle-income countries rely on the literature summarized in the *supplementary material Table S6*. Multiplied by the share of unmet surgical need, this gave the expected mortality risk and the number of days absent from employment resulting from not providing surgery to the local or highest standard to an individual with appendicitis (*Fig. 1*).

In the next step, the expected mortality risk was multiplied by the age-specific incidence of appendicitis to obtain the total number of expected deaths for a given country and age group. The associated income losses were calculated by multiplying the number of expected deaths by the earnings the individuals were expected to have received if they had not died. Similarly, the expected number of absent days was multiplied by the share of unmet surgical need and the incidence of appendicitis to obtain the total number of expected lost working days for a given country. Absenteeism-related income losses were calculated by multiplying the lost working days by the average daily wage. Estimates of the average annual wage for each country were extracted from the International Labour Organization^16,17^ and OECD^18^ databases, and all costs were expressed in US dollar purchasing power parity (PPP), deflated to the year 2015. All costs were aggregated by WHO region to display the main results. Country-level results are available in the *supplementary material Fig. S16, Table S11*.

## Results

*Table 1* shows the key statistics used to calculate the economic burden of unmet surgical need by WHO region. The estimated share of unmet need was higher using the first approach than the second for all regions except the Americas. This was also evident at country level (*supplementary material Fig. S8*).

**Table 1:** Key statistics by WHO region

	Africa	Americas	Eastern Mediterranean	Europe	South-East Asia	Western Pacific	World
**No. of countries**	44	29	18	50	10	19	170
Incidence per 100 000, mean (s.d.)*	184.27 (72.21)	359.73 (185.64)	264.86 (47.48)	232.25 (45.75)	517.36 (526.16)	234.50 (111.59)	262.05 (177.08)
**Surgical volume per 100 000, mean (s.d.)†**	1167 (1305)	5635 (5158)	3305 (2243)	7890 (4463)	1784 (1878)	4637 (6365)	4557 (4767)
**No. of appendicectomies per 100 000 (approach 2), mean (s.d.)‡**	74.17 (62.19)	213.05 (236.18)	112.63 (73.14)	181.78 (137.17)	107.49 (87.45)	132.88 (97.81)	142.10 (142.65)
**Unmet need (%) (approach 1), mean (s.d.)‡**	76.65 (26.10)	24.59 (28.75)	35.48 (35.73)	8.20 (18.18)	67.21 (29.96)	40.23 (36.02)	38.65 (38.14)
**Unmet need (%) (approach 2), mean (s.d.)‡**	60.24 (27.19)	33.64 (32.62)	31.08 (31.57)	5.21 (15.18)	58.19 (36.95)	20.36 (28.37)	31.85 (34.15)
**Wage per capita, mean (s.d.)§**	6544 (7450)	17 609 (12 994)	18 437 (12 664)	28 174 (16 895)	6288 (3694)	20 689 (19 966)	17 618 (16 056)
**Total population (millions)¶**	979.14	974.59	659.54	913.32	1921.74	1844.54	7301.06

Approach 1 calculates unmet need for appendicitis assuming that the proportion of this unmet need is equivalent to the unment need for all conditions requiring surgery. Approach 2 calculates unmet need as the relative difference of estimated appendicectomies (calculated as World Bank income group-specific share of surgical volume) to the number of appendicitis cases. *Institute for Health Metrics and Evaluation Global Burden of Disease. †Holmer *et al.*^10^. ‡Detailed description available in *supplementary material S.3*. §Organisation for Economic Co-operation and Development, International Labour Organization (harmonized to 2015 US dollar purchasing power parity). ¶World Bank World Development Indicators. Country-specific inputs are shown in *supplementary material Table S9* and graphs of unmet need by region and income group in *Fig. S8*.

**Fig. 2:**
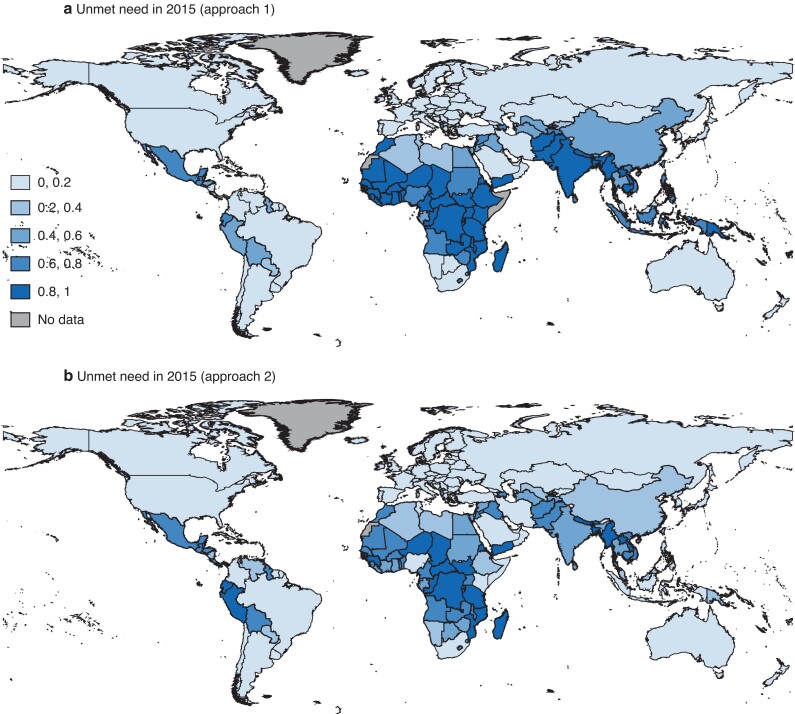
Estimated share of unmet need in 2015 by country Calculated using **a** approach 1 and **b** approach 2. Approach 1 calculates unmet need for appendicitis assuming that the proportion of this unmet need is equivalent to the unment need for all conditions requiring surgery. Approach 2 calculates unmet need as the relative difference of estimated number of appendicectomies (calculated as World Bank income group-specific share of surgical volume) to the number of appendicitis cases.

Country-specific shares of unmet need are shown in *Fig. 2*, which allows a more detailed comparison of the two approaches. For most countries, approach 1 yielded a higher unmet need than approach 2, whereas the reverse applied mostly to Latin American countries (*supplementary material Fig. S8*).

As an intermediate outcome, excess mortality resulting from not providing surgery to local standards was estimated at 4185 per 100 000 patients with appendicitis using approach 1 and 3448 per 100 000 using approach 2 across the whole sample (*supplementary material Table S10*). The rate was highest in Africa (8299 per 100 000 for approach 1 and 6522 per 100 000 for approach 2) and South-East Asia (7277 and 6301 per 100 000, respectively). The mortality rate from not providing surgery at the highest standard was slightly higher, on average 4237 per 100 000 (approach 1) and 3500 per 100 000 (approach 2) across the sample.

The resulting income loss estimates are shown in *Table 2*. Mortality-related income losses resulting from not providing surgery to the current local standard of care amounted to $91 031 million (2015 PPP) for all countries using approach 1, ranging from $1415 million for Europe and $36 637 million for Western Pacific. Using approach 2, the mortality-related income losses totalled $71 984 million, ranging from $889 million in Europe to $24 040 million for South-East Asia. Expressed as a percentage of gross domestic product (GDP), the economic burden ranged from 0.0050 per cent of GDP in Europe to 0.2602 per cent of GDP in South-East Asia using approach 1, and from 0.0031 per cent of GDP in Europe to 0.2144 per cent in South-East Asia using approach 2. The mortality-related income losses associated with not providing surgery to the highest standard of care were slightly higher, but very similar to the income losses of not providing surgery to local standards.

**Table 2:** Economic burden estimates

		Africa	Americas	Eastern Mediterranean	Europe	South-East Asia	Western Pacific	World
**No. of countries**		44	29	18	50	10	19	170
**Mortality-related income losses**								
Not providing surgery to local standard								
Approach 1	US $ (millions)	6219	9777	7808	1415	29 186	36 627	91 031
	% of GDP	0.1781	0.0352	0.1232	0.0050	0.2602	0.1337	0.0870
Approach 2	US $ (millions)	4445	13 316	6819	889	24 040	22 476	71 984
	% of GDP	0.1273	0.0479	0.1076	0.0031	0.2144	0.0820	0.0688
Not providing surgery to highest standard								
Approach 1	US $ (millions)	6305	9969	7919	1619	29 419	37 151	92 383
	% of GDP	0.1806	0.0359	0.1249	0.0057	0.2623	0.1356	0.0883
Approach 2	US $ (millions)	4532	13 508	6930	1094	24 273	23 000	73 336
	% of GDP	0.1298	0.0486	0.1093	0.0038	0.2164	0.0840	0.0701
**Absenteeism-related income losses**								
Not providing surgery to local standard								
Approach 1	US $ (millions)	95	154	122	23	446	622	1461
	% of GDP	0.0027	0.0006	0.0019	0.0001	0.0040	0.0023	0.0014
Approach 2	US $ (millions)	69	206	107	14	372	389	1157
	% of GDP	0.0020	0.0007	0.0017	0.0001	0.0033	0.0014	0.0011
Not providing surgery to highest standard								
Approach 1	US $ (millions)	165	318	214	206	631	1086	2622
	% of GDP	0.0047	0.0011	0.0034	0.0007	0.0056	0.0040	0.0025
Approach 2	US $ (millions)	141	367	200	198	561	863	2330
	% of GDP	0.0040	0.0013	0.0032	0.0007	0.0050	0.0032	0.0022
**Total economic burden**								
Not providing surgery to local standard								
Approach 1	US $ (millions)	6314	9931	7929	1437	29 631	37 249	92 492
	% of GDP	0.1808	0.0358	0.1251	0.0051	0.2642	0.1360	0.0884
Approach 2	US $ (millions)	4515	13 521	6925	903	24 411	22 865	73 141
	% of GDP	0.1293	0.0487	0.1093	0.0032	0.2177	0.0835	0.0699
Not providing surgery to highest standard								
Approach 1	US $ (millions)	6470	10 287	8133	1825	30 050	38 238	95 004
	% of GDP	0.1853	0.0370	0.1283	0.0064	0.2680	0.1396	0.0908
Approach 2	US $ (millions)	4672	13 875	7130	1291	24 834	23 863	75 666
	% of GDP	0.1338	0.0500	0.1125	0.0045	0.2214	0.0871	0.0723

US dollars are expressed in 2015 purchasing power parity. Absolute costs are the sum of the respective country-level costs by region; relative costs are absolute costs divided by the sum of country gross domestic product (GDP) by region. Approach 1 calculates unmet need as the relative difference of surgical volume to the minimum surgical volume proposed by the Lancet Commission on Global Surgery. Approach 2 calculates unmet need as the relative difference of estimated appendicectomies (calculated as World Bank income group-specific share of surgical volume) to the number of appendicitis cases. Country-specific results can be found in *supplementary material Fig. S10, Fig. S11*, and *Table S11*; intermediate results by region and income group in *supplementary material Fig. S9*; and sex-specific results by region in *supplementary material Fig. S12* and *Fig. S13*.

Similar to the mortality-related income losses, absenteeism-related income losses associated with not providing surgery to local standards were lowest in Europe and highest in Western Pacific when estimated using either approach. In Europe, income losses were $23 million for approach 1 and $14 million for approach 2; in Western Pacific, losses were $622 million and $389 million respectively. However, the difference between income losses of not providing surgery to local *versus* highest standards was much larger than for the mortality estimates. For South-East Asia, the economic burden increased by about 50 per cent, for all other regions except Europe between 75 and 120 per cent, and for Europe it increased 8-fold (approach 1) or 13-fold (approach 2).

Combining mortality and absenteeism-related income losses, the global economic burden of not providing surgery to local standards amounted to $92 492 million using approach 1 and $73 141 million using approach 2. The additional economic burden of not providing surgery to the highest standard was $2512 million for approach 1 and $2525 million USD for approach 2. The economic burden of unmet access to surgical care at local standards comprised between 87 and 97 per cent of the total economic burden in all regions except Europe (*supplementary material Fig. S15*).

**Fig. 3:**
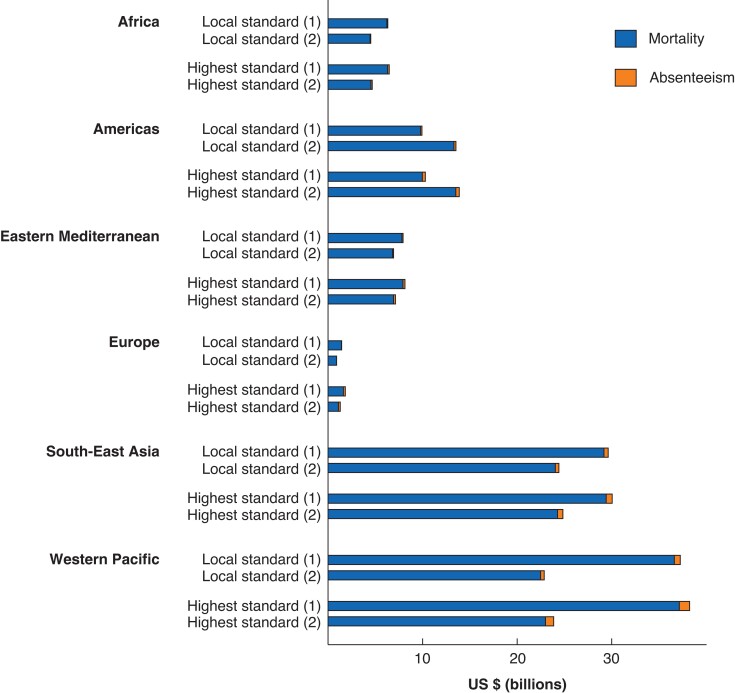
Composition of economic burden Economic burden is expressed in 2015 US dollar purchasing power parity. Approach 1 calculates unmet need as the relative difference of surgical volume to the minimum surgical volume proposed by the Lancet Commission on Global Surgery. Approach 2 calculates unmet need as the relative difference of estimated number of appendicectomies (calculated as World Bank income group-specific share of surgical volume) to the number of appendicitis cases.

Absenteeism-related income losses contributed to a small fraction of the economic burden of unmet surgical need for appendicitis, irrespective of the approach employed or benchmark (*Fig. 3*). The absolute economic burden was highest in South-East Asia and Western Pacific, but the difference between the two approaches was large for both regions. However, even the lower estimates for both regions yielded some 1.5–2-fold higher absolute costs than most other regions.

## Discussion

This study has identified a substantial absolute and relative economic burden associated with failure to provide adequate or quality surgical services for the treatment of acute appendicitis. This economic burden, although substantial for any low- or middle-income country, varied by more than 50-fold across different geographic regions.

Reduction in mortality and morbidity, and thus reduction in patient’s income losses, through provision of surgery requires that surgical care can be accessed and that care, once accessed, is of high quality. The Lancet Global Health Commission on High Quality Health Systems^19^ has suggested that lack of quality care is a greater contributor to lives lost than lack of access. In the present analysis, the economic burden was calculated both in terms of improving access to local quality of care and improving access to care at the best global standard. This was done with the aim of teasing apart the relative contributions of lack of access and lack of quality to the economic burden. It is notable that, for treatment of appendicitis, major improvements can be achieved by improving access to care delivered at local standards, and that the additional benefits from meeting the care standards of high-income countries, which were taken to be reflective of high quality, are relatively marginal. This suggests that most benefit can be gained by improving access to care at standards already attained within a geographic region. At first glance, this stands in contrast to the findings of a previous study^20^ that calculated the unmet surgical need of digestive diseases in low- and middle-income countries in terms of disability-adjusted life-years (DALYs). It was found that 45 per cent of the current surgically avertable burden in terms of DALYs could be avoided with scaled up access to higher-quality surgical care (defined by a lower case fatality rate). This may be explained in part by differences in methodology, but most likely relates to the impact of surgery on mortality from different digestive diseases. Simple emergency surgery for appendicitis has a substantial effect on mortality, maximizing the impact of reduced access.

Appendicectomy was selected because it is an emergency procedure required worldwide, and for which every surgeon receives training. Unlike specialist procedures, accessing any surgically ready facility should enable appropriate care. For more complex procedures, lack of appropriate surgical expertise is more likely to influence outcomes adversely. The present study found that improving access to appendicectomy at current local standards of care can substantially decrease mortality. This shows that, despite calls for improved quality, access should not be neglected, especially for the most common emergency procedures. Although they did not compare outcomes resulting from lack of access *versus* lack of quality, other studies^21,22^ have shown that lack of access to surgical care is a huge issue in low- and middle-income countries, particularly for emergency conditions^7^. Access is a multidimensional problem, encompassing service availability, and geospatial, financial, and sociocultural considerations. Solutions therefore require engagement of multiple stakeholders^23,24^. Although dimensions of geospatial, financial, and sociocultural considerations certainly need to be addressed, they offer complex challenges^25^. However, given the relative simplicity of surgery for appendicitis, it may be that service availability issues can be addressed more readily by task-shifting or sharing, to compensate for the worldwide shortage of surgeons^10^. Task shifting or sharing has been applied successfully to caesarean section and inguinal hernia in some contexts^26,27^. Increasing the availability of technicians could grow surgical services more rapidly than can possibly be achieved through traditional training. This solution could enable out-of-hours surgery in local hospitals and also release surgeons to provide increased diagnostic and perioperative care (which is particularly important in the emergency setting). It could also enable increased efficiency of theatre utilization. Improved access to emergency appendicectomy will thereby provide benefits in surgical care that reach beyond the treatment of appendicitis.

Although training surgical providers comes at a cost, if countries can raise revenue from earnings forgone as a result of morbidity or mortality associated with lack of access to quality care for appendicitis, and invest 5 per cent of this into improving services, as a recommended minimum share of public spending on health^28^, this could easily cover the costs of training. These findings can be put into perspective by taking India as an example. According to Global Burden of Disease data, India had an incidence rate of appendicitis of 121 per 100 000 in 2015 (world median 240 per 100 000), or about 765 000 cases in the age group 20–64 years, for which the economic burden was calculated. The present study estimated an unmet need for 653 000 people (85 per cent) in approach 1, and 418 000 people (55 per cent) in approach 2. Total costs for not providing surgery to a local standard amounted to $14 086 million in approach 1 and $9017 million in approach 2. In contrast, total care costs were 3616 rupees or $69.5 for an appendicectomy at a tertiary care hospital in India in 2010–2011^29^. If India could invest 5 per cent of the foregone earnings (for example, extracted through taxation) in surgical care, this would free up resources to provide 10.1 million (approach 1) or 6.5 million (approach 2) additional appendicectomies, thus closing the gap in surgical need. Such service development would inevitably provide wider benefits, particularly in emergency surgical care, as no service is provided in isolation. Measuring the societal impact of such an investment would be expected to demonstrate substantial additional improvements in care and associated societal benefits.

Although the results as a whole are striking, there are some nuances within the findings that are worthy of explanation. The economic burden, relative to GDP, of unmet surgical need was highest in South-East Asia, followed by Africa. Africa and South-East Asia had the greatest economic burden as both had a comparatively high share of unmet surgical need according to the estimates. For South-East Asia, a large contributor to the economic burden was the high incidence rate in Nepal, Bhutan, and Bangladesh according to the Global Burden of Disease data. Without these outliers, South-East Asia would have ranked second after Africa. The ranking differed between economic burden relative to GDP and absolute economic burden of unmet surgical need. The absolute economic burden was highest in Western Pacific and South-East Asia, followed by the Americas, Eastern Mediterranean, and Africa. For Western Pacific, relatively high wages led to higher income losses, whereas the high unmet need and the extreme incidence rates contributed further to the high economic burden for South-East Asia. For most regions, the income losses were higher using approach 1 compared with approach 2; the reverse was, however, true for the Americas, and was also evident at the country level. This seemed to be driven by a combination of comparatively high surgical volume with an even higher incidence rate. For the first approach, the gap between actual surgical volume and the need to achieve a surgical volume of 5000 per 100 000 was considered to estimate the unmet need. As the surgical volume was quite high in the Americas, this resulted in a reasonably low unmet need when using approach 1. Still, the surgical volume was not high enough to counterbalance the very high incidence rate, such that the estimate of unmet need in approach 2 exceeded the estimate of approach 1.

There are limitations to this study. The main constraint is the availability of data on appendicitis incidence, outcomes, and appendicectomies in low- and middle-income countries. The Global Burden of Disease project provides country-level estimates of the incidence of appendicitis, but these diverge from administrative data in high-income countries, probably owing to different data sources (see *appendix S.3*). Still, the Global Burden of Disease data are the only nationally comparable and comprehensive incidence data available. Additionally, there are large gaps in the surgical outcomes data, particularly in the emergency setting. Uniquely, this study benefited from accessing the raw data in the COVIDSurg Collaborative and GlobalSurg Collaborative studies which provided global prospectively collected outcomes data. The extrapolation to all low- and middle-income countries might not be accurate for the context of every country, and might obscure variations between certain countries. Similarly, there are no reliable global data to distinguish outcomes by age or sex. As the extrapolation is based on comparable data from several low- and middle-income countries, the results should yield reasonable estimates. Furthermore, because of uncertain and conflicting data on appendicectomies, two analyses were provided in an attempt to provide two different angles on unmet need. The assumption is that the approaches give a reasonable second-best option in the absence of data on appendicectomies performed. Finally, the models are static, in the sense that feedback mechanisms were not incorporated. For example, the models do not account for changes in surgical quality if the access to surgical care increases. Yet, the direction of such feedback mechanisms is likely to depend on many different factors, so any assumptions regarding such mechanisms would be highly debatable.

This study has shown that, for many low- and middle-income countries, investment in the provision of emergency surgery for appendicitis can be cost-effective by substantially reducing the economic burden of the illness. Development of local and district surgical services could have a positive knock-on effect, enabling access to care for other surgical emergencies and even elective procedures. This additional benefit will need to be evaluated in prospective studies, but might be substantial.

### Collaborators

National Institute for Health Research (NIHR) Global Surgery Collaboration: AA Essam, Abd Elkhalek Sallam, Abd Elrahman Elshafay, Abd El-Rahman Hegazy Khedr, Abdalla Gamal Saad, Abdalla Gharib, Abdalla Kenibar, Abdallah Salah Elsherbiny, Abdalrahman Adel, Abdelaziz Abdelaal, Abdelaziz Osman Abdelaziz Elhendawy, Abdelfatah Hussein, Abdelkader Belkouchi, Abdelmalek Hrora, Abdelrahman Adelshone, Abdelrahman Alkammash, Abdelrahman Assal, Abdelrahman Geuoshy, Abdelrahman Haroun, Abdelrahman Mohammed, Abdelrahman Sayed, Abdelrahman Soliman, Abdelrhman Essam Elnemr, Abdelrhman KZ Darwish, Abdelrhman Osama Elsebaaye, Abdul Khalique, Abdul Rehman Alvi, Abdul Wahid Anwar, Abdulaziz Altwijri, Abdullah Al-Mallah, Abdullah Almoflihi, Abdullah Altamimi, Abdullah Daqeeq, Abdullah Dwydar, Abdullah Gouda, Abdullah Hashim, Abdulmalik Altaf, Abdulmalik Huwait, Abdulrahman Abdel-Aty, Abdulrahman M. Altwigry, Abdulrahman Sheshe, Abdulrasheed A Nasir, AbdulRazzaq Oluwagbemiga Lawal, Abdulshafi Khaled Abdrabou, Abdurrahaman Sheshe, Abdussemiu Abdurrazzaaq, Abebe Bekele Zerihun, Abeer Al-shammari, Abeer El Gendy, Abeer Esam, Abeer Marey, Abhishek Mittal, Abiboye Yifieyeh, Abid Bin Mahamood, Abidemi Adesuyi, Abouelatta Khairy Aly, Abrar Nawawi, Adam Gyedu, Ade Waterman, Adedapo Osinowo, Adedeji Fatuga, Adel Albiety, Adel B Hassanein, Adel Denewar, Adeleke Adekoya, Ademola Adebanjo, Ademola Adeyeye, Ademola Popoola, Adesina Adedeji, Adesoji O Ademuyiwa, Adesoji Tade, Adewale Adeniyi, Adewale O Adisa, Adham Tarek, Adomas Ladukas, Adrian F. Palma, Afifatun Hasanah, Afizah Salleh, Afnan Abdelfatah, Afnan Altamimi, Afnan Altamini, Agazi Fitsum, Agboola Taiwo, Ahamed Hassan, Ahed Ghaben, Ahmad Abdel Fattah, Ahmad Abdel Razaq Al Rafati, Ahmad Aboelkassem Ibrahem, Ahmad Aldalaq, Ahmad Ali, Ahmad Almallah, Ahmad Alrifaie, Ahmad Ashour, Ahmad Bakr, Ahmad Bani-Sadar, Ahmad Bin Adnan, Ahmad Elbatahgy, Ahmad Faraz, Ahmad Gudal, Ahmad Hasan, Ahmad Khaled Sabe, Ahmad Khoja, Ahmad Nashaat, Ahmad Qaissieh, Ahmad Sabe, Ahmad Saber Sleem, Ahmad Sakr, Ahmad Shalabi, Ahmad Uzair Qureshi, Ahmed Aamer, Ahmed Abd El Galeel, Ahmed Abd Elmoen Elhusseiny, Ahmed Abd Elsameea, Ahmed Abdelkareem, Ahmed Abdelmotaleb Ghazy, Ahmed Abo El Magd, Ahmed Abo Elazayem, Ahmed Adamu, Ahmed Adel, Ahmed Afandy, Ahmed Ahmed, Ahmed Alghamdi, Ahmed Ali, Ahmed Al-khatib, Ahmed Altibi, Ahmed Alzahrani, Ahmed Ata, Ahmed Badr, Ahmed Dahy, Ahmed Diab, Ahmed El Kashash, Ahmed El Kholy, Ahmed Elgaili Khalid Musa, Ahmed Elgebaly, Ahmed Elkelany, Ahmed Elkholy, Ahmed El-Sehily, Ahmed Essam, Ahmed Fahiem, Ahmed Farag, Ahmed Fawzy, Ahmed Fouad, Ahmed Gad, Ahmed Ghanem, Ahmed Gheith, Ahmed Gomaa, Ahmed Hafez El-Badri Kotb, Ahmed Hammad, Ahmed Hassan, Ahmed Hossam Eldin Fouad Rida, Ahmed Ismail, Ahmed Karim, Ahmed Khyrallh, Ahmed Lasheen, Ahmed M. Rashed, Ahmed Magdy, Ahmed Mahmoud Abdelraouf, Ahmed Menshawy, Ahmed Meshref, Ahmed Mohamed Afifi, Ahmed mohamed Ibrahim, Ahmed Mohameden, Ahmed Mohammed, Ahmed Mokhtar, Ahmed Mosad, Ahmed Moustafa, Ahmed Moustafa Saeed, Ahmed Negida, Ahmed Rabeih Mohammed, Ahmed Rabie Mohamed, Ahmed Ragab Nayel, Ahmed Ragab Soliman, Ahmed Raslan, Ahmed Raza, Ahmed Refaat, Ahmed Rslan, Ahmed Sabry, Ahmed Sabry El-Hamouly, Ahmed Safwan Marey, Ahmed Saidbadr, Ahmed Sakr, Ahmed Samir, Ahmed Shahine, Ahmed Sheta, Ahmed Soliman, Ahmed Tammam, Ahmed Tarek Abdelbaset Hassan, Ahmed W. Shamsedine, Ahmed Zaki, Ahmed Zaki Eldeeb, Ahmed Zohair, Ahmedali M Kandil, Ahmedglal Elnagar, Ahsan Zil-E-Ali, Aijaz Jabbar, Ailsa Claire Snaith, Ainhoa Costas-Chavarri, Aiste Austraite, Ajayesh Mistry, Akin Olaolorun, Akinlabi E Ajao, Al Faifi Jubran, Ala Shamasneh, Alaa Abouelnasr, Alaa Al-Buhaisi, Alaa Bowabsak, Alaa El Jamassi, Alaa Elazab, Alaa Elhadad, Alaa Fergany, Alaa Habeebullah, Alaa Hassan, Alaa Shabkah, Alaa Shaheen, Alaba Adesina, Alan Baird, Alan Grant, Alasdair Ball, Alban Cacurri, Albert Mohale Mphatsoe, Alberto Realis Luc, Alejandro Matheu, Alejandro Munera, Alemayehu Ginbo Bedada, Alessandro Favero, Alessio Maniscalco, Alexander Canta Calua, Alexander J Fowler, Alexandra Gerosa, Alexandre Horobjowsky, Alexandre Venancio De Sousa, Alexia Farrugia, Alexis Pierre Arnaud, Alfio Alessandro Russo, Alfredo Gulielmi, Ali Ababneh, Ali Abo El Dahab, Ali Amin Ahmed Ata, Ali Khan Niazi, Ali Kiasat, Ali Mohamed Hammad, Ali Zardab, Ali Zeynel Abidin Balkan, Aliaa Gamal Toeema, Aliaa Sadek, Aliaksandr Filatau, Aliang Latif, Alibeth Andres Baquero Suarez, Alice Faure, Alice Niragire, Alina Robledo-Rabanal, Aline Broch, Alireza Hasheminia, Alisdair Macdonald, Aliyu Ndajiwo, Allan Novak, Alphonse Zeta Mutabazi, Alvaro Enrique Mendoza Beleño, Alvin Ee Zhiun Cheah, Aly Abd Elrazek, Aly Nasr, Aly Sanad, Alyaa Halim Elgendy, Alyne Daltri Lazzarini Cury, Amal Ibrahim, Amandine Martin, Amani Althwainy, Amany Abouzahra, Amany Eldosouky Mohammed, Amar Kourdouli, Amel Hashish, Amerdip Birring, Amgad Al Meligy, Amina Abdelhamid, Aminah Hanum Haji Abdul Majid, Aminu Mohammad, Amir Ait Kaci, Amira Atef Omar, Amira Elsawy, Amira Hassan Bekhet, Amira Reda, Amjad Abu Qumbos, Amjad Elmashala, Ammar Gado, Amna Mamdouh Mohamed, Amna Mohamed, Amoudtha Rasendran, Amr Ahmed Saleh, Amr Fadel, Amr Hasan, Amr Hassaan, Amr Hossameldin, Amr Muhammad Elkorashy, Amr Tarek Hafez, Amreen Faruq, Amro Aglan, Ana Cecilia Manchego Bautista, Ana Lucia Contreras-Vergara, Ana Maria Sandoval Barrantes, Ana Vega Carreiro De Freitas, Ana Vega Freitas, Anam Rashid, Anan Rady Abdelazeam, Anand Kirishnan, Anass Majbar, Anastasia Bamicha, Anastasios Stefanopoulos, Anders Thorell, Andre Das, Andre Dubois, Andre L Mihaljevic, Andre Navarro, Andrea Allegri, Andrea Armellini, Andrea Belli, Andrea Bondurri, Andrea Echevarria Rosas Moran, Andrea Natili, Andrea Ruzzenente, Andrea Simioni, Andreass Haloho, Andrei Tanase, Andrej Kolosov, Andrejus Subocius, Andrew G N Robertson, Andrew Kirby, Andrew Mcguigan, Andrew Spina, Andrey Litvin, Andrius Burmistrovas, Andrius Strazdas, Andy Arenas, Aneel Bhangu, Anele Rudzenskaite, Angel David Pérez Rojas, Angela Dell, Angelica Genoveva Vergara Mejia, Angeline Charles, Angelo Antoniozzi, Angelo Benevento, Angelos Tselos, Angham Solaiman El-Ma'doul, Anjana Sreedharan, Ankur Bhatnagar, Ann Kjellin, Anna Lasek, Anna Maffioli, Anna Powell, Anna Rinaldi, Anna Watts, Annalisa lo Conte, Annamaria Bigaran, Annelisse Ashton, Annisa Dewi Fitriana Mukin, Antanas Gulbinas, Antanas Zadoroznas, Anthonius Santoso Rulie, Anthony Ajiboye, Anthony Avoka, Anthony Chuk-Him Lai, Anthony Davor, Anthony Sander, Antje Oosterkamp, Antoinette Bediako-Bowan, Antonella La Brocca, Antônio Leal, Antonio Nocito, Antonio Ramos-De La Medina, Antonio Taddei, Anwar Atiyeh, Anyomih Theophilus Teddy Kojo, Aoife Driscoll, Apar Shah, Apostolos Vlachogiorgos, April Camilla Roslani, Aram Abdelhaq, Arazzelly del Pilar Paucar, Arcangelo Picciariello, Areej Tarek, Arezo Kanani, Ari Leppäniemi, Arianna Birindelli, Arij Ibrahim, Arjun Nesaratnam, Arlindawati Suyadi, Armando José Román Velásquez, Arnaud Bonnard, Arnav Agarwal, Aroub Alkaaki, Arturas Vaicius, Arvin Khamajeet, Arvo Reinsoo, Arwa Abouzaid, Arwa Elfarargy, Arwa Ibrahim, Arwa Mohamed, Asaf Kedar, Asdaq Ahmed, Ased Ali, Aseel Alnusairat, Aseel Hamarshi, Aseel Musleh, Ash Prabhudesai, Ashraf A. Maghrabi, Ashraf Morsi, Ashrarur Rahman Mitul, Asmaa Abdelgelil, Asmaa Abdel-Rahman Al-Aarag, Asmaa Rezq, Asmaa Salah, Aspasia Papailia, Assmaa Badwy, Astrid Leusink, Ata Khan, Ataa Ahmed, Athanasia Bamicha, Athar Eysa, Athirah Zulkifli, Atif Mahdi, Attia Attia, Attia Mohamed Attia, Audrey Clarissa, Audrius Dulskas, Audrius Parseliunas, Augusto Zani, Aung Kyaw Tun, Aurel Mironescu, Aurel Sandu Mironescu, Aurelien Scalabre, Aurora Mariani, Aurore Haffreingue, Aurore Thollot, Ausrine Usaityte, Austė Skardžiukaitė, Awais Raza, Aya Abdel Fatah Ibraheem, Aya Aboarab, Aya Adel Elsharkawy, Aya El-Sawy, Aya Elwaey, Aya Firwana, Aya Hagar, Aya Hammad, Aya Mohamed Fathy, Aya Reda, Aya Yehia Ata, Ayah Hamdan, Ayat Hassaan, Ayman And Taher, Ayman Elwan, Ayman Nabawi, Ayman Salman, Ayman Shwky, Ayokunle Ogunyemi, Azher Herebat, Azmina Verjee, Babajide Adenekan, Babatunde Odeyemi, Badr Eldin Adel, Badreldin Adel Tawfik, Bahar Busra Ozkan, Bakeer Mohamed, Bakhtiar Nighat, Bandar Albeladi, Bárbara Málaga, Barbara Mijuskovic, Barbara Pereira Silvestre, Basant Kumar, Basem Sieda, Bashir Bello, Basim Alghamdi, Basma Magdy, Basma Mahmoud, Basmah Alhassan, Bassant Mowafy, Beatrice Brunoni, Beatrix Weber, Belen Sanchez, Ben Thompson, Benoît Parmentier, Bernard Limoges, Bernard Van Duren, Bernhard Wolf, Bertrand Dousset, Besmir Grizhja, Bettina Lieske, Betty Maillot, Billal Mansour, Bjorn Frisk, Bogdan Diaconescu, Bogdan-Valeriu Martian, Boris Marinkovic, Brendan Skelly, Brian Cameron, Britta Dedekind, Bruno Noukpozounkou, Bryon Frankie Hon Khi Chong, Bylapudi Seshu Kumar, Caio Vinícius Barroso de Lima, Calogero Iacono, Cameron Fairfield, Camila Sanchez Samaniego, Camilla Cona, Camilo Lopez-Arevalo, Caoimhe Normile, Caranj Venugopal, Carla Cecilia Ramã­rez Cabrera, Carla Pierina García Torres, Carlo Corbellini, Carlos Alejandro Arroyo Basto, Carlos Iván Pérez Velásquez, Carlos Morales, Carlos Nsengiyumva, Carlos Paz Galvez, Carmen Capito, Carmen Fernández, Carmina Diaz-Zorrilla, Carolina Guzmán Dueñas, Carolina Oliveira Felipe, Caroline Clifford, Caroluce K Musyoka, Catherine A Shaw, Cathy Magee, Cecile Muller, Cecilia Costa, Cecilia Tolg, Cecilia Wredberg, Celeste Del Basso, Céline Grosos, Cesar Augusto Azmitia Mendizabal, Cesar Miranda, Cesar Razuri, Cezar Ciubotaru, Chali Chibuye, Challine Alexandre, Charing Cheuk Ling Szeto, Charles Dally, Charlotte Jane Mcintyre, Chaymae Benyaiche, Chean Leung Chong, Chee Siong Wong, Cheewei Gan, Chelise Currow, Chelsea Deane, Cheng Chun Goh, Cherry Koh, Cheryl Ou Yong, Chetan Khatri, Chi Chung Foo, Chi Ying Jacquelyn Fok, Chia Kong, Chiara Ceriani, Chimwemwe Kwatiwani, Chingwan Yip, Chintya Tedjaatmadja, Choon Seng Chong, Chouikh Taieb, Choy Ling Tan, Chris Bode, Chris Lee, Christel Leanne Almanon, Christian Hinojosa, Christian Lari Coompson, Christin Schoewe, Christina Neophytou, Christina P Major, Christina Panteli, Christine Mizzi, Christoforos Ferousis, Christopher Bode, Christos Agalianos, Christos Anthoulakis, Christos Barkolias, Christos Dervenis, Chu-Ann Chai, Chui Yee Wong, Ciara Fahy, Cicilia Viany Evajelista, Cirugia De Emergencia, Ciskje Zarb, Citra Dewi Mohd Ali, Claire Sharpin, Clara Milagros Herrera Puma, Clare M Rees, Clare Morgan, Claudia Reali, Claudio Arcudi, Claudio Fermani, Claudio Gabriel Fermani, Clemens Nawara, Clement Onuoha, Clodagh Mangan, Colleen Sampson, Collins Nwokoro, Colombani Jean-Francois, Constantinos Marinos, Cornelius Mukuzunga, Corrado Bottini, Craig Gouldthorpe, Crislee Elizabeth Lopez, Cristina Fernandes, Crystal Yern Nee Chow, Cutting Edge Manipal, Dale Vimalachandran, Dalia Alkhabbaz, Dalia Hemeda, Damien Brown, Damir Ljuhar, Dan L Deckelbaum, Dana Jaradat, Danelo Du Plessis, Daniel Ardian Soeselo, Daniel Cox, Daniel Dabessa, Daniel Estuardo Marroquín Rodríguez, Daniel Hamill, Daniel Nel, Daniel Youssef, Daniela Magri, Daniele Angelieri, Daniele Gui, Danilo Herrera Cruz, Danjuma Sale, Dansou Gaspard Gbessi, Dario Andreotti, Darius Kazanavicius, Darragh McCullagh, David Mcnish, David Merlini, David Monterroso Cohen, Davide De Boni, Davide Rossi, Dayang Nita Abdul Aziz, DC Grobler, Debora Schivo, Deborah Chiesa, Deimante Mikuckyte, Deividas Dragatas, Demi Gray, Diaa Eldin Abdelazeem Amin Elsorogy, Diaa Moustafa Elbendary Elsawahly, Diaaaldin Zahran, Diana Duarte Cadogan, Diana Sanchez, Dickson Bandoh, Diego Alonso Romani Pozo, Diego Antezana, Diego Coletta, Diego Romani, Diego Sasia, Dietmar Öfner, Dieudonne Duhoranenayo, Dimitri Aristotle Raptis, Dimitrios Balalis, Dimitrios K Manatakis, Dimitrios Karousos, Dimitrios Korkolis, Dimitrios Kyziridis, Dimitrios Lytras, Dimitrios Papageorgiou, Dimitrios Sfougaris, Dimitris-Christos Zachariades, Dina Al-Marakby, Dina Faizatur Rahmah, Dina Gamal, Dina Tarek, Dineshwary Periasammy, Diogo Vinicius dos Santos, Dion Morton, Diya Mirghani, Djifid Morel Seto, DM Cocker, Dmitri A Raptis, Dmitri Nepogodiev, Dmitri Raptis, Doaa Emadeldin, Doaa Gamil, Doaa Hasan, Doaa Hasanain, Doaa Maher Abdelrouf, Domenica Pata, Domingos Mapasse, Dominic Charles Marshall, Donal B O’Connor, Donatas Danys, Donatas Venskutonis, Dorota Radkowiak, Doug Bowley, Dovilè Majauskyté, Dulan Irusha Samaraweera, Durvesh Lacthman Jethwani, Dush Iyer, Dushyant Iyer, Dzianis Khokha, Dzmitry Paulouski, Ebenezer Takyi Atkins, Echaieb Anis, Edgar Domini, Edilberto Temoche, Eduardo Huaman, Edvard Grisin, Edvinas Dainius, Efeson Thomas, Egle Preckailaite, Ehab Alnawam, Ehab Mamdouh, Eirik Kjus Aahlin, Eirini Kefalidi, Ekow Mensah, Elaine Borg, Eldaa Prisca Refianti Sutanto, Eleanor Marks, Elena Goldin, Elena Muzio, Elena Vendramin, Elena Zdanyte Sruogiene, Eleonora Ciccioli, Elio Jovine, Elisa Francone, Elisabeth Jensen, Elissa Rifhan Mohd Basir, Elizabeth Snyder, Ella Teasdale, Elliot Akoto, Elodie Gaignard, Elodie Haraux, Elsa Robert, Elsayed Ali, Elsayed Gamaly, Emad Abdallah, Emad Al-Dakka, Emad Ali Ahmed, Emad Aljohani, Emad Mohamed Saeed Taha, Eman Abd Al Raouf, Eman Abdelmageed, Eman Abuqwaider, Eman Adel Sayma, Eman Elwy, Eman Emara, Eman Hashad, Eman Ibrahim, Eman Magdy, Eman Magdy Hegazy, Eman Mahmoud Abdulhakeem, Eman Mohamed Ibrahim, Eman Mohamed Morshedy, Eman Nofal, Eman Rashad, Eman Yahya Mansor, Emanuel Barrios, Emanuele Rausa, Emeka Nwabuoku, Emilia De Luca, Emilie Eyssartier, Emilio Dijan, Emma Blower, Emma Jurdell, Emma Upchurch, Emmanuel Acquah, Emmanuel Akatibo, Emmanuel Barrios, Emmy Runigamugabo, Enas Alaloul, Enas Alqahtani, Enoch Dagoe, Enoch Tackie, Eriberto Farinella, Eric Ackom, Eric Kofi Appiah, Erick Samuel Florez Farfan, Erik Hervieux, Erik Schadde, Erika Vicario, Erikas Laugzemys, Ernest Yemalin Stephane Ahounou, Eslam Elbanby, Eslam Ezzat, Esraa Abd Elkhalek, Esraa Abdalmageed Kasem, Esraa Alm Eldeen, Esraa El-Gizawy, Esraa Elhalawany, Esraa El-Taher, Esraa Gamal, Esraa Ghanem, Esraa Kasem, Esraa Samir Elbanby, Esraay Zakaria, Ethar Hany, Etienne Courboin, Eu Xian Lee, Euan Macdonald, Eugene Niyirera, Eugenio Grasset, Eugenio Morandi, Eugenio Panieri, Eva Borin, Evangelos Voulgaris, Evelina Slapelyte, Evelina Woin, Ewan Macdermid, Ewen M Harrison, Eyad Khalifah, Ezio Veronese, Fabian Deichsel, Fabrizio Aquilino, Fahd Abdel Sabour, Faisal Idris, Faith Qi Hui Leong, Fanjandrainy Rasoaherinomenjanahary, Farah Mahmoud Ali, Farhana Iftekhar, Farrag Sayed, Fatai Balogun, Fatema Al Bastawis, Fatema Asi, Fathee Nada, Fathi Elzowawi, Fathia Abd El-Salam, Fathy Sroor, Fatima Baluch, Fatimah I Elgendy, Fatma Elkady, Faustin Ntirenganya, Fawzia Abdellatif Elsherif, Fawzy Mohamed, Fayez Elian Al Barrawi, Fazlin Noor, Federica Bianco, Federica Falaschi, Federico Coccolini, Fei Zheng, Felipe Zapata, Felix Alakaloko, Felix Lee, Feng Yih Chai, Ferdy Iskandar, Fernanda Altoe, Fernanda Frade, Fernande Djivoh, Fernando Espinoza, Fernando Fernandez-Bueno, Fernando Tale, Ferry Fitriya Ayu Andika, Fidelis Jacklyn Adella, Filippo Di Franco, Finaritra Casimir Fleur Prudence Rahantasoa, Fitjerald Henry, Fitriana Nur Rahmawati, Florence Dedey, Florian Primavesi, Florin-Mihail Iordache, Fong Yee Lam, Foteini Koumpa, Francesca Steccanella, Francesco Pata, Francesco Riente, Francesco Ruben Giardino, Francesco Selvaggi, Francis Abantanga, Francis Dossou, Francisco Fujii, Francisco Regalado, Francois-Coridon Helene, Françoise Schmitt, Frank Enoch Gyamfi, Frank Owusu, Fred Alexander Naranjo Aristizã¡bal, Fred Hodonou, Frederick Du Toit, Frederique Sauvat, Fredrik Wogensen, Frehun Ayele Asele, Fridiz Saravia, Gabriel Pardo, Gabriela Elisa Nita, Gaetano Gallo, Gaetano Luglio, Gaetano Tessera, Galaleldin Abdelazim, Gamal Shimy, Gandau Naa Barnabas, Garba Samson, Gareth Irwin, Gehad El Ashal, Gehad Samir El Sayed, Gehad Tawfik, Gemma Humm, Genoveffa Balducci, George Christian Manrique Sila, George Ihediwa, George Manrique Sila, Georges Azzie, Georgette Marie Camilleri, Georgios Gemenetzis, Georgios Gkiokas, Georgios Karabelias, Georgios Kyrou, Georgios Tzikos, Gerardo Perrotta, Gerfried Teufelberger, Germain Ahlonsou, German Minguez, Geta Maharaj, Gezim Galiqi, Ghada Elhoseny, Ghada Saied Nagy, Ghiath Al Saied, Ghina Shamim Shamsi, Giacomo Nastri, Giacomo Pata, Gianfranco Cocorullo, Gianluca Curletti, Gianluca Pagano, Gianluca Pellino, Gianmaria Confalonieri, Gianpiero Gravante, Giedrius Lauzikas, Giles Dawnay, Gintaras Simutis, Giorgio Vasquez, Giovanni Landolfo, Giovanni Lazzari, Giovanni Luca Lamanna, Giovanni Pascale, Giovanni Pesenti, Giovanni Sgroi, Giridhar H Devadasar, Gisele Moreira, Giuliano Borda-Luque, Giuseppe Clerico, Giuseppe Rotunno, Giuseppe Salamone, Giuseppe Sammarco, Gokhan Lap, Greg Padmore, Gregorio Tugnoli, Gregory Kouraklis, Greta Mclachlan, Greta Wood, Greta Žiubrytė, Guillaume Podevin, Guillermo Sanchez Rosenberg, Guo Liang Yong, Gurdeep Singh Mannu, Gurpreet Singh Banipal, Gustavo Miguel Machain Vega, Gustavo Peixoto Soares Miguel, Gustavo Pereira Fraga, Gustavo Recinos, Gustavo Rodolfo Pertersen Servin, Haaris A. Shiwani, Hadeel Al-farram, Hafiz Hakim, Hagar Zidan, Hager Abdul Aziz Amin, Hager Abdulaziz, Hager Ahmed El-badawy, Hager Elwakil, Hager Tolba, Hagir Zain Elabdin, Haidar Hajeh, Hala Ahmed, Hala Saad, Halima Aliyu, Hamdi Ebdewi, Hamza Asumah, Hamza Waleed, Hanan Adel Saad, Haney Youssef, Hani Natalie, Hanna Royson, Hannah Anderson-Knight, Hannah Burns, Hannah S Thomas, Hans-Ivar Pahlsson, Harish Neelamraju Lakshmi, Harriet Jordan, Hasan Ismael Ibraheem, Hasan Ismael Ibraheem Al-Hameedi, Hasbi Maulana Arsyad, Hasnain Abbas Dharamshi, Hassan Ali Mostafa, Hatem El-Sheemy, Haya Tahboub, Hayam Ahmed, Hayden Kretzmann, Hayssam Rashwan, Haytham Abudeeb, Hazem Khaled, Hazmi Dwinanda Nurqistan, Heather Bougard, Heba Baraka, Heba Gamal, Heba Shaker, Hector Shibao Miyasato, Helen Mohan, Helen Woodward, Helena Franco, Helene Francois-Coridon, Helmut Alfredo Segovia Lohse, Hend Adel Gawad Shakshouk, Hend Kandil, Hend Mahmoud, Henri Kotobi, Henry Mendel, Henry Nnaj, Herlin Karismaningtyas, Herman Cruz, Hesham Magdy, Hesham Mohammed Bakry, Hian Ee Heng, Hildur Thorarinsdottir, Hisham Safa, Hisham Samih, Hogea Mircea, Hong Kong SAR, Hong Yee Wong, Hoong-Yin Chong, Hope Edem Kofi Kordorwu, Hope Glover-Addy, Horacio Paredes Decoud, Hosni Khairy Salem, Hossam Dawoud, Hossam Elfeki, Hossam Emadeldin, Houda Bachri, Hunain Shiwani, Hussein Ali, Hussein El-Kashef, Hussein Mohammed, Hussien Ahmed, Iason-Antonios Papaskarlatos, Ibrahem Abdelmotaleb, Ibrahim AbdelFattah, Ibrahim Alhabli, Ibrahim Al-Slaibi, Ibrahim AlYoussef, Ibrahim Elzayat, Ibrahim Elzayyat, Ibrahim N. Alomar, Ibrahim Rakha, Ibrahim Raza, Ida Björklund, Idelso Vasquez, Ignas Rakita, Ihab Hassan, Ihdaa Adawi, Iloba Njokanma, Iman Elkadsh, Immacolata Iannone, Ingemar Havemann, Ioannis Kyriazanos, Ioannis Patoulias, Ioannis Valioulis, Ionasc Dan, Ionut Negoi, Ionut-Bogdan Diaconescu, Irene Montes, Irene Ortega-Vazquez, Isaac Amole, Isaac Bertuello, Isaac Hanley, Isam Bsisu, Islam Magdy El Sayed, Ismael Isaac Zelada Alvarez, Ismail Lawani, Israa Abdullah Aziz Al-Azraqi, Israa Adel, Israa Awad, Israa Qawasmi, Ivan Mendoza Restrepo, J Edward Fitzgerald, Jack Almy, Jacqueline Sheehan, Jaime Andres Montoya Botero, Jaime Herrera-Matta, Jakeline Restrepo, Jakov Mihanovic, James Adeniran, James Brown, James Davies, James Giles, James Glasbey, James Olivier, James Pape, James Richards, James Wheeler, James Yang, Jamie Shah, Janet Pagnozzi, Jannin Salcedo, Jasim Amin, Jason Brown, Javier Pastora, Javier Rosales, Jazmin Coronel, Jean Bréaud, Jean De La Croix Allen Ingabire, Jean-Baptiste Marret, Jean-Francois Colombani, Jean-François Lecompte, Jeffrey Dalli, Jehad Hassan Youssif, Jehad Meqbil, Jemina Onimowo, Jen Cornick, Jenifa Jeyakumar, Jennifer Nowers, Jennifer Rickard, Jennifer Skehan, Jerry Makama, Jesse Ron Swire Ting, Jessica Juliana Tan, Jessica Patricia Gonzales Stuva, Jessica Roth, Jessica Souza Luiz, Jia Hao Law, Jia Yng Siaw, Jian Er Saw, Jibran Abbasy, Jiheon Song, Jimy Harold Jara Quezada, Joachim Amoako, Joachim Wiborg, Joanna Swann, Jo-Anne Carreira, Joanne Edwards, Joe Vincent, Joel Kin Tan, Joe-Nat Clegg-Lamptey, Johanna Joosten, Johanna Nyberg, Johannes Kurt Schultz, Johannes Wiik Larsen, John Bondin, John F. Camilleri-Brennan, John Jemuel V. Mora, John Lee Y Allen, John Whitaker, Jolanta Gribauskaite, Jon Arne Søreide, Jon Kristian Narvestad, Jonathan Ajah, Jonathan Dakubo, Jonathan Heath, Jonathan R L Wild, Jonny Setiawan, Jorge Armando Chungui Bravo, Jorge Torres Cardozo, Jose Aguilar-Jimenez, Jose Andres Garcia-Marin, Jose Antonio Cabala Chiong, Jose Costa-Maia, José Hamasaki, José Luis Hamasaki Hamaguchi, Jose Luis Rodicio, Jose María Vergara Celis, José René Arévalo Azmitia, Joselyn Ye, Joseph Awuku-Asabre, Josephine Psaila, Joshua Luck, Joshua Michael Clements, Joyeta Razzaque, Juan Camilo Correa, Juan Carpio, Juan Gouws, Juan Jaime Herrera Matta, Juan Manuel Carmona, Juan Marcelo Delgado, Juana Kabba, Jubran J Al-Faifi, Julia Guasti Pinto Vianna, Julian Camilleri-Brennan, Juliana Menegussi, Julien Leroux, Julien Rod, Juliette Hascoet, Julio Jimenez, Junyeong Oh, Juozas Kutkevicius, Justas Kuliavas, Justas Žilinskas, Justin Chak Yiu Lam, Justus Lando, Ka Hin Gabriel Li, Ka Wai Leung, Kai Yin Lee, Kalangu Kabongo, Kalitha Pinnagoda, Kalon Hewage, Kamau Kinandu, Kamran Faisal Bhopal, Kandasami Palayan, Kareem Dabbour, Kareem Elshaer, Karen Bailey, Karim Hilal, Karl Bonavia, Karolis Lagunavicius, Karolis Varkalys, Kate Cross, Kate Yu-Ching Chang, Katharina Beate Reinisch, Katharine Whitehurst, Katherine Gash, Kathryn Chu, Kathryn Lee, Katie Connor, Katrin Gudlaugsdottir, Kaustuv Das, Kazeem Atobatele, KC Janardha, Kean Leong Koay, Keat-Seong Poh, Keiran David Clement, Keith Sammut, Keith Say Kwang Tan, Kenneth Aaniana, Kenneth Johnson, Kenneth Mealy, Kenneth Thorsen, Kenny Turpo Espinoza, Kent Pluke, Kestutis Strupas, Kevin C. Conlon, Kevin Turpo Espinoza, Khaled Abozeid, Khaled Alhady, Khaled Aljboor, Khaled Dawood, Khaled Hesham Elbisomy, Khaled Ibrahim, Khaled Khattab, Khaled Naser El Deen, Khalid Mahmud, Khalid Qurie, Khalid Salah El-Dien, Khalil Abdul Bassit, Khaoula Boukhal, Khlood Ashour, Kholod Tarek Lasheen, Kholoud Abdelbadeai, Khurram Khan, Khuzaimah Zahid Syibrah, Kieran Atkinson, Kieran Ka Kei Li, Kirsten Lafferty, Kjetil Søreide, Knut Magne Augestad, Kolonia Konstantina, Konstantinos Farmakis, Konstantinos Gasteratos, Kornelija Maceviciute, Kpèmahouton René Keke, Kresimir Zamarin, Kristian Styles, Kristijonas Jasaitis, Kristijonas Jokubonis, Kristina Cassar, Kuet Jun Chung, Kuhaendran Gunaseelan, Kuok Chung Lee, Kurt Carabott, Kwabena Agbedinu, Kwaku Boakye-Yiadom, Kwame Maison, Kwasi Asare-Bediako, Kwasi Kusi, Kyaw Phyo Aung, Kylie Joan-yi Szeto, Kyriakos Psarianos, Laimonas Uščinas, Lalith Asanka Jayasooriya Jayasooriya Arachchige, Lana Abusalem, Larissa Ines Páez Lopez, Lau Wen Liang Joel, Laura Gavagna, Laura Koskenvuo, Laura Lorenzon, Laura Luque, Laurent Fourcade, Lawal Abdullahi, Lawani Ismaïl, Lawrence Bongani Khulu, Layza-Alejandra Mercado Rodriguez, Lee Shi Yeo, Leif Israelsson, Lemuel Davies Bray, Lenin Peña, Leo Licari, Leonardo Solaini, Li Jing Yeang, Liam Henderson, Liam Richardson, Liana Roodt, Lillian Reza, Linas Urbanavicius, Linas Venclauskas, Linda Alvi Madrid Barrientos, Linda Andersson, Ling Wilson, Linn Nymo, Linnea Mauro, Liviu Iuliu Muntean, Liviu Muntean, Ljiljana Jeremic, Lofty-John Anyanwu, Lopna Ahmed Mohamed Ahmed, Lorena Fuentes-Rivera, Lorena Rodriguez, Lorena Solar García, Lorraine Sproule, Lotfy Eldamaty, Luai Jamal, Luana Ayres Da Silva, Lubna Sabeeh, Luc Hervé Samison, Luca Ansaloni, Luca Bortolasi, Luca Turati, Lucia Duinhouwer, Lucian Corneliu Vida, Lucile Fievet, Lucio Selvaggi, Ludwing Alexander Zeta Solis, Luen Shaun Chew, Luigi Bonavina, Luigi Bucci, Luigi Maria Cloro, Luis Alberto Valente Laufer, Luis Barneo, Luis Joaquín García Florez, Luis M. Helguero-Santin, Luis Miguel Alvarez Barreda, Luis Tale, Luisa Giavarini, Luiz Carlos Barros De Castro Segundo, Luiza Sarmento Tatagiba, Lukas Eisner, Lusi Padma Sulistianingsih Mata, Maarten Vermaas, Mabel Amoako-Boateng, Maciej Walędziak, Madan Jha, Madelaine Gimzewska, Mads Gran, Maeve O'neill, Magdalini Mitroudi, Magnus Boijsen, Maha Al-faqawi, Maha Elmasry, Maha Gamal Mohamad Hamad, Maha Nasr, Mahadevan Deva Tata, Mahitab Essam, Mahitab Morsy Farahat, Mahmoud A. Elnajjar, Mahmoud Abdelshafy, Mahmoud Abdelshakour, Mahmoud Abdulgawad, Mahmoud Ahmed Fathi Abozyed, Mahmoud Alrahawy, Mahmoud Amreia, Mahmoud Badawy, Mahmoud Eldafrawy, Mahmoud Elfiky, Mahmoud Elkhadragy Maher, Mahmoud Elkhadrawi, Mahmoud Elsayed Moghazy, Mahmoud Gomah, Mahmoud M. Saad, Mahmoud Mohamed Metwally, Mahmoud Morsi, Mahmoud Saad, Mahmoud Saami, Mahmoud Salama, Mahmoud Salma, Mahmoud Shalaby, Mahmoud Warda, Mahmoud Zakaria, Mahmut Arif Yuksek, Mahnoor Javaid, Mahnuma Mahfuz Estee, Mai Ebidy, Mai Mohamed Ebidy, Mai Salama, Maíra Cassa Careta, Maja Marcus, Majd Dabboor, Majed Aboelella, Makafui Dayie, Makki Elsayed, Malcolm Falzon, Maleeha Hassan, Malin Sund, Man Fung Leung, Man Hon Andrew Yeung, Manar Abd-Elmawla, Manar Saeed, Mantas Drungilas, Mantas Jokubauskas, Mantas Vilčinskas, Manuel Francisco Roxas, Manuel Hache-Marliere, Manuel Lopez, Manuel Rodriguez Castro, Manuela Mendez, Manzoor Dar, Maram Abu-toyour, Maram Salah, Marcelo O´Higgins Roche, Marco Catani, Marco Maria Pascale, Marco Migliore, Mardelangel Zapata Ponze De Leon, Margaret O'Shea, Margarita Montrimaite, Margherita Notarnicola, Margub Hussain, Maria Clara Mendoza Arango, Maria Giovanna Grella, Maria Hjertberg, Maria Isabel Villegas Lanau, Maria Jesusa B. Maño, Maria Lorena Aguilera, Maria Marta Modolo, Maria Mayasari, Maria Novella Ringressi, Maria Soledad Gonzales Montejo, Maria Soledad Merlo, Maria Utter, María Valcarcel-Saldaña, Maria-Lorena Aguilera-Arevalo, Mariam Darweesh, Mariam O. Gad, Mariam Saad Aboul-Naga, Mariano Cesare Giglio, Mariastella Malavenda, Marie Carmela Lapitan, Marie Dione Parreno-Sacdalan, Marie Paul, Mariette Renaux-Petel, Marija Agius, Marilia Del Carmen Escalante Salas, Marilla Dickfos, Marina Luiza Pimenta, Mario Contreras Urquizu, Mario Corbellino, Mário Jacobe, Mario Lopez, Mario Pasini, Mario Trompetto, Marisa Leal, Marisol Manriquez-Reyes, Mariuca Popa, Mark Ian Hampton, Mark Sykes, Mark Wagener, Markus Zuber, Marte Bliksøen, Martha Glynn, Martin Jarmin, Martin Kyereh, Martina Perino, Martina Yusuf Shawky, Martinique Vella-Baldacchino, Marvin Vargas, Marwa Altarayra, Marwa Elashmawy, Marwa Elshobary, Marwa Hamdan, Marwa Sayed, Marwan Abubakr, Marwan Fahim, Marwan Shawki, Maryam Ali Khan, Maryna Shubianok, Mashael Al-Mousa, Masood Alghamdi, Masood Jawaid, Massiell Machaca, Massimiliano Dal Canto, Massimo Coletti, Matas Pažuskis, Matei Bratu, Matei Razvan Bratu, Mateusz Rubinkiewicz, Matteo Papandrea, Matteo Ripa, Mattew Ekow, Matthew Baldacchino, Matthew Billy, Matthew Young-Han Kim, Matthieu Peycelon, Matti Tolonen, Maureen Bezzina, Maurizio Foco, Mawaddah Alrajraji, Max Dénakpo, Max Rath, Mayaba Maimbo, Mazed Mohamed, Mazen Hassanain, Megan Turner, Mehmet Ali Yavuz, Mehmet Gumar, Mehmet Uluşahin, Melanie Castro Mollo, Melanie Zapata Ponze De Leon, Menatalla Salem, Mengistu Worku, Menna Tallah Ramadan, Mennaallah Hafez, Mennat-Allah Mustafa, Menold Archee P. Redota, Meran Allam, Meric Mericliler, Merna Mostafa, Meryem Abbouch, Metwally Aboraya, Michael Amoah, Michael Cox, Michael Edye, Michael Gillespie, Michael Hanrahan, Michael Livingston, Michael Puttick, Michael Stoddart, Michael Van Niekerk, Michael Walsh, Michael Wilson, Michail Kontos, Michail Margaritis, Michał Janik, Micheal Ohene-Yeboah, Michela Monteleone, Michele Carlucci, Michele Sacco, Michelle Mccarthy, Midhun Mohan, Miguel Angel Paludi, Miguel Siguantay, Mihael Radic, Mihaela Vartic, Miklosh Bala, Milaksh Kumar Nirumal, Milan Radojkovic, Milica Nestorovic, Millika Ghetia, Mindaugas Kiudelis, Mircea Beuran, Mircea Hogea, Mirko Mangiapane, Mitchelle Solange De Fã Tima Linares Delgado, Moayad Othman, Mobolaji Oludara, Modise Zacharia Koto, Mohamad Baheeg, Mohamad Bakhaidar, Mohamad Jeffrey Bin Ismail, Mohamed A Abdelaziz, Mohamed A Amer, Mohamed A Baky Fahmy, Mohamed Abbas, Mohamed Abd El Slam, Mohamed Abdelaty, Mohamed Abdelaty Mohamed, Mohamed Abdelkhalek, Mohamed Abdelraheim, Mohamed Abozaid, Mohamed Abozed Abdullah, Mohamed Abuseif, Mohamed Adel Badenjki, Mohamed Ali Ghonaim, Mohamed Ali Mahmoud, Mohamed Ameen, Mohamed Ammar, Mohamed Asal, Mohamed Awad Elkarim Hamad Mohamed, Mohamed Dablouk, Mohamed El Halawany, Mohamed Elazoul, Mohamed Elbermawy, Mohamed Elfil, Mohamed Elsehimy, Mohamed Elzayat, Mohamed Etman, Mohamed F Zalabia, Mohamed Fares, Mohamed Fawzy Mahrous Badr, Mohamed Fouad Hamed, Mohamed Gadelkarim, Mohamed Ghoneem, Mohamed Gulamhussein, Mohamed Hafez, Mohamed Hashish, Mohamed Hassab Alnaby, Mohamed Husseini, Mohamed Ibrahim, Mohamed Ismail, Mohamed Karkeet, Mohamed Kelany, Mohamed Mabrouk, Mohamed Magdy, Mohamed Mahmoud, Mohamed Moamen Mohamed, Mohamed Moaty, Mohamed Mostafa, Mohamed Mustafa, Mohamed Nashat, Mohamed Nazir, Mohamed Reda loaloa, Mohamed Rezal Abdul Aziz, Mohamed Sabry Ammar, Mohamed Salah, Mohamed Salah Elhelbawy, Mohamed Seisa, Mohamed Shaalan, Mohamed Sleem, Mohamed Sobhi Jabal, Mohamed Youssef, Mohamed Zidan, Mohamedraed Elshami, Mohammad Abdulkhalek Habeeb, Mohammad Aboraya, Mohammad Adawi, Mohammad Alherz, Mohammad Aliyu, Mohammad Elsayed Omar, Mohammad Ghannam, Mohammad Ghassan Alwafai, Mohammad Mohsin Arshad, Mohammad Rashid, Mohammadasim Amjad, Mohammed Alamoudi, Mohammed Alhendy, Mohammed AlRowais, Mohammed Alsaggaf, Mohammed Alzahrani, Mohammed Bukari, Mohammed Deputy, Mohammed Elgheriany, Mohammed Elsayed, Mohammed Elshaar, Mohammed Elsiddig, Mohammed Firdouse, Mohammed G. Azizeldine, Mohammed Hanafy, Mohammed Ismail, Mohammed Kamal Ismail, Mohammed Mousa, Mohammed Mousa Salem, Mohammed Mustafa Hassan Mohammed, Mohammed Mustafa Mohammed, Mohammed Najjar, Mohammed Nasr, Mohammed Osman, Mohammed Osman Dablouk, Mohammed Saeed, Mohammed Saleh A. Alghamdi, Mohammed Ubaid Alsaggaf, Mohammed Yahia Mohamed Aly, Mohannad Aledrisy, Mojolaoluwa Olugbemi, Mona Hamdy Madkor, Mona Hosh, Mona Rashad, Monica Bassem, Monique Moron Munhoz, Monty Khajanchi, Morgan Haines, Morvarid Ashtari, Mostada Samy, Mostafa Abdelkader, Mostafa Ahmed Bahaa Eldin, Mostafa Allam, Mostafa Gemeah, Mostafa Mahmoud Eid, Mostafa Qenawy, Mostafa Samy, Mostafa Seif, Mostafa Shalaby, Mousa Mustafa, Moustafa Ibrahim Mahmoud, Moustafa R. Aboelsoud, Msafiri Kimaro, Muayad Ahmed Alfarsi, Muhamed M H Farhan-Alanie, Muhammad Adil, Muhammad Alkelani, Muhammad Amsyar Auni Lokman, Muhammad Bin Hasnan, Muhammad Daniyan, Muhammad El-Saied Ahmad Muhammad Gohar, Muhammad Fathi Waleed Omar, Muhammad Habib Ibrahim, Muhammad Mohsin Furqan, Muhammad Rashid Minhas Qadir, Muhammad Saqlain, Muhammad Shawqi, Muhammad Talha Butt, Muhammad Taqiyuddin Yahaya, Muhammad Waqar, Muhammed Masood Riaz, Muhammed Talaat, Muhtarima Haque, Muna Rommaneh, Murad Aljiffry, Murat Karakahya, Musah Yakubu, Muslimat Alada, Mustafa Farhad, Mustafa Mohammed Taher, Muthukumaran Rangarajan, Muwaffaq Mezeil Telfah, Myint Tun, Myranda Attard, Nada Ahmed Reda Elsayed, Nada El-Sagheer, Nada Elzahed, Nada Mohamed Bekhet, Nader Abd El Hamid, Nadia Khalid Abd El-Latif, Nadia Ortiz, Nadin Elsayed, Nadya Johanna, Nahilia Carrasco, Najwa Nadeem, Naomi J Wright, Napoleon Mendez, Narimantas E. Samalavicius, Nashat Ghandora, Nasir Bustangi, Natale Di Martino, Natalie Blencowe, Natalie Redgrave, Nathalie Botto, Nathania Sutandi, Nawal Sadig, Nazmie Kariem, Nebil Behar, Nebiyou Seyoum Abebe, Nebyou Seyoum, Nebyou Seyoum Abebe, Neel Gobin, Neel Limaye, Neerav Aruldas, Nehal Yosri Elsayed Abdel-Wahab, Neil Smart, Nelson Manuel Urbina Rojas, Nelson Msiska, Nerijus Kaselis, Nermeen Soubhy El-Shahat, Nermin M Badwi, Nermin Mohamed Badwi, Nesma Elfouly, Nicholas Phillips, Nichole Starr, Nicola Chetta, Nicola Zanini, Nicolas Henric, Nicole D'aguzan, Nicole Grech, Nicoleta Panait, Nicoletta Leone, Nicolò Falco, Nidhi Gyanchandani, Nigel J Hall, Nihaal Shaikh, Niiarmah Adu-Aryee, Nik Azim Nik Abdullah, Nik Ritza Kosai, Nikica Pezelj, Nikki Green, Nikolaos Gouvas, Nikolaos Ivros, Nikolaos Mitroudis, Nikolaos Nikoloudis, Nikolaos Zampitis, Nithya Niranjan, Niveshni Maistry, Noha Abdullah, Noha Abdullah Soliman, Noha Maraie, Noha Wael, Nohad Osman, Noman Shahzad, Nora Abdul Aziz, Norah Al Subaie, Noran Abdel-Hameed, Noran Halim El Gendy, Norbert Uzabumwana, Norberto Herrera, Norma Depalma, Nosisa Sishuba, Nouf Akeel, Noura A. Attallah, Nourhan Adam, Nourhan Anwar, Nourhan Elsabbagh, Nourhan Medhat Elhadary, Nourhan Mesbah, Nourhan Semeda, Nourhan Soliman, Novia Adhitama, Nowrin F. Aman, Nuno Muralha, Nur Zulaika Riswan, Nurlaila Ayu Purwaningsih, Nyawira Ngayu, Octavio Garaycochea, Oday Halhouli, Ogechukwu Taiwo, Ola Sherief Abd El Hameed, Olabisi Osagie, Olabode Oshodi, Olajide Abiola, Olalekan Ajai, Oliver Warren, Oliver Ziff, Olivier Abbo, Olivier Azzis, Olivier Rosello, Olubukola Faturoti, Olufemi Habeeb, Olumide Elebute, Oluseyi Ogunsua, Oluwaseyi Adebola, Oluwatomi Odutola, Omar Abdelkader, Omar Abdulbagi, Omar Aguilera, Omar Alahmady, Omar Arafa, Omar Ghoneim, Omar Hesham, Omar Mattar, Omar Moussa, Omar Osman, Omar Salah, Omar Saleh, Omnia Aboelmagd, Omnia Mosalum, Omobolaji O Ayandipo, Omolara Faboya, Omolara Williams, Opeoluwa Adesanya, Orestis Ioannidis, Osaid H. Alser, Osama Algohary, Osama Mohamed, Osama Mohamed Salah, Osama Mokhtar Mohamed Hassan, Osama Saadeldeen Ebrahim, Osama Seifelnasr, Osman Imoro, Ossama Al-Obaedi, Otto Coyoy-Gaitan, Ourdia Bouali, Owusu Emmanuel Abem, Oyediran Kehinde Timothy, Oyindamola Oshati, Pablo Ramazzini, Pål Aksel Næss, Pamphile A Assouto, Panchali Sarmah, Pandi Eduard, Panu Mentula, Paola Salusso, Paola Violi, Paolino De Marco, Paolo Aonzo, Paolo Silvani, Paolo Ubiali, Patrizio Mao, Paul Kielty, Paul Sutton, Paul Ugalde, Paul Witherspoon, Paul Wondoh, Pauline Gastaldi, Paulius Karumnas, Paulius Kondrotas, Paulo Alves Bezerra Morais, Pedro Angel Toribio Orbegozo, Peep Talving, Pei Ying Koh, Per Weber, Per-Olof Lundgren, Peter Deutschmann, Peter Labib, Peter Wiel Monrad-Hansen, Petras Višinskas, Phebe Anggita Gultom, Philip Alexander, Philip Choi, Philip Mshelbwala, Philip Taah Amoako, Philippe Buisson, Phoebe De Bono, Phumudzo Ndwambi, Pier Paolo Grandinetti, Piergiorgio Danelli, Pierpaolo Sileri, Pietra Ligure, Pietro Mingrone, Pigeneswaren Yoganathan, Piotr Major, Poddevin Francois, Povilas Ignatavicius, Povilas Mazrimas, Prasad Pitigala Arachchi, Pratik Jain, Prince Kwakyeafriyie, Prisca A.L. Har, Pui Xin Chin, Puneet Malik, Puyearashid Nashidengo, Qinyang Liu, Quentin Alimi, Quentin Ballouhey, Quinn Ellison, R. Goh Ern Tze, Rachel King, Rachel Moore, Radhian Amandito, Radin Mohd Nurrahman Radin Dorani, Rafael Araujo, Rafael Soley, Rafał Roszkowski, Raffaele Galleano, Ragavan Narayanan, Ragnar Herikstad, Rahma Kamil, Rajeev Satoskar, Rakan Kabariti, Ralph F Staerkle, Ram Nataraja, Ramadan Oumer, Ramadan Shaker, Ramdan Shaker, Ramesh Jonnalagadda, Ramon Alvarado Jaramillo, Ramón Augusto Melo Cardozo, Rana Mamdouh, Rana Saadeh, Raquel Rodríguez-Uría, Raquillet Claire, Rasha Abdelhamed, Razvan-Matei Bratu, Reda Žilinskienė, Redouane Mammar Bennai, Reem Alyahya, Reem Fakher, Reem Husseiny, Reem Khreishi, Reem Mohammed Hassan Balila, Rehab Elashry, Reham Alaa El-Din, Reham Alshareef, Reham Saad, Renato Melo, Reuban D'cruz, Reuben Goh Ern Tze, Reynu Rajan, Rezaul Karim, Ricardo Velasquez, Richard Gilbert, Richard Lilford, Richard Opoku-Agyeman, Richard Spence, Richard William Gilbert, Richmond Hagan, Rifan Alyami, Riinu Ots, Ritauras Rakauskas, Roaa Khan, Robert George, Robert Karlo, Robert Kerley, Robert Mcintyre, Robert Morton, Robert Parker, Robert Tyler, Roberta Bugeja, Roberta Tutino, Roberta Villa, Robertas Baltrunas, Robertas Pranevicius, Roberto Cautiero, Roberto Cirocchi, Roberto Faccincani, Roberto Klappenbach, Roberto Macchiavello, Roberto Peltrini, Roberto Schiavone, Robinson Mas, Roel Matos-Puig, Rofida Elsemelawy, Roger Lawther, Roger Schmid, Rohan Ardley, Rohi Shah, Rokas Rackauskas, Rokayah Julaihi, Rokia Sakr, Roland Osuoji, Romeo Guevara, Romeo Lages Simoes, Romualdas Riauka, Ronald Coasaca Huaraya, Ronald Renato Barrionuevo Ojeda, Ronan Cahill, Rony Camacho, Rory Callan, Rosario Sacco, Rose Khreishi, Rosie Mcdonald, Ross Bowe, Ross Coomber, Rowida Elmelegy, Roxanne Chenn, Roy Quek, Rubén Balmaceda, Rubén Darío Arias Pacheco, Ruben Rivas, Ruben Santiago Restrepo Giraldo, Rudy Gunawan, Rula Zaa'treh, Ruqaya Kadhim Mohammed Jawad Al-Hasani, Ruta Mazelyte, Ruth Blanco, Ruth Gratton, Ruth Scicluna, Ryan Adams, Ryan Choon Kiat Tan, Ryan Mcintosh, S.V. Kinnera, Saad Al Awwad, Sabbir Karim, Sabine Irtan, Sabrina Asturias, Sabrina Dardenne, Sabry Mohy Eldeen Mahmoud, Safia Ali, Safwat Al-Nahrawi, Saged Elsherbiney, Sahar Abdoun Ishag Idris, Sahar Jaber, Sahlu Wondimu, Saiba Abdul-Latif, Said Alyacoubi, Sakhaa Hanoun, Saleem El-Rabaa, Saleh A. Alnuqaydan, Saleh Alqahtani, Salim Anderson Khouri Ferreira, Sally Elshanwany, Sally Hallam, Salma Magdy, Salma Mansour, Salma Said Elkolaly, Salman Aldhafeeri, Salomone Di Saverio, Salwa Khallaf, Sam Arman, Sam Debrah, Sam Seisay, Samaa Mahmoud Al Attar, Samah Afana, Samantha Corro-Diaz Gonzalez, Samar Abdelhady, Samar Adel Ismail, Samar Saad, Samar Soliman, Sameer Kushwaha, Sameh Emile, Sameh Sarsik, Sami Martin Sundstrom, Samson Olori, Samuel Essoun, Samuel Nigo, Samuel Osei-Nketiah, Samuel S. Y. Sii, Samuel Sani Ali, Sandip Kumar, Sandra Ahlqvist, Sandrine Kwizera, Sandro Pasquali, Sani Ali Samuel, Sanju Sobnach, Santiago Villalobos, Sara Abd Elmageed Barakat, Sara Ahmed, Sara Al-saqqa, Sara Amr Mohamed Farouk, Sara Arafa, Sara Ayad, Sara Elhamouly, Sara Etienne, Sara Ghanem, Sara Kharsa, Sara Mahmoud Abdel-Kader, Sara Mamdouh Matter, Sara María Contreras Mérida, Sara Mehrez, Sara Mohammed, Sara W Al-Saqqa, Sarah Abdelghany, Sarah Antar, Sarah Benammi, Sarah Braungart, Sarah Hafez, Sarah Rayne, Sarah Sahel, Sarah Samy, Saraibrahim Ahmed, Saskia Highcock, Saud Aljohani, Saulius Bradulskis, Saulius Mikalauskas, Savino Occhionorelli, Savni Satoskar, Sawsan Adel Awad, Sayed Sarwary, Sayeda Nazmum Nahar, Sayeeda Aktar Tori, Sayinthen Vivekanantham, Scott K D'amours, Sean Mizzi, Sebastian Bernardo Shu Yip, Sebastian King, Sebastian Shu, Sebastian Sierra, Sebastien Gaujoux, Sebestian Shu, Sefeldin Mahdi, Selina Chiu, Selina Man Yeng Chiu, Semay Desta, Serena Manfreda, Serge Kapenda Tshisola, Sergio Estupinian, Sergio Ribaldi, Sergio Zegarra, Servio Tulio Torres Rodriguez, Shadid Al Amin, Shadid Alamin, Shady Elhadry, Shady Hussein, Shady Mahmoud, Shagorika Talukder, Shahadatul Shaharuddin, Shahinaz Alaa El-Din, Shaimaa Aql, Shalon Guevara Torres, Shamsudeen Aliyu, Sharad Karandikar, Sharon Koh, Shaza Rabie Mohamed, Shereen Elsheikh, Sherif Shehata, Sherif Tariq, Shimaa Gamal, Shimaa Said Elkholy, Shireen Gaafar, Shirish Tewari, Shiva Dindyal, Shivanee Tharmalingam, Shorouk El Mesery, Shpetim Ymeri, Shravan Nadkarni, Shruti Ayyar, Shu Ning Kong, Shuang Yi Teo, Shyam Gokani, Shyang Yee Lim, Silje Holte, Silvia Basilicò, Silvia Boni, Silvia De Franciscis, Simon George Gosling, Simon Gosling, Simon Ng, Simon Stock, Simona Juciute, Simona Kasputyte, Simone Conci, Simone Sandler, Simone Targa, Sir Young Yam, Siti Mohd Desa Asilah, Siti Nur Alia Kamarulzamil, Sivasuriya Sivaganesh, Siyaka Itopa Suleiman, Siyi Chung, Soaad Elsobky, Sofia Mouttalib, Soha Abushamleh, Sohaila Elmihy, Soliman Magdy Ahmed, Sondos Turkustani, Sophian Hmila, South Africa, Srinivas Pai, Sriram Bhat, SS Prasad, Stassen Paul, Stavros Parasyris, Stefan Botes, Stefan Breitenstein, Stefan Zammit, Stefano Berti, Stefano Cucumazzo, Stefano M.M Basso, Stefano Roncali, Stella Binna Kim, Sten Saar, Stephanie Hiu-wai Kwok, Stephanie Van Straten, Stephen Dias, Stephen J Chapman, Stephen Kache, Stephen Mcaleer, Stephen R Knight, Stephen Tabiri, Steponas Petrikenas, Stuart J Fergusson, Styliani Parpoudi, Stylianos Germanos, Sudipta Roy, Sukrit Suresh, Sule Burger, Suleiman Baba, Sultan Almuallem, Sung-Hee Kim, Sunil Kumar, Suparna Das, Suraya Bahar, Susan Aviles, Susan Limache, Susan Wndy Mathew, Susana Yrma Aranzabal Durand, Svetlana Doris Brincat, Swantje Kruspi, Swapnil Roy, Syed Abdul Wahhab Eusoffee Wan Ali, Syed Altaf Naqvi, Syed Asaat ul Razi, Sylvia Batista Lemaire, Sylvie Mochet, Syrine Rekhis, T Ariani Widiastini, Tagang Ebogo Ngwa, Taha Yusufali, Taher Al-taher, Tahir Muhammad Yaseen, Tahir Yaseen, Tahira Naqvi, Taiwo Akeem Lawal, Taiwo Lawal, Tan Arulampalam, Tanzeela Gala, Tapan Kumar, Tara Grima, Tarek Ezzat, Tarek Razek, Tasneem Idress, Tasnia Hamid Kanta, Tatsiana Shachykava, Taufiq Khan, Tebian Hassanein Ahmed Ali, Tessa Fautz, Tewodros Worku, Thamer Nouh, Thays Brunelli Pugliesi, Thea Dimech, Thelma Tembo, Thelma Xerri, Theodore Pezas, Theodosios Theodosopoulos, Thiago Fernandes Giuriato, Thierry Alihonou, Thomas Feidantsis, Thomas Fozard, Thomas G Weiser, Thomas M Drake, Thomas Olagboyega Olajide, Thomas Pinkney, Thomas Prudhomme, Thomas Sherman, Thomas Tetens Moe, Thuraya Alzayat, Thusitha Sampath Hettiarachchi, Tien Seng Bryan Lee, Timothy White, Tina Gaarder, Tobias Schuetz, Todisoa Emmanuella Christina Tolotra, Tolg Cecilia, Tom AM Malik, Tom Arthur, Tom Falconer Hall, Tomas Abaliksta, Tomas Jankus, Tomas Poškus, Tommaso Bocchetti, Tommaso Campagnaro, Tommaso Fontana, Tony Mak, Toqa Khafagy, Torhild Veen, Trude Beate Wold, Tsz-Yan Katie Chan, Tuan Nur'Azmah Tuan Mat, Tunde Sholadoye, TWC Mak, Tyler Rouse, Tzu-Ling Chen, Uday Muddebihal, Ufuk Karabacak, Ulf Gunnarsson, Ulf Gustafsson, Umar Muktar, Umberto Tedeschi, Umme Salma, Usama Hantour, Uthman Alamoudi, Valdemaras Jotautas, Valentine Parent, Vanessa Dina Palomino Castillo, Vanessa Msosa, Vania Guglielmo, Vania Silvestri, Vasileios Despotidis, Vasileios Kalles, Vasiliki Soulou, Vassilis Kalles, Veereanna Shatkar, Venerand Barendegere, Veronica Grassi, Veronica Lazzari, Vicky Jennings, Victor Dassah, Victor Etwire, Victor Kong, Victor Manuel Quintero Riaza, Victor Nwinee, Victoria K Proctor, Vijaid Upadhyaya, Vijay Gadhvi, Viktorija Ambrozeviciute, Viktorija Nevieraite, Ville Sallinen, Vimalakanthan Thanusan, Vincas Jonas Banaitis, Virgilijus Beisa, Viviana Sollazzo, Vivien Graffieille, Vizir Jean Paul Nsengimana, Vladimir Khokha, Vu Thanh Hien Le, Vytautas Gaižauskas, Vytautas Lipnickas, Wahid Anwer, Wai Cheong Soon, Wai Him Lam, Wairimu Ndegwa, Waleed Thabet, Walid Adham, Walter Forno, Walter Ruiz Panez, Wan Nurul ‘Ain Wan Mohd Nasir, Wanigasekara Senanayake Mudiyanselage Kithsiri Janakantha Senanayake, Ward Hamsho, Wasim Dar, Wedyan Alhazmi, Wei Guo, Weiguang Ho, Weihei Dao, Wendy Leslie Messa Aguilar, Wennweoi Goh, Wifanto Saditya Jeo, Wilfredo Pino, William Appeadu-Mensah, William Beasley, William Bonney, William Hutch, William J. Lossius, William Milligan, Willy Alcca Ticona, Wing Sum Li, Witold Chachulski, Xavier Delforge, Xianelle Rodriguez, Xinwei Low, Xue Wei Chan, Ya Theng Neo, Yacoubou Imorou Souaibou, Yahaya Ukwenya, Yahya Salama, Yaseen Rajjoub, Yasmein Ibrahim, Yasmin Abd-Elrasoul, Yasmin Elfouly, Yasmin Hegazy, Yasmin Soliman, Yasser Abd El Salam, Yee Wen Tan, Yehia Zakaria, Yella Reddy, Yi Koon Tan, Yi Ting Mok, Yih Jeng Cheong, Yiing Yee Gan, Yishan Der, Yogendra Praveen Mogan, Yomna Allam, Yomna Hosny Asar, Yong Yong Tew, Yousef Abuowda, Yousra El Shoura, Ysabel Esthefany Alejos Bermúdez, Yücel Cengiz, Yuk Hong Eric Cheung, Yuksel Altinel, Yung Kok Ng, Yuri Macchitella, Yves Aigrain, Zaher Mikwar, Zahra Jaffry, Zain Ali Khan, Zainab Iftikhar, Zaynab M Elsayed, Zhongtao Zhang, Zi Hao Sam, Zigmantas Urniežius, Zilvinas Dambrauskas, Zineb Bentounsi, Zygimantas Tverskis.

## Supplementary Material

znac195_Supplementary_Data
