# Supplementary material for: Global economic burden of unmet surgical need for appendicitis
Source: Br J Surg. 2022 Jul 26;109(10):995–1003. doi: 10.1093/bjs/znac195 (PMC10364778; doi:10.1093/bjs/znac195)
Supplement: znac195_Supplementary_Data [file znac195_supplementary_data.zip › Appendix 13-07-22.docx]

# **Appendix:**

## Authorship

**Writing committee**

Anna Reuter, Lisa Rogge, Mark Monahan, Mwayi Kachapila, Dion G. Morton, Justine Davies, Sebastian Vollmer

**Data development committee**

Anna Reuter, Lisa Rogge, Mark Monahan, Mwayi Kachapila, James Glasbey, Maja Marcus, Alexander J Fowler, Mohamed Gulamhussein, Alasdair Ball, Dmitri Nepogodiev

**Collaborators**

*GlobalSurg Collaborative writing group*: Hannah S Thomas, Thomas G Weiser, Thomas M Drake, Stephen R Knight, Cameron Fairfield, Adesoji O Ademuyiwa, Maria Lorena Aguilera, Philip Alexander, Sara W Al-Saqqa, Giuliano Borda-Luque, Ainhoa Costas-Chavarri, Faustin Ntirenganya, J Edward Fitzgerald, Stuart J Fergusson, James Glasbey, J.C. Allen Ingabire, Lawani Ismaïl, Hosni Khairy Salem, Anyomih Theophilus Teddy Kojo, Marie Carmela Lapitan, Richard Lilford, Andre L Mihaljevic, Dion Morton, Alphonse Zeta Mutabazi, Dmitri Nepogodiev, Adewale O Adisa, Riinu Ots, Francesco Pata, Thomas Pinkney, Tomas Poškus, Ahmad Uzair Qureshi, Antonio Ramos-De la Medina, Sarah Rayne, Catherine A Shaw, Sebastian Shu, Richard Spence, Neil Smart, Stephen Tabiri, Aneel Bhangu, Ewen M Harrison

*GlobalSurg Collaborative study guarantor*: Ewen M Harrison

*GlobalSurg Collaborative patient representatives*: Azmina Verjee, Emmy Runigamugabo

*GlobalSurg Collaborative protocol development*: Adesoji O Ademuyiwa, Adewale O Adisa, Maria Lorena Aguilera, Afnan Altamini, Philip Alexander, Sara W Al-Saqqa, Giuliano Borda-Luque, Jen Cornick, Ainhoa Costas-Chavarri, Thomas M Drake, Stuart J Fergusson, J Edward Fitzgerald, James Glasbey, J.C Allen Ingabire, Lawani Ismaïl, Zahra Jaffry, Hosni Khairy Salem, Chetan Khatri, Andrew Kirby, Anyomih Theophilus Teddy Kojo, Marie Carmela Lapitan, Richard Lilford, Andre L Mihaljevic, Midhun Mohan, Dion Morton, Alphonse Zeta Mutabazi, Dmitri Nepogodiev, Faustin Ntirenganya, Riinu Ots, Francesco Pata, Thomas Pinkney, Tomas Poškus, Ahmad Uzair Qureshi, Antonio Ramos-De la Medina, Sarah Rayne, Gustavo Recinos, Kjetil Søreide, Catherine A Shaw, Sebastian Shu, Richard Spence, Neil Smart, Stephen Tabiri, Ewen M Harrison, Aneel Bhangu

*National leads (GlobalSurg 1):*

**Lead coordinator,** Chetan Khatri; **Australia,** Neel Gobin; **Brazil**, Ana Vega Freitas; **Canada,** Nigel Hall; **China & Hong Kong,** Sung-Hee Kim; **Egypt**, Ahmed Negida, Hosni Khairy; **England**, Zahra Jaffry, Stephen J Chapman; **France**, Alexis P Arnaud; **Ghana**, Stephen Tabiri; **Guatemala**, Gustavo Recinos; **India**, Cutting Edge Manipal, Midhun Mohan; **Indonesia**, Radhian Amandito; **Iraq**, Marwan Shawki; **Ireland**, Michael Hanrahan; **Italy**, Francesco Pata; **Lithuania**, Justas Zilinskas; **Malaysia**, April Camilla Roslani, Cheng Chun Goh; **Nigeria**, Adesoji O Ademuyiwa; **Northern Ireland**, Gareth Irwin; **Peru**, Sebastian Shu, Laura Luque; **Saudi** **Arabia**, Hunain Shiwani, Afnan Altamimi, Mohammed Ubaid Alsaggaf; **Scotland**, Stuart J Fergusson; **South** **Africa**, Richard Spence, Sarah Rayne; **Sri** **Lanka**, Jenifa Jeyakumar; **Sweden**, Yucel Cengiz; **Switzerland**, Dmitri A Raptis; **Wales**, James C Glasbey.

*National leads (GlobalSurg 2)*:

**Argentina,** Maria Marta Modolo; **Australia**, Dushyant Iyer, Sebastian King, Tom Arthur; **Bangladesh,** Sayeda Nazmum Nahar; **Barbados,** Ade Waterman; **Benin,** Lawani Ismaïl; **Botswana,** Michael Walsh; **Canada,** Arnav Agarwal, Augusto Zani, Mohammed Firdouse, Tyler Rouse; **China,** Qinyang Liu; **Colombia,** Juan Camilo Correa; **Egypt,** Hosni Khairy Salem; **Estonia,** Peep Talving; **Ethiopia,** Mengistu Worku; **France**, Alexis Arnaud; **Ghana,** Stephen Tabiri; **Greece,** Vassilis Kalles; **Guatemala**, Maria Lorena Aguilera, Gustavo Recinos; **India**, Basant Kumar, Sunil Kumar; **Indonesia**, Radhian Amandito; **Ireland**, Roy Quek; **Italy**, Francesco Pata, Luca Ansaloni; **Jordan**, Ahmed Altibi; **Lithuania**, Donatas Venskutonis, Justas Zilinskas, Tomas Poskus; **Madagascar**, John Whitaker; **Malawi**, Vanessa Msosa; **Malaysia**, Yong Yong Tew; **Malta**, Alexia Farrugia, Elaine Borg; **Mexico**, Antonio Ramos-De la Medina; **Morocco**, Zineb Bentounsi; **Nigeria**, Adesoji O Ademuyiwa; **Norway**, Kjetil Søreide; **Pakistan,** Tanzeela Gala; **Palestinian Territory,** Ibrahim Al-Slaibi, Haya Tahboub, Osaid H. Alser; **Peru,** Diego Romani, Sebestian Shu; **Poland,** Piotr Major; **Romania**, Aurel Mironescu, Matei Bratu, Amar Kourdouli; **Saint Kitts and Nevis,** Aliyu Ndajiwo; **Saudi Arabia,** Abdulaziz Altwijri, Mohammed Ubaid Alsaggaf, Ahmad Gudal, Al Faifi Jubran; **Sierra Leone,** Sam Seisay; **Singapore,** Bettina Lieske; **South Africa,** Sarah Rayne, Richard Spence; **Spain,** Irene Ortega; **Sri Lanka,** Jenifa Jeyakumar, Kithsiri J. Senanayake; **Sudan,** Omar Abdulbagi; **Sweden,** Yucel Cengiz; **Switzerland,** Dmitri Raptis; **Turkey,** Yuksel Altinel; **United Kingdom,** Chia Kong, Ella Teasdale, Gareth Irwin, Michael Stoddart, Rakan Kabariti, Sukrit Suresh; **United States,** Katherine Gash, Ragavan Narayanan; **Zambia,** Mayaba Maimbo

*Local collaborators (GlobalSurg 1):*

**Argentina:** Claudio Fermani, Ruben Balmaceda, Maria Marta Modolo (Hospital Luis Lagomaggiore). **Australia:** Ewan Macdermid, Neel Gobin, Roxanne Chenn, Cheryl Ou Yong, Michael Edye (Blacktown Hospital), Martin Jarmin, Scott K D'amours, Dushyant Iyer (Liverpool Hospital, The University Of New South Wales), Daniel Youssef, Nicholas Phillips, Jason Brown (Royal Brisbane & Women's Hospital), Robert George, Cherry Koh, Oliver Warren (The Royal Prince Alfred Hospital, Australia), Isaac Hanley (The Tweed Hospital), Marilla Dickfos (Toowoomba Hospital). **Austria:** Clemens Nawara, Dietmar Öfner, Florian Primavesi (Department Of Surgery, Paracelsus Medical University Salzburg). **Bangladesh:** Ashrarur Rahman Mitul, Khalid Mahmud (Dhaka Shishu (Children) Hospital), Margub Hussain, Hafiz Hakim, Tapan Kumar (Dhaka Medical College Hospital), Antje Oosterkamp (Lamb Hospital). **Benin:** Pamphile A Assouto, Ismail Lawani, Yacoubou Imorou Souaibou (Centre National Hospitalier Et Universitaire Hubert Koutoukou Maga). **Brunei:** Aung Kyaw Tun, Chean Leung Chong, (Pmmpmhamb Hospital) Giridhar H Devadasar, Chean Leung Chong, Muhammad Rashid Minhas Qadir, (Ssb Hospital), Kyaw Phyo Aung, Lee Shi Yeo, Chean Leung Chong (Ripas Hospital). **Brazil:** Vanessa Dina Palomino Castillo, Monique Mor0n Munhoz, Gisele Moreira (Conjunto Hospitalar De Sorocaba), Luiz Carlos Barros De Castro Segundo, Salim Anderson Khouri Ferreira, Maíra Cassa Careta (Hospital Da Santa Casa De Misericórdia De Vitória), Stella Binna Kim, Alexandre Venancio De Sousa, Alyne Daltri Lazzarini Cury (Hospital De Caridade Sãƒo De Paula), Gustavo Peixoto Soares Miguel, Ana Vega Carreiro De Freitas, Barbara Pereira Silvestre (Hospital Estadual Doutor Jayme Dos Santos Neves), Julia Guasti Pinto Vianna, Carolina Oliveira Felipe, Luis Alberto Valente Laufer (Hospital Estadual Doutor Jayme Santos Neves), Fernanda Altoe, Luana Ayres Da Silva, Marina Luiza Pimenta, Thiago Fernandes Giuriato, Paulo Alves Bezerra Morais, Jessica Souza Luiz (Hospital Estadual Dr Jayme Santos Neves), Rafael Araujo, Juliana Menegussi, Marisa Leal, Caio Vinícius Barroso de Lima, Luiza Sarmento Tatagiba, Antônio Leal (Hospital Infantil Nosa Senhora Da Gloria), Diogo Vinicius dos Santos, Gustavo Pereira Fraga, Romeo Lages Simoes (Hospital De Clinicas, University Of Campinas). **Cambodia:** Simon Stock (World Mate Emergency Hospital). **Cameroon:** Samuel Nigo, Juana Kabba, Tagang Ebogo Ngwa, James Brown (Mbingo Baptist Hospital). **Canada:** Sebastian King, Augusto Zani, Georges Azzie, Mohammed Firdouse, Sameer Kushwaha, Arnav Agarwal (The Hospital For Sick Children), Karen Bailey, Brian Cameron, Michael Livingston (McMaster Children's Hospital), Alexandre Horobjowsky, Dan L Deckelbaum, Tarek Razek (Centre for Global Surgery, McGill University Health Centre). **Chile:** Boris Marinkovic, Eugenio Grasset, Nicole D'aguzan (Hospital Del Salvador), Eugenio Grasset, Julio Jimenez, Roberto Macchiavello (Hospital Luis Tisne). **China:** Zhongtao Zhang, Wei Guo, Junyeong Oh, Fei Zheng (Beijing Friendship Hospital). **Colombia:** Irene Montes, Sebastian Sierra, Manuela Mendez (Clinica Ces), Maria Isabel Villegas, Maria Clara Mendoza Arango, Ivan Mendoza, (Clinica Las Vegas), Fred Alexander Naranjo Aristizã¡bal, Jaime Andres Montoya Botero, Victor Manuel Quintero Riaza (Hospital Pablo Tobon Uribe), Jakeline Restrepo, Carlos Morales, Maria Clara Mendoza Arango (Hospital Universitario San Vicente Fundacion), Herman Cruz, Alejandro Munera, Maria Clara Mendoza Arango (Ips Universitaria Clinica Leon Xiii). **Croatia:** Robert Karlo, Edgar Domini, Jakov Mihanovic (Zadar General Hospital), Mihael Radic, Kresimir Zamarin, Nikica Pezelj (General Hospital Sibenik). **Dominican Republic:** Manuel Hache-Marliere, Sylvia Batista Lemaire, Ruben Rivas (Cedimat - Centro De Diagnostico Medicina Avanzada, Laboratorio Y Telemedicina). **Egypt:** Ahmed Khyrallh, Ahamed Hassan, Gamal Shimy, Mohamed A Baky Fahmy (Al-Azher Universty Hospital); Ayman Nabawi, Mohamed Elfil, Mohamed Ghoneem, Muhammad El-Saied Ahmad Muhammad Gohar, Mohamed Asal, Mostafa Abdelkader, Mahmoud Gomah, Hayssam Rashwan, Mohamed Karkeet, Ahmed Gomaa (Alexandria Main University Hospital); Amr Hasan, Ahmed Elgebaly, Omar Saleh, Ahmad Abdel Fattah, Abdullah Gouda, Abd Elrahman Elshafay, Abdalla Gharib, Ahmed Menshawy, Mohammed Hanafy, Abdullah Al-Mallah, Mahmoud Abdulgawad, Mohamad Baheeg, Mohammed Alhendy, Ibrahim AbdelFattah, Abdalla Kenibar, Omar Osman, Mostafa Gemeah, Ahmed Mohammed, Abdalrahman Adel, Abdalla Gharib, Abdelrahman Mohammed, Abdelrahman Sayed, Mohamed Abozaid (Al-Hussein Hospital); Ahmed Hafez El-Badri Kotb, Ali Amin Ahmed Ata, Mohammed Nasr, Abdelrahman Alkammash, Mohammed Saeed, Nader Abd El Hamid, Attia Mohamed Attia, Ahmed Abd El Galeel, Eslam Elbanby, Khalid Salah El-Dien, Usama Hantour, Omar Alahmady, Billal Mansour, Amr Muhammad Elkorashy (Bab El-Shareia Hospital); Emad Mohamed Saeed Taha, Kholod Tarek Lasheen, Salma Said Elkolaly, Nehal Yosri Elsayed Abdel-Wahab, Mahmoud Ahmed Fathi Abozyed, Ahmed Adel, Ahmed Moustafa Saeed, Gehad Samir El Sayed, Jehad Hassan Youssif (Banha University Hospital); Soliman Magdy Ahmed, Nermeen Soubhy El-Shahat, Abd El-Rahman Hegazy Khedr (Belbes Central Hospital); Abdelrhman Osama Elsebaaye, Mohamed Elzayat, Mohamed Abdelraheim, Ibrahim Elzayat, Mahmoud Warda, Khaled Naser El Deen, Abdelrhman Essam Elnemr, Omar Salah, Mohamed Abbas, Mona Rashad, Ibrahim Elzayyat, Dalia Hemeda, Gehad Tawfik, Mai Salama, Hazem Khaled, Mohamed Seisa, (El Dawly Hospital - Mansoura); Kareem Elshaer, Abdelfatah Hussein, Mahmoud Elkhadrawi (El Mahalla General Hospital); Ahmed Mohamed Afifi, Osama Saadeldeen Ebrahim, Mahmoud Mohamed Metwally (El - Mataria Educational Hospital); Rowida Elmelegy, Diaa Moustafa Elbendary Elsawahly, Hisham Safa, Eman Nofal, Mohamed Elbermawy, Ahmed Abdelmotaleb Ghazy, Hisham Samih, Asmaa Abdelgelil, Sarah Abdelghany, Ahmed El Kholy, Metwally Aboraya, Fatma Elkady, Mahmoud Salma, Sarah Samy, Reem Fakher, Aya Aboarab, Ahmed Samir, Ahmed Sakr, Abdelrahman Haroun, Asmaa Abdel-Rahman Al-Aarag, Ahmed Elkholy, Sally Elshanwany (El-Menshawy Hospital); Esraa Ghanem (Elshohadaa Central Hospital); Ahmed Tammam, Ali Mohamed Hammad, Yousra El Shoura, Gehad El Ashal, Hosni Khairy (Kasr Alainy School Of Medicine); Sarah Antar, Sara Mehrez, Mahmoud Abdelshafy, Maha Gamal Mohamad Hamad, Mona Hosh, Emad Abdallah, Basma Magdy, Thuraya Alzayat, Elsayed Gamaly, Hossam Elfeki, Amany Abouzahra, Shereen Elsheikh, Fatimah I Elgendy (Mansoura University Hospitals); Fathia Abd El-Salam, Osama Seifelnasr, Mohamed Ammar, Athar Eysa, Aliaa Sadek, Aliaa Gamal Toeema, Aly Nasr, Mohamed Abuseif, Hagar Zidan, Sara Abd Elmageed Barakat, Nadin Elsayed, Yasmin Abd Elrasoul, Ahmed Elkelany, Mohamed Sabry Ammar, Mennat-Allah Mustafa, Yasmin Hegazy, Mohamed Etman, Samar Saad, Mahmoud Alrahawy, Ahmed Raslan, Mahmoud Morsi, Ahmed Rslan, Ahmed Sabry, Hager Elwakil, Heba Shaker, Hagar Zidan, Yasmin Abd-Elrasoul, Ahmed Elkelany (Menoufia University Hospitals); Hussein El-Kashef, Mohamed Shaalan, Areej Tarek (Minia University Hospital); Ayman Elwan, Ahmed Ragab Nayel, Mostafa Seif, Ayman Elwan, Doaa Emadeldin, Mohamed Ali Ghonaim, Ahmad Almallah, Ahmed Fouad, Eman Adel Sayma (New Damietta University Hospital); Ahmad Elbatahgy, Angham Solaiman El-Ma'doul, Ahmed Mosad, Hager Tolba, Diaa Eldin Abdelazeem Amin Elsorogy, Hassan Ali Mostafa, Amira Atef Omar, Ola Sherief Abd El Hameed, Ahmed Lasheen (Quweisna Central Hospital In Quweisna); Yasser Abd El Salam, Ashraf Morsi, Mohammed Ismail (Ras El Tin General Hospital); Hager Ahmed El-badawy, Mohamed A Amer, Ahmed Elkelany, Ahmed Elkelany, Ahmed Sabry El-Hamouly, Noura A. Attallah, Omnia Mosalum, Ahmed Afandy, Ahmed Mokhtar, Alaa Abouelnasr, Sara Ayad, Ramdan Shaker, Rokia Sakr, Ramadan Shaker, Mahmoud Amreia, Soaad Elsobky, Mohamed Mustafa, Ahmed Abo El Magd, Abeer Marey, Amr Tarek Hafez, Mohamed F Zalabia (Shebin Elkom Teaching Hospital, Menoufia); Mohamed Moamen Mohamed, Amr Fadel, Emad Ali Ahmed (Sohag University Hospital); Ahmad Ali, Mohammad Ghassan Alwafai, Abdullah Dwydar, Sara Kharsa, Ehab Mamdouh, Hatem El-Sheemy, Ibrahim AlYoussef, Abouelatta Khairy Aly, Ahmad Aldalaq, Ehab Alnawam, Dalia Alkhabbaz (Souad Kafafi University Hospital); Mahmoud Saad, Shady Hussein, Ahmed Abo Elazayem, Ahmed Meshref, Marwa Elashmawy, Mohammed Mousa, Ahmad Nashaat, Sara Ghanem, Zaynab M Elsayed, Aya Elwaey, Iman Elkadsh (Suez Canal University Hospitals); Mariam Darweesh, Ahmed Mohameden, Mennaallah Hafez (Suez General Hospital); Ahmed Badr, Assmaa Badwy, Mohamed Abd El Slam (Talla Central Hospital); Mohamed Elazoul, Safwat Al-Nahrawi, Lotfy Eldamaty, Fathee Nada, Mohamed Ameen, Aya Hagar, Mohamed Elsehimy, Mohammad Aboraya, Hossam Dawoud, Shorouk El Mesery, Abeer El Gendy, Ahmed Abdelkareem, Ahmed Safwan Marey, Mostafa Allam, Sherif Shehata, Khaled Abozeid, Marwa Elshobary, Ahmed Fahiem, Sameh Sarsik, Amel Hashish, Mohamed Zidan, Mohamed Hashish, Shaimaa Aql, Abdelaziz Osman Abdelaziz Elhendawy (Tanta University Hospital); Mohamed Husseini, Esraa Kasem, Ahmed Gheith, Yasmin Elfouly, Ahmed Ragab Soliman, Yasmein Ibrahim, Nesma Elfouly, Ahmed Fawzy, Ahmed Hassan, Mohammad Rashid, Abdallah Salah Elsherbiny, Basem Sieda, Nermin M Badwi, Mohammed Mustafa Hassan Mohammed, Osama Mohamed, Mohammad Abdulkhalek Habeeb (Zagazig University Hospitals). **Ethiopia:** Mengistu Worku, Nichole Starr (Dessie Referral Hospital), Semay Desta, Sahlu Wondimu, Nebyou Seyoum Abebe (Minilik Ii Hospital), Efeson Thomas, Frehun Ayele Asele, Daniel Dabessa (Myungsung Christian Medical Center), Nebiyou Seyoum Abebe, Abebe Bekele Zerihun (Tikur Anbessa Hospital). **Finland:** Panu Mentula, Ari Leppäniemi, Ville Sallinen (Helsinki University Central Hospital). **France:** Aurelien Scalabre, Fernanda Frade, Sabine Irtan (Trousseau Hospital Sorbonnes Universités, Upmc Univ Paris), Vivien Graffeille, Elodie Gaignard, Quentin Alimi (Chu De Rennes), Quentin Alimi, Vivien Graffieille, Elodie Gaignard (Fr Rennes University), Olivier Abbo, Sofia Mouttalib, Ourdia Bouali (Hopital Des Enfants), Erik Hervieux, Yves Aigrain, Nathalie Botto (Hopital Necker Enfants Malades), Alice Faure, Lucile Fievet, Nicoleta Panait (Hopitâl Nord), Emilie Eyssartier, Francoise Schmitt, Guillaume Podevin (Pediatric Surgery Department, University Hospital Of Angers, France), Valentine Parent, Amandine Martin, Alexis Pierre Arnaud (Rennes University Hospital), Cecile Muller, Arnaud Bonnard, Matthieu Peycelon (Robert Debré Children University Hospital). **Ghana:** Francis Abantanga, Kwaku Boakye-Yiadom, Mohammed Bukari (Komfo Anokye Teaching Hospital), Frank Owusu (Offinso District Hospital), Joseph Awuku-Asabre, Stephen Tabiri, Lemuel Davies Bray (University For Development Studies, School Of Medicine And Health Sciences, General Surgery Department, Tamale Teaching Hospital). **Greece:** Dimitrios Lytras, Kyriakos Psarianos, Anastasia Bamicha (Achillopoyleio General Hospital Of Volos), Eirini Kefalidi (Attikon General Hospital), Georgios Gemenetzis (Attikon University Hospital), Christos Dervenis, Nikolaos Gouvas, Christos Agalianos (Konstantopouleio General Hospital Of Athens), Michail Kontos, Gregory Kouraklis, Dimitrios Karousos (Laiko University Hospital), Stylianos Germanos, Constantinos Marinos (Larissa General Hospital), Christos Anthoulakis, Nikolaos Nikoloudis, Nikolaos Mitroudis (Serres General Hospital). **Guatemala:** Gustavo Recinos, Sergio Estupinian, Walter Forno (Hospital De Accidentes Ceibal), José René Arévalo Azmitia (Hospital General De Enfermedades, Cirugia De Emergencia, Instituto Guatemalteco De Seguridad Social), Carla Cecilia Ramã­rez Cabrera (Hospital General De Enfermedades, Servicio De Cirugia Abdominal, Instituto Guatemalteco De Seguridad Social), Romeo Guevara, Maria Aguilera, Napoleon Mendez, Cesar Augusto Azmitia Mendizabal, Pablo Ramazzini, Mario Contreras Urquizu (Hospital General San Juan De Dios), Fernando Tale, Rafael Soley, Emanuel Barrios, Emmanuel Barrios (Hospital Juan Jose Arevalo Bermejo), Daniel Estuardo Marroquín Rodríguez, Carlos Iván Pérez Velásquez, Sara María Contreras Mérida (Hospital Regional De Retalhuleu), Francisco Regalado, Mario Lopez, Miguel Siguantay (Hospital Roosevelt, Guatemala). **Hong Kong:** Fong Yee Lam, Kylie Joan-yi Szeto, Charing Cheuk Ling Szeto, Wing Sum Li, Kieran Ka Kei Li, Man Fung Leung, Tony Mak, Simon Ng (Prince of Wales Hospital). **India:** SS Prasad, Anand Kirishnan, Nidhi Gyanchandani (KMC Hospital), Bylapudi Seshu Kumar, Muthukumaran Rangarajan (Kovai Medical Centre & Hospital), Sriram Bhat, Anjana Sreedharan, S.V. Kinnera (Kasturba Medical College), Yella Reddy, Caranj Venugopal, Sunil Kumar (Pes Institute Of Medical Sciences & Research), Abhishek Mittal (Safdarjung Hospital,New Delhi), Shravan Nadkarni, Harish Neelamraju Lakshmi, Puneet Malik (Sawai Man Singh Medical College & Hospitals, Jaipur, Rajasthan), Neel Limaye, Srinivas Pai, Pratik Jain (Sdm College Of Medical Sciences And Hospital), Monty Khajanchi, Savni Satoskar, Rajeev Satoskar (Seth Gordhandas Sunderdas Medical College And King Edward Memorial Hospital), Abid Bin Mahamood (Travancore Medical College Hospital). **Indonesia:** Eldaa Prisca Refianti Sutanto, Daniel Ardian Soeselo, Chintya Tedjaatmadja (Atmajaya Hospital), Fitriana Nur Rahmawati, Radhian Amandito, Maria Mayasari (Dr Cipto Mangunkusumo General Hospital, Jakarta). **Iraq:** Ruqaya Kadhim Mohammed Jawad Al-Hasani, Hasan Ismael Ibraheem Al-Hameedi, Hasan Ismael Ibraheem, Israa Abdullah Aziz Al-Azraqi (Alsader Medical City), Lubna Sabeeh, Rahma Kamil, Marwan Shawki (Baghdad Medical City), Muwaffaq Mezeil Telfah (Department Of Surgery, College Of Medicine, University Of Mosul, Al-Jumhoori Teaching Hospital). **Ireland:** Amoudtha Rasendran, Jacqueline Sheehan, Robert Kerley, Caoimhe Normile, Richard William Gilbert, Jiheon Song, Mohamed Dablouk, Linnea Mauro, Mohammed Osman Dablouk, Michael Hanrahan, Paul Kielty, Eleanor Marks (Cork University Hospital), Simon Gosling, Michelle Mccarthy, Amoudtha Rasendran (Cork University Hospital & University College Cork), Diya Mirghani, Syed Altaf Naqvi, Chee Siong Wong (Limerick University Hospital), Siyi Chung, Reuban D'cruz, Ronan Cahill (Mater Misericordiae University Hospital), Simon George Gosling, Michelle Mccarthy, Amoudtha Rasendran, Ciara Fahy, Jiheon Song, Michael Hanrahan, Diana Duarte Cadogan, Anna Powell, Richard Gilbert, Caroline Clifford, Caoimhe Normile, Aoife Driscoll (Mercy University Hospital), Stassen Paul, Chris Lee, Ross Bowe (Midlands Regional Hospital Mullingar), William Hutch, Michael Hanrahan (University College Cork), Helen Mohan, Maeve O'neill, Kenneth Mealy (Wexford General Hospital). **Italy:** Piergiorgio Danelli, Andrea Bondurri, Anna Maffioli (Azienda Ospedaliera Luigi Sacco - Polo Universitario), Mario Pasini, Giacomo Pata, Stefano Roncali (Azienda Ospedaliera Spedali Civili Di Brescia - Chirurgia Generale), Paolo Silvani, Michele Carlucci, Roberto Faccincani (Irccs Ospedale San Raffaele), Luigi Bonavina, Yuri Macchitella, Chiara Ceriani (University of Milan, IRCCS Policlinico San Donato), Gregorio Tugnoli, Salomone Di Saverio, Khaled Khattab (Maggiore Hospital), Miguel Angel Paludi, Domenica Pata, Luigi Maria Cloro (Nicola Giannettasio Hospital), Andrea Allegri, Luca Ansaloni, Federico Coccolini (Papa Giovanni Xxiii Hospital), Ezio Veronese, Luca Bortolasi, Alireza Hasheminia (San Bonifacio Hospital), Giacomo Nastri, Massimiliano Dal Canto, Stefano Cucumazzo (Santa Croce Hospital), Francesco Pata, Angelo Benevento, Gaetano Tessera (Sant'Antonio Abate Hospital, Gallarate), Pier Paolo Grandinetti, Alessio Maniscalco, Giovanni Luca Lamanna (Santi Benvenuto E Rocco Hospital Asur), Luca Turati, Giovanni Sgroi, Emanuele Rausa (Treviglio Hospital), Roberta Villa, Michela Monteleone, David Merlini (Unita' Di Chirurgia D'urgenza Azienda Ospedaliera Salvini), Federico Coccolini, Luca Ansaloni, Andrea Allegri (Unit Of General Surgery I, Papa Giovanni Xxiii Hospital), Veronica Grassi, Roberto Cirocchi, Alban Cacurri (University Of Perugia). **Libya:** Hamza Waleed, Ahmed Diab, Fathi Elzowawi (Misurata Central Hospital). **Lithuania:** Mantas Jokubauskas, Karolis Varkalys, Donatas Venskutonis (Kaunas Clinical Hospital), Robertas Pranevicius, Viktorija Ambrozeviciute (Klaipedas Seaman Hospital), Simona Juciute, Austė Skardžiukaitė (Lietuvos Sveikatos Mokslų Universitetas), Donatas Venskutonis, Saulius Bradulskis, Linas Urbanavicius, Aiste Austraite, Romualdas Riauka, Justas Zilinskas, Zilvinas Dambrauskas (Lithuanian University Of Health Sciences), Paulius Karumnas, Zigmantas Urniezius, Reda Zilinskiene (Republic Hospital Of Kaunas), Anele Rudzenskaite (Republic Hospital Of Panevezys), Ausrine Usaityte, Margarita Montrimaite, Nerijus Kaselis (Republic Klaipeda Hospital), Andrius Strazdas, Kristijonas Jokubonis (Stasys Kudirka Regional Hospital Of Alytus), Kornelija Maceviciute, Virgilijus Beisa, Tomas Poskus, Kestutis Strupas, Erikas Laugzemys (Vilnius University, Center Of Abdominal Surgery), Andrej Kolosov, Valdemaras Jotautas, Ignas Rakita (Vilnius University Hospital Santariskiu Clinics), Saulius Mikalauskas, Darius Kazanavicius, Rokas Rackauskas, Kestutis Strupas, Tomas Poskus, Virgilijus Beisa (Vilnius University Hospital Santariskiu Klinikos), Ritauras Rakauskas, Egle Preckailaite (Vsi Jonavos Ligonine). **Malawi:** Ross Coomber, Kenneth Johnson, Jennifer Nowers (Queen Elizabeth Hospital). **Malaysia:** Dineshwary Periasammy, Afizah Salleh, Andre Das (Hospital Kajang), Reuben Goh Ern Tze, Milaksh Nirumal Kumar, Nik Azim Nik Abdullah (Sarawak General Hospital), Nik Ritza Kosai, Mustafa Taher, Reynu Rajan (Universiti Kebangsaan Malaysia Medical Centre), Hoong Yin Chong, April Camilla Roslani, Cheng Chun Goh (University Malaya Medical Centre). **Malta:** Marija Agius, Elaine Borg, Maureen Bezzina, Roberta Bugeja, Martinique Vella-Baldacchino, Andrew Spina, Josephine Psaila (Mater Dei Hospital, Malta). **Martinique:** Helene Francois-Coridon, Cecilia Tolg, Jean-Francois Colombani (Department Of Pediatric Surgery, Mother And Children's Hospital, University Hospital Of Martinique). ***Mexico*:** Carmina Diaz-Zorrilla, Antonio Ramos-De La Medina, Samantha Corro-Diaz Gonzalez (Hospital Español de Veracruz). **Mozambique:** Mário Jacobe, Domingos Mapasse, Elizabeth Snyder (Hospital Central Maputo). **New Zealand:** Ramadan Oumer, Mohammed Osman (Whangarei Hospital, Northland District Health Board). **Nigeria:** Aminu Mohammad, Lofty-John Anyanwu, Abdulrahman Sheshe (Aminu Kano Teaching Hospital), Alaba Adesina, Olubukola Faturoti, Ogechukwu Taiwo (Babcock University Teaching Hospital), Muhammad Habib Ibrahim, Abdulrasheed A Nasir, Siyaka Itopa Suleiman (Federal Medical Centre, Birnin Kebbi), Adewale Adeniyi, Opeoluwa Adesanya, Ademola Adebanjo (Federal Medical Centre), Roland Osuoji, Kazeem Atobatele, Ayokunle Ogunyemi, Omolara Williams, Mobolaji Oludara, Olabode Oshodi (Lagos State University Teaching Hospital), Adesoji Ademuyiwa, AbdulRazzaq Oluwagbemiga Lawal, Felix Alakaloko, Olumide Elebute, Adedapo Osinowo, Christopher Bode(Lagos University Teaching Hospital), Abidemi Adesuyi (National Hospital Abuja), Adesoji Tade, Adeleke Adekoya, Collins Nwokoro (Olabisi Onabanjo University Teaching Hospital),Omobolaji O Ayandipo, Taiwo Akeem Lawal, Akinlabi E Ajao (University College Hospital),Samuel Sani Ali, Babatunde Odeyemi, Samson Olori (University of Abuja Teaching Hospital),Ademola Popoola, Ademola Adeyeye, James Adeniran (University of Ilorin Teaching Hospital). **Norway:** William J. Lossius (Department Of Gastrointestinal Surgery, St. Olavs Hospital, Trondheim University Hospital), Ingemar Havemann (Soerlandet Hospital Kristiansand), Kenneth Thorsen, Jon Kristian Narvestad, Kjetil Soreide (Stavanger University Hospital), Trude Beate Wold, Linn Nymo (University Hospital Of North Norway, Troms). **Oman:** Mohammed Elsiddig, Manzoor Dar (Sohar Hospital). **Pakistan:** Kamran Faisal Bhopal, Zainab Iftikhar, Muhammad Mohsin Furqan (Bahawal Victoria Hospital), Bakhtiar Nighat, Masood Jawaid, Abdul Khalique (Dow University Hospital), Ahsan Zil-E-Ali, Anam Rashid (Fatima Memorial Hospital), Hasnain Abbas Dharamshi, Tahira Naqvi, Ahmad Faraz (Karachi Medical And Dental College, Abbasi Shaheed Hospital), Abdul Wahid Anwar, Tahir Muhammad Yaseen, Ghina Shamim Shamsi, Ghina Shamsi, Tahir Yaseen, Wahid Anwer (The Indus Hospital). **Paraguay:** Horacio Paredes Decoud, Omar Aguilera, Ismael Isaac Zelada Alvarez, Juan Marcelo Delgado, Gustavo Miguel Machain Vega, Helmut Alfredo Segovia Lohse (Hospital De Clínicas). **Peru:** Wendy Leslie Messa Aguilar, Jose Antonio Cabala Chiong, Ana Cecilia Manchego Bautista (Carlos Alberto Seguin Escobedo National Hospital. Essalud), Eduardo Huaman, Sergio Zegarra, Rony Camacho (Hospital Necional Guillermo Almenara), Jose María Vergara Celis, Diego Alonso Romani Pozo (Hospital De Emergencias Pediatricas), José Hamasaki, Edilberto Temoche, Jaime Herrera-Matta (Hospital De Policia), Carla Pierina García Torres, Luis Miguel Alvarez Barreda, Ronald Renato Barrionuevo Ojeda (Hospital Goyeneche), Octavio Garaycochea (Hospital Ii -1 Minsa Moyobamba), Melanie Castro Mollo, Mitchelle Solange De Fã Tima Linares Delgado, Francisco Fujii (Hospital Maria Auxiliadora), Ana Cecilia Manchego Bautista, Wendy Leslie Messa Aguilar, Jose Antonio Cabala Chiong (Hospital Nacional Carlos Alberto Seguin), Susana Yrma Aranzabal Durand, Carlos Alejandro Arroyo Basto, Nelson Manuel Urbina Rojas (Hospital Nacional Edgardo Rebagliati Martins-Essalud), Sebastian Bernardo Shu Yip, Ana Lucia Contreras Vergara, Andrea Echevarria Rosas Moran, Giuliano Borda Luque, Manuel Rodriguez Castro, Ramon Alvarado Jaramillo (Hospital Nacional Cayetano Heredia), George Manrique Sila, Crislee Elizabeth Lopez, Mardelangel Zapata Ponze De Leon, Massiell Machaca, Ronald Coasaca Huaraya, Andy Arenas, Crislee López, Clara Milagros Herrera Puma, Wilfredo Pino, Christian Hinojosa, Melanie Zapata Ponze De Leon, Susan Limache, George Manrrique Sila, Layza-Alejandra Mercado Rodriguez (Hospital Regional Honorio Delgado Espinoza). **Portugal:** Renato Melo, Jose Costa-Maia, Nuno Muralha (Servico De Cirurgia Geral - Centro Hospitalar Sao Joao - Porto). **Reunion:** Frederique Sauvat (Chu Reunion). **Romania:** Ionasc Dan, Mircea Hogea, Pandi Eduard (Emergency Clinical Hospital Brasov), Razvan-Matei Bratu, Mircea Beuran, Ionut-Bogdan Diaconescu, Bogdan-Valeriu Martian, Florin-Mihail Iordache, Mihaela Vartic (Emergency Clinical Hospital Bucharest), Lucian Corneliu Vida, Liviu Iuliu Muntean, Aurel Sandu Mironescu (Spitalul Clinic De Copii Brasov). **Rwanda:** Vizir Jean Paul Nsengimana (Chuk), Alice Niragire, Jean De La Croix Allen Ingabire, Eugene Niyirera (University Teaching Hospital Of Kigali). **San Marino:** Nicola Zanini, Elio Jovine, Giovanni Landolfo (San Marino State Hospital). **Saudi Arabia:** Ibrahim N. Alomar, Saleh A. Alnuqaydan, Abdulrahman M. Altwigry (Buraydah Central Hospital), Moayad Othman, Nohad Osman (Imam Abdulrahman Al Faisal Hospital), Enas Alqahtani (King Abdulaziz Hospital Al Ahsa National Guard), Mohammed Alzahrani, Rifan Alyami, Emad Aljohani (King Abdulaziz Medical City), Ibrahim Alhabli, Zaher Mikwar, Sultan Almuallem (King Abdulaziz Medical City (King Khalid National Guard Hospital, Jeddah), Emad Aljohani, Rifan Alyami, Mohammed Alzahrani (King Abdulaziz Medical City, Riyadh), Abrar Nawawi, Mohamad Bakhaidar, Ashraf A. Maghrabi, Mohammed Alsaggaf, Murad Aljiffry, Abdulmalik Altaf, Ahmad Khoja, Alaa Habeebullah, Nouf Akeel (Department of Surgery, Faculty of Medicine, King Abdulaziz University, Jeddah, Saudi Arabia), Nashat Ghandora, Abdullah Almoflihi, Abdulmalik Huwait (King Fahad General Hospital), Abeer Al-shammari, Mashael Al-Mousa (King Fahad Hospital), Masood Alghamdi, Walid Adham, Bandar Albeladi, Muayad Ahmed Alfarsi, Atif Mahdi, Saad Al Awwad (King Fahd Hospital), Afnan Altamimi, Thamer Nouh, Mazen Hassanain (King Khaled University Hospital, King Saud University), Salman Aldhafeeri, Nawal Sadig, Osama Algohary (King Khalid General Hospital), Mohannad Aledrisy, Ahmad Gudal, Ahmad Alrifaie (King Khalid National Guard Hospital), Mohammed AlRowais, Amani Althwainy (Department of Surgery, King Saud University), Alaa Shabkah, Uthman Alamoudi, Mawaddah Alrajraji (National Guard Hospital), Basim Alghamdi, Saud Aljohani, Abdullah Daqeeq (Rcymc), Jubran J Al-Faifi (Security Forces Hospital). **South Africa:** Vicky Jennings, Nyawira Ngayu, Rachel Moore (Chris Hani Baragwanath Academic Hospital), Victor Kong (Edendale Hospital), Hayden Kretzmann, Katie Connor, Daniel Nel (Frere Hospital), Colleen Sampson, Richard Spence, Eugenio Panieri (Groote Schuur), Sarah Rayne, Nosisa Sishuba (Helen Joseph Hospital, Department Of Surgery, University Of The Witwatersrand), Myint Tun, Albert Mohale Mphatsoe, Jo-Anne Carreira (Leratong Hospital), Ella Teasdale, Mark Wagener (Ngwelezana Hospital), Stefan Botes, Danelo Du Plessis (Rob Ferreira Hospital). **Spain:** Fernando Fernandez-Bueno (Hospital Central De La Defensa Gomez Ulla), Jose Aguilar-Jimenez, Jose Andres Garcia-Marin (Hospital Morales Meseguer. Sms), Lorena Solar García, Luis Joaquín García Florez, Rubén Darío Arias Pacheco (Hospital San Agustín), Janet Pagnozzi, Jimy Harold Jara Quezada, Jose Luis Rodicio, German Minguez, Raquel Rodríguez-Uría, Paul Ugalde, Camilo Lopez-Arevalo, Luis Barneo, Jessica Patricia Gonzales Stuva (Hospital Universitario Central De Asturias), Irene Ortega-Vazquez, Lorena Rodriguez, Norberto Herrera (Severo Ochoa University Hospital). **Sri Lanka:** Prasad Pitigala Arachchi, Wanigasekara Senanayake Mudiyanselage Kithsiri Janakantha Senanayake, Lalith Asanka Jayasooriya Jayasooriya Arachchige (Department Of General Surgery, Teaching Hospital Kandy), Sivasuriya Sivaganesh, Dulan Irusha Samaraweera, Vimalakanthan Thanusan (National Hospital Of Sri Lanka). *Sudan*: Ahmed Elgaili Khalid Musa, Reem Mohammed Hassan Balila, Mohamed Awad Elkarim Hamad Mohamed (Ibrahim Malik Teaching Hospital), Hussein Ali, Hagir Zain Elabdin, Alaa Hassan (Jarash International Specialized Hospital), Sefeldin Mahdi, Hala Ahmed, Sahar Abdoun Ishag Idris (Khartoum Teaching Hospital), Makki Elsayed, Mohammed Elsayed, Mohamed Mahmoud (Omdurman Teaching Hospital). **Sweden:** Magnus Boijsen, Per-Olof Lundgren (Capio St Goran Hospital), Ulf Gustafsson, Ali Kiasat (Danderyds Hospital), Fredrik Wogensen, Fredrik Wogensen, Emma Jurdell, Anders Thorell (Department Of Surgery, Ersta Hospital, Stockholm), Hildur Thorarinsdottir, Maria Utter (Helsingborgs Lasarett), Sami Martin Sundstrom (Hudiksvall Sjukhus), Cecilia Wredberg, Ann Kjellin (Karolinska Universitetssjukhuset), Johanna Nyberg, Bjorn Frisk (Skaraborg Hospital Skovde), Malin Sund, Linda Andersson, Ulf Gunnarsson (Department Of Surgical And Perioperative Sciences, Umea University and Umea University Hospital), Yücel Cengiz, Sandra Ahlqvist, Ida Björklund (Sundsvall Hostpital) Hanna Royson, Per Weber (Vaexjoe Central Hospital), Hans-Ivar Pahlsson, Eva Borin (Visby Hospital, Department Of Surgery), Maria Hjertberg (Vrinnevi Hospital), Hanna Royson, Per Weber (Vaxjo Central Hospital). **Switzerland:** Roger Schmid, Debora Schivo, Vasileios Despotidis (Bürgerspital Solothurn), Stefan Breitenstein, Ralph F Staerkle, Erik Schadde (Kantonsspital Winterthur), Fabian Deichsel, Alexandra Gerosa, Antonio Nocito (Kantonsspital Baden), Dimitri Aristotle Raptis, Barbara Mijuskovic, Markus Zuber, Lukas Eisner (Kantonsspital Olten), Swantje Kruspi, Katharina Beate Reinisch, Christin Schoewe (Kreisspital Fuer Das Freiamt Muri Ag), Allan Novak, Adrian F. Palma, Gerfried Teufelberger (Kreisspital Muri, Department Of Surgery). **Tanzania:** Msafiri Kimaro, Rachel King (Mbalizi Christian Designated Hospital). **Turkey:** Ali Zeynel Abidin Balkan, Mehmet Gumar, Mehmet Ali Yavuz (Harran University Research And Treatment Hospital), Ufuk Karabacak, Gokhan Lap, Bahar Busra Ozkan (Ondokuz Mayis University, Medical Faculty), Bahar Busra Ozkan, Murat Karakahya (Ordu University Training And Research Hospital). **United Kingdom:** Ryan Adams, Robert Morton, Liam Henderson, Ruth Gratton, Keiran David Clement, Kate Yu-Ching Chang, David Mcnish, Ryan Mcintosh, William Milligan (Aberdeen Royal Infirmary), Brendan Skelly, Hannah Anderson-Knight, Roger Lawther (Altnagelvin Area Hospital), Jemina Onimowo, Veereanna Shatkar, Shivanee Tharmalingam (Barking, Havering And Redbridge University Hospitals Trust (Queens Hospital, Romford), Evelina Woin, Tessa Fautz, Oliver Ziff (Barnet General Hospital), Shiva Dindyal, Sam Arman, Shagorika Talukder, Sam Arman, Vijay Gadhvi, Shagorika Talukder (Basildon And Thurrock University Foundation Trust), Luen Shaun Chew, Jonathan Heath (Blackpool Victoria Teaching Hospitals), Natalie Blencowe, Sally Hallam, Katherine Gash (Bristol Royal Infirmary), Gurdeep Singh Mannu, Dimitris-Christos Zachariades, Ailsa Claire Snaith (Buckinghamshire NHS Trust), Thusitha Sampath Hettiarachchi, Arjun Nesaratnam, James Wheeler (Cambridge University Hospitals NHS Foundation Trust), Darragh McCullagh, Joshua Michael Clements, Ata Khan (Causeway Hospital), Foteini Koumpa, Christina Neophytou, Jessica Roth, Wai Cheong Soon, Mohammed Deputy, Ahmed Ahmed, Annelisse Ashton, Joe Vincent, Jack Almy, Taufiq Khan, John Lee Y Allen, Charlotte Jane Mcintyre, Dominic Charles Marshall (Charing Cross Hospital, Imperial College Healthcare NHS Trust), Mark Sykes, Nebil Behar, Harriet Jordan (Chelsea And Westminster Hospital), Yaseen Rajjoub, Thomas Sherman (Cheltenham General Hospital), Timothy White, Anna Watts, Rohan Ardley (Chesterfield Royal Hospital NHS Foundation Trust), Tan Arulampalam, Apar Shah, Damien Brown (Colchester Hospital University NHS Foundation Trust), Emma Blower, Paul Sutton, Konstantinos Gasteratos, Dale Vimalachandran (Countess Of Chester Hospital), Cathy Magee, Gareth Irwin, Andrew Mcguigan (Craigavon Area Hospital), Stephen Mcaleer, Clare Morgan (Daisy Hill Hospital), Sarah Braungart (Department Of Paediatric Surgery, Leeds General Infirmary), Kirsten Lafferty, Peter Labib, Andrei Tanase, Clodagh Mangan, Lillian Reza, Lillian Reza, Andrei Tanase, Clodagh Mangan (Derriford Hospital), Helen Woodward, Craig Gouldthorpe, Megan Turner (Diana Princess Of Wales Hospital), Jonathan R L Wild, Tom AM Malik, Victoria K Proctor (Doncaster Royal Infirmary NHS Foundation Trust), Kalon Hewage, James Davies (Dorset County Hospital), Andre Dubois, Sayed Sarwary, Ali Zardab, Alan Grant, Robert Mcintyre (Dr Grays Hospital), Yogendra Praveen Mogan, Weiguang Ho, Bryon Frankie Hon Khi Chong (Dumfries And Galloway Royal Infirmary), Shirish Tewari, Gemma Humm, Eriberto Farinella (East And North Hertfordshire NHS Trust Lister Hospital) Nigel J Hall, Naomi J Wright, Christina P Major (Evelina Children's Hospital), Thelma Xerri, Phoebe De Bono, Jasim Amin, Mustafa Farhad, John F. Camilleri-Brennan, Andrew G N Robertson, Thelma Xerri, Joanna Swann, James Richards, Jasim Amin, Aijaz Jabbar, Phoebe De Bono, Myranda Attard, Hannah Burns, Euan Macdonald, Matthew Baldacchino, Jennifer Skehan, Julian Camilleri-Brennan (Forth Valley Royal Hospital), Tom Falconer Hall, Madelaine Gimzewska, Greta Mclachlan (Frimley Park Hospital), Jamie Shah, James Giles (George Elliot Hospital), Selina Chiu, Beatrix Weber, Selina Man Yeng Chiu, Saskia Highcock (Gilbert Bain Hospital), Maleeha Hassan, William Beasley, Apostolos Vlachogiorgos, Stephen Dias, Geta Maharaj, Rosie Mcdonald (Glangwili General Hospital), Alisdair Macdonald, Paul Witherspoon, Alan Baird (Glasgow Southern General Hospital), Panchali Sarmah, Nikki Green, Haney Youssef (Good Hope Hospital), Kate Cross, Clare M Rees, Bernard Van Duren (Great Ormond Street Hospital For Children NHS Foundation Trust), Emma Upchurch (Great Western Hospital), Khurram Khan, Haytham Abudeeb, Ahmed Hammad (Hairmyres Hospital, NHS Lanarkshire), Sharad Karandikar, Doug Bowley, Ahmed Karim (Heart Of England Foundation Trust), Witold Chachulski, Liam Richardson, Giles Dawnay, Ben Thompson, Ajayesh Mistry, Aneel Bhangu, Millika Ghetia, Sudipta Roy, Ossama Al-Obaedi, Millika Ghetia, Kaustuv Das (Hereford County Hospital), Ash Prabhudesai, DM Cocker, Jessica Juliana Tan (Hillingdon Hospital), Robert Tyler, Filippo Di Franco, Shruti Ayyar (Hinchingbrooke Hospital), Sayinthen Vivekanantham, Shyam Gokani (Imperial College London), Michael Gillespie, Katrin Gudlaugsdottir (Inverclyde Royal Hospital), Theodore Pezas, Chelise Currow, Matthew Young-Han Kim (Ipswich Hospital NHS Trust), Amerdip Birring, Joanne Edwards, Ased Ali, Suparna Das, Madan Jha, Kieran Atkinson (James Cook University Hospital), Joshua Luck, Thomas Fozard, Michael Puttick (John Radcliffe Hospital), Yahya Salama, Rohi Shah, Ahmad Aboelkassem Ibrahem, Hamdi Ebdewi, Gianpiero Gravante, Saleem El-Rabaa (Kettering General Hospital), Henry Nnajiuba, Rebecca Allott, Aman Bhargava (King George Hospital), Zoe Chan, Zaffar Hassan (Kings College Hospital), Misty Makinde, David Hemingway, Ramzana Dean, Alexander Boddy, Ahmed Aber, Vijay Patel (Leicester Royal Infirmary), Jehangirshaw Parakh (Leighton Hospital - Mid Cheshire NHS Foundation Trust), Sunil Parthiban (Lister Hospital), Harmony Kaur Ubhi, Simon-Peter Hosein (Luton And Dunstable Hospital), Simon Ward, Kamran Malik (Macclesfield District General Hospital), Leifa Jennings, Tom Newton, Mirna Alkhouri, Min Kyu Kang, Christopher Houlden, Jonathan Barry (Morriston Hospital), Imtanan Raza, Alistair Farquharson, Sanjeet Bhattacharya (NHS Ayrshire), William Milligan, Kate Chang, Liam Henderson (NHS Grampian), Michael S J Wilson, Yan Ning Neo, Ibrahim Ibrahim, Emily Chan, Fraser S Peck, Pei J Lim, Alexander S North, Rebecca Blundell, Adam Williamson (Ninewells Hospital, NHS), Dina Fouad, Ashish Minocha (Norfolk And Norwich University Hospital), Kathryn Mccarthy, Emma Court, Alice Chambers (North Bristol NHS Trust), Jenna Yee, Ji Chung Tham, Ceri Beaton (North Devon District Hospital), Una Walsh, Joseph Lockey, Salman Bokhari, Lara Howells, Megan Griffiths, Laura Yallop (Northwick Park Hospital), Shailinder Singh, Omar Nasher, Paul Jackson (Nottingham Children's Hospital At Queens Medical Centre Campus), Michael Puttick, Joshua Luck, Thomas Fozard (Oxford University Hospitals), Abdul Muiz Shariffuddin, Weng Chee Ho, Michael Sj Wilson, Gurpreet Pabla (Perth Royal Infirmary), Saed Ramzi, Shady Zeidan, Jennifer Doughty (Plymouth Hospitals NHS Trust), Sidhartha Sinha, Ross Davenport, Jason Lewis (Princess Alexandra Hospital), Leo Duffy, Elizabeth Mcaleer, Eleanor Williams (Princess Of Wales Hospital), Robin Som, Omar Javed (Queen Elizabeth Hospital Woolwich), Matthew Boal, Nicola Harrison, Habib Tafazal, Omar Javed, Tom Brogden, Dmitri Nepogodiev, Ewen Griffiths (Queen Elizabeth Hospital Birmingham) Rhalumi Daniel Obute, Thomas E Glover, David J Clark (Queen Elizabeth Hospital King's Lynn), Mohamed Boshnaq, Mansoor Akhtar, Pascale Capleton, Samer Doughan, Mohamed Rabie, Ismail Mohamed (Queen Elizabeth The Queen Mother Hospital), Duncan Samuel, Lauren Dickson, Matthew Kennedy, Eleanor Dempster, Emma Brown, Natalie Maple, Eimear Monaghan, Bernhard Wolf, Alicia Garland (Raigmore Hospital), Arthur Mcphee, David Anderson, Robert Anderson (Royal Alexandria Hospital), Sarah Hassan, Paul Sutton, Dave Smith (Royal Bolton Hospital), Jonathan Lund, Catherine Boereboom, Jennifer Murphy, Gillian Tierney, Samson Tou (Royal Derby Hospital), Eleanor Franziska Zimmermann, Neil James Smart, Andrea Marie Warwick (Royal Devon And Exeter National Health Service Foundation Trust), Theodora Stasinou, Ian Daniels, Kim Findlay-Cooper (Royal Devon & Exeter NHS Foundation Trust), Stefan Mitrasinovic, Swayamjyoti Ray, Massimo Varcada, Rovan D'Souza, Sharif Omara (Royal Free Hospital), Matthew Spurr, Lucienne Parkinson, Anthony Hanks (Royal Glamorgan Hospital), Jennifer Ma, Emily Abington, Meera Ramcharn, Gethin Williams (Royal Gwent Hospital), Joseph Winstanley, Ewan D. Kennedy, Emily NW Yeung (Royal Hospital For Sick Children), Stuart J Fergusson, Catrin Jones, Stephen O'neill, Shujing Jane Lim, Ignatius Liew, Hari Nair, Cameron Fairfield, Julia Oh, Samantha Koh, Andrew Wilson, Catherine Fairfield (Royal Infirmary Of Edinburgh), Delran Anandkumar, Ashok Kirupagaran, Timothy F Jones, Hew Dt Torrance, Alexander J Fowler, Charmilie Chandrakumar, Priyank Patel, Syed Faaz Ashraf, Sonam M. Lakhani, Aaron Lawson Mclean, Sonia Basson (Royal London Hospital), Jeremy Batt, Catriona Bowman, Michael Stoddart, Natasha Benons (Royal United Hospital Bath), Clare Mason, Rebecca Harrison, John Quayle (Salford Royal NHS Foundation Trust), Tom Barker, Virginia Summerour, Edward Harper (Sandwell And West Birmingham Hospitals NHS Trust), Caroline Smith, Matthew Hampton (Sheffield Children's Hospital), Sophie K Pitt, Alex E Ward, Timothy O’Connor, Emily G Heywood, Thomas M Drake (Sheffield Teaching Hospitals NHS Foundation Trust), Abeed Chowdhury, Sina Hossaini, Nicholas Fs Watson (Sherwood Forest Hospitals NHS Foundation Trust), Doug Mckechnie, Ayaan Farah, Anita Chun (Southend University Hospital), Hoey Koh, Grace Lim, Graham Sunderland (Southern General Hospital), Laura Gould, Alice Chambers (Southmead Hospital), P C Munipalle, H Rooney, D R L Browning (Southmead Hospital, North Bristol NHS Trust), Bernadette Pereira, Kristof Nemeth, (St Georges Healthcare NHS Trust), Emily Decker, Stefano Giuliani, Aly Shalaby (St.George's Healthcare NHS Trust And University), Shafaque Shaikh, Chern Yan Tan, Ebrahim Y A Palkhi (St. James's University Hospital), Aleksandra Szczap, Swathikan Chidambaram, Chee Yang Chen, Kavian Kulasabanathan, Srishti Chhabra (St Mary's Hospital), Elisabeth Kostov, Philippe Harbord, James Barnacle (St. Mary's Hospital), Madan Mohan Palliyil, Mina Zikry, Johnathan Porter, Charef Raslan, Mohammed Saeed, Shazia Hafiz, Niksa Soltani, Katie Baillie (Stockport NHS Foundation Trust), Priyanka Singh, Shailee Sheth, Kishen Patel, Mahry Khalili, Jeesoo Choi, Matthew Benger (St Thomas' Hospital), Lucy Marples, Alastair Macfarlane, Ramesh Thurairaja (St. Thomas Hospital), Tamsin Boyce, Harriet Whewell, Elin Jones (The Royal Gwent Hospital), Francesca Th'ng, Nichola Robertson (The Royal Infirmary Of Edinburgh), Ahmad Mirza, Haroon Saeed, Simon Galloway (The University Hospital Of South Manchester), Gia Elena, Mohammad Afzal, Mohamed Zakir (United Lincolnshire Hospitals - Pilgrim Hospital), Peter Sodde, Charles Hand, Aiesha Sriram, Tamsyn Clark, Patrick Holton, Amy Livesey (University Hospital Coventry And Warwickshire), Yashashwi Sinha, Fahad Mujtaba Iqbal, Indervir Singh Bharj (University Hospital Of North Midlands), Adriana Rotundo, Cara Jenvey, Robert Slade (University Hospital Of North Staffordshire NHS Trust), David Golding, Samuel Haines, Ali Adel Ne'ma Abdullah, Thomas W Tilston, Dafydd Loughran, Danielle Donoghue, Lorenzo Giacci, Mohamed Ashur Sherif, Peter Harrison, Alethea Tang (University Hospital Of Wales), Deevia Kotecha (University Hospitals Leicester - Leicester Royal Infirmary), Mohamed Elshaer, Tomas Urbonas, Amjid Riaz, Annie Chapman, Parisha Acharya, Joseph Shalhoub (Watford General Hospital), Cathleen Grossart, David McMorran (Western General Hospital), Makhosini Mlotshwa, William Hawkins, Sofronis Loizides (Western Sussex Hospitals NHS Trust), Kandaswamy Krishna, Melanie Orchard, Chik Wai Ho (Weston General Hospital), Peter Thomson, Shahab Khan, Fiona Taylor, Jalak Shukla, Emma Elizabeth Howie (Whipps Cross University Hospital), Linda Macdonald, Olusegun Komolafe, Neil Mcintyre (Wishaw General Hospital), James Cragg, Jody Parker, Duncan Stewart (Wrexham Maelor Hospital), Luke Lintin, Julia Tracy, Tahir Farooq (Yeovil District Hospital). **United States:** George Molina, Haytham Kaafarani, Laura Luque (Massachusetts General Hospital), Robel Beyene, Jack Sava, Mark Scott (Medstar Washington Hospital Center), Mamta Swaroop, Raelene Kennedy (Northwestern Memorial Hospital), Ijeoma A Azodo, Daithi Heffernan, Tristen Chun, Andrew Stephen (The Rhode Island Hospital), Melanie Sion, Michael S. Weinstein, Viren Punja (Thomas Jefferson University Hospital), Nikolay Bugaev, Monica Goodstein, Shadi Razmdjou (Tufts Medical Center), Eric Etchill, Juan Carlos Puyana, Matthew Kesinger (University of Pittsburgh Medical Center - Presbyterian Hospital), Lena Napolitano, Kathleen To, Mark Hemmila (University Of Michigan). **Zambia:** Oliver Todd, Edward Jenner, Ellen Hoogakker (St Francis Hospital).

*Local collaborators (GlobalSurg 2):*

**Albania:** Besmir Grizhja, Shpetim Ymeri, Gezim Galiqi (Spitali Rajonal Shkoder); **Argentina:** Roberto Klappenbach, Diego Antezana, Alvaro Enrique Mendoza Beleño, Cecilia Costa, Belen Sanchez, Susan Aviles (Hospital Zonal General De Agudos Simplemente Evita); Maria Marta Modolo, Claudio Gabriel Fermani, Rubén Balmaceda, Santiago Villalobos, Juan Manuel Carmona (Hospital Luis C. Lagomaggiore, Mendoza); **Australia**: Daniel Hamill, Peter Deutschmann, Simone Sandler, Daniel Cox (Alice Springs Hospital); Ram Nataraja, Claire Sharpin, Damir Ljuhar (Monash Medical Center); Demi Gray, Morgan Haines (Port Macquarie Base Hospital); Dush Iyer, Nithya Niranjan, Scott D'Amours (Liverpool Hospital); Morvarid Ashtari, Helena Franco, (Gold Coast University Hospital). **Bangladesh**: Ashrarur Rahman Mitul, Sabbir Karim, (Dhaka Shishu (Children) Hospital); Nowrin F. Aman, Mahnuma Mahfuz Estee (Holy Family Red Crescent Medical College & Hospital); Umme Salma, Joyeta Razzaque, Tasnia Hamid Kanta (Dhaka Medical College And Hospital); Sayeeda Aktar Tori, Shadid Alamin, Swapnil Roy, Shadid Al Amin, Rezaul Karim (Armed Forces Medical College); Muhtarima Haque, Amreen Faruq, Farhana Iftekhar (Birdem Bangladesh Institute Of Research And Rehabilitation In Diabetes Endocrine And Metabolic Disorder General Hospital); **Barbados**: Margaret O'Shea, Greg Padmore, Ramesh Jonnalagadda (Queen Elizabeth Hospital). **Belarus**: Andrey Litvin, Aliaksandr Filatau, Dzmitry Paulouski, Maryna Shubianok, Tatsiana Shachykava (Gomel Regional Clinical Hospital); Dzianis Khokha, Vladimir Khokha (City Hospital) **Benin**: Fernande Djivoh, Lawani Ismaïl, Francis Dossou (Centre National Hospitalier Et Universitaire Hubert Koutoukou Maga); Djifid Morel Seto, Dansou Gaspard Gbessi, Bruno Noukpozounkou, Yacoubou Imorou Souaibou, (Centre National Hospitalier Et Universitaire Hubert Koutoukou Maga); Kpèmahouton René Keke, Fred Hodonou (Clinique Vignon); Ernest Yemalin Stephane Ahounou, Thierry Alihonou (Hopital El Fateh); Max Dénakpo, Germain Ahlonsou (Hospital Saint Luc). **Botswana**: Alemayehu Ginbo Bedada (Princess Marina Hospital). **Burundi**: Carlos Nsengiyumva, Sandrine Kwizera, Venerand Barendegere (Hopital Militaire De Kamenge). **Cambodia**: Philip Choi, Simon Stock (World Mate Emergency Hospital) **Canada**: Luai Jamal, Mohammed Firdouse, Augusto Zani, Georges Azzie, Sameer Kushwaha, Arnav Agarwal (The Hospital For Sick Children). **China**: Tzu-Ling Chen, Chingwan Yip (Fudan University Affiliated Huashan Hospital). **Colombia**: Irene Montes, Felipe Zapata, Sebastian Sierra (Clinica CES); Maria Isabel Villegas Lanau, Maria Clara Mendoza Arango, Ivan Mendoza Restrepo (Clinica Las Vegas), Sebastian Sierra, Ruben Santiago Restrepo Giraldo, Maria Clara Mendoza Arango (Hospital Universitario San Vicente Fundación). **Croatia**: Edgar Domini, Robert Karlo, Jakov Mihanovic (Zadar General Hospital). **Egypt**: Mohamed Youssef, Hossam Elfeki, Waleed Thabet, Aly Sanad, Gehad Tawfik, Ahmed Zaki, Noran Abdel-Hameed, Mohamed Mostafa, Muhammad Fathi Waleed Omar, Ahmed Ghanem, Emad Abdallah, Adel Denewar, Eman Emara, Eman Rashad, Ahmad Sakr, Rehab Elashry, Sameh Emile (Mansoura University Hospital); Toqa Khafagy, Sara Elhamouly, Arwa Elfarargy, Amna Mamdouh Mohamed, Ghada Saied Nagy, Abeer Esam, Eman Elwy, Aya Hammad, Salwa Khallaf, Eman Ibrahim, Ahmed Saidbadr, Ahmed Moustafa, Amany Eldosouky Mohammed, Mohammed Elgheriany, Eman Abdelmageed, Eman Abd Al Raouf, Esraa Samir Elbanby, Maha Elmasry, Mahitab Morsy Farahat, Eman Yahya Mansor, Eman Magdy Hegazy, Esraa Gamal, Heba Gamal, Hend Kandil, Doaa Maher Abdelrouf, Mohamed Moaty,(Menofiya University Hospital); Dina Gamal, Nada El-Sagheer, Mohamed Salah, Salma Magdy, Asmaa Salah, Ahmed Essam, Ahmed Ali, Mahmoud Badawy, Sara Ahmed, (Beni Suef University Hospital); Mazed Mohamed, Abdelrahman Assal, Mohamed Sleem, Mai Ebidy, Aly Abd Elrazek, Diaaaldin Zahran, Nourhan Adam, Mohamed Nazir, Adel B Hassanein, Ahmed Ismail, Amira Elsawy, Rana Mamdouh, Mohamed Mabrouk, Lopna Ahmed Mohamed Ahmed, Mohamed Hassab Alnaby, Eman Magdy, Manar Abd-Elmawla, Marwan Fahim, Bassant Mowafy, Moustafa Ibrahim Mahmoud, Meran Allam, Muhammad Alkelani, Noran Halim El Gendy, Mariam Saad Aboul-Naga, Reham Alaa El-Din, Alyaa Halim Elgendy, Mohamed Ismail, Mahmoud Shalaby, Aya Adel Elsharkawy, Mahmoud Elsayed Moghazy, Khaled Hesham Elbisomy, Hend Adel Gawad Shakshouk, Mohamed Fouad Hamed, Mai Mohamed Ebidy, Mostafa Abdelkader, Mohamed Karkeet (Alexandria Main University Hospital); Hayam Ahmed, Israa Adel, Mohammad Elsayed Omar, Mohamed Ibrahim, Omar Ghoneim, Omar Hesham, Shimaa Gamal, Karim Hilal, Omar Arafa, Sawsan Adel Awad, Menatalla Salem, Fawzia Abdellatif Elsherif, Nourhan Elsabbagh, Moustafa R. Aboelsoud, Ahmed Hossam Eldin Fouad Rida, Amr Hossameldin, Ethar Hany, Yomna Hosny Asar, Nourhan Anwar, Mohamed Gadelkarim, Samar Abdelhady, Eman Mohamed Morshedy, Reham Saad, Nourhan Soliman, Mahmoud Salama (Alexandria Medical Research Institute); Eslam Ezzat, Arwa Mohamed, Arwa Ibrahim, Alaa Fergany, Sara Mohammed, Aya Reda, Yomna Allam, Hanan Adel Saad, Afnan Abdelfatah, Aya Mohamed Fathy, Ahmed El-Sehily, Esraa Abdalmageed Kasem, Ahmed Tarek Abdelbaset Hassan, Ahmed Rabeih Mohammed, Abdalla Gamal Saad, Yasmin Elfouly, Nesma Elfouly, Arij Ibrahim, Amr Hassaan, Mohammed Mustafa Mohammed, Ghada Elhoseny, Mohamed Magdy, Esraa Abd Elkhalek, Yehia Zakaria, Tarek Ezzat, Ali Abo El Dahab , Mohamed Kelany, Sara Arafa, Osama Mokhtar Mohamed Hassan, Nermin Mohamed Badwi, Ahmad Saber Sleem, Hussien Ahmed, Kholoud Abdelbadeai, Mohamed Abozed Abdullah (Faculty Of Medicine, Zagazig University); Muhammad Amsyar Auni Lokman, Suraya Bahar, Anan Rady Abdelazeam, Abdelrahman Adelshone, Muhammad Bin Hasnan, Athirah Zulkifli, Siti Nur Alia Kamarulzamil, Abdelaziz Elhendawy, Aliang Latif, Ahmad Bin Adnan, Shahadatul Shaharuddin, Aminah Hanum Haji Abdul Majid, Mahmoud Amreia, Dina Al-Marakby, Mahmoud Salma, Mohamad Jeffrey Bin Ismail, Elissa Rifhan Mohd Basir, Citra Dewi Mohd Ali, Aya Yehia Ata (Faculty Of Medicine, Tanta University); Maha Nasr, Asmaa Rezq, Ahmed Sheta, Sherif Tariq, Abd Elkhalek Sallam, Abdelrhman KZ Darwish, Sohaila Elmihy, Shady Elhadry, Ahmed Farag, Haidar Hajeh, Abdelaziz Abdelaal, Amro Aglan, Ahmed Zohair, Mahitab Essam, Omar Moussa, Esraa El-Gizawy, Mostafa Samy, Safia Ali, Esraa Elhalawany, Ahmed Ata, Mohamed El Halawany, Mohamed Nashat, Samar Soliman, Alaa Elazab, Mostada Samy, (El-Menshawy General Hospital); Mohamed A Abdelaziz, Khaled Ibrahim, Ahmed mohamed Ibrahim, Ammar Gado, Usama Hantour (Al-Hussein Hospital); Esraa Alm Eldeen, Mohamed Reda loaloa, Arwa Abouzaid, Mostafa Ahmed Bahaa Eldin, Eman Hashad, Fathy Sroor, Doaa Gamil, Eman Mahmoud Abdulhakeem, Mahmoud Zakaria, Fawzy Mohamed, Marwan Abubakr, Elsayed Ali, Hesham Magdy, Ahmed Refaat, Menna Tallah Ramadan, Mohamed Abdelaty Mohamed, Salma Mansour, Hager Abdul Aziz Amin, Ahmed Rabie Mohamed, Mahmoud Saami, Nada Ahmed Reda Elsayed, Adham Tarek, Sabry Mohy Eldeen Mahmoud, Islam Magdy El Sayed, Amira Reda, Martina Yusuf Shawky, Mohammed Mousa Salem, Shahinaz Alaa El-Din, Noha Abdullah Soliman, Muhammed Talaat, Shahinaz Alaael-Dein, Ahmed Abd Elmoen Elhusseiny, Noha Abdullah, Mohammed Elshaar, Aya Abdel Fatah Ibraheem, Hager Abdulaziz, Mohammed Kamal Ismail, Mona Hamdy Madkor, Mohamed Abdelaty, Sara Mahmoud Abdel-Kader, Osama Mohamed Salah, (Benha Faculty Of Medicine); Mahmoud Eldafrawy, Ahmed Zaki Eldeeb, Mostafa Mahmoud Eid (October 6 University Hospital); Attia Attia, Khalid Salah El-Dien, Ayman Shwky (Bab El-Shareia University Hospital); Mohamed Adel Badenjki, Abdelrahman Soliman, Samaa Mahmoud Al Attar, (The Memorial Soaad Kafafi University Hospital); Farrag Sayed, Fahd Abdel Sabour, Mohammed G. Azizeldine, Muhammad Shawqi, Abdullah Hashim, Ahmed Aamer, Ahmed Mahmoud Abdelraouf, Mahmoud Abdelshakour, Amal Ibrahim, Basma Mahmoud, Mohamed Ali Mahmoud, Mostafa Qenawy, Ahmed M. Rashed, Ahmed Dahy, Marwa Sayed, Ahmed W. Shamsedine, Bakeer Mohamed, Ahmad Hasan, Mahmoud M. Saad, Khalil Abdul Bassit (Assiut University Hospital); Nadia Khalid Abd El-Latif, Nada Elzahed, Ahmed El Kashash, Nada Mohamed Bekhet, Sarah Hafez, Ahmed Gad, Mahmoud Elkhadragy Maher, Ahmed Abd Elsameea, Mohamed Hafez, Ahmad Sabe, Ataa Ahmed, Ahmed Shahine, Khaled Dawood, Shireen Gaafar, Reem Husseiny, Omnia Aboelmagd, Ahmed Soliman, Nourhan Mesbah, Hossam Emadeldin, Amgad Al Meligy, Amira Hassan Bekhet, Doaa Hasan, Khaled Alhady, Ahmad Khaled Sabe, Mahmoud A. Elnajjar, Majed Aboelella, Ward Hamsho, Ihab Hassan, Hala Saad, Galaleldin Abdelazim, Hend Mahmoud, Noha Wael, Ahmedali M Kandil, Ahmed Magdy, Shimaa Said Elkholy, Badr Eldin Adel, Kareem Dabbour, Saged Elsherbiney, Omar Mattar, Abdulshafi Khaled Abdrabou, Mohammed Yahia Mohamed Aly, Abdelrahman Geuoshy, Ahmedglal Elnagar, Saraibrahim Ahmed, Ibrahem Abdelmotaleb, Amr Ahmed Saleh, Manar Saeed, Shady Mahmoud, Badreldin Adel Tawfik, Samar Adel Ismail, Esraay Zakaria, Mariam O. Gad‏, Mohamed Salah Elhelbawy, Monica Bassem, Noha Maraie, Nourhan Medhat Elhadary, Nourhan Semeda, Shaza Rabie Mohamed, Hesham Mohammed Bakry, AA Essam (Kasr Al-Ainy Faculty Of Medicine, Cairo University); Dina Tarek, Khlood Ashour, Alaa Elhadad, Abdulrahman Abdel-Aty, Ibrahim Rakha, Sara Mamdouh Matter, Rasha Abdelhamed, Omar Abdelkader, Ayat Hassaan, Yasmin Soliman, Amna Mohamed, Sara Ghanem, Sara Amr Mohamed Farouk, Eman Mohamed Ibrahim, Esraa El-Taher (Faculty Of Medicine Seuz Canal University); Merna Mostafa, Mohamed Fawzy Mahrous Badr, Rofida Elsemelawy, Aya El-Sawy, Ahmad Bakr, Ahmad Abdel Razaq Al Rafati (Smouha University Hospital); **Estonia**: Sten Saar, Arvo Reinsoo, Peep Talving (The North Estonia Medical Centre); **Ethiopia**: Nebyou Seyoum, Tewodros Worku, Agazi Fitsum, (Addis Ababa University, College Of Health Sciences, School Of Medicine); **Finland:** Matti Tolonen, Ari Leppäniemi, Ville Sallinen (Helsinki University Hospital); **France**: Benoît Parmentier, Matthieu Peycelon, Sabine Irtan (Trousseau Hospital, APHP); Sabrina Dardenne, Elsa Robert (GHICL); Betty Maillot, Etienne Courboin, Alexis Pierre Arnaud, Juliette Hascoet (CHU Rennes); Olivier Abbo, Amir Ait Kaci, Thomas Prudhomme (CHU Toulouse); Quentin Ballouhey, Céline Grosos, Laurent Fourcade (CHU Limoges); Tolg Cecilia, Colombani Jean-Francois, Francois-Coridon Helene (CHU Martinique); Xavier Delforge, Elodie Haraux (CHU Amiens Picardie); Bertrand Dousset, Roberto Schiavone, Sebastien Gaujoux, (Cochin - APHP); Jean-Baptiste Marret, Aurore Haffreingue, Julien Rod (CHU Caen); Mariette Renaux-Petel (CHU Rouen); Jean-François Lecompte, Jean Bréaud, Pauline Gastaldi (CHU Lenval Nice); Chouikh Taieb, Raquillet Claire, Echaieb Anis (Hopital Robert Ballanger, Paris); Nasir Bustangi, Manuel Lopez, Aurelien Scalabre (CHU De Saint Etienne); Maria Giovanna Grella (CHU Poitiers); Aurora Mariani, Guillaume Podevin, Françoise Schmitt (CHU Angers); Erik Hervieux, Aline Broch, Cecile Muller (Hopital Necker Enfants Malades, APHP). **Ghana**: Stephen Tabiri, Anyomih Theophilus Teddy Kojo, Dickson Bandoh, Francis Abantanga, Martin Kyereh, Hamza Asumah, Eric Kofi Appiah, Paul Wondoh (Tamale Teaching Hospital); Adam Gyedu, Charles Dally, Kwabena Agbedinu, Michael Amoah, Abiboye Yifieyeh, (Kwame Nkrumah University of Science and Technology/Komfo Anokye Teaching Hospital); Frank Owusu (St. Particks Hospital); Mabel Amoako-Boateng, Makafui Dayie, Richmond Hagan, Sam Debrah, (Cape Coast Teaching Hosital); Micheal Ohene-Yeboah, Joe-Nat Clegg-Lampety, Victor Etwire, Jonathan Dakubo, Samuel Essoun, William Bonney, Hope Glover-Addy, Samuel Osei-Nketiah, Joachim Amoako, Niiarmah Adu-Aryee, William Appeadu-Mensah, Antoinette Bediako-Bowan, Florence Dedey (Korle Bu Teaching Hospital); Mattew Ekow, Emmanuel Akatibo, Musah Yakubu (Baptist Medical Center, Nalerigu); Hope Edem Kofi Kordorwu, Kwasi Asare-Bediako, Enoch Tackie (Keta Hospital District Hospital, Keta); Kenneth Aaniana, Emmanuel Acquah, Richard Opoku-Agyeman, Anthony Avoka, Kwasi Kusi, Kwame Maison, (Techiman Holy Family Hospital); Frank Enoch Gyamfi (Berekum Holy Family Hospital); Gandau Naa Barnabas, Saiba Abdul-Latif (Upper West Regional Hospital); Philip Taah Amoako (Samapa Government Hospital); Anthony Davor, Victor Dassah (Upper East Regional Hospital); Enoch Dagoe (St. Mary's Hospital); Prince Kwakyeafriyie (Essumejaman Sda Hospital, Dominase); Elliot Akoto, Eric Ackom, Ekow Mensah (Dormaa Presbyterian Hospital); Ebenezer Takyi Atkins, Christian Lari Coompson (Brongho-Ahafo Regional Hospital, Sunyani); **Greece:** Nikolaos Ivros, Christoforos Ferousis, Vasileios Kalles, Christos Agalianos, Ioannis Kyriazanos, Christos Barkolias, Angelos Tselos, Georgios Tzikos, Evangelos Voulgaris (Naval And Veterans Hospital); Dimitrios Lytras, Athanasia Bamicha, Kyriakos Psarianos (Achillopoyleio General Hospital Of Volos); Anastasios Stefanopoulos (General Hospital Of Nafplio, Department Of Surgery); Ioannis Patoulias, Dimitrios Sfougaris, Ioannis Valioulis (G. Gennimatas Hospital); Dimitrios Balalis, Dimitrios Korkolis, Dimitrios K Manatakis (Saint Savvas Cancer Hospital); Georgios Kyrou, Georgios Karabelias, Iason-Antonios Papaskarlatos (General And Oncological Hospital Of Kifissia-Athens); Kolonia Konstantina, Nikolaos Zampitis, Stylianos Germanos (General Hospital Of Larissa); Aspasia Papailia, Theodosios Theodosopoulos, Georgios Gkiokas (2nd Dept Of Surgery, Aretaieion Hospital, National & Kapodistrian University Of Athens School Of Medicine); Magdalini Mitroudi, Christina Panteli, Thomas Feidantsis, Konstantinos Farmakis, (G. Gennimatas General Hospital); Dimitrios Kyziridis, Orestis Ioannidis, Styliani Parpoudi (4th Surgical Department, Aristotle University Of Thessaloniki, General Hospital, Papanikolaou); Georgios Gemenetzis, Stavros Parasyris (Attikon University Hospital); Christos Anthoulakis, Nikolaos Nikoloudis, Michail Margaritis (Serres General Hospital); **Guatemala:** Maria-Lorena Aguilera-Arevalo, Otto Coyoy-Gaitan, Javier Rosales (Hospital General San Juan De Dios); Luis Tale, Rafael Soley, Emmanuel Barrios (Juan Josè Arevalo Bermejo); Servio Tulio Torres Rodriguez, Carlos Paz Galvez, Danilo Herrera Cruz (Hospital San Vicente); Guillermo Sanchez Rosenberg, Alejandro Matheu, David Monterroso Cohen (Hospital Herrera Llerandi); **Haiti:** Marie Paul, Angeline Charles (Hopital Universitaire De Mirebalais); **Hong Kong SAR, China:** Justin Chak Yiu Lam, Man Hon Andrew Yeung, Chi Ying Jacquelyn Fok, Ka Hin Gabriel Li, Anthony Chuk-Him Lai, Yuk Hong Eric Cheung, Hong Yee Wong, Ka Wai Leung, Tien Seng Bryan Lee, Wai Him Lam, Weihei Dao, Stephanie Hiu-wai Kwok, Tsz-Yan Katie Chan, Yung Kok Ng, TWC Mak (Prince Of Wales Hospital); Qinyang Liu, Chi Chung Foo, James Yang, (University Of Hong Kong); **India:** Basant Kumar, Ankur Bhatnagar, Vijaid Upadhyaya, (Sanjay Gandhi Post Graduate Institute Of Medical Sciences); Sunil Kumar (Excelcare Hospital); Uday Muddebihal, Wasim Dar, KC Janardha (Manipal Hospital); Philip Alexander, Neerav Aruldas, (Lady Willingdon Hospital); **Indonesia:** Fidelis Jacklyn Adella, Anthonius Santoso Rulie, Ferdy Iskandar, Jonny Setiawan (Atma Jaya Hospital); Cicilia Viany Evajelista, Hani Natalie, Arlindawati Suyadi (Dr. Oen Surakarta Hospital); Rudy Gunawan, Herlin Karismaningtyas, Lusi Padma Sulistianingsih Mata, Ferry Fitriya Ayu Andika, Afifatun Hasanah, T Ariani Widiastini, Nurlaila Ayu Purwaningsih, Annisa Dewi Fitriana Mukin, Dina Faizatur Rahmah, Hazmi Dwinanda Nurqistan, Hasbi Maulana Arsyad, Novia Adhitama (Rsd Dr Soebandi); Wifanto Saditya Jeo, Nathania Sutandi, Audrey Clarissa, Phebe Anggita Gultom, Matthew Billy, Andreass Haloho, Radhian Amandito, Nadya Johanna, Felix Lee (Rsupn Cipto Mangunkusumo); **Ireland:** Radin Mohd Nurrahman Radin Dorani, Martha Glynn, Mohammad Alherz, Wennweoi Goh, Haaris A. Shiwani, Lorraine Sproule, Kevin C. Conlon (Tallaght Hospital, Trinity College Dublin) **Israel:** Miklosh Bala, Asaf Kedar (Hadassah Hebrew University Medical Center); **Italy:** Luca Turati, Federica Bianco, Francesca Steccanella, (Treviglio Hospital); Gaetano Gallo, Mario Trompetto, Giuseppe Clerico (Department of Colorectal Surgery, S. Rita Clinic, Vercelli); Matteo Papandrea, Giuseppe Sammarco, Rosario Sacco (Department of Medical and Surgical Sciences, Policlinico Universitario Mater Domini Campus Salvatore Venuta, Catanzaro); Angelo Benevento, Francesco Pata, Luisa Giavarini (Sant'Antonio Abate Hospital, Gallarate); Mariano Cesare Giglio, Luigi Bucci, Gianluca Pagano, Viviana Sollazzo, Roberto Peltrini, Gaetano Luglio (Federico II University Of Naples); Arianna Birindelli, Salomone Di Saverio, Gregorio Tugnoli (Maggiore Hospital); Miguel Angel Paludi, Pietro Mingrone, Domenica Pata (Nicola Giannettasio Hospital, Rossano); Francesco Selvaggi, Lucio Selvaggi, Gianluca Pellino, Natale Di Martino (Universitá della Campania “Luigi Vanvitelli”, Naples); Gianluca Curletti, Paolo Aonzo, Raffaele Galleano (Ospedale Santa Corona, Pietra Ligure (SV)); Stefano Berti, Elisa Francone, Silvia Boni, (S. Andrea Hospital, Poll-Asl 5, La Spezia), Laura Lorenzon, Annalisa lo Conte, Genoveffa Balducci (Sant’Andrea Hospital, Sapienza University of Rome); Gianmaria Confalonieri, Giovanni Pesenti (Azienda Ospedaliera Alessandro Manzoni); Laura Gavagna, Giorgio Vasquez, Simone Targa, Savino Occhionorelli, Dario Andreotti (Azienda Ospedaliero-Universitaria Di Ferrara); Giacomo Pata, Andrea Armellini, Deborah Chiesa (A.O. Spedali Civili Di Brescia); Fabrizio Aquilino, Nicola Chetta, Arcangelo Picciariello (Azienda Ospedaliero Universitaria Consorziale Policlinico Di Bari); Mohamed Abdelkhalek, Andrea Belli, Silvia De Franciscis (Istituto Nazionale Tumori Fondazione, Pascale-I.R.C.C.S.); Annamaria Bigaran, Alessandro Favero, Stefano M.M Basso (Azienda Per L'assistenza Sanitaria N. 5 Friuli Occidentale); Paola Salusso, Martina Perino, Sylvie Mochet, Diego Sasia, Francesco Riente, Marco Migliore (Azienda Sanitaria Ospedaliera San Luigi Gonzaga); David Merlini, Silvia Basilicò, Carlo Corbellini (Ospedale Di Rho - ASST Rhodense); Veronica Lazzari, Yuri Macchitella, Luigi Bonavina (IRCCS Policlinico, University of Milano, San Donato); Daniele Angelieri, Diego Coletta, Federica Falaschi, Marco Catani, Claudia Reali, Mariastella Malavenda, Celeste Del Basso, Sergio Ribaldi, Massimo Coletti, Andrea Natili, Norma Depalma, Immacolata Iannone, Angelo Antoniozzi, Davide Rossi (Policlinico Umberto I Emergency Surgery Department); Daniele Gui, Gerardo Perrotta, Matteo Ripa, Francesco Ruben Giardino, Maurizio Foco, (Fondazione Policlinico Universitario Agostino Gemelli); Erika Vicario, Federico Coccolini, Luca Ansaloni, Gabriela Elisa Nita (AO Papa Giovanni XXIII); Nicoletta Leone, Andrea Bondurri, Anna Maffioli (Ospedale Sacco); Andrea Simioni, Davide De Boni, Sandro Pasquali (IOV - Istituto Oncologico Veneto); Elena Goldin, Elena Vendramin, Eleonora Ciccioli (Azienda Ospedaliera di Padova); Umberto Tedeschi, Luca Bortolasi, Paola Violi, Tommaso Campagnaro, Simone Conci, Giovanni Lazzari, Calogero Iacono, Alfredo Gulielmi, Serena Manfreda (Azienda Ospedaliera Universitaria Integrata di Verona); Anna Rinaldi, Maria Novella Ringressi, Beatrice Brunoni (Azienda Ospedaliera Universitaria Careggi); Giuseppe Salamone, Mirko Mangiapane, Paolino De Marco, Antonella La Brocca, Roberta Tutino, Vania Silvestri, Leo Licari, Tommaso Fontana, Nicolò Falco, Gianfranco Cocorullo, (Policlinico Paolo Giaccone di Palermo); Mostafa Shalaby, Pierpaolo Sileri, Claudio Arcudi (Policlinico Tor Vergata Hospital, Rome); **Jordan:** Isam Bsisu, Khaled Aljboor, Lana Abusalem, Aseel Alnusairat, Ahmad Qaissieh, Emad Al-Dakka, Ali Ababneh, Oday Halhouli (Jordan University Hospital); **Kenya:** Taha Yusufali, Hussein Mohammed (Kenyatta National Hospital); Justus Lando, Robert Parker, Wairimu Ndegwa (Tenwek Hospital); **Lithuania:** Mantas Jokubauskas, Jolanta Gribauskaite, Donatas Venskutonis (Lithuanian University Of Health Sciences); Justas Kuliavas, Audrius Dulskas, Narimantas E. Samalavicius (Klaipeda University Hospital, National Cancer Institute); Kristijonas Jasaitis, Audrius Parseliunas, Viktorija Nevieraite, Margarita Montrimaite, Evelina Slapelyte, Edvinas Dainius, Romualdas Riauka, Zilvinas Dambrauskas, Andrejus Subocius, Linas Venclauskas, Antanas Gulbinas, Saulius Bradulskis, Simona Kasputyte, Deimante Mikuckyte, Mindaugas Kiudelis, Justas Zilinskas, Tomas Jankus, Steponas Petrikenas (Lithuanian University of Health Sciences); Matas Pažuskis, Zigmantas Urniežius, Mantas Vilčinskas (Republican Hospital of Kaunas); Vincas Jonas Banaitis, Vytautas Gaižauskas, Edvard Grisin, Povilas Mazrimas, Rokas Rackauskas, Mantas Drungilas, Karolis Lagunavicius, Vytautas Lipnickas, Dovilè Majauskyté, Valdemaras Jotautas, Tomas Abaliksta, Laimonas Uščinas, Gintaras Simutis, Adomas Ladukas, Donatas Danys, Erikas Laugzemys, Saulius Mikalauskas, Tomas Poškus, Elena Zdanyte Sruogiene, (Vilnius University Hospital); Petras Višinskas, Reda Žilinskienė, Deividas Dragatas (Hospital Of Jonava); Andrius Burmistrovas, Zygimantas Tverskis (Taurage Hospital); Arturas Vaicius, Ruta Mazelyte, Antanas Zadoroznas (Viesoji Istaiga Rokiskio Rajono Ligonine); Nerijus Kaselis, Greta Žiubrytė, (Republican Hospital Of Klaipeda); **Madagascar:** Finaritra Casimir Fleur Prudence Rahantasoa, Luc Hervé Samison, Fanjandrainy Rasoaherinomenjanahary, Todisoa Emmanuella Christina Tolotra (Joseph Ravoahangy Andrianavalona Hospital); **Malawi:** Cornelius Mukuzunga, Vanessa Msosa, Chimwemwe Kwatiwani, Nelson Msiska (Kamuzu Central Hospital); **Malaysia:** Feng Yih Chai, Siti Mohd Desa Asilah, Khuzaimah Zahid Syibrah (Hospital Keningau); Pui Xin Chin, Afizah Salleh, Nur Zulaika Riswan (Kajang Hospital); April Camilla Roslani, Hoong-Yin Chong, Nora Abdul Aziz, Keat-Seong Poh, Chu-Ann Chai, Sandip Kumar (University Malaya Medical Centre); Mustafa Mohammed Taher, Nik Ritza Kosai, Dayang Nita Abdul Aziz, Reynu Rajan (Universiti Kebangsaan Malaysia Medical Centre UKMMC); Rokayah Julaihi, Durvesh Lacthman Jethwani, Muhammad Taqiyuddin Yahaya, Nik Azim Nik Abdullah, Susan Wndy Mathew, Kuet Jun Chung, Milaksh Kumar Nirumal, R. Goh Ern Tze, Syed Abdul Wahhab Eusoffee Wan Ali (Sarawak General Hospital); Yiing Yee Gan, Jesse Ron Swire Ting (Hospital Sibu); Samuel S. Y. Sii, Kean Leong Koay, Yi Koon Tan, Alvin Ee Zhiun Cheah, Chui Yee Wong, Tuan Nur'Azmah Tuan Mat, Crystal Yern Nee Chow, Prisca A.L. Har, Yishan Der (Hospital Sultanah Aminah); Yong Yong Tew, Fitjerald Henry, Xinwei Low (Selayang Hospital); Ya Theng Neo, Hian Ee Heng, Shu Ning Kong, Cheewei Gan, Yi Ting Mok, Yee Wen Tan, Kandasami Palayan, Mahadevan Deva Tata, Yih Jeng Cheong (Hospital Tuanku Ja'afar); Kuhaendran Gunaseelan, Wan Nurul 'Ain Wan Mohd Nasir, Pigeneswaren Yoganathan, (Hospital Keningau); Eu Xian Lee, Jian Er Saw, Li Jing Yeang, Pei Ying Koh, Shyang Yee Lim, Shuang Yi Teo (Hospital Pulau Pinang / Penang Medical College); **Malta:** Nicole Grech, Daniela Magri, Kristina Cassar, Christine Mizzi, Malcolm Falzon, Nihaal Shaikh, Ruth Scicluna, Stefan Zammit, Elaine Borg, Sean Mizzi, Svetlana Doris Brincat, Thelma Tembo, Vu Thanh Hien Le, Tara Grima, Keith Sammut, Kurt Carabott, Alexia Farrugia, Ciskje Zarb, Andre Navarro, Thea Dimech, Georgette Marie Camilleri, Isaac Bertuello, Jeffrey Dalli, Karl Bonavia (Mater Dei Hospital); **Mexico**: Samantha Corro-Diaz, Marisol Manriquez-Reyes, Antonio Ramos-De la Medina (Hospital Español de Veracruz); **Morocco**: Amina Abdelhamid, Abdelmalek Hrora, Sarah Benammi, Houda Bachri, Meryem Abbouch, Khaoula Boukhal, Redouane Mammar Bennai, Abdelkader Belkouchi, Mohamed Sobhi Jabal, Chaymae Benyaiche (IBN Sina Hospital); **Netherlands:** Maarten Vermaas, Lucia Duinhouwer, (Ijsselland Hospital); **Nicaragua:** Javier Pastora, Greta Wood, Maria Soledad Merlo (Hospital Escuela Oscar Danilo Rosales Arguello); **Nigeria:** Akinlabi Ajao, Omobolaji Ayandipo, Taiwo Lawal, Abdussemiu Abdurrazzaaq, (University College Hospital, Ibadan); Muslimat Alada, Abdulrasheed Nasir, James Adeniran, Olufemi Habeeb, Ademola Popoola, Ademola Adeyeye (University Of Ilorin Teaching Hospital,Ilorin); Ademola Adebanjo, Opeoluwa Adesanya, Adewale Adeniyi (Federal Medical Centre, Abeokuta,); Henry Mendel, Bashir Bello, Umar Muktar (Usmanu Danfodiyo University Teaching Hospital);); Adedapo Osinowo, Thomas Olagboyega Olajide, Oyindamola Oshati, George Ihediwa, Babajide Adenekan, Victor Nwinee, Felix Alakaloko, Adesoji Ademuyiwa, Olumide Elebute, Abdulrazzaq Lawal, Chris Bode, Mojolaoluwa Olugbemi (Lagos University Teaching Hospital); Alaba Adesina, Olubukola Faturoti, Oluwatomi Odutola, Oluwaseyi Adebola, Clement Onuoha, Ogechukwu Taiwo (Babcock University Teaching Hospital); Omolara Williams, Fatai Balogun, Olalekan Ajai, Mobolaji Oludara, Iloba Njokanma, Roland Osuoji (Lagos State University Teaching Hospital); Stephen Kache, Jonathan Ajah, Jerry Makama (Barau Dikko Teaching Hospital, Kaduna State University, Kaduna); Ahmed Adamu, Suleiman Baba, Mohammad Aliyu, Shamsudeen Aliyu, Yahaya Ukwenya, Halima Aliyu, Tunde Sholadoye, Muhammad Daniyan, Oluseyi Ogunsua (Ahmadu Bello University Teaching Hospital Zaria); Lofty-John Anyanwu, Abdurrahaman Sheshe, Aminu Mohammad (Aminu Kano Teaching Hospital); Samson Olori, Philip Mshelbwala, Babatunde Odeyemi, Garba Samson, Oyediran Kehinde Timothy, Sani Ali Samuel (University Of Abuja Teaching Hospital,); Anthony Ajiboye, Ademola Adeyeye, Isaac Amole, Olajide Abiola, Akin Olaolorun (Bowen University Teaching Hospital); **Norway:** Kjetil Søreide, Torhild Veen, Arezo Kanani, Kristian Styles, Ragnar Herikstad, Johannes Wiik Larsen, Jon Arne Søreide (Stavanger University Hospital); Elisabeth Jensen, Mads Gran, Eirik Kjus Aahlin (University Hospital Of Northern Norway); Tina Gaarder, Peter Wiel Monrad-Hansen, Pål Aksel Næss (Oslo University Hospital); Giedrius Lauzikas, Joachim Wiborg, Silje Holte (Sykehuset Telemark HF); Knut Magne Augestad, Gurpreet Singh Banipal, Michela Monteleone, Thomas Tetens Moe, Johannes Kurt Schultz (Akershus University Hospital); **Palestine:** Taher Al-taher, Ayah Hamdan, Ayman Salman, Rana Saadeh, Aseel Musleh, Dana Jaradat, Soha Abushamleh, Sakhaa Hanoun, Amjad Abu Qumbos, Aseel Hamarshi, Ayman And Taher (Al Makassed Islamic Charitable Society Hospital, Jerusalem); Israa Qawasmi, Khalid Qurie, Marwa Altarayra, Mohammad Ghannam, Alaa Shaheen, Azher Herebat (Alia Governmental Hospital); Aram Abdelhaq, Ahmad Shalabi, Maram Abu-toyour, Fatema Asi, Ala Shamasneh, Anwar Atiyeh, Mousa Mustafa, Rula Zaa'treh, Majd Dabboor (Palestine Medical Complex); Enas Alaloul, Heba Baraka, Jehad Meqbil, Alaa Al-Buhaisi, Mohamedraed Elshami, Samah Afana, Sahar Jaber, Said Alyacoubi, Yousef Abuowda (Islamic University of Gaza Medical School, European Gaza Hospital & Shifa Hospital); Tasneem Idress, Eman Abuqwaider (Mizan hospital); Sara Al-saqqa, Alaa Bowabsak, Alaa El Jamassi, Doaa Hasanain, Hadeel Al-farram, Maram Salah, Aya Firwana, Marwa Hamdan, Israa Awad (Al-Shifa Hospital); Ahmad Ashour, Fayez Elian Al Barrawi (Bit Hanoun Hospital); Ahmed Al-khatib, Maha Al-faqawi, Mohamed Fares (Nasser Hospital); Amjad Elmashala, Mohammad Adawi, Ihdaa Adawi (Beit Jala Governmental Hospital); Reem Khreishi, Rose Khreishi (Martyr Thabet Govermental hospital, Tulkarem); Ahmad ashour, Ahed Ghaben (Indonesian Hospital, Gaza); **Pakistan:** Najwa Nadeem, Muhammad Saqlain (Allied Hospital, Faisalabad); Jibran Abbasy, Abdul Rehman Alvi, Tanzeela Gala, Noman Shahzad (Aga Khan University); Kamran Faisal Bhopal, Zainab Iftikhar, Muhammad Talha Butt, Syed Asaat ul Razi, Asdaq Ahmed, Ali Khan Niazi (Bahawal Victoria Hospital); Ibrahim Raza, Fatima Baluch, Ahmed Raza, Ahmad Bani-Sadar, Ahmad Uzair Qureshi, Muhammad Adil, Awais Raza, (King Edward Medical University, Mayo Hospital, Lahore); Mahnoor Javaid, Muhammad Waqar, Maryam Ali Khan (CMH Lahore Medical And Dental College); Mohammad Mohsin Arshad, Mohammadasim Amjad (Nishtar Medical College And Hospital); **Paraguay:** Gustavo Miguel Machain Vega, Jorge Torres Cardozo, Marcelo O´Higgins Roche, Gustavo Rodolfo Pertersen Servin, Helmut Alfredo Segovia Lohse, Larissa Ines Páez Lopez, Ramón Augusto Melo Cardozo (Hospital de Clínicas, II Cátedra de Clínica Quirúrgica, Universidad Nacional de Asunción); **Peru:** Fernando Espinoza, Angel David Pérez Rojas, Diana Sanchez, Camila Sanchez Samaniego, Shalon Guevara Torres, Alexander Canta Calua, Cesar Razuri, Nadia Ortiz, Xianelle Rodriguez, Nahilia Carrasco, Fridiz Saravia, Hector Shibao Miyasato, María Valcarcel-Saldaña, Ysabel Esthefany Alejos Bermúdez, Juan Carpio, Walter Ruiz Panez, Pedro Angel Toribio Orbegozo, (Hospital Nacional Arzopispo Loayza); Carolina Guzmán Dueñas, Kevin Turpo Espinoza, Ana Maria Sandoval Barrantes, Jorge Armando Chungui Bravo, Sebastian Shu, Lorena Fuentes-Rivera, Carmen Fernández, Diego Romani, Bárbara Málaga, Joselyn Ye (Hospital Cayetano Heredia); Ricardo Velasquez, Jannin Salcedo (Clínica De Especialidades Médicas); Ana Lucia Contreras-Vergara, Angelica Genoveva Vergara Mejia, Maria Soledad Gonzales Montejo (Hospital Nacional Guillermo Almenara Irigoyen); Marilia Del Carmen Escalante Salas, Willy Alcca Ticona, Marvin Vargas, George Christian Manrique Sila, Robinson Mas, Arazzelly del Pilar Paucar (Hospital Regional De Ayacucho); Armando José Román Velásquez, Alina Robledo-Rabanal, Ludwing Alexander Zeta Solis, (Hospital III José Cayetano Heredia); Kenny Turpo Espinoza (Hospital Nacional Maria Auxiliadora); José Luis Hamasaki Hamaguchi, Erick Samuel Florez Farfan, Linda Alvi Madrid Barrientos, Juan Jaime Herrera Matta (Hospital De Policia); **Philippines:** John Jemuel V. Mora, Menold Archee P. Redota, Manuel Francisco Roxas, Maria Jesusa B. Maño, (The Medical City); Marie Dione Parreno-Sacdalan, Marie Carmela Lapitan, Christel Leanne Almanon (Department Of Surgery, Philippine General Hospital, University Of The Philippines Manila); **Poland:** Maciej Walędziak, Rafał Roszkowski, Michał Janik (Department Of General, Oncological, Metabolic And Thoracic Surgery, Military Institute Of Medicine, Warsaw); Anna Lasek, Piotr Major, Dorota Radkowiak, Mateusz Rubinkiewicz (2nd Department Of Surgery, Jagiellonian University Medical College); **Portugal:** Cristina Fernandes, Jose Costa-Maia, Renato Melo (Centro Hospitalar De São João); **Romania:** Liviu Muntean, Aurel Sandu Mironescu, Lucian Corneliu Vida (Spitalul Clinic De Copii Brasov); Amar Kourdouli, Mariuca Popa (Spital Judetean De Urgenta Din Craiova); Hogea Mircea (Spitalul Clinic Judetean Brasov); Mihaela Vartic, Bogdan Diaconescu, Matei Razvan Bratu, Ionut Negoi, Mircea Beuran, Cezar Ciubotaru (Emergency Hospital of Bucharest); **Rwanda:** J.C Allen Ingabire, Alphonse Zeta Mutabazi, Norbert Uzabumwana (University Teaching Hospital Of Kigali); Dieudonne Duhoranenayo (Kibungo Hospital); **San Marino:** Elio Jovine, Nicola Zanini, Giovanni Landolfo (San Marino State Hospital); **Saudi Arabia:** Murad Aljiffry, Faisal Idris, Mohammed Saleh A. Alghamdi, Ashraf Maghrabi, Abdulmalik Altaf, Aroub Alkaaki, Ahmad Khoja, Abrar Nawawi, Sondos Turkustani, (Department of Surgery, Faculty of Medicine, King Abdulaziz University Hospital, Jeddah) Eyad Khalifah, Ahmad Gudal, Adel Albiety, Sarah Sahel, Reham Alshareef, Mohammed Najjar (Department of Surgery, King Abdulaziz University Hospital and Oncology Center, Jeddah) Ahmed Alzahrani, Ahmed Alghamdi, Wedyan Alhazmi, (King Fahad Hospital, Jeddah) Ghiath Al Saied, Mohammed Alamoudi, Muhammed Masood Riaz (King Fahad Medical City, Riyadh); Mazen Hassanain, Basmah Alhassan, Abdullah Altamimi, Reem Alyahya, Norah Al Subaie, Fatema Al Bastawis, Afnan Altamimi, Thamer Nouh, Roaa Khan, (King Khaled University Hospital); **Serbia:** Milan Radojkovic, Ljiljana Jeremic, Milica Nestorovic (Clinic For General Surgery, Clinical Center Nis); **Singapore:** Jia Hao Law, Keith Say Kwang Tan, Ryan Choon Kiat Tan, Joel Kin Tan, Lau Wen Liang Joel, Bettina Lieske, Xue Wei Chan, Faith Qi Hui Leong, Choon Seng Chong, Sharon Koh, Kai Yin Lee, Kuok Chung Lee (National University Hospital); **South Africa:** Kent Pluke, Britta Dedekind, Puyearashid Nashidengo, Mark Ian Hampton (Victoria Hospital Wynberg); Johanna Joosten, Sanju Sobnach, Liana Roodt, Anthony Sander, James Pape, Richard Spence (Groote Schuur); Niveshni Maistry (Charlotte Maxeke Johannesburg Academic Hospital); Phumudzo Ndwambi, Kamau Kinandu, Myint Tun (Leratong Hospital); Frederick Du Toit, Quinn Ellison, Sule Burger, DC Grobler, Lawrence Bongani Khulu (Tembisa Tertiary Provincial Hospital); Rachel Moore, Vicky Jennings, Astrid Leusink (Chris Hani Baragwanath Academic Hospital); Nazmie Kariem, Juan Gouws, Kathryn Chu, Heather Bougard, Fazlin Noor, Angela Dell (New Somerset Hospital); Sarah Rayne, Stephanie Van Straten, (Helen Joseph Hospital, University Of Witwatersrand); Arvin Khamajeet, Serge Kapenda Tshisola, Kalangu Kabongo (Stanger Hospital); Victor Kong (Edendale Hospital); Yoshan Moodley, Frank Anderson, Thandinkosi Madiba (Inkosi Albert Luthuli Central Hospital); Flip du Plooy (Mediclinic Potchefstroom); Leila Hartford, Gareth Chilton, Parveen Karjiker (Mitchell's Plain District Hospital); Matlou Ernest Mabitsela, Sibongile Ruth Ndlovu (Dr George Mukhari Academic Hospital); Maria Badicel, Robert Jaich (Milpark Hospital); **Spain:** Jaime Ruiz-Tovar (University Hospital Rey Juan Carlos); Luis Garcia-Florez, Jorge L. Otero-Díez, Virginia Ramos Pérez, Nuria Aguado Suárez (Hospital Universitario San Agustín); Javier Minguez García, Sara Corral Moreno, Maria Vicenta Collado , Virginia Jiménez Carneros, Javier García Septiem (Hospital Universitario de Getafe); Mariana Gonzalez, Antonio Picardo, Enrique Esteban, Esther Ferrero, Irene Ortega, (Infanta Sofía University Hospital); Eloy Espin-Basany, Ruth Blanco-Colino, Valeria Andriola (Hospital Valle De Hebron); Lorena Solar García, Elisa Contreras, Carmen García Bernardo, Janet Pagnozzi, Sandra Sanz, Alberto Miyar de León, Asnel Dorismé, Joseluis Rodicio, Aida Suarez, Jessica Stuva, Tamara Diaz Vico (Central University Hospital Of Asturias); Laura Fernandez-Vega, Carla Soldevila-Verdeguer, Fatima Sena-Ruiz, Natalia Pujol-Cano, Paula Diaz-Jover, José Maria Garcia-Perez, Juan Jose Segura-Sampedro, Cristina Pineño-Flores, David Ambrona-Zafra, Andrea Craus-Miguel, Patricia Jimenez-Morillas, Angela Mazzella (Hospital Universitario Son Espases); **Sri Lanka**: A.B Jayathilake, S.P.B Thalgaspitiya, L.S. Wijayarathna, P.M.S.N. Wimalge (University Surgical Unit, Teaching Hospital Anuradhapura); **St. Kitts And Nevis:** Hakeem Ayomi Sanni, Aliyu Ndajiwo, Ogheneochuko Okenabirhie (Joseph N France Hospital); **Sudan:** Anmar Homeida, Abobaker Younis, Omer Abdelbagi Omer, Mustafa Abdulaziz, Ali Mussad, Ali Adam (University of Gezira); **Sweden:** Yucel Cengiz, Ida Björklund, Sandra Ahlqvist, Sandra Ahlqvist, (Sundsvall Hospital); Anders Thorell, Fredrik Wogensen (Ersta Hospital); Arestis Sokratous, Michaela Breistrand (Mora Hospital); Hildur Thorarinsdottir (Helsingborgs Lasarett); Johanna Sigurdadottir, Maziar Nikberg, Abbas Chabok (Västmanlands Hospital Västerås); Maria Hjertberg (Department Of Surgery And Department Of Clinical And Experimental Medicine, Linköping University, Norrköping, Sweden); Peter Elbe, Deborah Saraste, Wiktor Rutkowski, Louise Forlin (Karolinska Universitetssjukhuset, Solna); Karoliina Niska, Malin Sund (Umea University Hospital); **Switzerland:** Dennis Oswald, Georgios Peros, Rafael Bluelle, Katharina Reinisch, Daniel Frey, Adrian Palma (Gzo Spital Wetzikon); Dimitri Aristotle Raptis, Lucius Zumbühl, Markus Zuber (Kantonsspital Olten); Roger Schmid, Gabriela Werder (Buergerspital Solothurn); Antonio Nocito, Alexandra Gerosa, Silke Mahanty (Kantonsspital Baden); Lukas Werner Widmer, Julia Müller, Alissa Gübeli (Hospital Davos); Grzegorz Zuk (Gzo Spital Wetzikon); **Turkey:** Osman Bilgin Gulcicek, Yuksel Altinel, Talar Vartanoglu, (Bagcilar Research And Training Hospital); Emin Kose, Servet Rustu Karahan, Mehmet Can Aydin (Okmeydanı Training And Research Hospital); Nuri Alper Sahbaz, Ilkay Halicioglu, Halil Alis (Bakirkoy Dr. Sadi Konuk Training And Research Hospital); Ipek Sapci, Can Adıyaman, Ahmet Murat Pektaş, Turgut Bora Cengiz, Ilkan Tansoker, Vedatcan Işler, Muazzez Cevik, Deniz Mutlu, Volkan Ozben, Berk Baris Ozmen, Sefa Bayram, Sinem Yolcu, Berna Buse Kobal, Ömer Faruk Toto, Haluk Cem Çakaloğlu (Acibadem University School of Medicine, Atakent Hospital); Kagan Karabulut, Vahit Mutlu, Bahar Busra Ozkan (Ondokuz Mayis University Medical Faculty); Saban Celik, Anil Semiz, Selim Bodur, Enisburak Gül, Busra Murutoglu, Reyyan Yildirim, Bahadir Emre Baki, Ekin Arslan, Ali Guner, Kadir Tomas (Karadeniz Technical University Faculty Of Medicine); **United Kingdom:** Nathan Walker, Nikhita Shrimanker, Michael Stoddart, Simon Cole (Royal United Hospital Bath); Ryan Breslin, Ravi Srinivasan (Blackpool Victoria Hospital); Mohamed Elshaer, Kristina Hunter, Ahmed Al-Bahrani (Watford General Hospital); Ignatius Liew, Nora Grace Mairs, Alistair Rocke, Lachlan Dick, Mobeen Qureshi (Inverclyde Royal Hospital); Debkumar Chowdhury (University Hospital Ayr); Naomi Wright, Clare Skerritt, Dorothy Kufeji (Guy's And St. Thomas' Hospitals); Adrienne Ho, Tharindra Dissanayake, Athula Tennakoon, Wadah Ali, (Pilgrim Hospital, United Lincolnshire Hospitals NHS Trust); Shujing Jane Lim, Charlene Tan, Stephen O'Neill, Catrin Jones (Victoria Hospital Kirkcaldy); Stephen Knight, Dima Nassif, Abhishek Sharma (Perth Royal Infirmary); Oliver Warren, Rebecca White, Aia Mehdi, Nathan Post, Eliana Kalakouti, Enkhbat Dashnyam, Frederick Stourton (Chelsea And Westminster Hospital); Ioannis Mykoniatis, Chelise Currow, (Northampton General Hospital); Francisca Wong, Ashish Gupta, Veeranna Shatkar (Queen's Hospital, BHR University Hospitals NHS Trust); Joshua Luck, Suraj Kadiwar, Alexander Smedley (North Middlesex University Hospital); Rebecca Wakefield, Philip Herrod, James Blackwell, Jonathan Lund, (Royal Derby Hospital); Fraser Cohen, Ashwath Bandi, Stefano Giuliani (St George's Hospital); Giles Bond-Smith, Theodore Pezas, Neda Farhangmehr, Tomas Urbonas, Miklos Perenyei (John Radcliffe Hospital, Oxford); Philip Ireland, Natalie Blencowe, Kirk Bowling, David Bunting (Gloucestershire Royal Hospital); Lydia Longstaff, Neil Smart, Kenneth Keogh (Royal Devon & Exeter Hospital); Hyunjin Jeon, Muhammad Rafaih Iqbal, Shivun Khosla, Anna Jeffery, James Perera (Maidstone & Tunbridge Wells NHS Trust); Ella Teasdale (Western Isles Hospital); Ahmad Aboelkassem Ibrahem, Tariq Alhammali, Yahya Salama (Kettering General Hospital NHS Trust); Rakan Kabariti, Shaun Oram (Nevill Hall Hospital); Thomas Kidd, Fraser Cullen, Christopher Owen, Michael Wilson, Seehui Chiu, Hannah Sarafilovic, (Ninewells Hospital); Jennifer Ploski, Elizabeth Evans, Athar Abbas, Sylvia Kamya, Norzawani Ishak, Carly Bisset, Cedar Andress, Ye Ru Chin (Royal Alexandra Hospital, Paisley); Priya Patel, David Evans (University Hospital, Wales); Anna Jeffery, James Perera (Maistone and Tunbridge Wells NHS Trust); Aidan Haslegrave, Adam Boggon, Kirsten Laurie, Katie Connor, Thomas Mann (Borders General Hospital); Dmitri Nepogodiev, Anahita Mansuri, Rachel Davies, Ewen Griffiths (University Hospitals Birmingham NHS Trust); Aized Raza Shahbaz, Calvin Eng, Farhat Din, Ariadne L'Heveder, Esther H.G. Park, Ramanish Ravishankar, Kirsten McIntosh, Jih Dar Yau, Luke Chan, Susan McGarvie (Western General Hospital, Edinburgh); Lingshan Tang, Hui Lim, Suhhuey Yap, Jay Park, Zhan Herr Ng, Shahrukh Mirza, Yun Lin Ang, Luke Walls, Ella Teasdale, Chloe Roy, Simon Paterson-Brown, Julian Camilleri-Brennan, Kenneth Mclean, Michelle S D'Souza, Savva Pronin, David Ewart Henshall, Eunice Zuling Ter (Royal Infirmary Of Edinburgh); Dina Fouad, Ashish Minocha (Norfolk And Norwich University Hospital); William English, Catrin Morgan, Dominic Townsend, Laura Maciejec, Shareef Mahdi, Onyinye Akpenyi, Elisabeth Hall, Hanaan Caydiid, Zakaria Rob, Tom Abbott, Hew D Torrance (The Royal London Hospital); Gareth Irwin, Robin Johnston (Ulster Hospital Dundonald); Mohammed Akil Gani, Gianpiero Gravante (Leicester Royal Infirmary); Shivanchan Rajmohan, Kiran Majid, Shiva Dindyal, Christopher Smith (West Middlesex University Hospital); Madanmohan Palliyil, Sanjay Patel, Luke Nicholson, Neil Harvey, Katie Baillie, Sam Shillito, Suzanne Kershaw, Rebecca Bamford, Peter Orton (Stockport NHS Foundation Trust); Elke Reunis, Robert Tyler, Wai Cheong Soon (Good Hope Hospital); Guled M. Jama, Dharminder Dhillon, Khyati Patel (Walsall Manor Hospital, Walsall); Shayanthan Nanthakumaran, Rachel Heard, Kar Yan Chen (Aberdeen Royal Infirmary); Behrad Barmayehvar, Uttaran Datta, Sivesh K Kamarajah, Sharad Karandikar (Heartlands Hospital); Sobhana Iftekhar Tani (Nottingham University Hospital NHS Trust); Eimear Monaghan, Philippa Donnelly, Michael Walker (Raigmore Hospital); Jehangirshaw Parakh, Sarah Blacker, Anil Kaul (Whiston Hospital); Arjun Paramasivan (Darlington Memorial Hospital); Sameh Farag, Ashrafun Nessa, Salwa Awadallah (Worthing Hospital, Western Sussex Hospital NHS Foundation Trust); Jieqi Lim, James Chean Khun Ng (Queen Elizabeth University Hospital, Glasgow); **United States**: Katherine Gash, Ravi P. Kiran, Alice Murray (New York Presbyterian Hospital / Columbia University Medical Center); Eric Etchill, Mohini Dasari, Juan Puyana (University Of Pittsburgh Medical Center - Presbyterian); Nadeem Haddad, Martin Zielinski, Asad Choudhry (Mayo Clinic); Celeste Caliman, Mieshia Beamon, Therese Duane (John Peter Smith Hospital); Ragavan Narayanan, Mamta Swaroop (Northwestern Memorial Hospital / Northwestern University); Jonathan Myers, Rebecca Deal, Erik Schadde (Rush University Medical Centre); Mark Hemmila, Lena Napolitano, Kathleen To (University Of Michigan Medical Center); **Zambia**: Alex Makupe, Joseph Musowoya, Mayaba Maimbo (Ndola Central Hospital); Niels Van Der Naald, Dayson Kumwenda, Alex Reece-Smith, Kars Otten, Anna Verbeek, Marloes Prins (St Francis Mission Hospital)

*GlobalSurg Collaborative data validators:*

**Argentina**: Alibeth Andres Baquero Suarez (Simplemente Evita), Ruben Balmaceda (Hospital Lagomaggiore); **Barbados**: Chelsea Deane (Queen Elizabeth Hospital); **Croatia**: Emilio Dijan (Zadar General Hospital); **Egypt**: Mahmoud Elfiky (Kasr Al Ainy Faculty of Medicine, Cairo University); **Finland**: Laura Koskenvuo (Helsinki University Hospital); **France**: Aurore Thollot (CHU Poitiers), Bernard Limoges (CHU Limoges), Carmen Capito (Hopital Necker Enfants Malades, APHP), Challine Alexandre (Hopital Cochin, APHP), Henri Kotobi (Trousseau Hospital, APHP), Julien Leroux (CHU Rouen), Julien Rod (CHU Caen), Kalitha Pinnagoda (CHU Toulouse), Nicolas Henric (CHU Angers), Olivier Azzis (CHU Rennes), Olivier Rosello (CHU Nice), Poddevin Francois (GHICL), Sara Etienne (CHU Saint Etienne); Philippe Buisson (CHU Amiens Picardie), Sophian Hmila (Hopital Robert Ballanger, Paris); **Ghana**: Joe-Nat Clegg-Lamptey (Korle Bu Teaching Hospital), Osman Imoro (Baptist Medical Centre), Owusu Emmanuel Abem (Komfo Anokye Teaching Hospital), Paul Wondoh (Upper West Regional Hospital); **Greece**: Dimitrios Papageorgiou (Naval And Veterans Hospital Of Athens), Vasiliki Soulou (Anticancer Hospital Of Athens Agios Savvas); **Guatemala**: Sabrina Asturias (Hospital Herrera Llerandi Amedesgua), Lenin Peña (Hospital General San Juan De Dios); **India**: Basant Kumar (Sanjay Gandhi Post Graduate Institute Of Medical College Lucknow); **Ireland**: Donal B O’Connor (Tallaght Hospital, Trinity College Dublin); **Italy**: Alberto Realis Luc (Santa Rita Clinic, Vercelli), Alfio Alessandro Russo (Treviglio Hospital), Andrea Ruzzenente (Azienda Ospedaliera Universitaria Integrata di Verona), Antonio Taddei (Azienda Ospedaliera Universitaria Careggi), Camilla Cona (IOV - Istituto Oncologico Veneto), Corrado Bottini (Sant'Antonio Abate Hospital, Gallarate), Giovanni Pascale (Azienda Ospedaliero-Universitaria di Ferrara), Giuseppe Rotunno (Nicola Giannettasio Hospital, Rossano), Leonardo Solaini (University Of Brescia, Spedali Civili Di Brescia ), Marco Maria Pascale (Fondazione Policlinico Universitario 'Agostino Gemelli' ), Margherita Notarnicola (University Of Bari 'Aldo Moro'), Mario Corbellino (Ospedale Luigi Sacco Milano), Michele Sacco (Federico II University of Naples), Paolo Ubiali (Azienda per L'Assistenza Sanitaria N. 5 'Friuli Occidentale', Pordenone), Roberto Cautiero (Second University Of Naples), Tommaso Bocchetti, (Sant’Andrea Hospital, Sapienza University of Rome), Elena Muzio, (S. Andrea Hospital, Poll-Asl 5, La Spezia); Vania Guglielmo (Policlinico Umberto I, Emergency Surgery Department); Eugenio Morandi (Ospedale di Rho – ASST Rhodense), Patrizio Mao (San Luigi Gonzaga Hospital, Orbassano); Emilia De Luca (Department of Medical and Surgical Sciences, Policlinico Universitario Mater Domini Campus Salvatore Venuta, Catanzaro), Margherita Notarnicola (Azienda Ospedaliero Universitaria Consorziale Policlinico Di Bari). **Jordan**: Farah Mahmoud Ali (Jordan University Hospital); **Lithuania**: Justas Žilinskas (Klaipeda Republic), Kestutis Strupas (Vilnius University Hospital), Paulius Kondrotas (Taurage County Hospital), Robertas Baltrunas (Rokiskis District Municipality Hospital); Juozas Kutkevicius (Department Of General Surgery, Lithuanian University Of Health Sciences), Povilas Ignatavicius (Hospital Of Lithuanian University Of Health Sciences Kaunas Clinics); **Malaysia**: Choy Ling Tan (Hospital Sultanah Aminah), Jia Yng Siaw (Hospital Sibu), Sir Young Yam (Penang Medical College); Ling Wilson (Sarawak General Hospital), Mohamed Rezal Abdul Aziz (University Malaya Medical Centre); **Malta**: John Bondin (Mater Dei Hospital); **Mexico**: Carmina Diaz Zorrilla (Hospital Espanol De Veracruz); **Morocco**: Anass Majbar (Centre Hospitalier Ibn Sina Rabat); **Nigeria**: Danjuma Sale (Barau Dikko Teaching Hospital), Lawal Abdullahi (Kano Aminu), Olabisi Osagie (University Of Abuja Teaching Hospital), Omolara Faboya (Lagos Lasuth); Adedeji Fatuga (Lagos Luth), Agboola Taiwo (Babcock University Teaching Hospital), Emeka Nwabuoku (Ahmadu Bello University Teaching Hospital); **Norway**: Marte Bliksøen (Oslo University Hospital); **Pakistan**: Zain Ali Khan (Bahawal Victoria Hospital, Bahawalpur); **Paraguay**: Jazmin Coronel (Hospital de Clínicas, II Cátedra de Clínica Quirúrgica, Universidad Nacional de Asunción); **Peru**: Cesar Miranda (Hospital Nacional Cayetano Heredia), Idelso Vasquez (Lima Almenara), Luis M. Helguero-Santin (Hospital Regional III Jose Cayetano Heredia – Piura); **Rwanda**: Jennifer Rickard (Centre Hospitalier Universitaire De Kigali); **Romania**: Aurel Mironescu (Spitalul Clinic De Copii Brasov); **Saint Kitts and Nevis**: Adesina Adedeji (Joseph N France Hospital); **Saudi Arabia**: Saleh Alqahtani (King Fahad General Hospital); **South Africa**: Max Rath (Groote Schuur Hospital), Michael Van Niekerk (New Somerset Hospital), Modise Zacharia Koto (Dr George Mukhari Academic Hospital); Roel Matos-Puig (Stanger Hospital); **Sweden**: Leif Israelsson (Sundsvall); **Switzerland**: Tobias Schuetz (Kantonsspital Olten); **Turkey**: Mahmut Arif Yuksek (Ondokuz Mayis University), Meric Mericliler (Acibadem University School of Medicine, Atakent Hospital), Mehmet Uluşahin (Karadeniz Technical University Farabi Hospital); **United Kingdom**: Bernhard Wolf (Raigmore Hospital Inverness), Cameron Fairfield (Royal Infirmary Of Edinburgh), Guo Liang Yong (Perth Royal Infirmary), Katharine Whitehurst (Royal Devon And Exeter), Michael Wilson (Ninewells Hospital And Medical School), Natalie Redgrave (John Radcliffe Hospital, Oxford); Caroluce K Musyoka (Royal Alexandra Hospital), James Olivier (Royal United Hospital Bath), Kathryn Lee (Queen Elizabeth Birmingham), Michael Cox (Royal Derby Hospital), Muhamed M H Farhan-Alanie (Inverclyde Royal Hospital), Rory Callan (North Middlesex University Hospital); **Zambia**: Chali Chibuye (Ndola Central Hospital)

*GlobalSurg Collaborative protocol translators:*

**Arabic,** Tebian Hassanein Ahmed Ali, Syrine Rekhis, Muna Rommaneh, Oday Halhouli**; Chinese,** Zi Hao Sam**; French,** Lawani Ismaïl**; Greek,** Vasileios Kalles**; Italian,** Francesco Pata, Gabriela Elisa Nita, Federico Coccolini, Luca Ansaloni**; Portuguese,** Thays Brunelli Pugliesi, Gabriel Pardo**; Spanish,** Ruth Blanco

## Detailed description of the method:

We determine three types of economic burden: consequences of not providing the local standard of care to all appendicitis patients, consequences of not providing the standard of care of high-income countries to all appendicitis patients, and the consequences of providing surgical care with local standard to the people who currently receive care and not providing surgical care to the people who currently receive no care.

To calculate these three types, we first estimated the excess occurrence of the adverse events of mortality and absenteeism per appendicitis case in a given country. Then, we multiplied these adverse events with the country’s incidence and annual wage.

### Occurrence of adverse events

To assess the occurrence of adverse events, we first estimated the extent of unmet need for surgical care in country i. We assumed that each case of appendicitis has a probability $p_{i}^{op}$ to be operated, and a probability $p_{i}^{noop}=1-p_{i}^{op}$ to forgo an operation (see Figure S 1). Assuming that every case of appendicitis should undergo a surgical procedure, we refer to the probability $p_{i}^{noop}$ as the *share of unmet surgical need for appendicitis*. We assumed that this share is zero in high-income countries (HICs)^[[1]](#footnote-1)^, but varies across low- and middle-income countries (LMICs) where access to care is limited^7^.

*Figure S 1 Probability tree 1: Probability of operation*


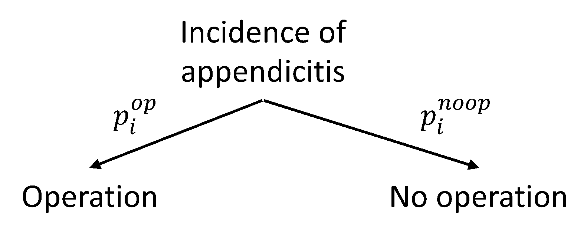


We estimated this share of unmet surgical need for LMICs using two approaches (see appendix Table S 2 for details on the data sources for both approaches):

1. The proportion of unmet need approach. Following the Lancet Commission on Global Surgery (LCoGS), we assumed the total surgical volume to meet need was 5,000 operations per 100,000 people^1^. The difference between the country’s actual surgical volume (obtained from Holmer et al (2019)^10^) and LCoGS estimate of volume to meet need was used to estimate the proportion of unmet need among the cases of appendicitis. Given that in many LMICs emergency surgeries^11^ (for example, appendectomies) form a larger proportion of total volume than in HICs, we interpret this derived proportion of unmet need as an upper estimate for the gap of unmet need.
2. The share of surgical volume approach. We assumed that appendectomies make up a certain share of the total surgical volume done (estimated from Holmer et al (2019)^10^). To estimate this share, we used information on the proportion of appendectomies to gastrointestinal surgery and information on the proportion of gastrointestinal surgery to total surgical volume from the literature and datasets as described in appendix section S.3. From this, we predicted the number of appendectomies for each country. The difference between a country’s incidence of appendicitis and the predicted number of appendectomies was used to estimate the proportion of unmet need among the cases of appendicitis.

Next, we assessed the consequences of met surgical need (operation) and unmet surgical need (no operation), as depicted in Figure S 2. We divided the consequences of an operation into three categories: successful operation, minor adverse events, and reoperation. Similarly, we subdivide the consequences of no operation into mild cases, moderate cases, and severe cases. Each consequence occurs with a specific probability $p$, and entails a certain outcome $\omega$ in the form of mortality risk (as % of appendectomies done) and absenteeism (as number of working days lost) as depicted in Table S 1. Sources of the estimates can be found in Table S 5 and Table S 6 in appendix S.3. The literature and available data do not allow us to disaggregate probabilities of scenarios in case of operation (e.g., of minor adverse events) or no operation (e.g., severe cases) or the associated outcomes $\omega$ coherently by country, age group, or sex^[[2]](#footnote-2)^. Hence, we assume them to be constant along these dimensions and to vary only between country income groups. In addition, we assume that the probabilities and outcomes are independent of the share of unmet need. This implicitly assumes that changes in the share of unmet need have no impact on the quality of care.

Figure S 2 Probability tree 2: Scenarios and outcomes of (no) operation


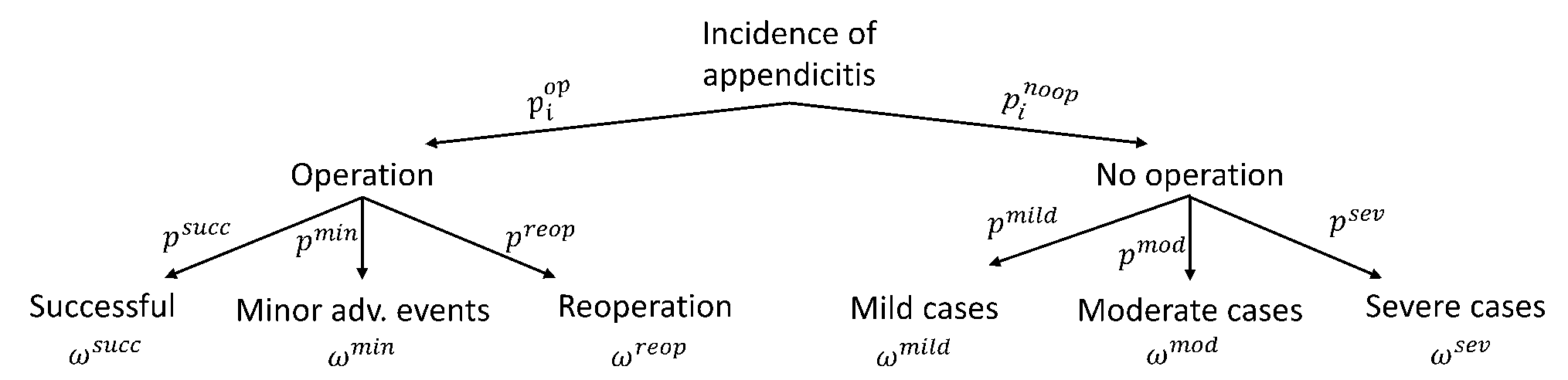


Table S 1 Scenario probabilities and respective outcomes across scenarios and income groups

|  |  | **Successful operation** | **Minor adv. events** | **Reoperation** | **Mild cases** | **Moderate cases** | **Severe cases** |
| --- | --- | --- | --- | --- | --- | --- | --- |
| **Probability of scenario** | LMIC | 82% | 15.5% | 2.5% | 10% | 68.5% | 21.5% |
|  | HIC | 87% | 10.5% | 2.5% | NA | NA | NA |
| **Outcome: Mortality risk** | LMIC | 0% | 0.6% | 0.6% | 0% | 10% | 19% |
|  | HIC | 0% | 0.1% | 0.1% | NA | NA | NA |
| **Outcome: Absen-teeism (days lost)** | LMIC | 7 | 24 | 60 | 4 | 15 | 60 |
|  | HIC | 4 | 15 | 60 | NA | NA | NA |

Based on this, we estimated the rate of excess adverse events due to not providing surgery with the local standard in LMICs, and not providing surgical care with the standard available in HICs as displayed in Figure S 3.

Figure S 3 Graphic display of the calculation of adverse events with the local or the highest standard


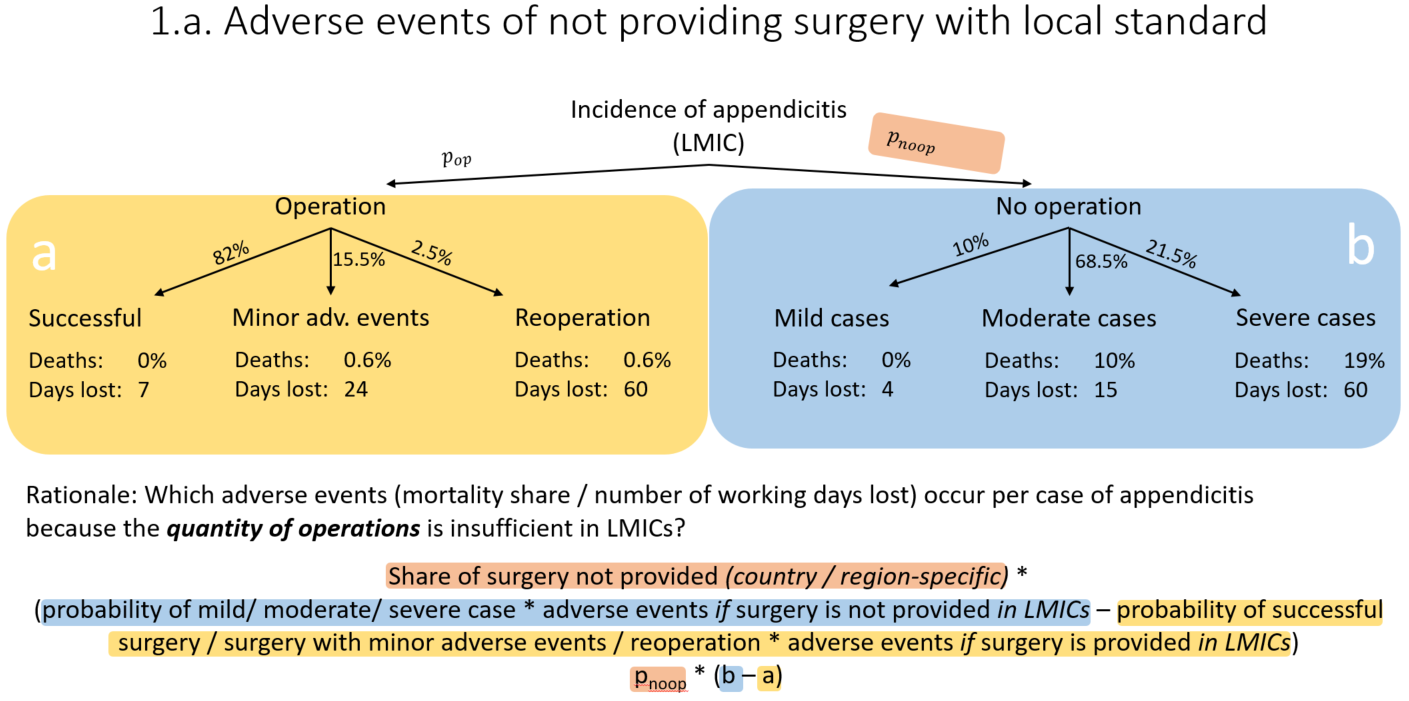

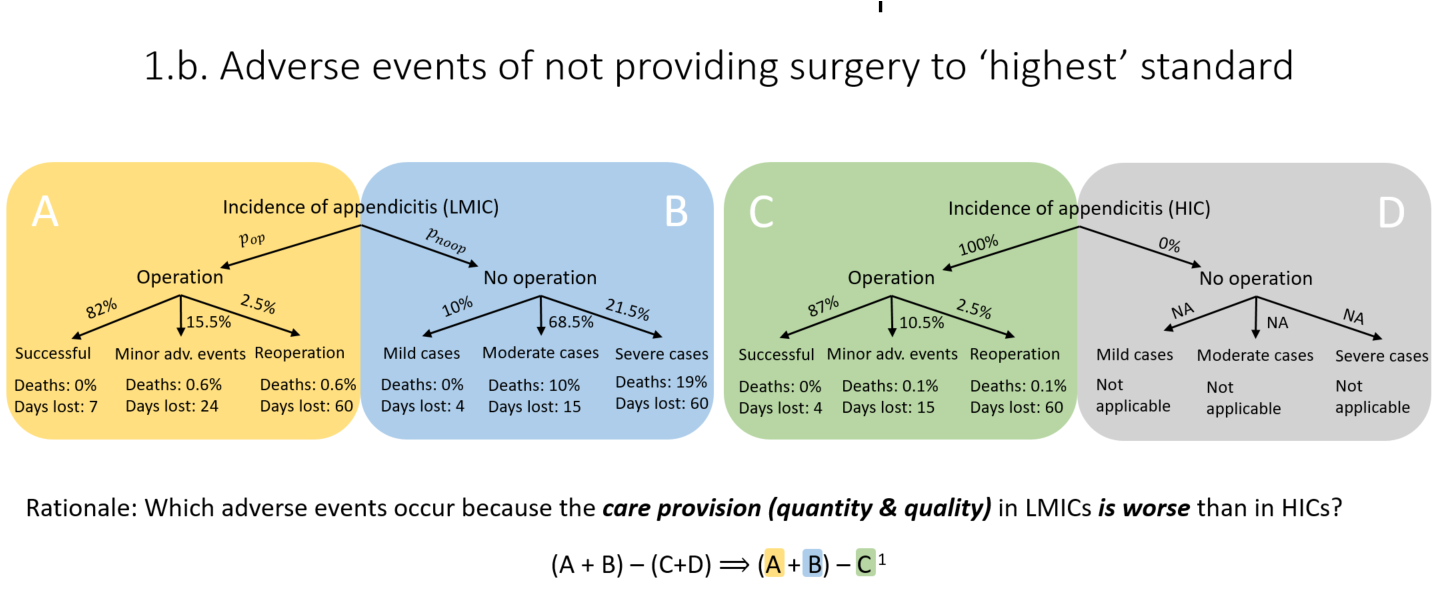


1 We assume that all appendicitis cases are operated in HICs, such that D=0; LMIC = low-and middle income countries; HIC= high income countries

Depending on the outcome of interest, we refer to the result as *rate of excess mortality* $\delta_{i}^{mort}$ respectively the *rate of excess absenteeism* $\delta_{i}^{abs}$. It denotes the (expected) additional mortality risk and working days lost per case of appendicitis due to unmet surgical need or substandard quality of care.

In addition, we estimated the rate of adverse events occurring under the current state of care, i.e., with some individuals treated with local standards, and others remaining untreated as displayed in Figure S 4.

Figure S 4 Graphic display of the total indirect cost calculation of the current state of care


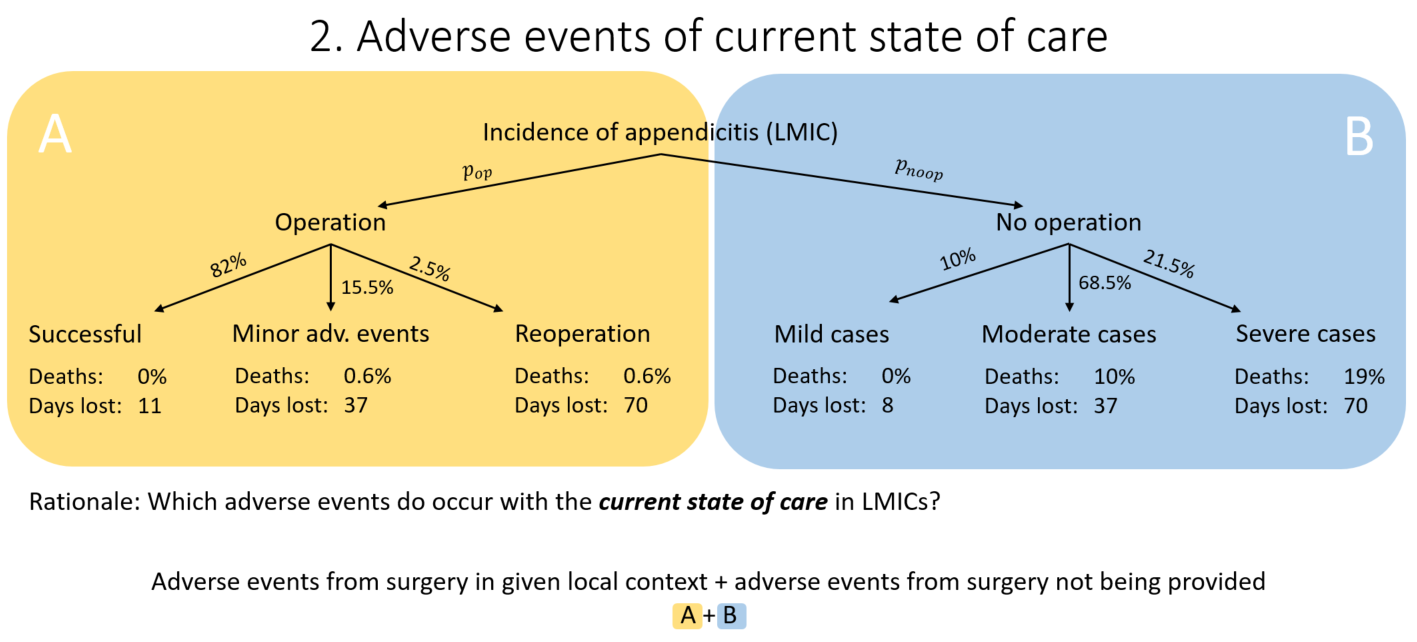


### Income losses

We calculated the income losses due to early mortality following Bommer et al. (2017)^30^. For each country, we calculated by age group how many people were expected to die due to appendicitis by multiplying the incidence of appendicitis with the excess mortality risk from each scenario. Then, we multiplied this excess mortality with the wage the individuals would be expected to have earned if they would not have died. As the existing wage data did not allow a disaggregation by age group, we assumed that the average patient with appendicitis earns the average annual wage.

Expressed as formula, the mortality related income losses for each age group $s$ in a given country $i$ are given as:

$$C_{s,i}^{mort}=annual wage_{i}*N_{s,i}*\sum_{t=0}^{T_{s}-1} \frac{1}{\left( 1+0.03 \right)^{t}}$$

with $N_{s,i}$ as the number of excess deaths in age group $s$ and country $i$, as well as $T_{s}$ as the number of years lost until retirement. To calculate the income losses due to early mortality, we assumed that individuals retire at age 65, age-group specific death occurs in the middle of each age group, and a discount rate of 0.03 per year.

We obtained the number of excess deaths $N_{s,i}$ by multiplying the rate of excess mortality with the incidence of appendicitis:

$$N_{s,i}=\delta_{i}^{mort}*Inc_{s,i}$$

Substituting this into the formula yields:

$$C_{s,i}^{mort}=annual wage_{i}*\delta_{i}^{mort}*Inc_{s,i}*\sum_{t=0}^{T_{s}-1} \frac{1}{\left( 1+0.03 \right)^{t}}$$

Note that within a given country, all variation in mortality related income losses across age groups is based on the variation in incidence and the respective discount factor.

In the same way, we calculated the income losses due to absenteeism following Bommer et al. (2017). For each country, we calculated by age group how many working days in total were lost due to appendicitis by multiplying the incidence of appendicitis (minus those who died) with the excess number of lost working days. Then, we calculated the income losses due to this excess absenteeism by multiplying it with the expected daily earnings.

The formula is given as:

$$C_{s,i}^{abs}=\frac{annual wage_{i}}{261}*N_{s,i}^{LFP}*\delta_{i}^{abs}$$

with $N_{s,i}^{LFP}$ as the number of individuals with unmet surgical need who are available to the labor force. To compute daily wages, we assumed that on average, annual earnings were distributed over 261 working days. As above, the existing data did not allow a disaggregation of wages by age group. We calculated $N_{s,i}^{LFP}$ as follows:

$$N_{s,i}^{LFP}=\left( 1-\delta_{i}^{mort} \right)*Inc_{s,i}$$

Substituting this into the formula yields:

$$C_{s,i}^{abs}=\frac{annual wage_{i}}{261}*\left( 1-\delta_{i}^{mort} \right)*Inc_{s,i}*\delta_{i}^{abs}$$

Note that within a given country, all variation in absenteeism related income losses across age groups is based on the variation in incidence.

### Aggregation and economic burden

The stratum-specific costs were aggregated by country and WHO region for the majority of analyses. For each scenario, the economic burden was calculated as the sum of mortality and absenteeism related income losses.

### Gender differences

Due to the low availability of gender-disaggregated data, a disaggregation of the economic burden by gender would be solely driven by the gendered difference in incidence rates as reported by the GBD estimates. Thus, they are not reported in the main part. An overview on the economic burden disaggregated by gender and region is provided in Figure S 13 and Figure S 14.

## Details on data sources:

The indicators for this study (unmet need, adverse outcomes and their probabilities, wages) are gathered and harmonized from several sources as listed in detail below.

### Incidence of appendicitis

A comparison of data on the incidence of appendicitis from the Global Burden of Disease estimates and similar data from the OECD revealed a large discrepancy, with the GBD data yielding about a twice as high incidence as OECD figures (see Figure S 5). A reason for this discrepancy might be that OECD data covers hospital discharge data, while GBD data is based on various data sources, including household surveys, which are harmonized and imputed for countries with missing data. Unfortunately, OECD data is mainly available for OECD countries, such that we cannot analyze whether the discrepancy between both sources varies over income groups or regions.

Figure S 5 Comparison of GBD incidence rate and OECD incidence rate


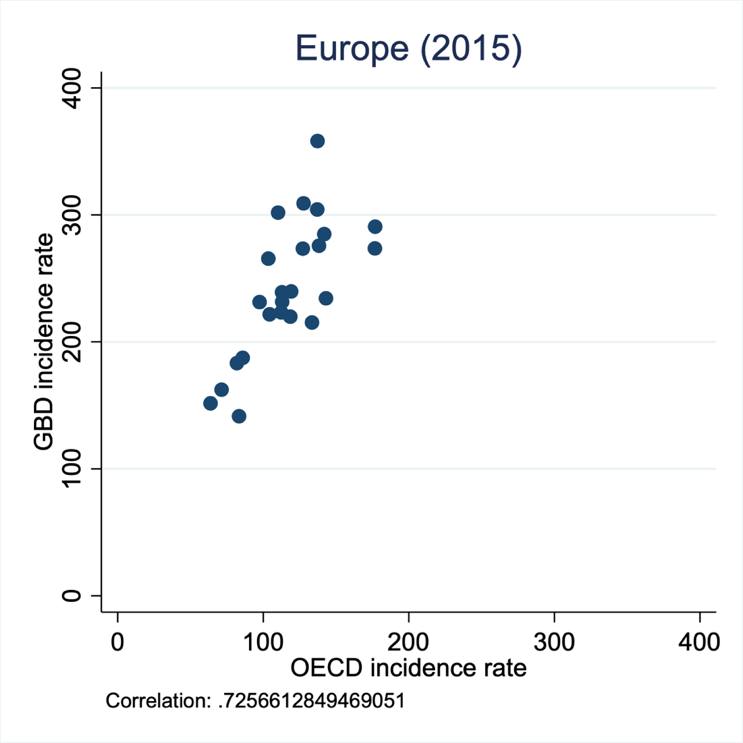


Another data source for the incidence of appendicitis would have been the systematic literature review by Ferris et al.^31^ This review pooled studies on appendicitis and appendectomies to construct estimates for the incidence of appendicitis by region. Assuming that not all appendicitis is treated, this would reveal an underestimate of appendicitis, which is systematically biased towards regions with a poorer access to surgery. Considering only studies on appendicitis, and only studies not older than 2000, only 19 studies remain. Most of them are from high-income countries, only the studies on Canada, South Korea, Taiwan and the US are on the national level, and some regions are covered by no study at all^[[3]](#footnote-3)^. Thus, for most countries and regions, data on appendicitis is missing, except for data provided by the GBD estimates.

### Unmet need

Table S 2 Data sources for indicators used to estimate the unmet surgical need

|  | Indicator name | Source | Comment |
| --- | --- | --- | --- |
| $\mathbf{In}\mathbf{c}_{\mathbf{s},\mathbf{i},\mathbf{y}}$ | Incidence | GBD^6^ | Available by country and year, disaggregated by age-group and sex, 1990-2019 |
| $\mathbf{p}_{\mathbf{noop,i}}\boldsymbol{\& p}_{\mathbf{op,i}}$ | Unmet surgical need in appendicitis approach 1 | Holmer et al (2019)^10^ | Surgical volume by country for the year 2015 (actual or imputed) |
|  |  | LCoGS^1^ | General need for surgery |
|  | Unmet surgical need in appendicitis approach 2 | Holmer et al (2019)^10^ | Surgical volume by country for the year 2015 (actual or imputed) |
|  |  | COVIDSurg Collaborative, GlobalSurg Collaborative data^12^ | Share of appendectomies in GI surgery by income group; derived from the number of appendectomies and other GI surgeries among patients undergoing any elective or emergency surgery in October 2020 in 1,674 hospitals in 112 countries (47 HICs, 33 UMICs, 22 LMICs, 10 LICs); see Table S 3 for income-group aggregates; due to the lower number of observations in LIC, we grouped low- and lower middle income countries in one category as the best available approximation. |
|  |  | Hospital Episode Statistics (HES) data^32^ | Share of GI in total surgery for high-income countries, based on hospital admissions in NHS hospitals in England, aggregated from 2014/15 to 2019/20 |
|  |  | LeBrun et al (2012)^33^  LeBrun et al (2013)^34^  Solis et al (2013)^35^  Bolkan et al (2014)^36^  Knowlton et al 2013^37^  Linden et al 2012^38^  Adde et al (2020)^39^  Albutt et al (2019)^40^  Gyedu et al (2018)^41^  Vansell et al (2014)^42^ | Share of GI in total surgery for low- and middle-income countries derived from the literature for varying years and countries, all available data is aggregated to the income group as depicted in Table S 4 |

Table S 3 Proportion of appendectomies in surgical volume and all its components by income group

|  | Income group | | | |  |
| --- | --- | --- | --- | --- | --- |
|  | High | Upper Middle | Lower Middle | Low | |
| Number of appendectomies ^a^ | 3,338 | 1,513 | 1,372 ^b^ | | |
| Total number of GI surgeries ^a^ | 24,848 | 6,938 | 6,601 ^b^ | | |
| Share of appendectomies in total GI surgery (number of appendectomies / total number of GI surgeries) | 13.43% | 21.81% | 20.78%^b^ | | |
| Share of GI to total surgical volume ^c^ | 9.36% | 21.36% | 36.09% | 45.55% | |
| Share of appendectomies in total surgical volume (Share of appendectomies in GI surgery*share of GI in total surgery) | 1.26% | 4.67% | 7.50% | 9.47% | |

a: from COVIDSurg Collaborative, GlobalSurg Collaborative database^12^; b: as the number of countries in the LIC category is lower than in the other income groups, we grouped LICs and LMICs into one figure; c: from the literature, see Table S 4 for details.

### Systematic review

The literature sources for the proportion of GI surgery in total surgical volume were identified by an Embase Database search. The search string and resulting numbers of studies are listed below.

1 surgical volume/ (680)

2 surgical capacity/ (4)

3 (Surgical adj8 an?esthesia adj8 capacity).ti,ab,kw. (36)

4 (Enumeration adj8 Operation?).ti,ab,kw. (18)

5 (Surgery adj8 an?esthesia adj8 capacity).ti,ab,kw. (48)

6 1 or 2 or 3 or 4 or 5 (777)

7 limit 6 to (human and english language and (article or article in press or "review")) (

Two reviewers (MK) and (MM) independently reviewed the articles to assess if they were peer reviewed papers that contained data on the proportion of surgeries that were GI surgeries. At the end disagreements between the reviewers were resolved by discussions and 10 articles were selected for data collection (see Table S 4). One study from Uganda^38^ was excluded as it reports emergency surgeries separately, making the total share not comparable to the other studies.

As several studies were available for lower-middle- and low-income countries, we selected the studies which the highest sample size from each income category. Hence, we used the proportion of surgeries that were GI surgeries were sourced from Bangladesh^34^ and Sierra Leone^36^ to represent lower-middle- and low-income countries respectively.

Table S 4 Literature sources and data for the proportion of GI surgery to total surgical volume for low- and middle-income countries

| Income group | Country and reference | Year | Proportion of GI to total surgical volume |
| --- | --- | --- | --- |
| Upper Middle | Guyana^42^ | Not reported | 21.36% |
| Lower Middle | Bolivia^33^ | 2011 | 45.29% |
|  | Nicaragua^35^ | 2010 | 50.44% |
|  | Bangladesh^34^ | 2012 | 36.09% |
|  | Ghana^41^ | 2014/15 | 18.41% |
| Low | Liberia^37^ | 2009-10 | 50.00% |
|  | Liberia^39^ | 2018 | 39.7% |
|  | Uganda^40^ | 2016 | 23.76% |
|  | Sierra Leone^36^ | 2012 | 45.55% |

### Probabilities and outcomes of surgical scenarios

#### Surgically treated appendicitis

For the probabilities and outcomes of the three operative scenarios, we relied on data from the GlobalSurg 1 and 2 studies^3,15^. These contain individual data from 8,611 patients (4,532 from GS1 and 4,079 from GS2) who underwent surgical treatment for appendicitis across countries in all income groups. The figures were calculated by combining low- and middle-income country data as there are too few observations from LICs to make it a separate category. We categorized three surgical scenarios: Successful surgery (succ) was defined as discharge from follow up without wound infection or reoperation in the first 30 days. Surgery with minor adverse (min) events was defined as wound infection identified within the first 30 days of surgery. Reoperation (reop) were those cases that returned to theatre for further surgery in the first 30 days following appendectomy. The respective mortality outcomes are retrieved from GlobalSurg 1 and 2 studies^3,15^, and the absenteeism outcomes from the literature as listed in detail in Table S 5.

Table S 5 Data sources for indicators on probabilities and outcomes of surgical scenarios

| Indicator name | Source | Value | Comment |
| --- | --- | --- | --- |
| *Probability of scenarios after operation:* | | |  |
| Successful surgery ($\mathbf{p}_{\mathbf{succ}}$) | GlobalSurg1 and 2^3,15^ | LMIC: 82%  HIC: 87% | Defined as discharge from follow up without wound infection or reoperation in the first 30 days of the appendectomy. |
| Minor adverse events ($\mathbf{p}_{\mathbf{min}}$) | GlobalSurg1 and 2^3,15^ | LMIC: 15.5%  HIC: 10.5% | Defined as wound infection identified within the first 30 days of the appendectomy. |
| Reoperation ($\mathbf{p}_{\mathbf{reop}}$) | GlobalSurg1 and 2^3,15^ | LMIC: 2.5%  HIC: 2.5% | Defined as those cases that returned to theatre for further surgery in the first 30 days after appendectomy. |
| *Outcomes after operation:* $\boldsymbol{\omega}$ | |  |  |
| Death rates by scenario | GlobalSurg1 and 2^3,15^ | $\boldsymbol{\omega}_{\mathbf{succ}}\boldsymbol{:}$ LMIC 0%  HIC: 0%  $\boldsymbol{\omega}_{\mathbf{min}}\boldsymbol{:}$ LMIC 0.6%  HIC: 0.1%  $\boldsymbol{\omega}_{\mathbf{reop}}$**:** LMIC 0.6%  HIC: 0.1% | Defined as mortality within the first 30 days of the appendectomy. |
| Lost working days by scenario | Di Saverio et al (2014)^43^ | $\boldsymbol{\omega}_{\mathbf{succ}}\boldsymbol{:}$ LMIC: 7 | Based upon recovery from open appendectomy in HIC (Italy): rounded value for 6.6 days reported by Di Saverio et al (2014). The assumption is that LMICs will have more open appendectomies hence we used HIC data in the absence of studies from LMICs. |
|  | Martin et al (1995)^44^ | $\boldsymbol{\omega}_{\mathbf{min}}\boldsymbol{:}$ LMIC: 24 | Based on the recovery of patients undergoing open appendectomy for acute appendicitis in HIC. Rounded from 23.6 days to 24 days returning to work reported in the study. The assumption is that LMICs will have more open appendectomies hence we used HIC data in the absence of studies from LMICs |
|  | Opmeer et al (2010)^45^ | $\boldsymbol{\omega}_{\mathbf{reop}}$**:** LMIC: 60 | Estimated as the weighted average of lost working days reported in the study among patients undergoing on-demand (50 days) and planned re-laparotomy (70 days) for severe peritonitis in a HIC. |
|  | Di Saverio et al (2014)^43^ | $\boldsymbol{\omega}_{\mathbf{succ}}\boldsymbol{:}$ HIC: 4 | The authors found that laparoscopic and open appendectomy surgery patients had 2.1 and 6.6 days return to daily activities respectively. We estimated 4 as a weighted the average for patients undergoing HIC appendectomy. |
|  | Long et al (2001)^46^ | $\boldsymbol{\omega}_{\mathbf{min}}\boldsymbol{:}$ HIC: 15 | A prospective randomised comparison that compared laparoscopic versus open appendectomy in USA. The study found that appendectomy patients have 14.6 days off work or school after the surgery (for both laparoscopic and open appendectomy). The value has been rounded to the nearest whole number. |
|  | Opmeer et al (2010)^45^ | $\boldsymbol{\omega}_{\mathbf{reop}}$**:** HIC: 60 | Estimated as the weighted average of lost working days reported in the study among patients undergoing on-demand (50 days) and planned re-laparotomy (70 days) for severe peritonitis in a HIC. |

#### Not surgically treated appendicitis

For the non-operative scenarios, we distinguish mild, moderate and severe cases of appendicitis. For the probabilities of each scenario, we relied on the data from GlobalSurg 1 and 2 studies^3,15^ for high income countries only as we assume that all appendicitis cases are treated surgically in these countries. We apply this distribution to all countries assuming there is little variation in pathogenesis globally and hence apparent global variation in disease severity is due to delayed presentation and treatment. Mild cases (mild) were defined as non-gangenous appendicitis with a normal appendix at surgery. Moderate cases (mod) were defined as those with purulent peritonitis (Infection within the abdominal cavity, beyond the walls of the appendix – without perforation)*.* Severe cases (sev) were defined as those with a perforated appendix (perforation of a gangrenous appendix with localised or generalised faecal contamination). For the mortality outcomes, it is assumed that mild cases resolve with conservative treatment and are thus expected to not result in mortality. Moderate cases are considered comparable to (treated) emergency acute abdomen, using a national audit from the UK^47^. This figure of 10% is also replicated in the mortality from ‘all-comer’ laparotomies in LIC in the GlobalSurg2^3^ data. Severe cases (perforated appendicitis) are considered to carry a mortality comparable to that of (treated) faecal peritonitis in high-income countries using data from the GenOSept study^48^. This study provided data for 977 faecal peritonitis patients, recruited in 102 centres across 16 countries between 29/09/2005 and 5/01/2011. The median age was 69.2 years (IQR, Interquartile range 58.3-77.1). The most common causes of faecal peritonitis were perforated diverticular disease (32.1%) and surgical anastomotic breakdown (31.1%). The mortality rate at 28 days was 19.1% and 31.6% at six months. We have purposely made this a conservative estimate as the mortality and morbidity figures are based on treated cases of emergency presentation with faecal/purulent peritonitis. It is assumed that the true adverse outcomes will be higher because a proportion of cases will not reach hospital presentation. For estimates of absenteeism, we again largely used recovery times based on HIC data. This data was however adjusted for lower rates of laparoscopic procedure in LMICs, and also incorporated the higher SSI rates see in LMICs resulting in additional delayed recovery.

Table S 6 Data sources for indicators on probabilities and outcomes of scenarios without surgery

| Indicator name | Source | Value | Comment |
| --- | --- | --- | --- |
| *Probability of scenarios after no operation:* | | |  |
| Mild case ($\mathbf{p}_{\mathbf{mild}}$) | GlobalSurg1 and 2^3,15^ for HICs | 10% | Normal appendix (at surgery) in HIC |
| Moderate case ($\mathbf{p}_{\mathbf{mod}}$) | GlobalSurg1 and 2^3,15^ for HICs | 68.5% | Purulent peritonitis (Infection within the abdominal cavity, beyond the walls of the appendix – without perforation) at surgery in HIC |
| Severe case ($\mathbf{p}_{\mathbf{sev}}$) | GlobalSurg1 and 2^3,15^ for HICs | 21.5% | Perforated appendix (perforation of a gangrenous appendix with localised or generalised faecal contamination) at surgery in HIC |
| *Outcomes without operation (*$\boldsymbol{\omega}$***)*** | |  |  |
| Death rates by scenario |  | $\boldsymbol{\omega}_{\mathbf{mild}}\boldsymbol{:}$ LMIC: 0% | Zero mortality in mild cases is a conservative assumption. |
|  | NELA ^47^, GlobalSurg1^15^ | $\boldsymbol{\omega}_{\mathbf{mod}}\boldsymbol{:}$ LMIC: 10% | Mortality of moderate (purulent peritonitis) cases as provided by NELA and validated by GlobalSurg1. |
|  | GenOSept Investigators (2014)^48^ | $\boldsymbol{\omega}_{\boldsymbol{sev}}\boldsymbol{:}$ LMIC: 19% | Mortality of severe cases is proxied by faecal peritonitis. This assumes that patients with gangrenous appendicitis will come to delayed surgery with faecal peritonitis (a best case scenario). |
| Lost working days by scenario | Di Saverio et al (2014)^49^ | $\boldsymbol{\omega}_{\mathbf{mild}}\boldsymbol{:}$ LMIC: 4 | The authors found that laparoscopic and open appendectomy surgery patients had 2.1 and 6.6 days return to daily activities respectively. We estimated 4 as a weighted the average for patients undergoing HIC appendectomy. |
|  | Long et al (2001)^46^ | $\boldsymbol{\omega}_{\mathbf{mod}}\boldsymbol{:}$ LMIC: 15 | A prospective randomised comparison that compared laparoscopic versus open appendectomy in USA. The study found that appendectomy patients have 14.6 days off work or school after the surgery (for both laparoscopic and open appendectomy). The value has been rounded to the nearest whole number. |
|  | Opmeer et al (2010)^45^ | $\boldsymbol{\omega}_{\boldsymbol{sev}}\boldsymbol{:}$ LMIC: 60 | Estimated as the weighted average of lost working days reported in the study among patients undergoing on-demand (50 days) and planned re-laparotomy (70 days) for severe peritonitis in a HIC Opmeer et al (2010). |

### Wage data

Data for annual wages are not available from a single source for all countries and therefore combined with the following procedure. As listed in detail in Table S 7, wage data can be retrieved from OECD annual wages, ILO monthly wages or proxied by labor shares in GDP (as provided by the ILO) multiplied with GDP per labor force member. Table S 8 provides an overview of the data availability from each source for each country. For the estimation of income losses, we use the wage of the year 2015 as this requires least assumptions regarding the surgical volume, which is only available for the year 2015. If data from several sources is available for the most recent year, we applied the following strategy: priority is given to OECD wage data, if this is not available, ILO wage data is chosen and only if none of these is available, the wage proxy based on labor shares is used. The wage data source, which is used for each country is listed in Table S 9. For the OECD countries, all wage indicators are available, so that the alternative measures can be validated against it (Figure S 6, Figure S 7).

Table S 7 Overview wage data

|  | Indicator name | Source | Comment |
| --- | --- | --- | --- |
| $\mathbf{annual} \mathbf{wag}\mathbf{e}_{\mathbf{i},\mathbf{y}}$ | Wage | OECD^18^, World Bank^50^ | Average annual wages for all (at the time) OECD countries as provided by the OECD from 1990-2019 in 2019 int. $ PPP; deflated to 2015 int. $ PPP using GDP Deflator as provided by the World Bank |
|  | Wage | ILO^16^ | Average monthly wages for varying list of countries as provided by ILO from 2008-2019 in 2015 int. $ PPP; multiplied by 12 for average annual wage |
|  | Wage proxy from labor shares in GDP | ILO^17^, World Bank^50^ | Modeled and imputed labor shares in GDP for varying list of countries from 2004 – 2017 as provided by ILO; multiplied by GDP per labor force member (int. $ 2015 PPP) for annual wage proxy |
|  | GDP per labor force member, GDP, population | World Bank^50^ | GDP and GDP per labor force member in int. $ PPP and total population number as provided by the World Bank from 1990-2019 |

Table S 8 Overview included wage data sources

| Wage data source for 2015 | Number of countries |
| --- | --- |
| OECD wage | 35 |
| ILO wage | 45 |
| ILO labor share | 92 |
| None | 31 |

Figure S 6 Comparison of ILO and OECD wage in 2015


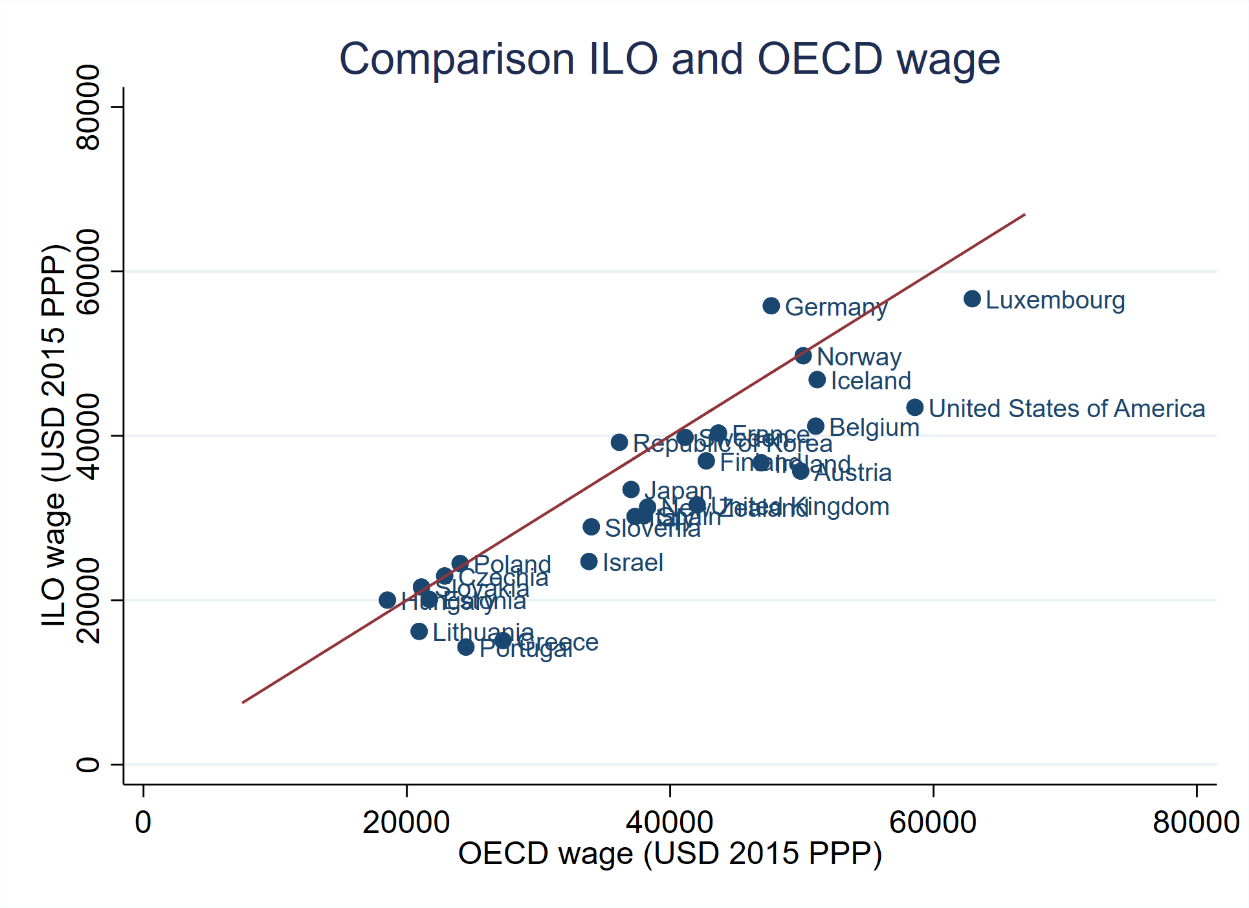


Figure S 7 Comparison of ILO labor share wage proxy and OECD wage in 2015


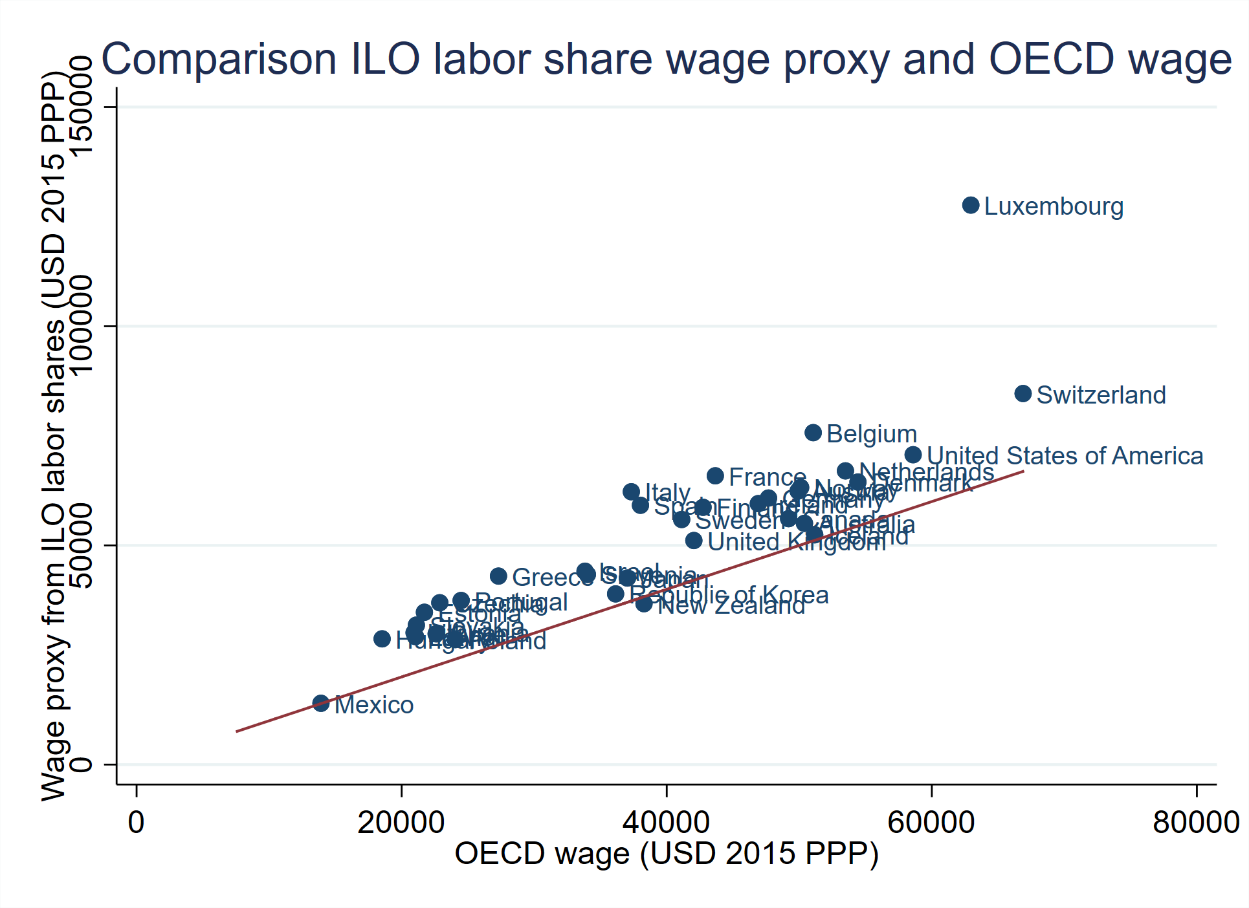


Table S 9 Country-wise overview of all inputs and unmet need estimates

| Country | Income group | WHO region | Incidence rate | Surgical volume per 100,000 | Estimated appendectomies | Average annual wage | Data source for wage | GDP in mn USD PPP | Population in thousand | Unmet need in %  (Appr. 1) | Unmet need in %  (Appr. 2) |
| --- | --- | --- | --- | --- | --- | --- | --- | --- | --- | --- | --- |
| Afghanistan | LIC | Eastern Mediterranean | 267 | 692 | 65 | 3,916 | ILO labor share | 71,068 | 34,414 | 86 | 75 |
| Albania | UMIC | Europe | 201 | 1,911 | 89 | 10,592 | ILO wage | 34,052 | 2,881 | 62 | 56 |
| Algeria | UMIC | Africa | 278 | 3,823 | 178 | 18,555 | ILO labor share | 430,121 | 39,728 | 24 | 36 |
| Angola | UMIC | Africa | 280 | 1,349 | 63 | 7,160 | ILO labor share | 150,081 | 27,884 | 73 | 78 |
| Argentina | UMIC | Americas | 283 | 6,527 | 304 | 19,182 | ILO labor share | 580,542 | 43,132 | 0 | 0 |
| Armenia | LMIC | Europe | 222 | 4,343 | 326 | 8,202 | ILO wage | 32,337 | 2,926 | 13 | 0 |
| Australia | HIC | Western Pacific | 255 | 28,823 | 362 | 50,390 | OECD wage | 1,102,934 | 23,816 | 0 | 0 |
| Austria | HIC | Europe | 358 | 13,648 | 172 | 49,913 | OECD wage | 445,632 | 8,643 | 0 | 0 |
| Azerbaijan | UMIC | Europe | 231 | 1,843 | 86 | 12,732 | ILO wage | 107,583 | 9,649 | 63 | 63 |
| Bahamas | HIC | Americas | 284 | 7,386 | 93 | 40,082 | ILO labor share | 12,992 | 374 | 0 | 0 |
| Bahrain | HIC | Eastern Mediterranean | 284 | 3,790 | 48 | 24,627 | ILO labor share | 62,394 | 1,372 | 0 | 0 |
| Bangladesh | LMIC | South-East Asia | 1,454 | 338 | 25 | 3,645 | ILO labor share | 509,716 | 156,256 | 93 | 98 |
| Barbados | HIC | Americas | 258 | 6,763 | 85 | 18,254 | ILO labor share | 4,281 | 285 | 0 | 0 |
| Belarus | UMIC | Europe | 271 | 18,394 | 857 | 15,364 | ILO labor share | 147,625 | 9,490 | 0 | 0 |
| Belgium | HIC | Europe | 300 | 20,888 | 263 | 51,053 | OECD wage | 538,955 | 11,274 | 0 | 0 |
| Belize | UMIC | Americas | 291 | 1,922 | 90 | 10,189 | ILO wage | 2,507 | 361 | 62 | 69 |
| Benin | LIC | Africa | 138 | 332 | 31 | 3,617 | ILO labor share | 31,059 | 10,576 | 93 | 77 |
| Bhutan | LMIC | South-East Asia | 1,396 | 2,597 | 195 | 9,034 | ILO labor share | 6,692 | 728 | 48 | 86 |
| Bolivia | LMIC | Americas | 771 | 2,132 | 160 | 24,713 | ILO wage | 82,952 | 10,870 | 57 | 79 |
| Bosnia and Herzegovina | UMIC | Europe | 177 | 4,550 | 212 | 10,472 | ILO wage | 42,136 | 3,429 | 9 | 0 |
| Botswana | UMIC | Africa | 351 | 4,348 | 203 | 18,010 | ILO labor share | 30,856 | 2,121 | 13 | 42 |
| Brazil | UMIC | Americas | 123 | 13,674 | 637 | 9,438 | ILO wage | 2,748,398 | 204,472 | 0 | 0 |
| Brunei Darussalam | HIC | Western Pacific | 447 | 6,032 | 76 | 69,177 | ILO labor share | 27,484 | 415 | 0 | 0 |
| Bulgaria | UMIC | Europe | 179 | 7,092 | 330 | 13,036 | ILO wage | 132,560 | 7,178 | 0 | 0 |
| Burkina Faso | LIC | Africa | 137 | 300 | 28 | 2,704 | ILO labor share | 33,509 | 18,111 | 94 | 79 |
| Burundi | LIC | Africa | 162 | 249 | 24 | 1,006 | ILO labor share | 7,451 | 10,160 | 95 | 85 |
| Cabo Verde | LMIC | Africa | 159 | 1,881 | 141 | 7,709 | ILO wage | 3,274 | 525 | 62 | 11 |
| Cambodia | LMIC | Western Pacific | 290 | 817 | 61 | 5,085 | ILO wage | 51,404 | 15,521 | 84 | 79 |
| Cameroon | LMIC | Africa | 144 | 738 | 55 | 2,920 | ILO labor share | 78,545 | 23,298 | 85 | 61 |
| Canada | HIC | Americas | 175 | 9,314 | 117 | 49,195 | OECD wage | 1,647,308 | 35,703 | 0 | 0 |
| Central African Republic | LIC | Africa | 260 | 162 | 15 | 1,127 | ILO labor share | 3,495 | 4,493 | 97 | 94 |
| Chad | LIC | Africa | 130 | 47 | 4 | 2,836 | ILO labor share | 26,570 | 14,111 | 99 | 97 |
| Chile | HIC | Americas | 339 | 6,682 | 84 | 22,602 | OECD wage | 402,456 | 17,969 | 0 | 0 |
| China | UMIC | Western Pacific | 173 | 2,691 | 125 | 14,572 | ILO wage | 16,464,849 | 1,371,220 | 46 | 28 |
| Colombia | UMIC | Americas | 422 | 27,385 | 1,276 | 13,947 | ILO labor share | 607,997 | 47,521 | 0 | 0 |
| Comoros | LIC | Africa | 178 | 672 | 64 | 4,109 | ILO labor share | 2,253 | 777 | 87 | 64 |
| Congo | LMIC | Africa | 259 | 676 | 51 | 6,962 | ILO labor share | 23,740 | 4,856 | 86 | 80 |
| Costa Rica | UMIC | Americas | 406 | 3,707 | 173 | 16,926 | ILO wage | 86,247 | 4,848 | 26 | 57 |
| Croatia | HIC | Europe | 224 | 6,162 | 77 | 38,222 | ILO labor share | 102,491 | 4,204 | 0 | 0 |
| Cyprus | HIC | Europe | 197 | 3,037 | 38 | 2,603 | ILO wage | 29,164 | 1,161 | 0 | 0 |
| Czechia | HIC | Europe | 220 | 6,247 | 79 | 22,876 | OECD wage | 373,453 | 10,546 | 0 | 0 |
| Côte d'Ivoire | LMIC | Africa | 134 | 896 | 67 | 4,094 | ILO labor share | 106,745 | 23,226 | 82 | 50 |
| DR Congo | LIC | Africa | 298 | 198 | 19 | 852 | ILO labor share | 54,403 | 76,245 | 96 | 94 |
| Denmark | HIC | Europe | 266 | 9,926 | 125 | 54,427 | OECD wage | 297,287 | 5,683 | 0 | 0 |
| Djibouti | LMIC | Eastern Mediterranean | 168 | 979 | 73 | 4,899 | ILO labor share | 4,074 | 914 | 80 | 56 |
| Dominican Republic | UMIC | Americas | 294 | 4,384 | 204 | 6,157 | ILO wage | 148,319 | 10,282 | 12 | 31 |
| Ecuador | UMIC | Americas | 814 | 1,501 | 70 | 13,404 | ILO labor share | 185,652 | 16,212 | 70 | 91 |
| Egypt | LMIC | Eastern Mediterranean | 260 | 2,039 | 153 | 7,089 | ILO wage | 748,147 | 92,443 | 59 | 41 |
| El Salvador | LMIC | Americas | 398 | 3,773 | 283 | 7,013 | ILO wage | 50,674 | 6,325 | 25 | 29 |
| Equatorial Guinea | UMIC | Africa | 295 | 3,754 | 175 | 38,894 | ILO labor share | 31,315 | 1,169 | 25 | 41 |
| Estonia | HIC | Europe | 263 | 9,703 | 122 | 21,717 | OECD wage | 38,976 | 1,315 | 0 | 0 |
| Eswatini | LMIC | Africa | 309 | 3,186 | 239 | 12,594 | ILO labor share | 8,345 | 1,104 | 36 | 23 |
| Ethiopia | LIC | Africa | 39 | 249 | 24 | 1,500 | ILO labor share | 152,328 | 100,835 | 95 | 39 |
| Fiji | UMIC | Western Pacific | 118 | 2,309 | 108 | 13,740 | ILO labor share | 10,460 | 869 | 54 | 9 |
| Finland | HIC | Europe | 240 | 11,346 | 143 | 42,742 | OECD wage | 245,537 | 5,480 | 0 | 0 |
| France | HIC | Europe | 223 | 9,069 | 114 | 43,664 | OECD wage | 2,863,821 | 66,548 | 0 | 0 |
| Gabon | UMIC | Africa | 270 | 2,654 | 124 | 15,798 | ILO labor share | 30,395 | 1,948 | 47 | 54 |
| Gambia | LIC | Africa | 148 | 339 | 32 | 2,440 | ILO labor share | 3,832 | 2,086 | 93 | 78 |
| Georgia | UMIC | Europe | 197 | 5,097 | 237 | 11,187 | ILO wage | 42,178 | 3,725 | 0 | 0 |
| Germany | HIC | Europe | 304 | 9,354 | 118 | 47,680 | OECD wage | 4,076,146 | 81,687 | 0 | 0 |
| Ghana | LMIC | Africa | 154 | 951 | 71 | 6,225 | ILO wage | 102,270 | 27,849 | 81 | 54 |
| Greece | HIC | Europe | 204 | 7,191 | 90 | 27,306 | OECD wage | 309,238 | 10,821 | 0 | 0 |
| Guatemala | LMIC | Americas | 514 | 1,423 | 107 | 6,985 | ILO wage | 121,046 | 15,567 | 72 | 79 |
| Guinea | LIC | Africa | 136 | 259 | 25 | 3,335 | ILO labor share | 20,469 | 11,432 | 95 | 82 |
| Guinea-Bissau | LIC | Africa | 143 | 431 | 41 | 1,580 | ILO labor share | 2,782 | 1,737 | 91 | 71 |
| Guyana | UMIC | Americas | 293 | 2,453 | 114 | 17,970 | ILO labor share | 8,389 | 767 | 51 | 61 |
| Haiti | LIC | Americas | 218 | 609 | 58 | 3,025 | ILO labor share | 25,315 | 10,696 | 88 | 74 |
| Honduras | LMIC | Americas | 519 | 2,334 | 175 | 6,879 | ILO wage | 44,434 | 9,113 | 53 | 66 |
| Hungary | HIC | Europe | 187 | 14,048 | 177 | 18,524 | OECD wage | 257,643 | 9,843 | 0 | 0 |
| Iceland | HIC | Europe | 279 | 9,249 | 116 | 51,165 | OECD wage | 16,642 | 331 | 0 | 0 |
| India | LMIC | South-East Asia | 121 | 734 | 55 | 7,280 | ILO labor share | 6,669,901 | 1,310,152 | 85 | 55 |
| Indonesia | LMIC | South-East Asia | 69 | 1,394 | 105 | 4,325 | ILO wage | 2,454,679 | 258,383 | 72 | 0 |
| Iran | UMIC | Eastern Mediterranean | 225 | 4,230 | 197 | 14,220 | ILO labor share | 873,893 | 78,492 | 15 | 12 |
| Iraq | UMIC | Eastern Mediterranean | 288 | 2,008 | 94 | 11,858 | ILO labor share | 369,498 | 35,572 | 60 | 68 |
| Ireland | HIC | Europe | 234 | 2,996 | 38 | 46,909 | OECD wage | 331,912 | 4,702 | 0 | 0 |
| Israel | HIC | Europe | 239 | 4,783 | 60 | 33,833 | OECD wage | 311,611 | 8,380 | 0 | 0 |
| Italy | HIC | Europe | 162 | 8,254 | 104 | 37,329 | OECD wage | 2,407,093 | 60,731 | 0 | 0 |
| Jamaica | UMIC | Americas | 289 | 3,839 | 179 | 12,897 | ILO labor share | 24,556 | 2,891 | 23 | 38 |
| Japan | HIC | Western Pacific | 303 | 8,909 | 112 | 37,028 | OECD wage | 5,070,943 | 127,141 | 0 | 0 |
| Jordan | UMIC | Eastern Mediterranean | 296 | 2,473 | 115 | 16,389 | ILO labor share | 91,222 | 9,267 | 51 | 61 |
| Kazakhstan | UMIC | Europe | 233 | 4,459 | 208 | 13,255 | ILO wage | 337,179 | 17,543 | 11 | 11 |
| Kenya | LMIC | Africa | 55 | 824 | 62 | 3,402 | ILO labor share | 156,382 | 47,878 | 84 | 0 |
| Kuwait | HIC | Eastern Mediterranean | 295 | 6,775 | 85 | 26,442 | ILO labor share | 194,233 | 3,836 | 0 | 0 |
| Kyrgyzstan | LMIC | Europe | 243 | 3,045 | 228 | 8,016 | ILO wage | 25,385 | 5,957 | 39 | 6 |
| Lao PDR | LMIC | Western Pacific | 319 | 601 | 45 | 6,041 | ILO labor share | 42,039 | 6,741 | 88 | 86 |
| Latvia | HIC | Europe | 276 | 18,404 | 231 | 21,042 | OECD wage | 50,851 | 1,978 | 0 | 0 |
| Lebanon | UMIC | Eastern Mediterranean | 282 | 5,444 | 254 | 23,436 | ILO labor share | 102,458 | 6,533 | 0 | 10 |
| Lesotho | LMIC | Africa | 294 | 1,104 | 83 | 5,117 | ILO labor share | 5,497 | 2,059 | 78 | 72 |
| Liberia | LIC | Africa | 150 | 813 | 77 | 1,415 | ILO labor share | 6,892 | 4,472 | 84 | 49 |
| Libya | UMIC | Eastern Mediterranean | 290 | 3,943 | 184 | 14,945 | ILO labor share | 63,446 | 6,418 | 21 | 37 |
| Lithuania | HIC | Europe | 291 | 10,189 | 128 | 20,935 | OECD wage | 84,625 | 2,905 | 0 | 0 |
| Luxembourg | HIC | Europe | 302 | 10,054 | 126 | 62,944 | OECD wage | 61,572 | 570 | 0 | 0 |
| Madagascar | LIC | Africa | 161 | 212 | 20 | 2,577 | ILO wage | 32,776 | 24,234 | 96 | 88 |
| Malawi | LIC | Africa | 173 | 367 | 35 | 671 | ILO labor share | 12,682 | 16,745 | 93 | 80 |
| Malaysia | UMIC | Western Pacific | 256 | 4,329 | 202 | 16,028 | ILO wage | 711,234 | 30,271 | 13 | 21 |
| Maldives | UMIC | South-East Asia | 227 | 6,442 | 300 | 13,968 | ILO labor share | 7,738 | 455 | 0 | 0 |
| Mali | LIC | Africa | 142 | 466 | 44 | 3,508 | ILO wage | 36,118 | 17,439 | 91 | 69 |
| Malta | HIC | Europe | 184 | 12,851 | 162 | 42,959 | ILO labor share | 17,101 | 445 | 0 | 0 |
| Mauritania | LMIC | Africa | 151 | 609 | 46 | 8,100 | ILO labor share | 17,996 | 4,046 | 88 | 70 |
| Mauritius | UMIC | Africa | 208 | 2,054 | 96 | 12,738 | ILO wage | 24,280 | 1,263 | 59 | 54 |
| Mexico | UMIC | Americas | 267 | 1,282 | 60 | 13,909 | OECD wage | 2,090,411 | 121,858 | 74 | 78 |
| Mongolia | LMIC | Western Pacific | 363 | 5,168 | 388 | 10,627 | ILO wage | 29,251 | 2,998 | 0 | 0 |
| Montenegro | UMIC | Europe | 190 | 4,312 | 201 | 18,202 | ILO wage | 10,446 | 622 | 14 | 0 |
| Morocco | LMIC | Eastern Mediterranean | 261 | 746 | 56 | 10,168 | ILO labor share | 245,549 | 34,664 | 85 | 79 |
| Mozambique | LIC | Africa | 189 | 296 | 28 | 1,230 | ILO labor share | 27,904 | 27,042 | 94 | 85 |
| Myanmar | LMIC | South-East Asia | 265 | 644 | 48 | 3,925 | ILO wage | 202,381 | 52,681 | 87 | 82 |
| Namibia | UMIC | Africa | 311 | 4,512 | 210 | 14,049 | ILO labor share | 20,683 | 2,315 | 10 | 32 |
| Nepal | LIC | South-East Asia | 874 | 198 | 19 | 1,887 | ILO labor share | 68,198 | 27,015 | 96 | 98 |
| Netherlands | HIC | Europe | 231 | 9,428 | 119 | 53,483 | OECD wage | 885,011 | 16,940 | 0 | 0 |
| New Zealand | HIC | Western Pacific | 263 | 6,099 | 77 | 38,285 | OECD wage | 178,391 | 4,609 | 0 | 0 |
| Nicaragua | LMIC | Americas | 444 | 4,585 | 344 | 7,014 | ILO labor share | 32,175 | 6,223 | 8 | 23 |
| Niger | LIC | Africa | 137 | 245 | 23 | 999 | ILO labor share | 22,155 | 20,002 | 95 | 83 |
| Nigeria | LMIC | Africa | 77 | 1,192 | 89 | 10,406 | ILO labor share | 820,655 | 181,137 | 76 | 0 |
| North Macedonia | UMIC | Europe | 186 | 3,845 | 179 | 18,073 | ILO labor share | 29,559 | 2,079 | 23 | 4 |
| Norway | HIC | Europe | 273 | 10,490 | 132 | 50,104 | OECD wage | 320,119 | 5,189 | 0 | 0 |
| Oman | HIC | Eastern Mediterranean | 301 | 2,452 | 31 | 30,911 | ILO labor share | 131,471 | 4,267 | 0 | 0 |
| Pakistan | LMIC | Eastern Mediterranean | 128 | 413 | 31 | 5,700 | ILO wage | 817,026 | 199,427 | 92 | 76 |
| Palestine | LMIC |  | 291 |  |  | 11,059 | ILO wage | 24,707 | 4,270 |  | 0 |
| Panama | UMIC | Americas | 412 | 6,378 | 297 | 16,355 | ILO wage | 108,733 | 3,968 | 0 | 28 |
| Papua New Guinea | LMIC | Western Pacific | 106 | 920 | 69 | 3,868 | ILO labor share | 30,011 | 8,108 | 82 | 35 |
| Paraguay | UMIC | Americas | 263 | 3,991 | 186 | 12,727 | ILO labor share | 74,271 | 6,689 | 20 | 29 |
| Peru | UMIC | Americas | 876 | 2,850 | 133 | 9,959 | ILO labor share | 345,488 | 30,471 | 43 | 85 |
| Philippines | LMIC | Western Pacific | 89 | 1,608 | 121 | 6,996 | ILO wage | 719,327 | 102,113 | 68 | 0 |
| Poland | HIC | Europe | 152 | 5,980 | 75 | 24,046 | OECD wage | 1,036,711 | 37,986 | 0 | 0 |
| Portugal | HIC | Europe | 141 | 8,602 | 108 | 24,487 | OECD wage | 313,168 | 10,358 | 0 | 0 |
| Puerto Rico | HIC |  | 263 |  |  | 66,598 | ILO labor share | 114,192 | 3,473 | 0 | 0 |
| Qatar | HIC | Eastern Mediterranean | 305 | 7,717 | 97 | 42,668 | ILO wage | 250,957 | 2,566 | 0 | 0 |
| Republic of Korea | HIC | Western Pacific | 362 | 3,351 | 42 | 36,143 | OECD wage | 1,907,146 | 51,015 | 0 | 0 |
| Republic of Moldova | LMIC | Europe | 273 | 4,902 | 368 | 9,350 | ILO wage | 26,156 | 2,835 | 2 | 0 |
| Romania | UMIC | Europe | 176 | 4,602 | 214 | 21,106 | ILO labor share | 442,622 | 19,816 | 8 | 0 |
| Russian Federation | UMIC | Europe | 204 | 6,595 | 307 | 24,873 | ILO labor share | 3,454,720 | 144,097 | 0 | 0 |
| Rwanda | LIC | Africa | 190 | 648 | 61 | 1,202 | ILO labor share | 18,920 | 11,369 | 87 | 68 |
| SVG | UMIC | Americas | 258 | 3,778 | 176 | 13,765 | ILO labor share | 1,282 | 109 | 24 | 32 |
| Saint Lucia | UMIC | Americas | 281 | 4,780 | 223 | 15,360 | ILO labor share | 2,446 | 179 | 4 | 21 |
| Samoa | LMIC | Western Pacific | 114 | 844 | 63 | 10,468 | ILO labor share | 1,148 | 194 | 83 | 45 |
| Sao Tome and Principe | LMIC | Africa | 149 | 2,087 | 157 | 5,012 | ILO labor share | 699 | 199 | 58 | 0 |
| Saudi Arabia | HIC | Eastern Mediterranean | 304 | 3,177 | 40 | 44,059 | ILO wage | 1,487,854 | 31,718 | 0 | 0 |
| Senegal | LIC | Africa | 143 | 390 | 37 | 4,078 | ILO labor share | 42,582 | 14,578 | 92 | 74 |
| Serbia | UMIC | Europe | 201 | 4,822 | 225 | 22,081 | ILO labor share | 106,044 | 7,095 | 4 | 0 |
| Sierra Leone | LIC | Africa | 148 | 334 | 32 | 2,043 | ILO labor share | 9,539 | 7,172 | 93 | 79 |
| Singapore | HIC | Western Pacific | 365 | 7,931 | 100 | 51,797 | ILO wage | 477,939 | 5,535 | 0 | 0 |
| Slovakia | HIC | Europe | 215 | 8,760 | 110 | 21,109 | OECD wage | 154,952 | 5,424 | 0 | 0 |
| Slovenia | HIC | Europe | 232 | 7,524 | 95 | 34,012 | OECD wage | 68,362 | 2,064 | 0 | 0 |
| Solomon Islands | LMIC | Western Pacific | 119 | 1,973 | 148 | 2,472 | ILO labor share | 1,499 | 603 | 61 | 0 |
| South Africa | UMIC | Africa | 130 | 4,732 | 220 | 20,574 | ILO labor share | 630,165 | 55,386 | 5 | 0 |
| Spain | HIC | Europe | 222 | 10,029 | 126 | 38,017 | OECD wage | 1,713,640 | 46,445 | 0 | 0 |
| Sri Lanka | LMIC | South-East Asia | 214 | 1,699 | 127 | 5,125 | ILO wage | 221,717 | 20,970 | 66 | 40 |
| Sudan | LMIC | Eastern Mediterranean | 267 | 1,968 | 148 | 4,752 | ILO labor share | 126,205 | 38,903 | 61 | 45 |
| Suriname | UMIC | Americas | 256 | 5,182 | 241 | 15,549 | ILO labor share | 6,344 | 559 | 0 | 6 |
| Sweden | HIC | Europe | 309 | 15,068 | 189 | 41,128 | OECD wage | 482,824 | 9,799 | 0 | 0 |
| Switzerland | HIC | Europe | 285 | 11,190 | 141 | 66,889 | OECD wage | 552,608 | 8,282 | 0 | 0 |
| Tajikistan | LMIC | Europe | 248 | 1,678 | 126 | 2,969 | ILO labor share | 21,702 | 8,454 | 66 | 49 |
| Thailand | UMIC | South-East Asia | 242 | 2,946 | 137 | 9,805 | ILO wage | 1,069,970 | 68,715 | 41 | 43 |
| Timor-Leste | LMIC | South-East Asia | 311 | 845 | 63 | 3,885 | ILO labor share | 3,899 | 1,196 | 83 | 80 |
| Togo | LIC | Africa | 138 | 396 | 37 | 1,654 | ILO labor share | 10,311 | 7,323 | 92 | 73 |
| Tonga | LMIC | Western Pacific | 113 | 3,012 | 226 | 8,156 | ILO labor share | 551 | 101 | 40 | 0 |
| Trinidad and Tobago | HIC | Americas | 250 | 6,744 | 85 | 36,205 | ILO labor share | 38,400 | 1,370 | 0 | 0 |
| Tunisia | LMIC | Eastern Mediterranean | 269 | 3,579 | 268 | 14,474 | ILO labor share | 106,637 | 11,180 | 28 | 0 |
| Turkey | UMIC | Europe | 274 | 11,006 | 513 | 23,334 | ILO labor share | 1,704,106 | 78,529 | 0 | 0 |
| Turkmenistan | UMIC | Europe | 243 | 2,605 | 121 | 16,549 | ILO labor share | 77,202 | 5,565 | 48 | 50 |
| Uganda | LIC | Africa | 167 | 513 | 49 | 2,010 | ILO labor share | 71,768 | 38,225 | 90 | 71 |
| Ukraine | LMIC | Europe | 217 | 5,402 | 405 | 6,529 | ILO wage | 336,113 | 45,154 | 0 | 0 |
| United Arab Emirates | HIC | Eastern Mediterranean | 279 | 7,072 | 89 | 31,308 | ILO labor share | 591,978 | 9,263 | 0 | 0 |
| United Kingdom | HIC | Europe | 183 | 6,921 | 87 | 42,047 | OECD wage | 2,808,960 | 65,116 | 0 | 0 |
| United Republic of Tanzania | LIC | Africa | 162 | 337 | 32 | 1,984 | ILO labor share | 106,599 | 51,483 | 93 | 80 |
| United States of America | HIC | Americas | 157 | 11,113 | 140 | 58,591 | OECD wage | 18,224,704 | 320,635 | 0 | 0 |
| Uruguay | HIC | Americas | 276 | 6,924 | 87 | 12,372 | ILO wage | 62,499 | 3,412 | 0 | 0 |
| Uzbekistan | LMIC | Europe | 248 | 2,584 | 194 | 9,614 | ILO wage | 146,683 | 31,299 | 48 | 22 |
| Vanuatu | LMIC | Western Pacific | 120 | 1,216 | 91 | 4,721 | ILO labor share | 776 | 271 | 76 | 24 |
| Viet Nam | LMIC | Western Pacific | 278 | 1,463 | 110 | 7,505 | ILO wage | 566,960 | 92,677 | 71 | 61 |
| Zambia | LMIC | Africa | 175 | 585 | 44 | 3,924 | ILO labor share | 43,739 | 15,879 | 88 | 75 |
| Zimbabwe | LIC | Africa | 257 | 1,151 | 109 | 17,206 | ILO wage | 39,048 | 13,815 | 77 | 58 |

*Note:* Approach 1 calculates unmet need as the relative difference of surgical volume to the minimum surgical volume proposed by the Lancet Commission on Global Surgery. Approach 2 calculates unmet need as the relative difference of estimated appendectomies (calculated as World Bank-income-group specific share of surgical volume) to the number of appendicitis cases.

## Additional results and sensibility checks

Figure S 8 Country- and region-wise comparison of unmet need according to approach 1 and 2


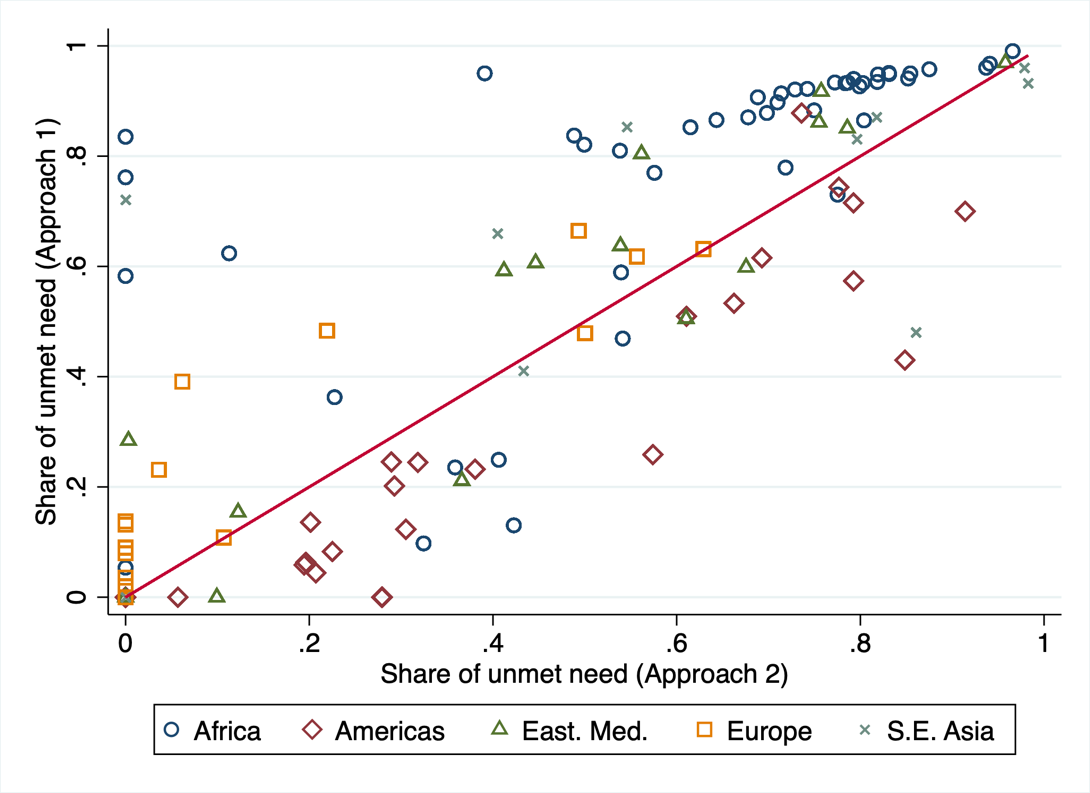


*Note:* Approach 1 calculates unmet need as the relative difference of surgical volume to the minimum surgical volume proposed by the Lancet Commission on Global Surgery. Approach 2 calculates unmet need as the relative difference of estimated appendectomies (calculated as World Bank-income-group specific share of surgical volume) to the number of appendicitis cases.

Figure S 9 Unmet need by region and income group


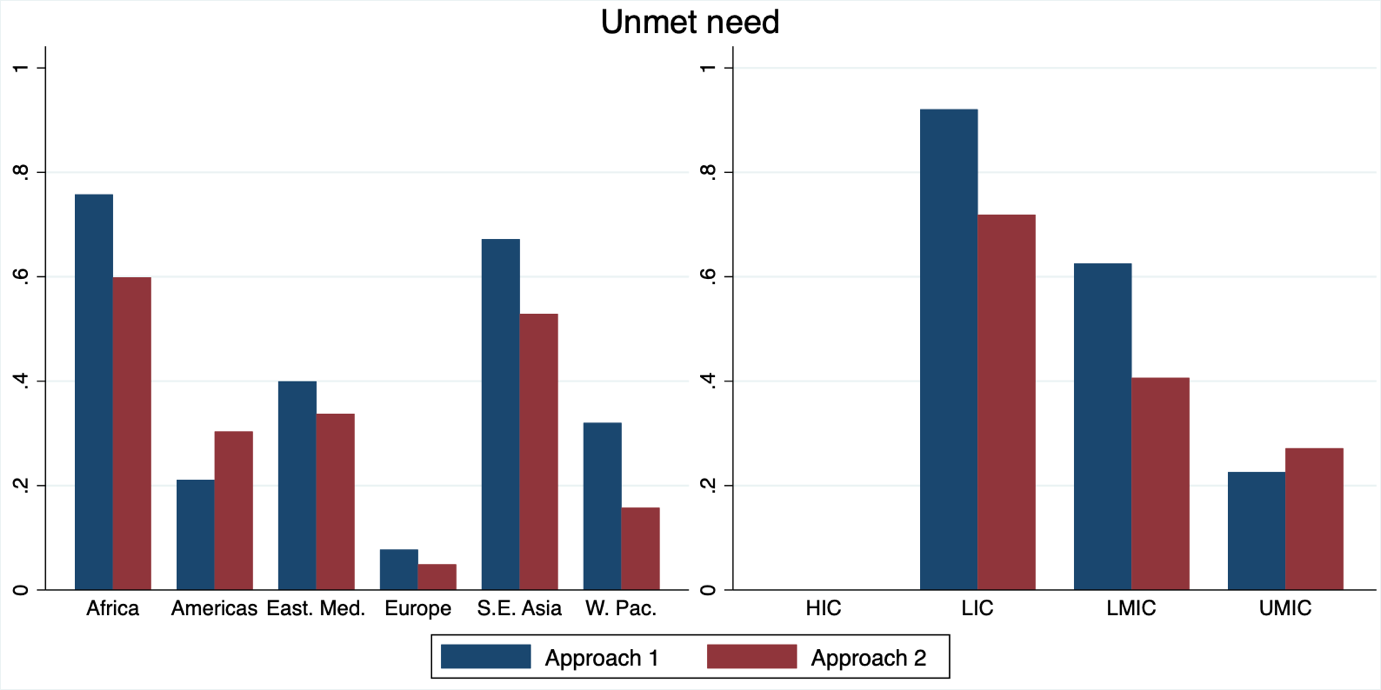


*Note:* Approach 1 calculates unmet need as the relative difference of surgical volume to the minimum surgical volume proposed by the Lancet Commission on Global Surgery. Approach 2 calculates unmet need as the relative difference of estimated appendectomies (calculated as World Bank-income-group specific share of surgical volume) to the number of appendicitis cases.

Figure S 10 Expected outcomes per case of appendicitis when not providing care to local standards


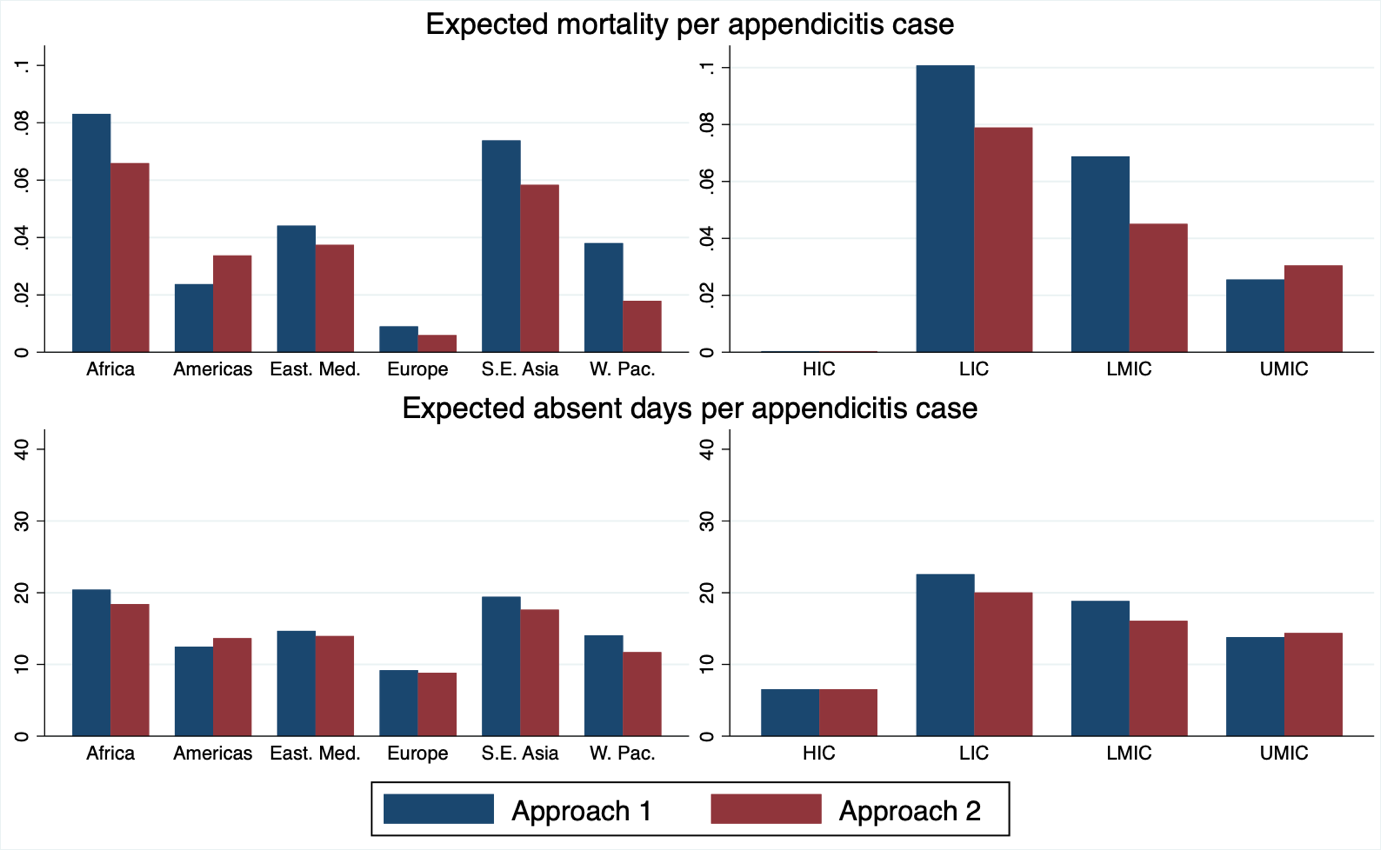


*Note:* Approach 1 calculates unmet need as the relative difference of surgical volume to the minimum surgical volume proposed by the Lancet Commission on Global Surgery. Approach 2 calculates unmet need as the relative difference of estimated appendectomies (calculated as World Bank-income-group specific share of surgical volume) to the number of appendicitis cases.

Table S 10 Estimates excess mortality due to unmet surgical need

|  | **Africa** | **Americas** | **Eastern Med.** | **Europe** | **South-East Asia** | **Western Pacific** | **World** |
| --- | --- | --- | --- | --- | --- | --- | --- |
| Countries | 44 | 29 | 18 | 50 | 10 | 19 | 170 |
| No surgery to local standard (1) | 8,299 | 2,663 | 3,841 | 888 | 7,277 | 4,356 | 4,185 |
|  | (2,826) | (3,113) | (3,868) | (1,968) | (3,243) | (3,899) | (4,129) |
| No surgery to local standard (2) | 6,522 | 3,642 | 3,366 | 564 | 6,301 | 2,204 | 3,448 |
|  | (2,943) | (3,532) | (3,418) | (1,643) | (4,001) | (3,071) | (3,697) |
| No surgery to high. standard (1) | 8,372 | 2,718 | 3,889 | 917 | 7,349 | 4,405 | 4,237 |
|  | (2,826) | (3,128) | (3,894) | (1,988) | (3,243) | (3,926) | (4,150) |
| No surgery to high. standard (2) | 6,594 | 3,697 | 3,414 | 593 | 6,373 | 2,254 | 3,500 |
|  | (2,943) | (3,551) | (3,443) | (1,659) | (4,001) | (3,089) | (3,717) |

Mortality per 100,000 cases of appendicitis by region. Depicted as mean (standard deviation). Approach 1 calculates unmet need as the relative difference of surgical volume to the minimum surgical volume proposed by the Lancet Commission on Global Surgery. Approach 2 calculates unmet need as the relative difference of estimated appendectomies (calculated as World Bank-income-group specific share of surgical volume) to the number of appendicitis cases.

Figure S 11 Map of mortality-related income losses due to not providing surgery to local standards


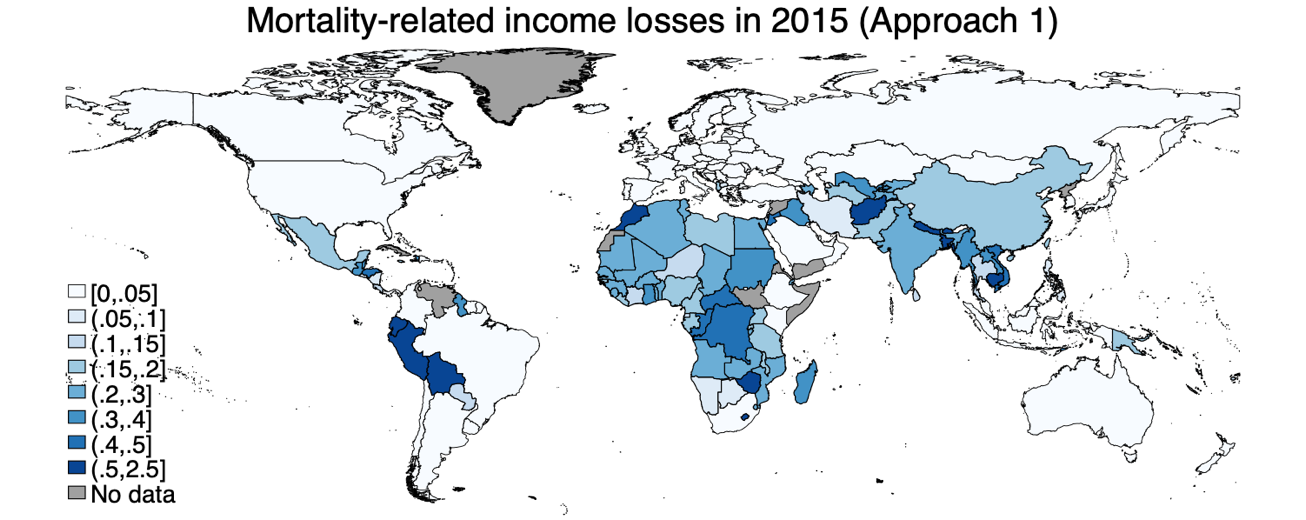


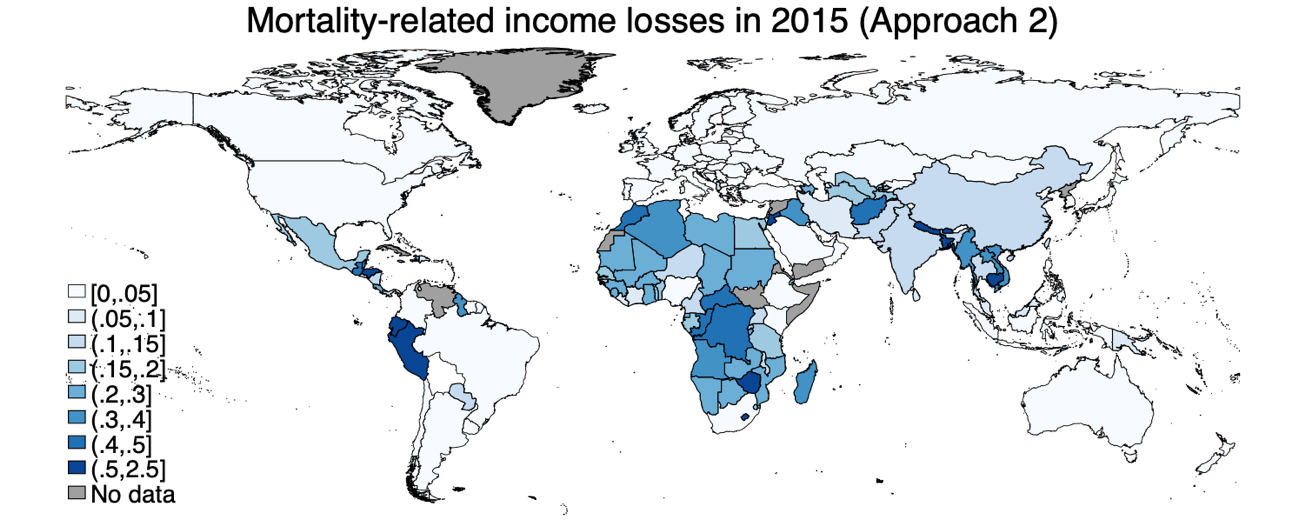


*Note:* Approach 1 calculates unmet need as the relative difference of surgical volume to the minimum surgical volume proposed by the Lancet Commission on Global Surgery. Approach 2 calculates unmet need as the relative difference of estimated appendectomies (calculated as World Bank-income-group specific share of surgical volume) to the number of appendicitis cases.

Figure S 12 Map of absenteeism-related income losses due to not providing surgery to local standards


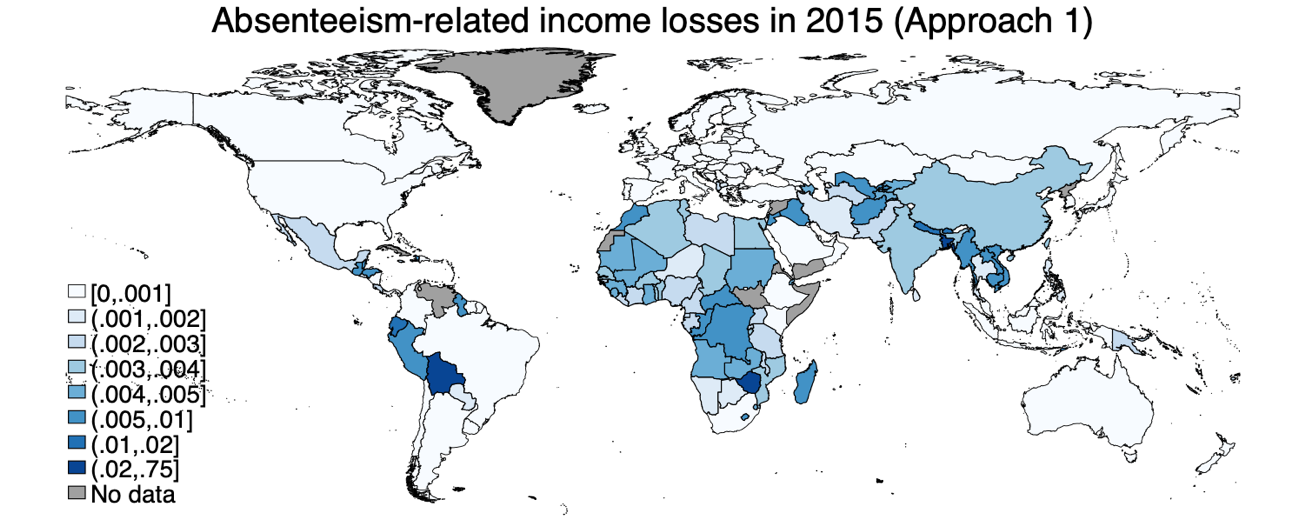


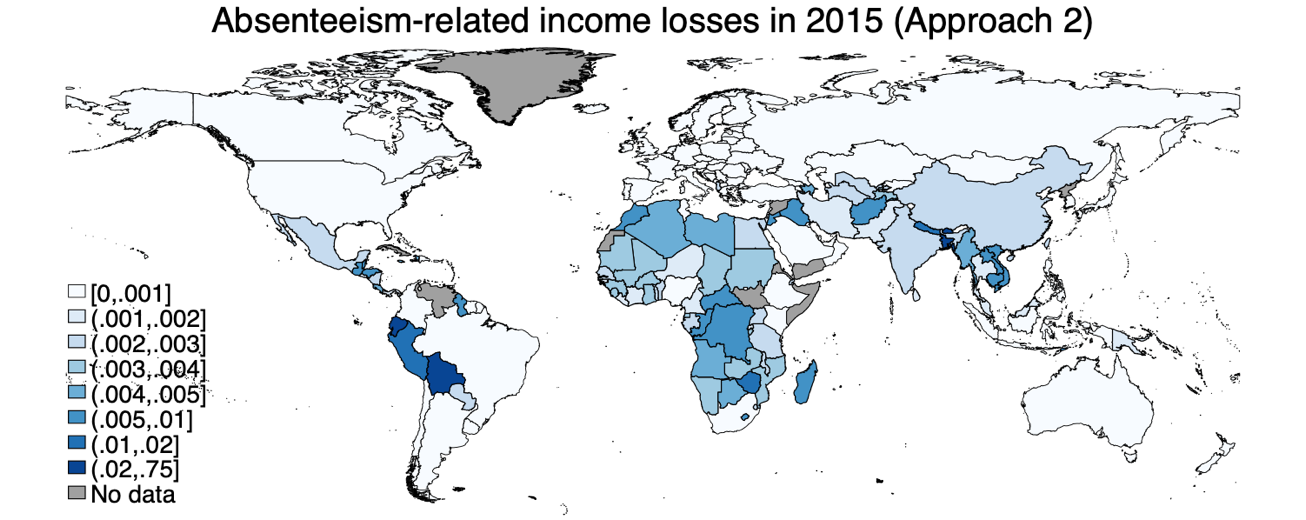


*Note:* Approach 1 calculates unmet need as the relative difference of surgical volume to the minimum surgical volume proposed by the Lancet Commission on Global Surgery. Approach 2 calculates unmet need as the relative difference of estimated appendectomies (calculated as World Bank-income-group specific share of surgical volume) to the number of appendicitis cases.

Figure S 13 Total economic burden of not providing surgery to local standard by gender


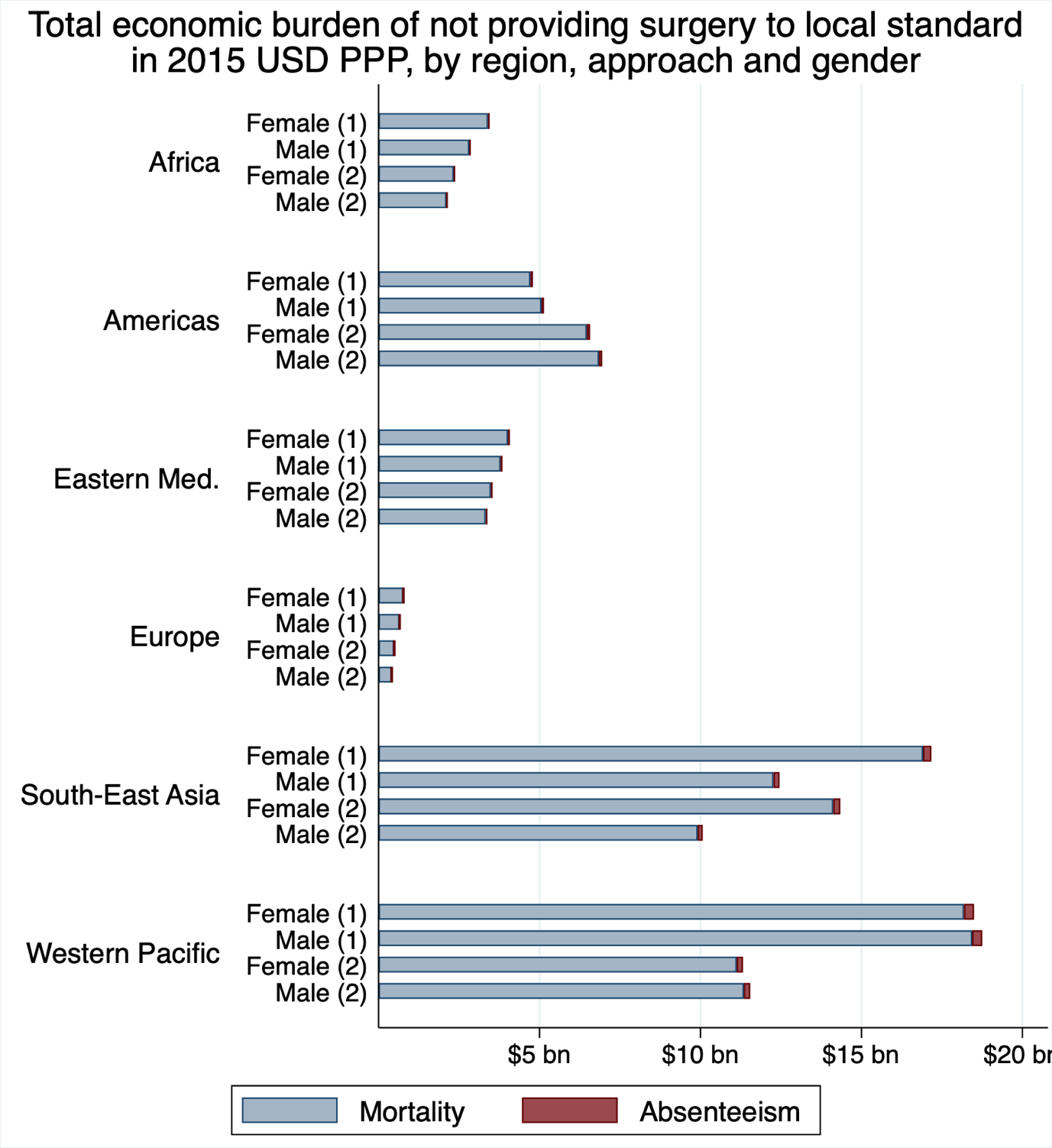


*Note:* All gender differences origin from the gender differences in incidence rates in the GBD estimates. Approach 1 calculates unmet need as the relative difference of surgical volume to the minimum surgical volume proposed by the Lancet Commission on Global Surgery. Approach 2 calculates unmet need as the relative difference of estimated appendectomies (calculated as World Bank-income-group specific share of surgical volume) to the number of appendicitis cases.

Figure S 14 Total economic burden of not providing surgery to local standard by gender


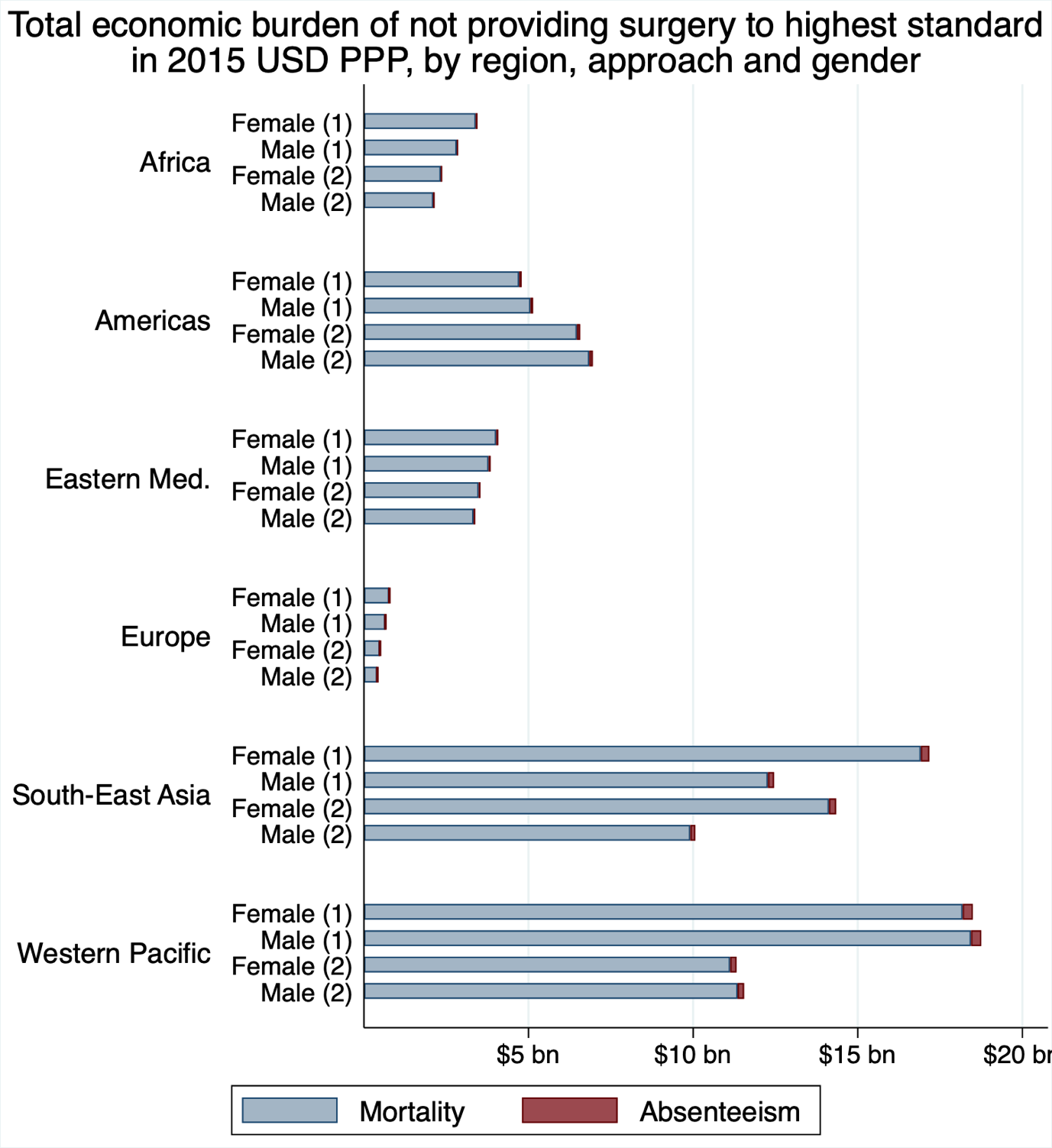


*Note:* All gender differences origin from the gender differences in incidence rates in the GBD estimates. Approach 1 calculates unmet need as the relative difference of surgical volume to the minimum surgical volume proposed by the Lancet Commission on Global Surgery. Approach 2 calculates unmet need as the relative difference of estimated appendectomies (calculated as World Bank-income-group specific share of surgical volume) to the number of appendicitis cases.

Figure S 15 Avoidable economic burden in relation to indirect economic burden of appendicitis


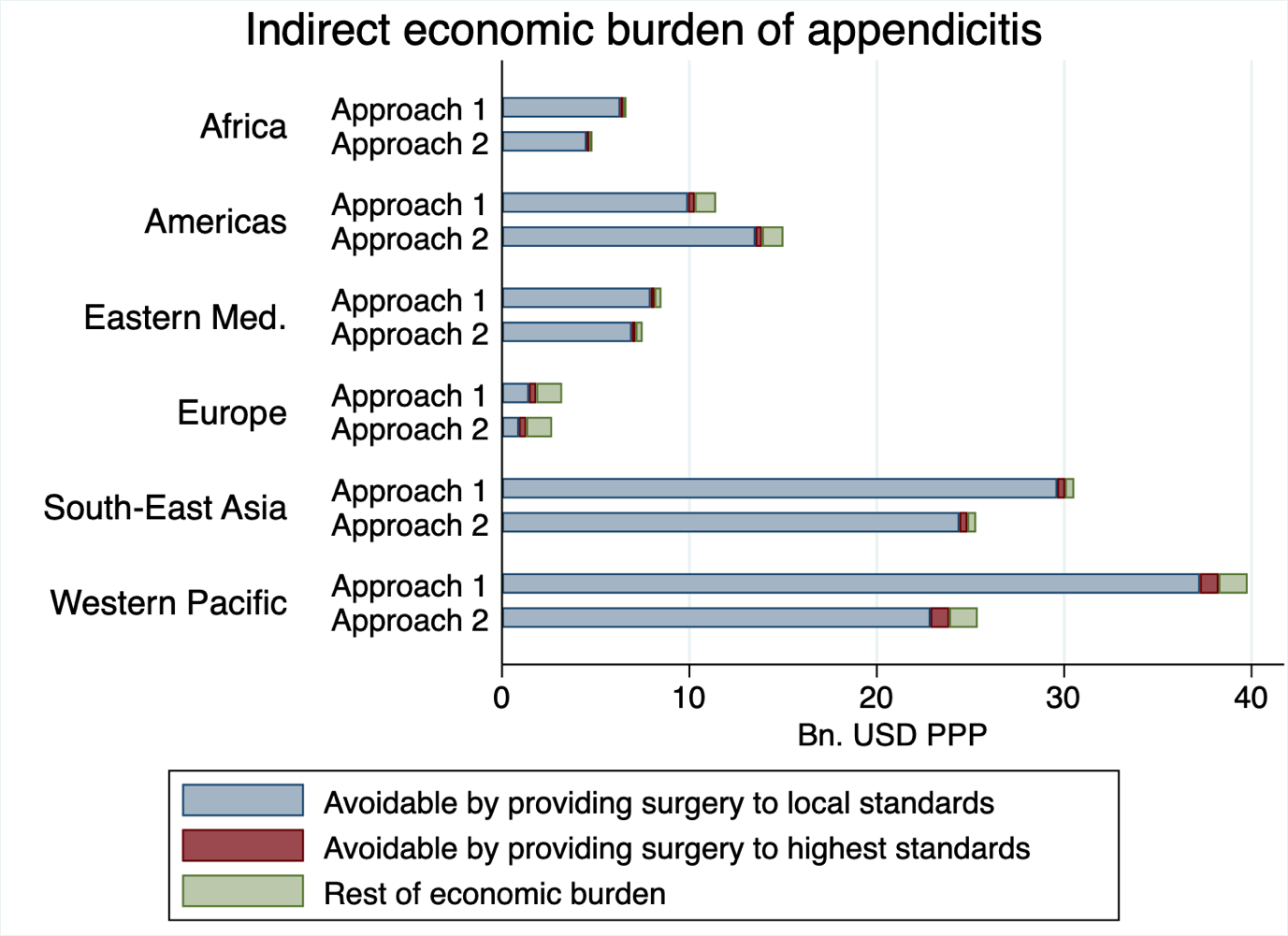


*Note:* The rest of the burden would remain if all people with appendectomies in low- and middle-income countries would retrieve surgery to highest standards. They occur due to adverse events and are modifiable by (global) medical/technological progress in the treatment of appendicitis. Approach 1 calculates unmet need as the relative difference of surgical volume to the minimum surgical volume proposed by the Lancet Commission on Global Surgery. Approach 2 calculates unmet need as the relative difference of estimated appendectomies (calculated as World Bank-income-group specific share of surgical volume) to the number of appendicitis cases.

Figure S 16 Total economic burden in % of GDP by approach


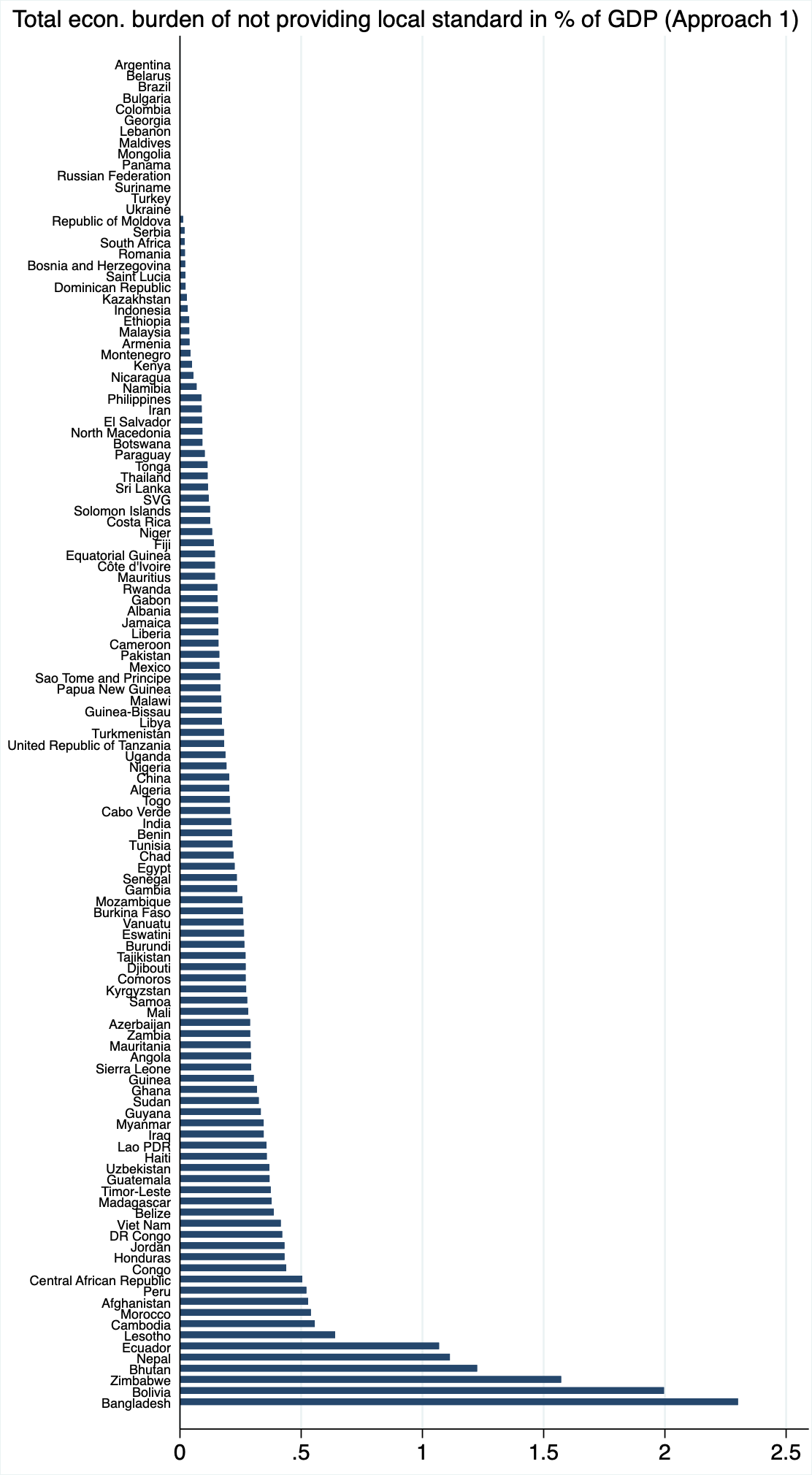


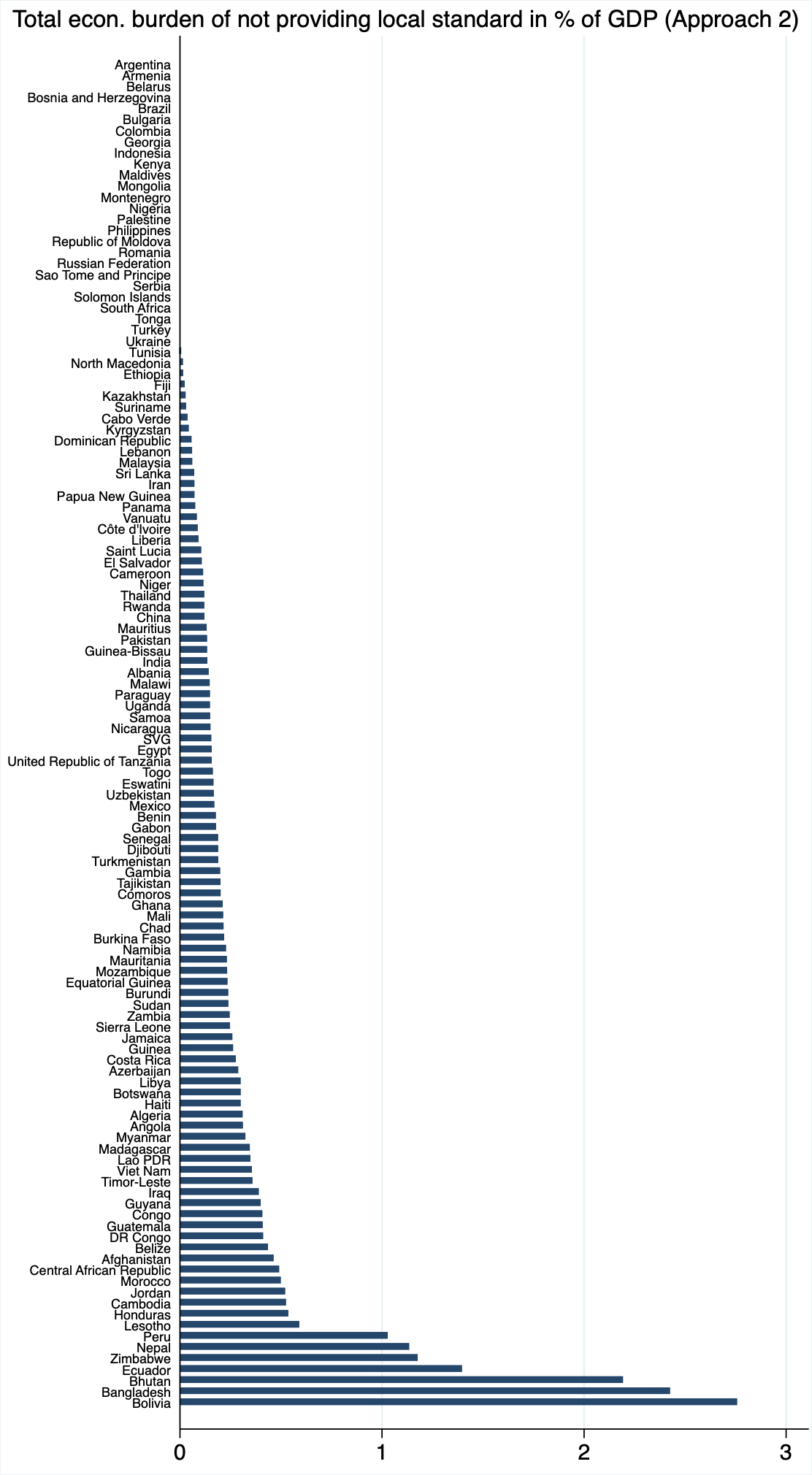


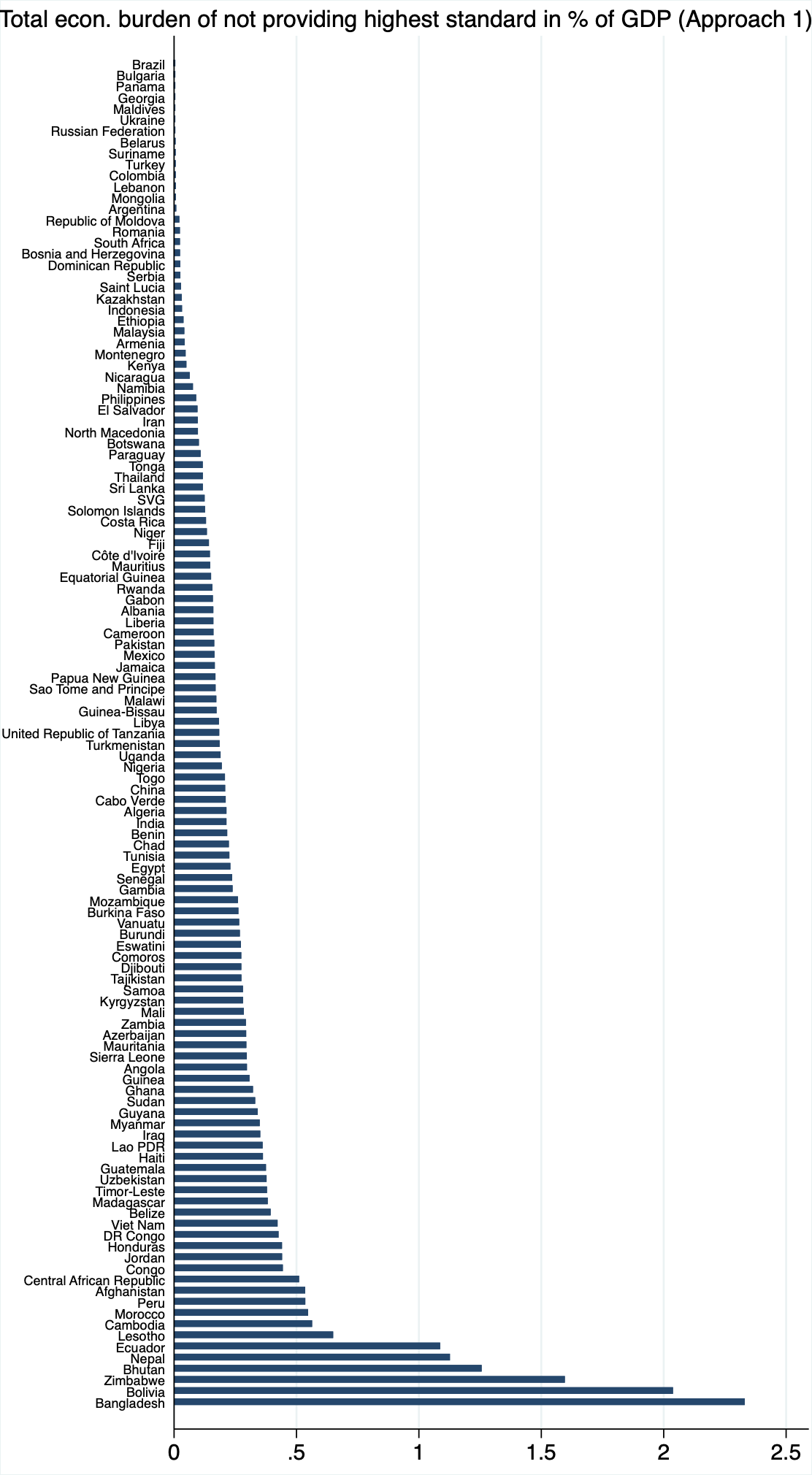


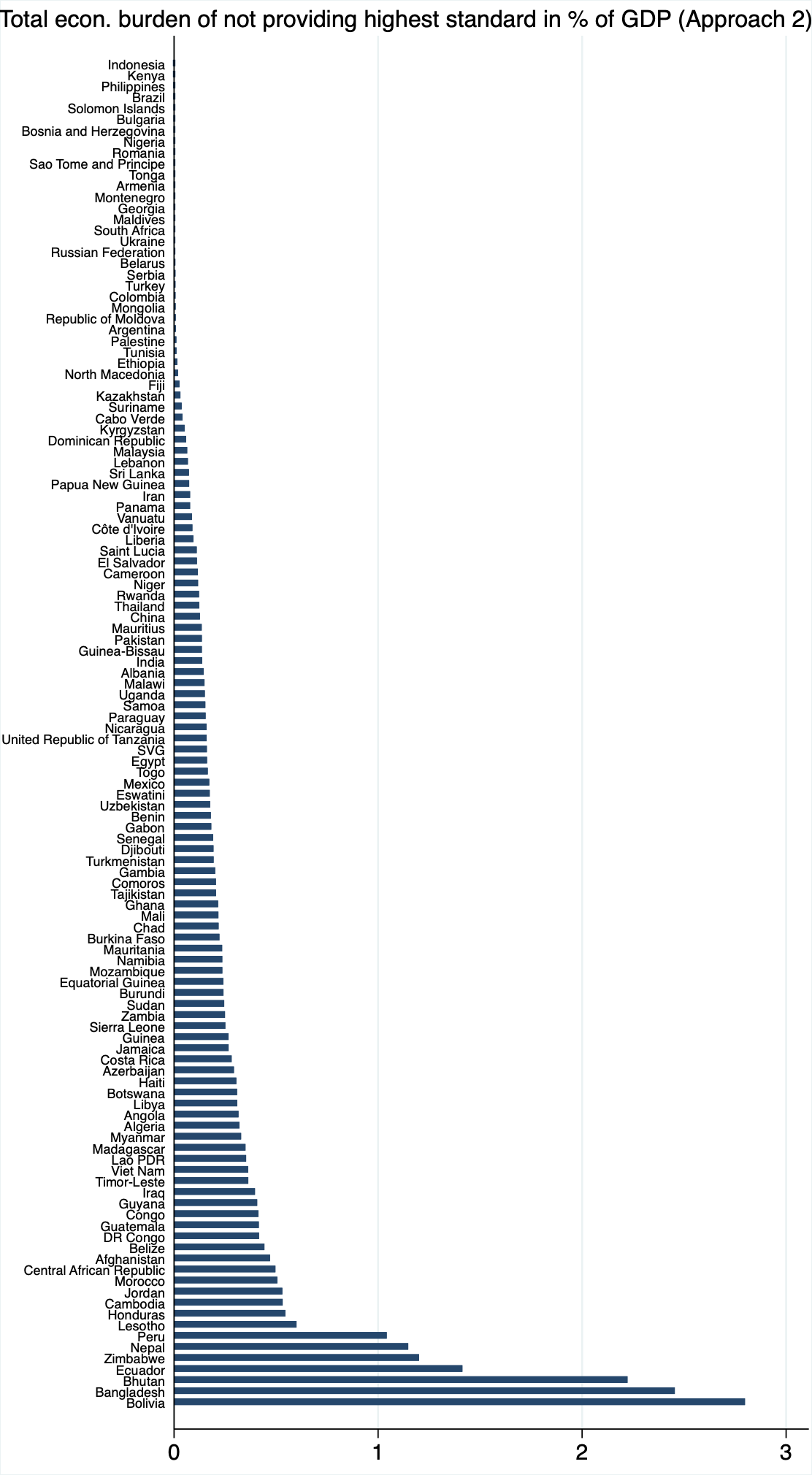


Table S 11 Country-wise economic burden as % of GDP

| Country | Income group | WHO region | Mortality-related burden in % of GDP | | | | Absenteeism-related burden in % of GDP | | | | Total economic burden in % of GDP | | | |
| --- | --- | --- | --- | --- | --- | --- | --- | --- | --- | --- | --- | --- | --- | --- |
|  |  |  | Local standard | | Highest standard | | Local standard | | Highest standard | | Local standard | | Highest standard | |
|  |  |  | Appr. 1 | Appr. 2 | Appr. 1 | Appr. 2 | Appr. 1 | Appr. 2 | Appr. 1 | Appr. 2 | Appr. 1 | Appr. 2 | Appr. 1 | Appr. 2 |
| Afghanistan | LIC | Eastern Mediterranean | 0.5208 | 0.4563 | 0.5249 | 0.4604 | 0.0078 | 0.0069 | 0.0109 | 0.0101 | 0.5286 | 0.4632 | 0.5358 | 0.4705 |
| Albania | UMIC | Europe | 0.1549 | 0.1396 | 0.1566 | 0.1413 | 0.0025 | 0.0023 | 0.0040 | 0.0037 | 0.1574 | 0.1419 | 0.1605 | 0.1450 |
| Algeria | UMIC | Africa | 0.2001 | 0.3051 | 0.2058 | 0.3108 | 0.0033 | 0.0050 | 0.0082 | 0.0098 | 0.2034 | 0.3100 | 0.2140 | 0.3205 |
| Angola | UMIC | Africa | 0.2887 | 0.3065 | 0.2913 | 0.3091 | 0.0044 | 0.0046 | 0.0065 | 0.0067 | 0.2931 | 0.3111 | 0.2978 | 0.3158 |
| Argentina | UMIC | Americas | 0.0000 | 0.0000 | 0.0047 | 0.0047 | 0.0000 | 0.0000 | 0.0043 | 0.0043 | 0.0000 | 0.0000 | 0.0090 | 0.0090 |
| Armenia | LMIC | Europe | 0.0388 | 0.0000 | 0.0408 | 0.0020 | 0.0007 | 0.0000 | 0.0024 | 0.0018 | 0.0395 | 0.0000 | 0.0432 | 0.0037 |
| Australia | HIC | Western Pacific | 0.0000 | 0.0000 | 0.0000 | 0.0000 | 0.0000 | 0.0000 | 0.0000 | 0.0000 | 0.0000 | 0.0000 | 0.0000 | 0.0000 |
| Austria | HIC | Europe | 0.0000 | 0.0000 | 0.0000 | 0.0000 | 0.0000 | 0.0000 | 0.0000 | 0.0000 | 0.0000 | 0.0000 | 0.0000 | 0.0000 |
| Azerbaijan | UMIC | Europe | 0.2846 | 0.2836 | 0.2877 | 0.2866 | 0.0045 | 0.0045 | 0.0070 | 0.0069 | 0.2891 | 0.2881 | 0.2946 | 0.2936 |
| Bahamas | HIC | Americas | 0.0000 | 0.0000 | 0.0000 | 0.0000 | 0.0000 | 0.0000 | 0.0000 | 0.0000 | 0.0000 | 0.0000 | 0.0000 | 0.0000 |
| Bahrain | HIC | Eastern Mediterranean | 0.0000 | 0.0000 | 0.0000 | 0.0000 | 0.0000 | 0.0000 | 0.0000 | 0.0000 | 0.0000 | 0.0000 | 0.0000 | 0.0000 |
| Bangladesh | LMIC | South-East Asia | 2.2667 | 2.3887 | 2.2830 | 2.4050 | 0.0348 | 0.0364 | 0.0477 | 0.0493 | 2.3015 | 2.4251 | 2.3308 | 2.4543 |
| Barbados | HIC | Americas | 0.0000 | 0.0000 | 0.0000 | 0.0000 | 0.0000 | 0.0000 | 0.0000 | 0.0000 | 0.0000 | 0.0000 | 0.0000 | 0.0000 |
| Belarus | UMIC | Europe | 0.0000 | 0.0000 | 0.0032 | 0.0032 | 0.0000 | 0.0000 | 0.0030 | 0.0030 | 0.0000 | 0.0000 | 0.0063 | 0.0063 |
| Belgium | HIC | Europe | 0.0000 | 0.0000 | 0.0000 | 0.0000 | 0.0000 | 0.0000 | 0.0000 | 0.0000 | 0.0000 | 0.0000 | 0.0000 | 0.0000 |
| Belize | UMIC | Americas | 0.3811 | 0.4289 | 0.3852 | 0.4330 | 0.0059 | 0.0066 | 0.0093 | 0.0099 | 0.3870 | 0.4355 | 0.3945 | 0.4430 |
| Benin | LIC | Africa | 0.2113 | 0.1748 | 0.2128 | 0.1763 | 0.0031 | 0.0026 | 0.0043 | 0.0038 | 0.2144 | 0.1774 | 0.2171 | 0.1801 |
| Bhutan | LMIC | South-East Asia | 1.2066 | 2.1600 | 1.2235 | 2.1768 | 0.0190 | 0.0326 | 0.0329 | 0.0458 | 1.2257 | 2.1926 | 1.2563 | 2.2226 |
| Bolivia | LMIC | Americas | 1.9653 | 2.7156 | 1.9882 | 2.7386 | 0.0309 | 0.0416 | 0.0497 | 0.0599 | 1.9962 | 2.7572 | 2.0379 | 2.7984 |
| Bosnia and Herzegovina | UMIC | Europe | 0.0211 | 0.0000 | 0.0227 | 0.0016 | 0.0004 | 0.0000 | 0.0018 | 0.0015 | 0.0215 | 0.0000 | 0.0245 | 0.0030 |
| Botswana | UMIC | Africa | 0.0911 | 0.2955 | 0.0958 | 0.3001 | 0.0015 | 0.0047 | 0.0055 | 0.0086 | 0.0927 | 0.3002 | 0.1014 | 0.3088 |
| Brazil | UMIC | Americas | 0.0000 | 0.0000 | 0.0010 | 0.0010 | 0.0000 | 0.0000 | 0.0008 | 0.0008 | 0.0000 | 0.0000 | 0.0018 | 0.0018 |
| Brunei Darussalam | HIC | Western Pacific | 0.0000 | 0.0000 | 0.0000 | 0.0000 | 0.0000 | 0.0000 | 0.0000 | 0.0000 | 0.0000 | 0.0000 | 0.0000 | 0.0000 |
| Bulgaria | UMIC | Europe | 0.0000 | 0.0000 | 0.0013 | 0.0013 | 0.0000 | 0.0000 | 0.0012 | 0.0012 | 0.0000 | 0.0000 | 0.0025 | 0.0025 |
| Burkina Faso | LIC | Africa | 0.2558 | 0.2157 | 0.2577 | 0.2176 | 0.0038 | 0.0032 | 0.0052 | 0.0047 | 0.2596 | 0.2190 | 0.2628 | 0.2222 |
| Burundi | LIC | Africa | 0.2617 | 0.2353 | 0.2635 | 0.2371 | 0.0039 | 0.0035 | 0.0053 | 0.0050 | 0.2655 | 0.2388 | 0.2688 | 0.2421 |
| Cabo Verde | LMIC | Africa | 0.2037 | 0.0368 | 0.2059 | 0.0390 | 0.0032 | 0.0006 | 0.0049 | 0.0025 | 0.2069 | 0.0374 | 0.2108 | 0.0415 |
| Cambodia | LMIC | Western Pacific | 0.5477 | 0.5165 | 0.5521 | 0.5209 | 0.0084 | 0.0080 | 0.0119 | 0.0115 | 0.5561 | 0.5245 | 0.5640 | 0.5324 |
| Cameroon | LMIC | Africa | 0.1565 | 0.1129 | 0.1577 | 0.1141 | 0.0023 | 0.0017 | 0.0033 | 0.0027 | 0.1588 | 0.1146 | 0.1610 | 0.1168 |
| Canada | HIC | Americas | 0.0000 | 0.0000 | 0.0000 | 0.0000 | 0.0000 | 0.0000 | 0.0000 | 0.0000 | 0.0000 | 0.0000 | 0.0000 | 0.0000 |
| Central African Republic | LIC | Africa | 0.4972 | 0.4834 | 0.5006 | 0.4869 | 0.0074 | 0.0073 | 0.0101 | 0.0099 | 0.5046 | 0.4907 | 0.5107 | 0.4968 |
| Chad | LIC | Africa | 0.2181 | 0.2126 | 0.2196 | 0.2141 | 0.0032 | 0.0031 | 0.0043 | 0.0042 | 0.2213 | 0.2158 | 0.2239 | 0.2184 |
| Chile | HIC | Americas | 0.0000 | 0.0000 | 0.0000 | 0.0000 | 0.0000 | 0.0000 | 0.0000 | 0.0000 | 0.0000 | 0.0000 | 0.0000 | 0.0000 |
| China | UMIC | Western Pacific | 0.1999 | 0.1192 | 0.2028 | 0.1221 | 0.0034 | 0.0021 | 0.0060 | 0.0047 | 0.2033 | 0.1213 | 0.2088 | 0.1268 |
| Colombia | UMIC | Americas | 0.0000 | 0.0000 | 0.0036 | 0.0036 | 0.0000 | 0.0000 | 0.0033 | 0.0033 | 0.0000 | 0.0000 | 0.0069 | 0.0069 |
| Comoros | LIC | Africa | 0.2668 | 0.1983 | 0.2689 | 0.2004 | 0.0041 | 0.0031 | 0.0057 | 0.0048 | 0.2709 | 0.2014 | 0.2746 | 0.2052 |
| Congo | LMIC | Africa | 0.4318 | 0.4014 | 0.4351 | 0.4047 | 0.0065 | 0.0061 | 0.0092 | 0.0088 | 0.4383 | 0.4075 | 0.4443 | 0.4135 |
| Costa Rica | UMIC | Americas | 0.1225 | 0.2720 | 0.1257 | 0.2752 | 0.0021 | 0.0044 | 0.0049 | 0.0071 | 0.1246 | 0.2764 | 0.1305 | 0.2823 |
| Croatia | HIC | Europe | 0.0000 | 0.0000 | 0.0000 | 0.0000 | 0.0000 | 0.0000 | 0.0000 | 0.0000 | 0.0000 | 0.0000 | 0.0000 | 0.0000 |
| Cyprus | HIC | Europe | 0.0000 | 0.0000 | 0.0000 | 0.0000 | 0.0000 | 0.0000 | 0.0000 | 0.0000 | 0.0000 | 0.0000 | 0.0000 | 0.0000 |
| Czechia | HIC | Europe | 0.0000 | 0.0000 | 0.0000 | 0.0000 | 0.0000 | 0.0000 | 0.0000 | 0.0000 | 0.0000 | 0.0000 | 0.0000 | 0.0000 |
| Côte d'Ivoire | LMIC | Africa | 0.1421 | 0.0865 | 0.1433 | 0.0876 | 0.0021 | 0.0013 | 0.0030 | 0.0023 | 0.1443 | 0.0878 | 0.1463 | 0.0899 |
| DR Congo | LIC | Africa | 0.4155 | 0.4054 | 0.4184 | 0.4083 | 0.0062 | 0.0060 | 0.0084 | 0.0083 | 0.4217 | 0.4114 | 0.4268 | 0.4166 |
| Denmark | HIC | Europe | 0.0000 | 0.0000 | 0.0000 | 0.0000 | 0.0000 | 0.0000 | 0.0000 | 0.0000 | 0.0000 | 0.0000 | 0.0000 | 0.0000 |
| Djibouti | LMIC | Eastern Mediterranean | 0.2668 | 0.1863 | 0.2690 | 0.1886 | 0.0041 | 0.0029 | 0.0058 | 0.0047 | 0.2709 | 0.1893 | 0.2748 | 0.1933 |
| Dominican Republic | UMIC | Americas | 0.0227 | 0.0563 | 0.0239 | 0.0575 | 0.0004 | 0.0009 | 0.0014 | 0.0020 | 0.0231 | 0.0572 | 0.0254 | 0.0594 |
| Ecuador | UMIC | Americas | 1.0521 | 1.3742 | 1.0622 | 1.3843 | 0.0165 | 0.0211 | 0.0248 | 0.0291 | 1.0687 | 1.3953 | 1.0870 | 1.4134 |
| Egypt | LMIC | Eastern Mediterranean | 0.2221 | 0.1545 | 0.2246 | 0.1570 | 0.0035 | 0.0025 | 0.0055 | 0.0046 | 0.2256 | 0.1570 | 0.2301 | 0.1616 |
| El Salvador | LMIC | Americas | 0.0898 | 0.1059 | 0.0922 | 0.1083 | 0.0015 | 0.0017 | 0.0036 | 0.0038 | 0.0913 | 0.1076 | 0.0958 | 0.1122 |
| Equatorial Guinea | UMIC | Africa | 0.1420 | 0.2315 | 0.1458 | 0.2353 | 0.0023 | 0.0036 | 0.0054 | 0.0068 | 0.1442 | 0.2351 | 0.1512 | 0.2420 |
| Estonia | HIC | Europe | 0.0000 | 0.0000 | 0.0000 | 0.0000 | 0.0000 | 0.0000 | 0.0000 | 0.0000 | 0.0000 | 0.0000 | 0.0000 | 0.0000 |
| Eswatini | LMIC | Africa | 0.2602 | 0.1632 | 0.2650 | 0.1680 | 0.0041 | 0.0026 | 0.0081 | 0.0067 | 0.2643 | 0.1658 | 0.2731 | 0.1747 |
| Ethiopia | LIC | Africa | 0.0375 | 0.0154 | 0.0378 | 0.0157 | 0.0005 | 0.0002 | 0.0007 | 0.0005 | 0.0381 | 0.0157 | 0.0385 | 0.0162 |
| Fiji | UMIC | Western Pacific | 0.1370 | 0.0228 | 0.1387 | 0.0245 | 0.0022 | 0.0004 | 0.0037 | 0.0019 | 0.1393 | 0.0232 | 0.1424 | 0.0264 |
| Finland | HIC | Europe | 0.0000 | 0.0000 | 0.0000 | 0.0000 | 0.0000 | 0.0000 | 0.0000 | 0.0000 | 0.0000 | 0.0000 | 0.0000 | 0.0000 |
| France | HIC | Europe | 0.0000 | 0.0000 | 0.0000 | 0.0000 | 0.0000 | 0.0000 | 0.0000 | 0.0000 | 0.0000 | 0.0000 | 0.0000 | 0.0000 |
| Gabon | UMIC | Africa | 0.1523 | 0.1757 | 0.1544 | 0.1778 | 0.0024 | 0.0028 | 0.0042 | 0.0045 | 0.1547 | 0.1784 | 0.1586 | 0.1824 |
| Gambia | LIC | Africa | 0.2327 | 0.1956 | 0.2344 | 0.1973 | 0.0034 | 0.0029 | 0.0047 | 0.0042 | 0.2361 | 0.1986 | 0.2391 | 0.2016 |
| Georgia | UMIC | Europe | 0.0000 | 0.0000 | 0.0022 | 0.0022 | 0.0000 | 0.0000 | 0.0020 | 0.0020 | 0.0000 | 0.0000 | 0.0042 | 0.0042 |
| Germany | HIC | Europe | 0.0000 | 0.0000 | 0.0000 | 0.0000 | 0.0000 | 0.0000 | 0.0000 | 0.0000 | 0.0000 | 0.0000 | 0.0000 | 0.0000 |
| Ghana | LMIC | Africa | 0.3130 | 0.2081 | 0.3156 | 0.2106 | 0.0047 | 0.0032 | 0.0067 | 0.0053 | 0.3177 | 0.2113 | 0.3223 | 0.2160 |
| Greece | HIC | Europe | 0.0000 | 0.0000 | 0.0000 | 0.0000 | 0.0000 | 0.0000 | 0.0000 | 0.0000 | 0.0000 | 0.0000 | 0.0000 | 0.0000 |
| Guatemala | LMIC | Americas | 0.3638 | 0.4030 | 0.3672 | 0.4064 | 0.0056 | 0.0061 | 0.0083 | 0.0088 | 0.3694 | 0.4091 | 0.3755 | 0.4152 |
| Guinea | LIC | Africa | 0.2999 | 0.2591 | 0.3020 | 0.2612 | 0.0044 | 0.0039 | 0.0060 | 0.0055 | 0.3043 | 0.2630 | 0.3081 | 0.2667 |
| Guinea-Bissau | LIC | Africa | 0.1690 | 0.1320 | 0.1702 | 0.1333 | 0.0025 | 0.0020 | 0.0034 | 0.0030 | 0.1715 | 0.1340 | 0.1737 | 0.1362 |
| Guyana | UMIC | Americas | 0.3282 | 0.3934 | 0.3325 | 0.3977 | 0.0052 | 0.0062 | 0.0088 | 0.0098 | 0.3335 | 0.3996 | 0.3414 | 0.4075 |
| Haiti | LIC | Americas | 0.3529 | 0.2957 | 0.3556 | 0.2984 | 0.0053 | 0.0045 | 0.0074 | 0.0067 | 0.3582 | 0.3003 | 0.3630 | 0.3051 |
| Honduras | LMIC | Americas | 0.4245 | 0.5275 | 0.4299 | 0.5328 | 0.0067 | 0.0082 | 0.0111 | 0.0126 | 0.4313 | 0.5357 | 0.4410 | 0.5454 |
| Hungary | HIC | Europe | 0.0000 | 0.0000 | 0.0000 | 0.0000 | 0.0000 | 0.0000 | 0.0000 | 0.0000 | 0.0000 | 0.0000 | 0.0000 | 0.0000 |
| Iceland | HIC | Europe | 0.0000 | 0.0000 | 0.0000 | 0.0000 | 0.0000 | 0.0000 | 0.0000 | 0.0000 | 0.0000 | 0.0000 | 0.0000 | 0.0000 |
| India | LMIC | South-East Asia | 0.2081 | 0.1331 | 0.2097 | 0.1347 | 0.0031 | 0.0021 | 0.0044 | 0.0034 | 0.2112 | 0.1352 | 0.2141 | 0.1381 |
| Indonesia | LMIC | South-East Asia | 0.0315 | 0.0000 | 0.0317 | 0.0003 | 0.0005 | 0.0000 | 0.0007 | 0.0003 | 0.0319 | 0.0000 | 0.0325 | 0.0005 |
| Iran | UMIC | Eastern Mediterranean | 0.0882 | 0.0702 | 0.0921 | 0.0740 | 0.0015 | 0.0012 | 0.0048 | 0.0045 | 0.0897 | 0.0714 | 0.0968 | 0.0785 |
| Iraq | UMIC | Eastern Mediterranean | 0.3400 | 0.3838 | 0.3438 | 0.3876 | 0.0053 | 0.0059 | 0.0084 | 0.0090 | 0.3453 | 0.3898 | 0.3522 | 0.3966 |
| Ireland | HIC | Europe | 0.0000 | 0.0000 | 0.0000 | 0.0000 | 0.0000 | 0.0000 | 0.0000 | 0.0000 | 0.0000 | 0.0000 | 0.0000 | 0.0000 |
| Israel | HIC | Europe | 0.0000 | 0.0000 | 0.0000 | 0.0000 | 0.0000 | 0.0000 | 0.0000 | 0.0000 | 0.0000 | 0.0000 | 0.0000 | 0.0000 |
| Italy | HIC | Europe | 0.0000 | 0.0000 | 0.0000 | 0.0000 | 0.0000 | 0.0000 | 0.0000 | 0.0000 | 0.0000 | 0.0000 | 0.0000 | 0.0000 |
| Jamaica | UMIC | Americas | 0.1556 | 0.2550 | 0.1601 | 0.2595 | 0.0026 | 0.0041 | 0.0064 | 0.0079 | 0.1582 | 0.2591 | 0.1665 | 0.2674 |
| Japan | HIC | Western Pacific | 0.0000 | 0.0000 | 0.0000 | 0.0000 | 0.0000 | 0.0000 | 0.0000 | 0.0000 | 0.0000 | 0.0000 | 0.0000 | 0.0000 |
| Jordan | UMIC | Eastern Mediterranean | 0.4244 | 0.5124 | 0.4300 | 0.5180 | 0.0067 | 0.0080 | 0.0114 | 0.0126 | 0.4311 | 0.5205 | 0.4414 | 0.5307 |
| Kazakhstan | UMIC | Europe | 0.0279 | 0.0275 | 0.0296 | 0.0292 | 0.0005 | 0.0005 | 0.0020 | 0.0020 | 0.0283 | 0.0280 | 0.0316 | 0.0312 |
| Kenya | LMIC | Africa | 0.0490 | 0.0000 | 0.0494 | 0.0004 | 0.0007 | 0.0000 | 0.0010 | 0.0003 | 0.0498 | 0.0000 | 0.0505 | 0.0007 |
| Kuwait | HIC | Eastern Mediterranean | 0.0000 | 0.0000 | 0.0000 | 0.0000 | 0.0000 | 0.0000 | 0.0000 | 0.0000 | 0.0000 | 0.0000 | 0.0000 | 0.0000 |
| Kyrgyzstan | LMIC | Europe | 0.2688 | 0.0424 | 0.2734 | 0.0471 | 0.0043 | 0.0007 | 0.0081 | 0.0047 | 0.2731 | 0.0432 | 0.2815 | 0.0517 |
| Lao PDR | LMIC | Western Pacific | 0.3517 | 0.3433 | 0.3543 | 0.3460 | 0.0053 | 0.0052 | 0.0074 | 0.0073 | 0.3570 | 0.3485 | 0.3617 | 0.3533 |
| Latvia | HIC | Europe | 0.0000 | 0.0000 | 0.0000 | 0.0000 | 0.0000 | 0.0000 | 0.0000 | 0.0000 | 0.0000 | 0.0000 | 0.0000 | 0.0000 |
| Lebanon | UMIC | Eastern Mediterranean | 0.0000 | 0.0592 | 0.0040 | 0.0631 | 0.0000 | 0.0010 | 0.0035 | 0.0045 | 0.0000 | 0.0601 | 0.0075 | 0.0676 |
| Lesotho | LMIC | Africa | 0.6305 | 0.5815 | 0.6359 | 0.5869 | 0.0096 | 0.0089 | 0.0139 | 0.0133 | 0.6401 | 0.5904 | 0.6499 | 0.6002 |
| Liberia | LIC | Africa | 0.1561 | 0.0911 | 0.1574 | 0.0923 | 0.0023 | 0.0014 | 0.0033 | 0.0024 | 0.1585 | 0.0925 | 0.1607 | 0.0947 |
| Libya | UMIC | Eastern Mediterranean | 0.1705 | 0.2952 | 0.1759 | 0.3006 | 0.0028 | 0.0048 | 0.0075 | 0.0094 | 0.1733 | 0.3000 | 0.1833 | 0.3100 |
| Lithuania | HIC | Europe | 0.0000 | 0.0000 | 0.0000 | 0.0000 | 0.0000 | 0.0000 | 0.0000 | 0.0000 | 0.0000 | 0.0000 | 0.0000 | 0.0000 |
| Luxembourg | HIC | Europe | 0.0000 | 0.0000 | 0.0000 | 0.0000 | 0.0000 | 0.0000 | 0.0000 | 0.0000 | 0.0000 | 0.0000 | 0.0000 | 0.0000 |
| Madagascar | LIC | Africa | 0.3725 | 0.3404 | 0.3751 | 0.3430 | 0.0055 | 0.0051 | 0.0075 | 0.0071 | 0.3781 | 0.3455 | 0.3827 | 0.3502 |
| Malawi | LIC | Africa | 0.1680 | 0.1449 | 0.1692 | 0.1461 | 0.0025 | 0.0022 | 0.0034 | 0.0031 | 0.1705 | 0.1471 | 0.1727 | 0.1492 |
| Malaysia | UMIC | Western Pacific | 0.0378 | 0.0598 | 0.0397 | 0.0617 | 0.0006 | 0.0010 | 0.0023 | 0.0027 | 0.0384 | 0.0608 | 0.0420 | 0.0643 |
| Maldives | UMIC | South-East Asia | 0.0000 | 0.0000 | 0.0023 | 0.0023 | 0.0000 | 0.0000 | 0.0020 | 0.0020 | 0.0000 | 0.0000 | 0.0043 | 0.0043 |
| Mali | LIC | Africa | 0.2774 | 0.2105 | 0.2794 | 0.2126 | 0.0041 | 0.0032 | 0.0057 | 0.0048 | 0.2815 | 0.2137 | 0.2851 | 0.2174 |
| Malta | HIC | Europe | 0.0000 | 0.0000 | 0.0000 | 0.0000 | 0.0000 | 0.0000 | 0.0000 | 0.0000 | 0.0000 | 0.0000 | 0.0000 | 0.0000 |
| Mauritania | LMIC | Africa | 0.2872 | 0.2284 | 0.2894 | 0.2306 | 0.0043 | 0.0035 | 0.0060 | 0.0052 | 0.2915 | 0.2318 | 0.2954 | 0.2358 |
| Mauritius | UMIC | Africa | 0.1422 | 0.1302 | 0.1438 | 0.1318 | 0.0024 | 0.0022 | 0.0037 | 0.0036 | 0.1445 | 0.1323 | 0.1475 | 0.1354 |
| Mexico | UMIC | Americas | 0.1602 | 0.1672 | 0.1616 | 0.1687 | 0.0025 | 0.0026 | 0.0037 | 0.0037 | 0.1626 | 0.1698 | 0.1653 | 0.1724 |
| Mongolia | LMIC | Western Pacific | 0.0000 | 0.0000 | 0.0041 | 0.0041 | 0.0000 | 0.0000 | 0.0036 | 0.0036 | 0.0000 | 0.0000 | 0.0077 | 0.0077 |
| Montenegro | UMIC | Europe | 0.0426 | 0.0000 | 0.0446 | 0.0021 | 0.0007 | 0.0000 | 0.0026 | 0.0019 | 0.0433 | 0.0000 | 0.0473 | 0.0040 |
| Morocco | LMIC | Eastern Mediterranean | 0.5317 | 0.4911 | 0.5358 | 0.4953 | 0.0082 | 0.0076 | 0.0115 | 0.0110 | 0.5398 | 0.4987 | 0.5474 | 0.5063 |
| Mozambique | LIC | Africa | 0.2539 | 0.2300 | 0.2557 | 0.2318 | 0.0038 | 0.0035 | 0.0052 | 0.0049 | 0.2577 | 0.2334 | 0.2609 | 0.2367 |
| Myanmar | LMIC | South-East Asia | 0.3399 | 0.3190 | 0.3425 | 0.3216 | 0.0053 | 0.0050 | 0.0074 | 0.0071 | 0.3452 | 0.3240 | 0.3499 | 0.3287 |
| Namibia | UMIC | Africa | 0.0674 | 0.2242 | 0.0721 | 0.2289 | 0.0011 | 0.0036 | 0.0051 | 0.0075 | 0.0686 | 0.2279 | 0.0772 | 0.2364 |
| Nepal | LIC | South-East Asia | 1.0965 | 1.1173 | 1.1042 | 1.1249 | 0.0167 | 0.0170 | 0.0228 | 0.0230 | 1.1132 | 1.1342 | 1.1269 | 1.1479 |
| Netherlands | HIC | Europe | 0.0000 | 0.0000 | 0.0000 | 0.0000 | 0.0000 | 0.0000 | 0.0000 | 0.0000 | 0.0000 | 0.0000 | 0.0000 | 0.0000 |
| New Zealand | HIC | Western Pacific | 0.0000 | 0.0000 | 0.0000 | 0.0000 | 0.0000 | 0.0000 | 0.0000 | 0.0000 | 0.0000 | 0.0000 | 0.0000 | 0.0000 |
| Nicaragua | LMIC | Americas | 0.0547 | 0.1485 | 0.0591 | 0.1529 | 0.0009 | 0.0025 | 0.0048 | 0.0063 | 0.0556 | 0.1510 | 0.0639 | 0.1592 |
| Niger | LIC | Africa | 0.1307 | 0.1142 | 0.1316 | 0.1151 | 0.0019 | 0.0017 | 0.0026 | 0.0024 | 0.1326 | 0.1159 | 0.1342 | 0.1175 |
| Nigeria | LMIC | Africa | 0.1891 | 0.0000 | 0.1908 | 0.0017 | 0.0029 | 0.0000 | 0.0042 | 0.0014 | 0.1920 | 0.0000 | 0.1949 | 0.0031 |
| North Macedonia | UMIC | Europe | 0.0910 | 0.0142 | 0.0936 | 0.0169 | 0.0016 | 0.0002 | 0.0039 | 0.0026 | 0.0925 | 0.0145 | 0.0975 | 0.0195 |
| Norway | HIC | Europe | 0.0000 | 0.0000 | 0.0000 | 0.0000 | 0.0000 | 0.0000 | 0.0000 | 0.0000 | 0.0000 | 0.0000 | 0.0000 | 0.0000 |
| Oman | HIC | Eastern Mediterranean | 0.0000 | 0.0000 | 0.0000 | 0.0000 | 0.0000 | 0.0000 | 0.0000 | 0.0000 | 0.0000 | 0.0000 | 0.0000 | 0.0000 |
| Pakistan | LMIC | Eastern Mediterranean | 0.1598 | 0.1319 | 0.1610 | 0.1331 | 0.0024 | 0.0020 | 0.0033 | 0.0029 | 0.1622 | 0.1339 | 0.1643 | 0.1360 |
| Panama | UMIC | Americas | 0.0000 | 0.0741 | 0.0018 | 0.0759 | 0.0000 | 0.0012 | 0.0016 | 0.0028 | 0.0000 | 0.0753 | 0.0034 | 0.0787 |
| Papua New Guinea | LMIC | Western Pacific | 0.1641 | 0.0705 | 0.1655 | 0.0719 | 0.0025 | 0.0011 | 0.0036 | 0.0023 | 0.1667 | 0.0717 | 0.1691 | 0.0741 |
| Paraguay | UMIC | Americas | 0.1006 | 0.1459 | 0.1039 | 0.1492 | 0.0017 | 0.0024 | 0.0046 | 0.0053 | 0.1023 | 0.1483 | 0.1085 | 0.1546 |
| Peru | UMIC | Americas | 0.5131 | 1.0124 | 0.5211 | 1.0204 | 0.0083 | 0.0156 | 0.0150 | 0.0220 | 0.5214 | 1.0280 | 0.5361 | 1.0424 |
| Philippines | LMIC | Western Pacific | 0.0874 | 0.0000 | 0.0883 | 0.0009 | 0.0013 | 0.0000 | 0.0020 | 0.0007 | 0.0888 | 0.0000 | 0.0903 | 0.0016 |
| Poland | HIC | Europe | 0.0000 | 0.0000 | 0.0000 | 0.0000 | 0.0000 | 0.0000 | 0.0000 | 0.0000 | 0.0000 | 0.0000 | 0.0000 | 0.0000 |
| Portugal | HIC | Europe | 0.0000 | 0.0000 | 0.0000 | 0.0000 | 0.0000 | 0.0000 | 0.0000 | 0.0000 | 0.0000 | 0.0000 | 0.0000 | 0.0000 |
| Puerto Rico | HIC |  | 0.0000 | 0.0000 | 0.0000 | 0.0000 | 0.0000 | 0.0000 | 0.0000 | 0.0000 | 0.0000 | 0.0000 | 0.0000 | 0.0000 |
| Qatar | HIC | Eastern Mediterranean | 0.0000 | 0.0000 | 0.0000 | 0.0000 | 0.0000 | 0.0000 | 0.0000 | 0.0000 | 0.0000 | 0.0000 | 0.0000 | 0.0000 |
| Republic of Korea | HIC | Western Pacific | 0.0000 | 0.0000 | 0.0000 | 0.0000 | 0.0000 | 0.0000 | 0.0000 | 0.0000 | 0.0000 | 0.0000 | 0.0000 | 0.0000 |
| Republic of Moldova | LMIC | Europe | 0.0130 | 0.0000 | 0.0174 | 0.0044 | 0.0002 | 0.0000 | 0.0043 | 0.0040 | 0.0132 | 0.0000 | 0.0217 | 0.0085 |
| Romania | UMIC | Europe | 0.0202 | 0.0000 | 0.0219 | 0.0017 | 0.0004 | 0.0000 | 0.0019 | 0.0016 | 0.0206 | 0.0000 | 0.0239 | 0.0033 |
| Russian Federation | UMIC | Europe | 0.0000 | 0.0000 | 0.0027 | 0.0027 | 0.0000 | 0.0000 | 0.0024 | 0.0024 | 0.0000 | 0.0000 | 0.0051 | 0.0051 |
| Rwanda | LIC | Africa | 0.1521 | 0.1184 | 0.1532 | 0.1196 | 0.0023 | 0.0018 | 0.0032 | 0.0028 | 0.1544 | 0.1203 | 0.1564 | 0.1224 |
| SVG | UMIC | Americas | 0.1169 | 0.1523 | 0.1201 | 0.1555 | 0.0019 | 0.0025 | 0.0047 | 0.0052 | 0.1189 | 0.1548 | 0.1248 | 0.1607 |
| Saint Lucia | UMIC | Americas | 0.0219 | 0.1031 | 0.0252 | 0.1065 | 0.0004 | 0.0017 | 0.0033 | 0.0046 | 0.0223 | 0.1048 | 0.0286 | 0.1111 |
| Samoa | LMIC | Western Pacific | 0.2731 | 0.1467 | 0.2753 | 0.1489 | 0.0043 | 0.0024 | 0.0060 | 0.0043 | 0.2774 | 0.1491 | 0.2814 | 0.1532 |
| Sao Tome and Principe | LMIC | Africa | 0.1638 | 0.0000 | 0.1657 | 0.0019 | 0.0025 | 0.0000 | 0.0041 | 0.0016 | 0.1664 | 0.0000 | 0.1698 | 0.0035 |
| Saudi Arabia | HIC | Eastern Mediterranean | 0.0000 | 0.0000 | 0.0000 | 0.0000 | 0.0000 | 0.0000 | 0.0000 | 0.0000 | 0.0000 | 0.0000 | 0.0000 | 0.0000 |
| Senegal | LIC | Africa | 0.2310 | 0.1860 | 0.2327 | 0.1876 | 0.0034 | 0.0028 | 0.0047 | 0.0041 | 0.2344 | 0.1888 | 0.2374 | 0.1918 |
| Serbia | UMIC | Europe | 0.0187 | 0.0000 | 0.0222 | 0.0035 | 0.0003 | 0.0000 | 0.0036 | 0.0032 | 0.0190 | 0.0000 | 0.0258 | 0.0068 |
| Sierra Leone | LIC | Africa | 0.2893 | 0.2438 | 0.2914 | 0.2459 | 0.0043 | 0.0037 | 0.0059 | 0.0053 | 0.2935 | 0.2475 | 0.2972 | 0.2512 |
| Singapore | HIC | Western Pacific | 0.0000 | 0.0000 | 0.0000 | 0.0000 | 0.0000 | 0.0000 | 0.0000 | 0.0000 | 0.0000 | 0.0000 | 0.0000 | 0.0000 |
| Slovakia | HIC | Europe | 0.0000 | 0.0000 | 0.0000 | 0.0000 | 0.0000 | 0.0000 | 0.0000 | 0.0000 | 0.0000 | 0.0000 | 0.0000 | 0.0000 |
| Slovenia | HIC | Europe | 0.0000 | 0.0000 | 0.0000 | 0.0000 | 0.0000 | 0.0000 | 0.0000 | 0.0000 | 0.0000 | 0.0000 | 0.0000 | 0.0000 |
| Solomon Islands | LMIC | Western Pacific | 0.1220 | 0.0000 | 0.1234 | 0.0013 | 0.0019 | 0.0000 | 0.0030 | 0.0012 | 0.1240 | 0.0000 | 0.1264 | 0.0025 |
| South Africa | UMIC | Africa | 0.0193 | 0.0000 | 0.0217 | 0.0024 | 0.0003 | 0.0000 | 0.0024 | 0.0021 | 0.0196 | 0.0000 | 0.0241 | 0.0045 |
| Spain | HIC | Europe | 0.0000 | 0.0000 | 0.0000 | 0.0000 | 0.0000 | 0.0000 | 0.0000 | 0.0000 | 0.0000 | 0.0000 | 0.0000 | 0.0000 |
| Sri Lanka | LMIC | South-East Asia | 0.1136 | 0.0697 | 0.1148 | 0.0708 | 0.0019 | 0.0012 | 0.0028 | 0.0022 | 0.1155 | 0.0709 | 0.1176 | 0.0730 |
| Sudan | LMIC | Eastern Mediterranean | 0.3201 | 0.2357 | 0.3237 | 0.2392 | 0.0050 | 0.0037 | 0.0078 | 0.0066 | 0.3251 | 0.2394 | 0.3315 | 0.2458 |
| Suriname | UMIC | Americas | 0.0000 | 0.0302 | 0.0035 | 0.0337 | 0.0000 | 0.0005 | 0.0031 | 0.0036 | 0.0000 | 0.0307 | 0.0067 | 0.0373 |
| Sweden | HIC | Europe | 0.0000 | 0.0000 | 0.0000 | 0.0000 | 0.0000 | 0.0000 | 0.0000 | 0.0000 | 0.0000 | 0.0000 | 0.0000 | 0.0000 |
| Switzerland | HIC | Europe | 0.0000 | 0.0000 | 0.0000 | 0.0000 | 0.0000 | 0.0000 | 0.0000 | 0.0000 | 0.0000 | 0.0000 | 0.0000 | 0.0000 |
| Tajikistan | LMIC | Europe | 0.2663 | 0.1978 | 0.2690 | 0.2004 | 0.0041 | 0.0031 | 0.0063 | 0.0053 | 0.2704 | 0.2009 | 0.2753 | 0.2057 |
| Thailand | UMIC | South-East Asia | 0.1121 | 0.1183 | 0.1140 | 0.1201 | 0.0019 | 0.0020 | 0.0035 | 0.0036 | 0.1140 | 0.1203 | 0.1175 | 0.1237 |
| Timor-Leste | LMIC | South-East Asia | 0.3686 | 0.3532 | 0.3716 | 0.3561 | 0.0056 | 0.0054 | 0.0080 | 0.0078 | 0.3742 | 0.3586 | 0.3796 | 0.3639 |
| Togo | LIC | Africa | 0.2022 | 0.1601 | 0.2037 | 0.1616 | 0.0030 | 0.0024 | 0.0041 | 0.0036 | 0.2052 | 0.1625 | 0.2078 | 0.1652 |
| Tonga | LMIC | Western Pacific | 0.1119 | 0.0000 | 0.1138 | 0.0019 | 0.0018 | 0.0000 | 0.0034 | 0.0017 | 0.1137 | 0.0000 | 0.1172 | 0.0036 |
| Trinidad and Tobago | HIC | Americas | 0.0000 | 0.0000 | 0.0000 | 0.0000 | 0.0000 | 0.0000 | 0.0000 | 0.0000 | 0.0000 | 0.0000 | 0.0000 | 0.0000 |
| Tunisia | LMIC | Eastern Mediterranean | 0.2131 | 0.0023 | 0.2181 | 0.0074 | 0.0035 | 0.0000 | 0.0079 | 0.0045 | 0.2166 | 0.0024 | 0.2260 | 0.0119 |
| Turkey | UMIC | Europe | 0.0000 | 0.0000 | 0.0036 | 0.0036 | 0.0000 | 0.0000 | 0.0032 | 0.0032 | 0.0000 | 0.0000 | 0.0068 | 0.0068 |
| Turkmenistan | UMIC | Europe | 0.1786 | 0.1865 | 0.1811 | 0.1890 | 0.0028 | 0.0030 | 0.0049 | 0.0050 | 0.1814 | 0.1894 | 0.1860 | 0.1940 |
| Uganda | LIC | Africa | 0.1847 | 0.1461 | 0.1861 | 0.1475 | 0.0027 | 0.0022 | 0.0038 | 0.0033 | 0.1875 | 0.1483 | 0.1899 | 0.1508 |
| Ukraine | LMIC | Europe | 0.0000 | 0.0000 | 0.0024 | 0.0024 | 0.0000 | 0.0000 | 0.0022 | 0.0022 | 0.0000 | 0.0000 | 0.0046 | 0.0046 |
| United Arab Emirates | HIC | Eastern Mediterranean | 0.0000 | 0.0000 | 0.0000 | 0.0000 | 0.0000 | 0.0000 | 0.0000 | 0.0000 | 0.0000 | 0.0000 | 0.0000 | 0.0000 |
| United Kingdom | HIC | Europe | 0.0000 | 0.0000 | 0.0000 | 0.0000 | 0.0000 | 0.0000 | 0.0000 | 0.0000 | 0.0000 | 0.0000 | 0.0000 | 0.0000 |
| United Republic of Tanzania | LIC | Africa | 0.1797 | 0.1547 | 0.1810 | 0.1559 | 0.0027 | 0.0023 | 0.0037 | 0.0033 | 0.1823 | 0.1570 | 0.1846 | 0.1593 |
| United States of America | HIC | Americas | 0.0000 | 0.0000 | 0.0000 | 0.0000 | 0.0000 | 0.0000 | 0.0000 | 0.0000 | 0.0000 | 0.0000 | 0.0000 | 0.0000 |
| Uruguay | HIC | Americas | 0.0000 | 0.0000 | 0.0000 | 0.0000 | 0.0000 | 0.0000 | 0.0000 | 0.0000 | 0.0000 | 0.0000 | 0.0000 | 0.0000 |
| Uzbekistan | LMIC | Europe | 0.3629 | 0.1647 | 0.3679 | 0.1698 | 0.0058 | 0.0027 | 0.0099 | 0.0070 | 0.3686 | 0.1674 | 0.3778 | 0.1767 |
| Vanuatu | LMIC | Western Pacific | 0.2578 | 0.0819 | 0.2600 | 0.0842 | 0.0040 | 0.0014 | 0.0059 | 0.0033 | 0.2618 | 0.0833 | 0.2659 | 0.0875 |
| Viet Nam | LMIC | Western Pacific | 0.4092 | 0.3504 | 0.4131 | 0.3543 | 0.0065 | 0.0057 | 0.0098 | 0.0089 | 0.4158 | 0.3561 | 0.4229 | 0.3632 |
| Zambia | LMIC | Africa | 0.2853 | 0.2422 | 0.2875 | 0.2444 | 0.0042 | 0.0037 | 0.0059 | 0.0054 | 0.2895 | 0.2459 | 0.2934 | 0.2497 |
| Zimbabwe | LIC | Africa | 1.5486 | 1.1584 | 1.5621 | 1.1719 | 0.0235 | 0.0180 | 0.0341 | 0.0288 | 1.5721 | 1.1764 | 1.5962 | 1.2007 |

Approach 1 calculates unmet need as the relative difference of surgical volume to the minimum surgical volume proposed by the Lancet Commission on Global Surgery. Approach 2 calculates unmet need as the relative difference of estimated appendectomies (calculated as World Bank-income-group specific share of surgical volume) to the number of appendicitis cases.

1. As noted in the main document, this can be confirmed using OECD data on hospitalization in 2015. [↑](#footnote-ref-1)
2. This is different to the probability to receive surgical care or not, i.e., to be operated or not, which is derived from country-level surgical volume as explained above. [↑](#footnote-ref-2)
3. 4 studies for Northern America (Canada: 2, USA: 2), 2 studies for Northern Europe (Denmark, UK), 3 studies for Southern Europe (Albania, Italy, Spain), 2 studies for the Middle East (Iran, Turkey), 5 studies for Asia (South Korea, Taiwan: 4), 3 studies for Africa (South Africa, Nigeria: 2), and none for Eastern Europe, Western Europe, Oceania, or Southern and Central America. [↑](#footnote-ref-3)
