# Supplementary material for: Global economic burden of unmet surgical need for appendicitis
Source: Br J Surg. 2022 Jul 26;109(10):995–1003. doi: 10.1093/bjs/znac195 (PMC10364778; doi:10.1093/bjs/znac195)
Supplement: znac195_Supplementary_Data [file znac195_supplementary_data.zip › Supplementary_material.docx]

# Supplementary material

## Appendix S1. Detailed description of the method:

We determine three types of economic burden: consequences of not providing the local standard of care to all appendicitis patients, consequences of not providing the standard of care of high-income countries to all appendicitis patients, and the consequences of providing surgical care with local standard to the people who currently receive care and not providing surgical care to the people who currently receive no care.

To calculate these three types, we first estimated the excess occurrence of the adverse events of mortality and absenteeism per appendicitis case in a given country. Then, we multiplied these adverse events with the country’s incidence and annual wage.

### Occurrence of adverse events

To assess the occurrence of adverse events, we first estimated the extent of unmet need for surgical care in country i. We assumed that each case of appendicitis has a probability $p_{i}^{op}$ to be operated, and a probability $p_{i}^{noop}=1-p_{i}^{op}$ to forgo an operation (see Figure S1). Assuming that every case of appendicitis should undergo a surgical procedure, we refer to the probability $p_{i}^{noop}$ as the *share of unmet surgical need for appendicitis*. We assumed that this share is zero in high-income countries (HICs)^[[1]](#footnote-1)^, but varies across low- and middle-income countries (LMICs) where access to care is limited^7^.

*Figure S1. Probability tree 1: Probability of operation*


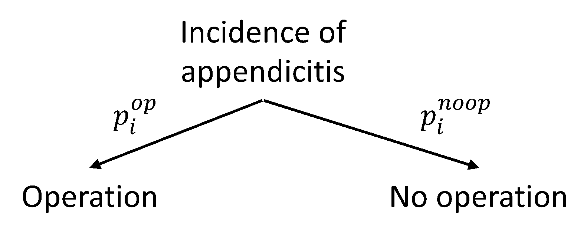


We estimated this share of unmet surgical need for LMICs using two approaches (see Table S2 for details on the data sources for both approaches):

1. The proportion of unmet need approach. Following the Lancet Commission on Global Surgery (LCoGS), we assumed the total surgical volume to meet need was 5,000 operations per 100,000 people^1^. The difference between the country’s actual surgical volume (obtained from Holmer et al (2019)^10^) and LCoGS estimate of volume to meet need was used to estimate the proportion of unmet need among the cases of appendicitis. Given that in many LMICs emergency surgeries^11^ (for example, appendectomies) form a larger proportion of total volume than in HICs, we interpret this derived proportion of unmet need as an upper estimate for the gap of unmet need.
2. The share of surgical volume approach. We assumed that appendectomies make up a certain share of the total surgical volume done (estimated from Holmer et al (2019)^10^). To estimate this share, we used information on the proportion of appendectomies to gastrointestinal surgery and information on the proportion of gastrointestinal surgery to total surgical volume from the literature and datasets as described in appendix S2. From this, we predicted the number of appendectomies for each country. The difference between a country’s incidence of appendicitis and the predicted number of appendectomies was used to estimate the proportion of unmet need among the cases of appendicitis.

Next, we assessed the consequences of met surgical need (operation) and unmet surgical need (no operation), as depicted in Figure S2. We divided the consequences of an operation into three categories: successful operation, minor adverse events, and reoperation. Similarly, we subdivide the consequences of no operation into mild cases, moderate cases, and severe cases. Each consequence occurs with a specific probability $p$, and entails a certain outcome $\omega$ in the form of mortality risk (as % of appendectomies done) and absenteeism (as number of working days lost) as depicted in Table S1. Sources of the estimates can be found in Table S5 and Table S6 in Appendix S2. The literature and available data do not allow us to disaggregate probabilities of scenarios in case of operation (e.g., of minor adverse events) or no operation (e.g., severe cases) or the associated outcomes $\omega$ coherently by country, age group, or sex^[[2]](#footnote-2)^. Hence, we assume them to be constant along these dimensions and to vary only between country income groups. In addition, we assume that the probabilities and outcomes are independent of the share of unmet need. This implicitly assumes that changes in the share of unmet need have no impact on the quality of care.

Figure S2. Probability tree 2: Scenarios and outcomes of (no) operation


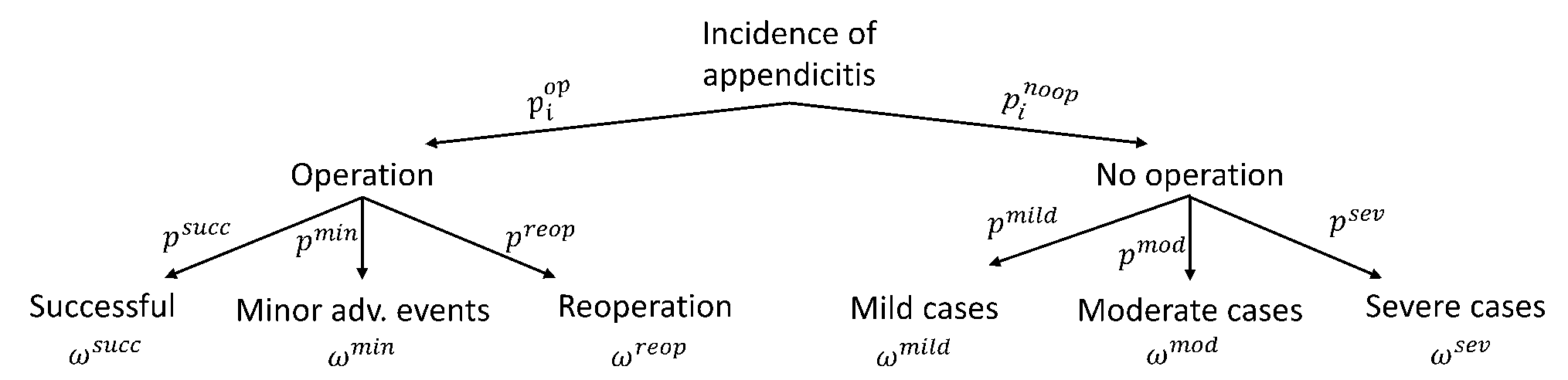


Table S1. Scenario probabilities and respective outcomes across scenarios and income groups

|  |  | **Successful operation** | **Minor adv. events** | **Reoperation** | **Mild cases** | **Moderate cases** | **Severe cases** |
| --- | --- | --- | --- | --- | --- | --- | --- |
| **Probability of scenario** | LMIC | 82% | 15.5% | 2.5% | 10% | 68.5% | 21.5% |
|  | HIC | 87% | 10.5% | 2.5% | NA | NA | NA |
| **Outcome: Mortality risk** | LMIC | 0% | 0.6% | 0.6% | 0% | 10% | 19% |
|  | HIC | 0% | 0.1% | 0.1% | NA | NA | NA |
| **Outcome: Absen-teeism (days lost)** | LMIC | 7 | 24 | 60 | 4 | 15 | 60 |
|  | HIC | 4 | 15 | 60 | NA | NA | NA |

Based on this, we estimated the rate of excess adverse events due to not providing surgery with the local standard in LMICs, and not providing surgical care with the standard available in HICs as displayed in Figure S3.

Figure S3. Graphic display of the calculation of adverse events with the local or the highest standard


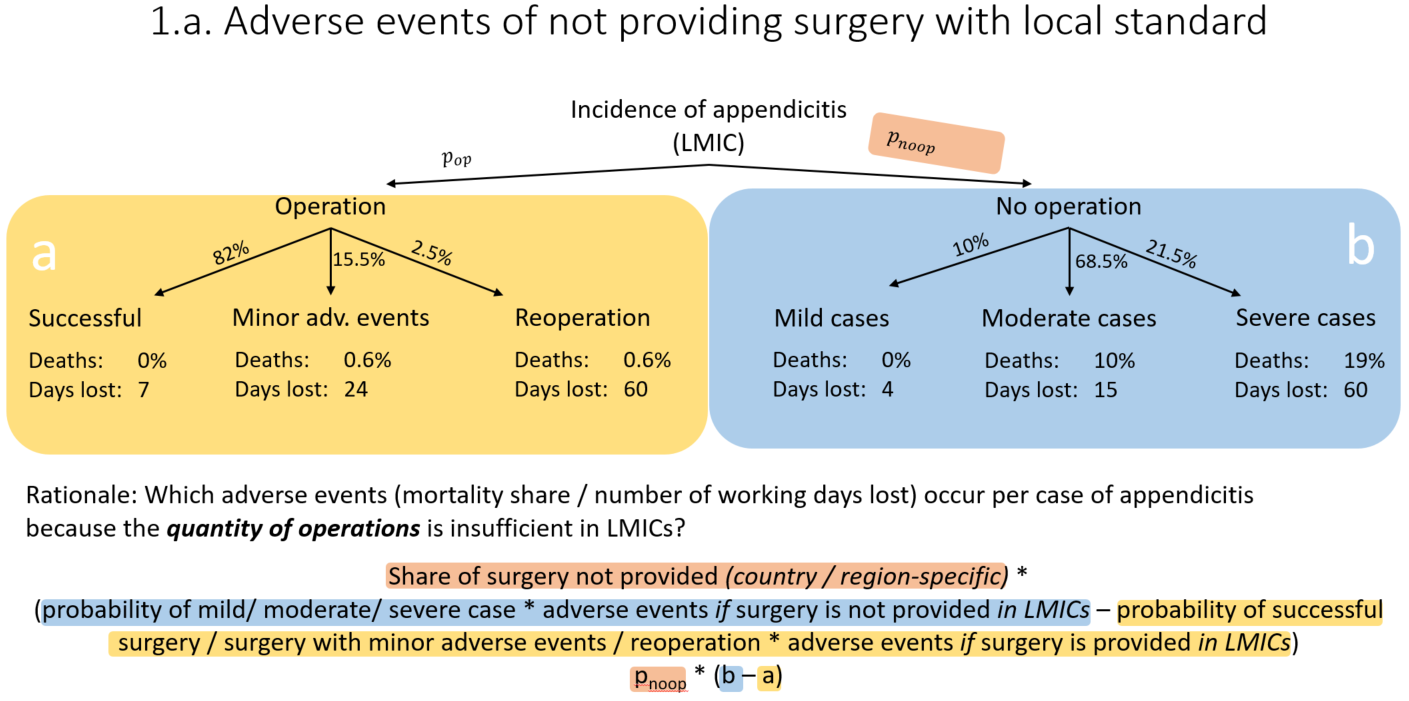

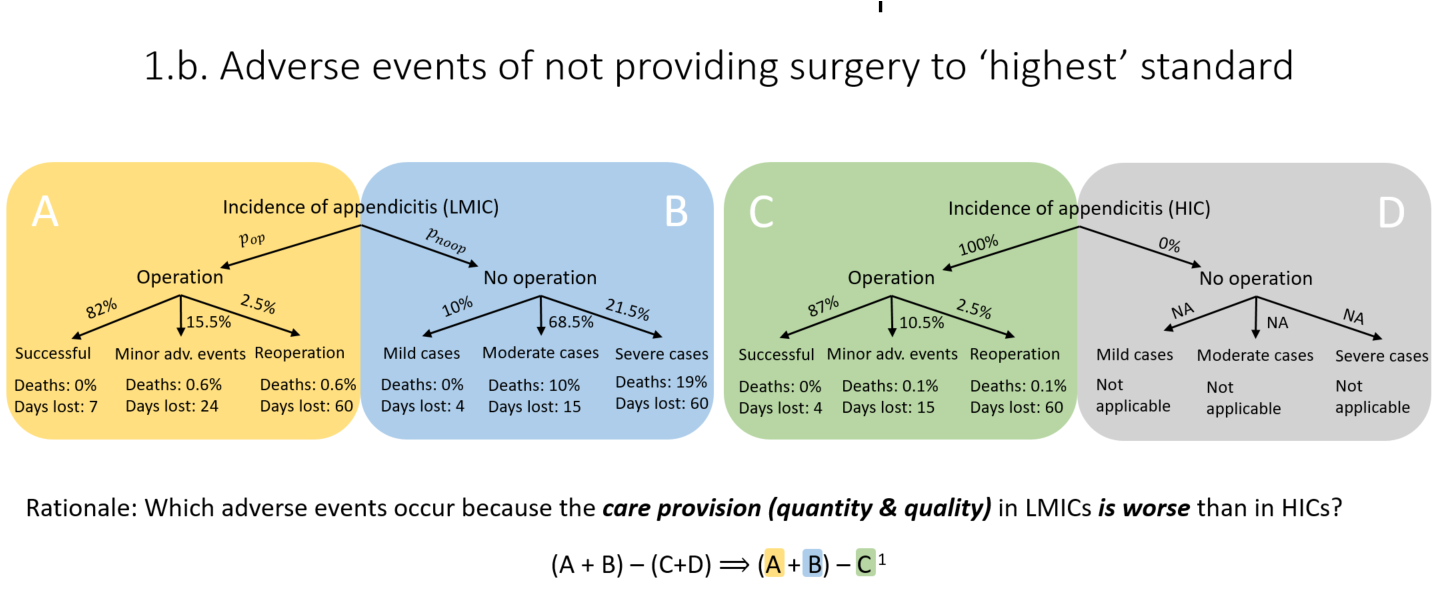


1 We assume that all appendicitis cases are operated in HICs, such that D=0; LMIC = low-and middle income countries; HIC= high income countries

Depending on the outcome of interest, we refer to the result as *rate of excess mortality* $\delta_{i}^{mort}$ respectively the *rate of excess absenteeism* $\delta_{i}^{abs}$. It denotes the (expected) additional mortality risk and working days lost per case of appendicitis due to unmet surgical need or substandard quality of care.

In addition, we estimated the rate of adverse events occurring under the current state of care, i.e., with some individuals treated with local standards, and others remaining untreated as displayed in Figure S4.

Figure S4. Graphic display of the total indirect cost calculation of the current state of care


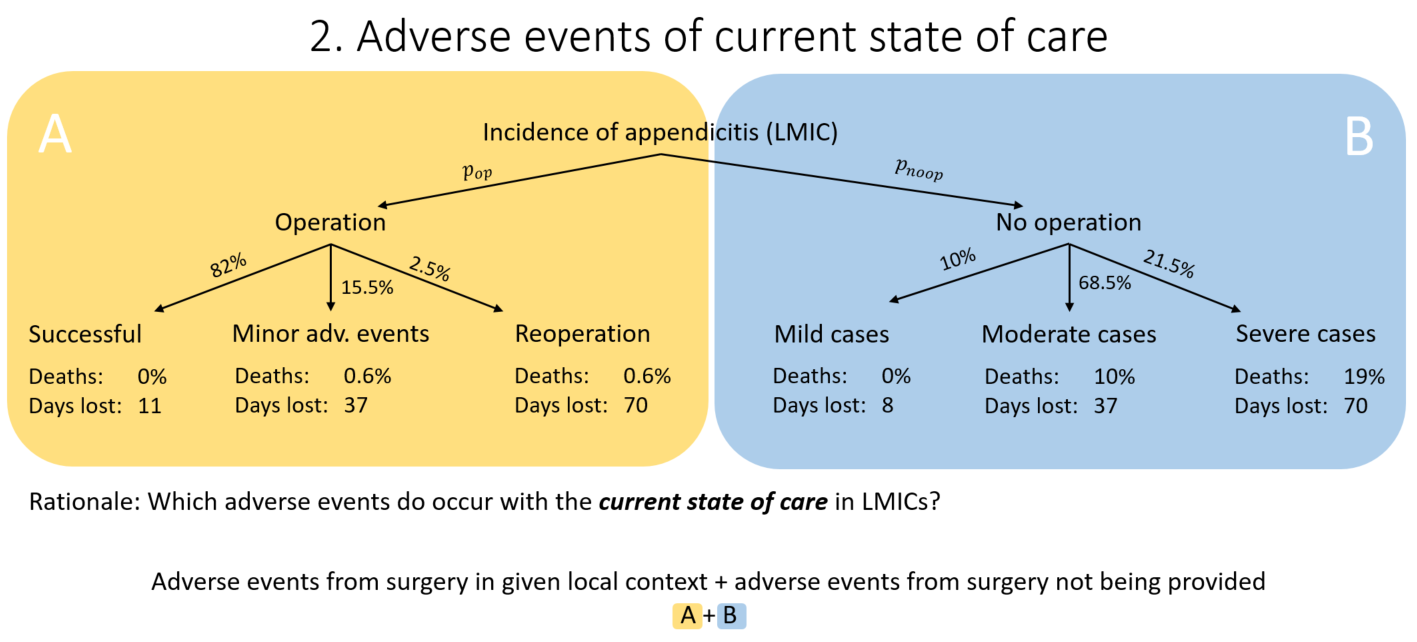


### Income losses

We calculated the income losses due to early mortality following Bommer et al. (2017)^30^. For each country, we calculated by age group how many people were expected to die due to appendicitis by multiplying the incidence of appendicitis with the excess mortality risk from each scenario. Then, we multiplied this excess mortality with the wage the individuals would be expected to have earned if they would not have died. As the existing wage data did not allow a disaggregation by age group, we assumed that the average patient with appendicitis earns the average annual wage.

Expressed as formula, the mortality related income losses for each age group $s$ in a given country $i$ are given as:

$$C_{s,i}^{mort}=annual wage_{i}*N_{s,i}*\sum_{t=0}^{T_{s}-1} \frac{1}{\left( 1+0.03 \right)^{t}}$$

with $N_{s,i}$ as the number of excess deaths in age group $s$ and country $i$, as well as $T_{s}$ as the number of years lost until retirement. To calculate the income losses due to early mortality, we assumed that individuals retire at age 65, age-group specific death occurs in the middle of each age group, and a discount rate of 0.03 per year.

We obtained the number of excess deaths $N_{s,i}$ by multiplying the rate of excess mortality with the incidence of appendicitis:

$$N_{s,i}=\delta_{i}^{mort}*Inc_{s,i}$$

Substituting this into the formula yields:

$$C_{s,i}^{mort}=annual wage_{i}*\delta_{i}^{mort}*Inc_{s,i}*\sum_{t=0}^{T_{s}-1} \frac{1}{\left( 1+0.03 \right)^{t}}$$

Note that within a given country, all variation in mortality related income losses across age groups is based on the variation in incidence and the respective discount factor.

In the same way, we calculated the income losses due to absenteeism following Bommer et al. (2017). For each country, we calculated by age group how many working days in total were lost due to appendicitis by multiplying the incidence of appendicitis (minus those who died) with the excess number of lost working days. Then, we calculated the income losses due to this excess absenteeism by multiplying it with the expected daily earnings.

The formula is given as:

$$C_{s,i}^{abs}=\frac{annual wage_{i}}{261}*N_{s,i}^{LFP}*\delta_{i}^{abs}$$

with $N_{s,i}^{LFP}$ as the number of individuals with unmet surgical need who are available to the labor force. To compute daily wages, we assumed that on average, annual earnings were distributed over 261 working days. As above, the existing data did not allow a disaggregation of wages by age group. We calculated $N_{s,i}^{LFP}$ as follows:

$$N_{s,i}^{LFP}=\left( 1-\delta_{i}^{mort} \right)*Inc_{s,i}$$

Substituting this into the formula yields:

$$C_{s,i}^{abs}=\frac{annual wage_{i}}{261}*\left( 1-\delta_{i}^{mort} \right)*Inc_{s,i}*\delta_{i}^{abs}$$

Note that within a given country, all variation in absenteeism related income losses across age groups is based on the variation in incidence.

### Aggregation and economic burden

The stratum-specific costs were aggregated by country and WHO region for the majority of analyses. For each scenario, the economic burden was calculated as the sum of mortality and absenteeism related income losses.

### Gender differences

Due to the low availability of gender-disaggregated data, a disaggregation of the economic burden by gender would be solely driven by the gendered difference in incidence rates as reported by the GBD estimates. Thus, they are not reported in the main part. An overview on the economic burden disaggregated by gender and region is provided in Figure S13 and Figure S14.

## Appendix S2: Details on data sources:

The indicators for this study (unmet need, adverse outcomes and their probabilities, wages) are gathered and harmonized from several sources as listed in detail below.

### Incidence of appendicitis

A comparison of data on the incidence of appendicitis from the Global Burden of Disease estimates and similar data from the OECD revealed a large discrepancy, with the GBD data yielding about a twice as high incidence as OECD figures (see Figure S5). A reason for this discrepancy might be that OECD data covers hospital discharge data, while GBD data is based on various data sources, including household surveys, which are harmonized and imputed for countries with missing data. Unfortunately, OECD data is mainly available for OECD countries, such that we cannot analyze whether the discrepancy between both sources varies over income groups or regions.

Figure S5. Comparison of GBD incidence rate and OECD incidence rate


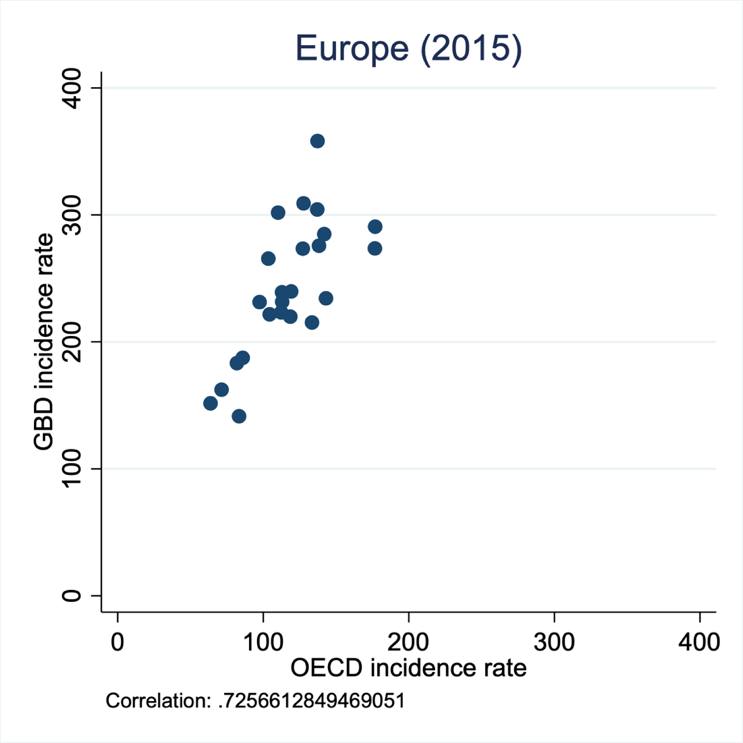


Another data source for the incidence of appendicitis would have been the systematic literature review by Ferris et al.^31^ This review pooled studies on appendicitis and appendectomies to construct estimates for the incidence of appendicitis by region. Assuming that not all appendicitis is treated, this would reveal an underestimate of appendicitis, which is systematically biased towards regions with a poorer access to surgery. Considering only studies on appendicitis, and only studies not older than 2000, only 19 studies remain. Most of them are from high-income countries, only the studies on Canada, South Korea, Taiwan and the US are on the national level, and some regions are covered by no study at all^[[3]](#footnote-3)^. Thus, for most countries and regions, data on appendicitis is missing, except for data provided by the GBD estimates.

### Unmet need

Table S2. Data sources for indicators used to estimate the unmet surgical need

|  | Indicator name | Source | Comment |
| --- | --- | --- | --- |
| $\mathbf{In}\mathbf{c}_{\mathbf{s},\mathbf{i},\mathbf{y}}$ | Incidence | GBD^6^ | Available by country and year, disaggregated by age-group and sex, 1990-2019 |
| $\mathbf{p}_{\mathbf{noop,i}}\boldsymbol{\& p}_{\mathbf{op,i}}$ | Unmet surgical need in appendicitis approach 1 | Holmer et al (2019)^10^ | Surgical volume by country for the year 2015 (actual or imputed) |
|  |  | LCoGS^1^ | General need for surgery |
|  | Unmet surgical need in appendicitis approach 2 | Holmer et al (2019)^10^ | Surgical volume by country for the year 2015 (actual or imputed) |
|  |  | COVIDSurg Collaborative, GlobalSurg Collaborative data^12^ | Share of appendectomies in GI surgery by income group; derived from the number of appendectomies and other GI surgeries among patients undergoing any elective or emergency surgery in October 2020 in 1,674 hospitals in 112 countries (47 HICs, 33 UMICs, 22 LMICs, 10 LICs); see Table S3 for income-group aggregates; due to the lower number of observations in LIC, we grouped low- and lower middle income countries in one category as the best available approximation. |
|  |  | Hospital Episode Statistics (HES) data^32^ | Share of GI in total surgery for high-income countries, based on hospital admissions in NHS hospitals in England, aggregated from 2014/15 to 2019/20 |
|  |  | LeBrun et al (2012)^33^  LeBrun et al (2013)^34^  Solis et al (2013)^35^  Bolkan et al (2014)^36^  Knowlton et al 2013^37^  Linden et al 2012^38^  Adde et al (2020)^39^  Albutt et al (2019)^40^  Gyedu et al (2018)^41^  Vansell et al (2014)^42^ | Share of GI in total surgery for low- and middle-income countries derived from the literature for varying years and countries, all available data is aggregated to the income group as depicted in Table S4 |

Table S3. Proportion of appendectomies in surgical volume and all its components by income group

|  | Income group | | | |  |
| --- | --- | --- | --- | --- | --- |
|  | High | Upper Middle | Lower Middle | Low | |
| Number of appendectomies ^a^ | 3,338 | 1,513 | 1,372 ^b^ | | |
| Total number of GI surgeries ^a^ | 24,848 | 6,938 | 6,601 ^b^ | | |
| Share of appendectomies in total GI surgery (number of appendectomies / total number of GI surgeries) | 13.43% | 21.81% | 20.78%^b^ | | |
| Share of GI to total surgical volume ^c^ | 9.36% | 21.36% | 36.09% | 45.55% | |
| Share of appendectomies in total surgical volume (Share of appendectomies in GI surgery*share of GI in total surgery) | 1.26% | 4.67% | 7.50% | 9.47% | |

a: from COVIDSurg Collaborative, GlobalSurg Collaborative database^12^; b: as the number of countries in the LIC category is lower than in the other income groups, we grouped LICs and LMICs into one figure; c: from the literature, see Table S4 for details.

### Systematic review

The literature sources for the proportion of GI surgery in total surgical volume were identified by an Embase Database search. The search string and resulting numbers of studies are listed below.

1 surgical volume/ (680)

2 surgical capacity/ (4)

3 (Surgical adj8 an?esthesia adj8 capacity).ti,ab,kw. (36)

4 (Enumeration adj8 Operation?).ti,ab,kw. (18)

5 (Surgery adj8 an?esthesia adj8 capacity).ti,ab,kw. (48)

6 1 or 2 or 3 or 4 or 5 (777)

7 limit 6 to (human and english language and (article or article in press or "review")) (

Two reviewers (MK) and (MM) independently reviewed the articles to assess if they were peer reviewed papers that contained data on the proportion of surgeries that were GI surgeries. At the end disagreements between the reviewers were resolved by discussions and 10 articles were selected for data collection (see Table S4). One study from Uganda^38^ was excluded as it reports emergency surgeries separately, making the total share not comparable to the other studies.

As several studies were available for lower-middle- and low-income countries, we selected the studies which the highest sample size from each income category. Hence, we used the proportion of surgeries that were GI surgeries were sourced from Bangladesh^34^ and Sierra Leone^36^ to represent lower-middle- and low-income countries respectively.

Table S4. Literature sources and data for the proportion of GI surgery to total surgical volume for low- and middle-income countries

| Income group | Country and reference | Year | Proportion of GI to total surgical volume |
| --- | --- | --- | --- |
| Upper Middle | Guyana^42^ | Not reported | 21.36% |
| Lower Middle | Bolivia^33^ | 2011 | 45.29% |
|  | Nicaragua^35^ | 2010 | 50.44% |
|  | Bangladesh^34^ | 2012 | 36.09% |
|  | Ghana^41^ | 2014/15 | 18.41% |
| Low | Liberia^37^ | 2009-10 | 50.00% |
|  | Liberia^39^ | 2018 | 39.7% |
|  | Uganda^40^ | 2016 | 23.76% |
|  | Sierra Leone^36^ | 2012 | 45.55% |

### Probabilities and outcomes of surgical scenarios

#### Surgically treated appendicitis

For the probabilities and outcomes of the three operative scenarios, we relied on data from the GlobalSurg 1 and 2 studies^3,15^. These contain individual data from 8,611 patients (4,532 from GS1 and 4,079 from GS2) who underwent surgical treatment for appendicitis across countries in all income groups. The figures were calculated by combining low- and middle-income country data as there are too few observations from LICs to make it a separate category. We categorized three surgical scenarios: Successful surgery (succ) was defined as discharge from follow up without wound infection or reoperation in the first 30 days. Surgery with minor adverse (min) events was defined as wound infection identified within the first 30 days of surgery. Reoperation (reop) were those cases that returned to theatre for further surgery in the first 30 days following appendectomy. The respective mortality outcomes are retrieved from GlobalSurg 1 and 2 studies^3,15^, and the absenteeism outcomes from the literature as listed in detail in Table S5.

Table S5. Data sources for indicators on probabilities and outcomes of surgical scenarios

| Indicator name | Source | Value | Comment |
| --- | --- | --- | --- |
| *Probability of scenarios after operation:* | | |  |
| Successful surgery ($\mathbf{p}_{\mathbf{succ}}$) | GlobalSurg1 and 2^3,15^ | LMIC: 82%  HIC: 87% | Defined as discharge from follow up without wound infection or reoperation in the first 30 days of the appendectomy. |
| Minor adverse events ($\mathbf{p}_{\mathbf{min}}$) | GlobalSurg1 and 2^3,15^ | LMIC: 15.5%  HIC: 10.5% | Defined as wound infection identified within the first 30 days of the appendectomy. |
| Reoperation ($\mathbf{p}_{\mathbf{reop}}$) | GlobalSurg1 and 2^3,15^ | LMIC: 2.5%  HIC: 2.5% | Defined as those cases that returned to theatre for further surgery in the first 30 days after appendectomy. |
| *Outcomes after operation:* $\boldsymbol{\omega}$ | |  |  |
| Death rates by scenario | GlobalSurg1 and 2^3,15^ | $\boldsymbol{\omega}_{\mathbf{succ}}\boldsymbol{:}$ LMIC 0%  HIC: 0%  $\boldsymbol{\omega}_{\mathbf{min}}\boldsymbol{:}$ LMIC 0.6%  HIC: 0.1%  $\boldsymbol{\omega}_{\mathbf{reop}}$**:** LMIC 0.6%  HIC: 0.1% | Defined as mortality within the first 30 days of the appendectomy. |
| Lost working days by scenario | Di Saverio et al (2014)^43^ | $\boldsymbol{\omega}_{\mathbf{succ}}\boldsymbol{:}$ LMIC: 7 | Based upon recovery from open appendectomy in HIC (Italy): rounded value for 6.6 days reported by Di Saverio et al (2014). The assumption is that LMICs will have more open appendectomies hence we used HIC data in the absence of studies from LMICs. |
|  | Martin et al (1995)^44^ | $\boldsymbol{\omega}_{\mathbf{min}}\boldsymbol{:}$ LMIC: 24 | Based on the recovery of patients undergoing open appendectomy for acute appendicitis in HIC. Rounded from 23.6 days to 24 days returning to work reported in the study. The assumption is that LMICs will have more open appendectomies hence we used HIC data in the absence of studies from LMICs |
|  | Opmeer et al (2010)^45^ | $\boldsymbol{\omega}_{\mathbf{reop}}$**:** LMIC: 60 | Estimated as the weighted average of lost working days reported in the study among patients undergoing on-demand (50 days) and planned re-laparotomy (70 days) for severe peritonitis in a HIC. |
|  | Di Saverio et al (2014)^43^ | $\boldsymbol{\omega}_{\mathbf{succ}}\boldsymbol{:}$ HIC: 4 | The authors found that laparoscopic and open appendectomy surgery patients had 2.1 and 6.6 days return to daily activities respectively. We estimated 4 as a weighted the average for patients undergoing HIC appendectomy. |
|  | Long et al (2001)^46^ | $\boldsymbol{\omega}_{\mathbf{min}}\boldsymbol{:}$ HIC: 15 | A prospective randomised comparison that compared laparoscopic versus open appendectomy in USA. The study found that appendectomy patients have 14.6 days off work or school after the surgery (for both laparoscopic and open appendectomy). The value has been rounded to the nearest whole number. |
|  | Opmeer et al (2010)^45^ | $\boldsymbol{\omega}_{\mathbf{reop}}$**:** HIC: 60 | Estimated as the weighted average of lost working days reported in the study among patients undergoing on-demand (50 days) and planned re-laparotomy (70 days) for severe peritonitis in a HIC. |

#### Not surgically treated appendicitis

For the non-operative scenarios, we distinguish mild, moderate and severe cases of appendicitis. For the probabilities of each scenario, we relied on the data from GlobalSurg 1 and 2 studies^3,15^ for high income countries only as we assume that all appendicitis cases are treated surgically in these countries. We apply this distribution to all countries assuming there is little variation in pathogenesis globally and hence apparent global variation in disease severity is due to delayed presentation and treatment. Mild cases (mild) were defined as non-gangenous appendicitis with a normal appendix at surgery. Moderate cases (mod) were defined as those with purulent peritonitis (Infection within the abdominal cavity, beyond the walls of the appendix – without perforation)*.* Severe cases (sev) were defined as those with a perforated appendix (perforation of a gangrenous appendix with localised or generalised faecal contamination). For the mortality outcomes, it is assumed that mild cases resolve with conservative treatment and are thus expected to not result in mortality. Moderate cases are considered comparable to (treated) emergency acute abdomen, using a national audit from the UK^47^. This figure of 10% is also replicated in the mortality from ‘all-comer’ laparotomies in LIC in the GlobalSurg2^3^ data. Severe cases (perforated appendicitis) are considered to carry a mortality comparable to that of (treated) faecal peritonitis in high-income countries using data from the GenOSept study^48^. This study provided data for 977 faecal peritonitis patients, recruited in 102 centres across 16 countries between 29/09/2005 and 5/01/2011. The median age was 69.2 years (IQR, Interquartile range 58.3-77.1). The most common causes of faecal peritonitis were perforated diverticular disease (32.1%) and surgical anastomotic breakdown (31.1%). The mortality rate at 28 days was 19.1% and 31.6% at six months. We have purposely made this a conservative estimate as the mortality and morbidity figures are based on treated cases of emergency presentation with faecal/purulent peritonitis. It is assumed that the true adverse outcomes will be higher because a proportion of cases will not reach hospital presentation. For estimates of absenteeism, we again largely used recovery times based on HIC data. This data was however adjusted for lower rates of laparoscopic procedure in LMICs, and also incorporated the higher SSI rates see in LMICs resulting in additional delayed recovery.

Table S6. Data sources for indicators on probabilities and outcomes of scenarios without surgery

| Indicator name | Source | Value | Comment |
| --- | --- | --- | --- |
| *Probability of scenarios after no operation:* | | |  |
| Mild case ($\mathbf{p}_{\mathbf{mild}}$) | GlobalSurg1 and 2^3,15^ for HICs | 10% | Normal appendix (at surgery) in HIC |
| Moderate case ($\mathbf{p}_{\mathbf{mod}}$) | GlobalSurg1 and 2^3,15^ for HICs | 68.5% | Purulent peritonitis (Infection within the abdominal cavity, beyond the walls of the appendix – without perforation) at surgery in HIC |
| Severe case ($\mathbf{p}_{\mathbf{sev}}$) | GlobalSurg1 and 2^3,15^ for HICs | 21.5% | Perforated appendix (perforation of a gangrenous appendix with localised or generalised faecal contamination) at surgery in HIC |
| *Outcomes without operation (*$\boldsymbol{\omega}$***)*** | |  |  |
| Death rates by scenario |  | $\boldsymbol{\omega}_{\mathbf{mild}}\boldsymbol{:}$ LMIC: 0% | Zero mortality in mild cases is a conservative assumption. |
|  | NELA ^47^, GlobalSurg1^15^ | $\boldsymbol{\omega}_{\mathbf{mod}}\boldsymbol{:}$ LMIC: 10% | Mortality of moderate (purulent peritonitis) cases as provided by NELA and validated by GlobalSurg1. |
|  | GenOSept Investigators (2014)^48^ | $\boldsymbol{\omega}_{\boldsymbol{sev}}\boldsymbol{:}$ LMIC: 19% | Mortality of severe cases is proxied by faecal peritonitis. This assumes that patients with gangrenous appendicitis will come to delayed surgery with faecal peritonitis (a best case scenario). |
| Lost working days by scenario | Di Saverio et al (2014)^49^ | $\boldsymbol{\omega}_{\mathbf{mild}}\boldsymbol{:}$ LMIC: 4 | The authors found that laparoscopic and open appendectomy surgery patients had 2.1 and 6.6 days return to daily activities respectively. We estimated 4 as a weighted the average for patients undergoing HIC appendectomy. |
|  | Long et al (2001)^46^ | $\boldsymbol{\omega}_{\mathbf{mod}}\boldsymbol{:}$ LMIC: 15 | A prospective randomised comparison that compared laparoscopic versus open appendectomy in USA. The study found that appendectomy patients have 14.6 days off work or school after the surgery (for both laparoscopic and open appendectomy). The value has been rounded to the nearest whole number. |
|  | Opmeer et al (2010)^45^ | $\boldsymbol{\omega}_{\boldsymbol{sev}}\boldsymbol{:}$ LMIC: 60 | Estimated as the weighted average of lost working days reported in the study among patients undergoing on-demand (50 days) and planned re-laparotomy (70 days) for severe peritonitis in a HIC Opmeer et al (2010). |

### Wage data

Data for annual wages are not available from a single source for all countries and therefore combined with the following procedure. As listed in detail in Table S7, wage data can be retrieved from OECD annual wages, ILO monthly wages or proxied by labor shares in GDP (as provided by the ILO) multiplied with GDP per labor force member. Table S8 provides an overview of the data availability from each source for each country. For the estimation of income losses, we use the wage of the year 2015 as this requires least assumptions regarding the surgical volume, which is only available for the year 2015. If data from several sources is available for the most recent year, we applied the following strategy: priority is given to OECD wage data, if this is not available, ILO wage data is chosen and only if none of these is available, the wage proxy based on labor shares is used. The wage data source, which is used for each country is listed in Table S9. For the OECD countries, all wage indicators are available, so that the alternative measures can be validated against it (Figure S6, Figure S7).

Table S7. Overview wage data

|  | Indicator name | Source | Comment |
| --- | --- | --- | --- |
| $\mathbf{annual} \mathbf{wag}\mathbf{e}_{\mathbf{i},\mathbf{y}}$ | Wage | OECD^18^, World Bank^50^ | Average annual wages for all (at the time) OECD countries as provided by the OECD from 1990-2019 in 2019 int. $ PPP; deflated to 2015 int. $ PPP using GDP Deflator as provided by the World Bank |
|  | Wage | ILO^16^ | Average monthly wages for varying list of countries as provided by ILO from 2008-2019 in 2015 int. $ PPP; multiplied by 12 for average annual wage |
|  | Wage proxy from labor shares in GDP | ILO^17^, World Bank^50^ | Modeled and imputed labor shares in GDP for varying list of countries from 2004 – 2017 as provided by ILO; multiplied by GDP per labor force member (int. $ 2015 PPP) for annual wage proxy |
|  | GDP per labor force member, GDP, population | World Bank^50^ | GDP and GDP per labor force member in int. $ PPP and total population number as provided by the World Bank from 1990-2019 |

Table S8. Overview included wage data sources

| Wage data source for 2015 | Number of countries |
| --- | --- |
| OECD wage | 35 |
| ILO wage | 45 |
| ILO labor share | 92 |
| None | 31 |

Figure S6. Comparison of ILO and OECD wage in 2015


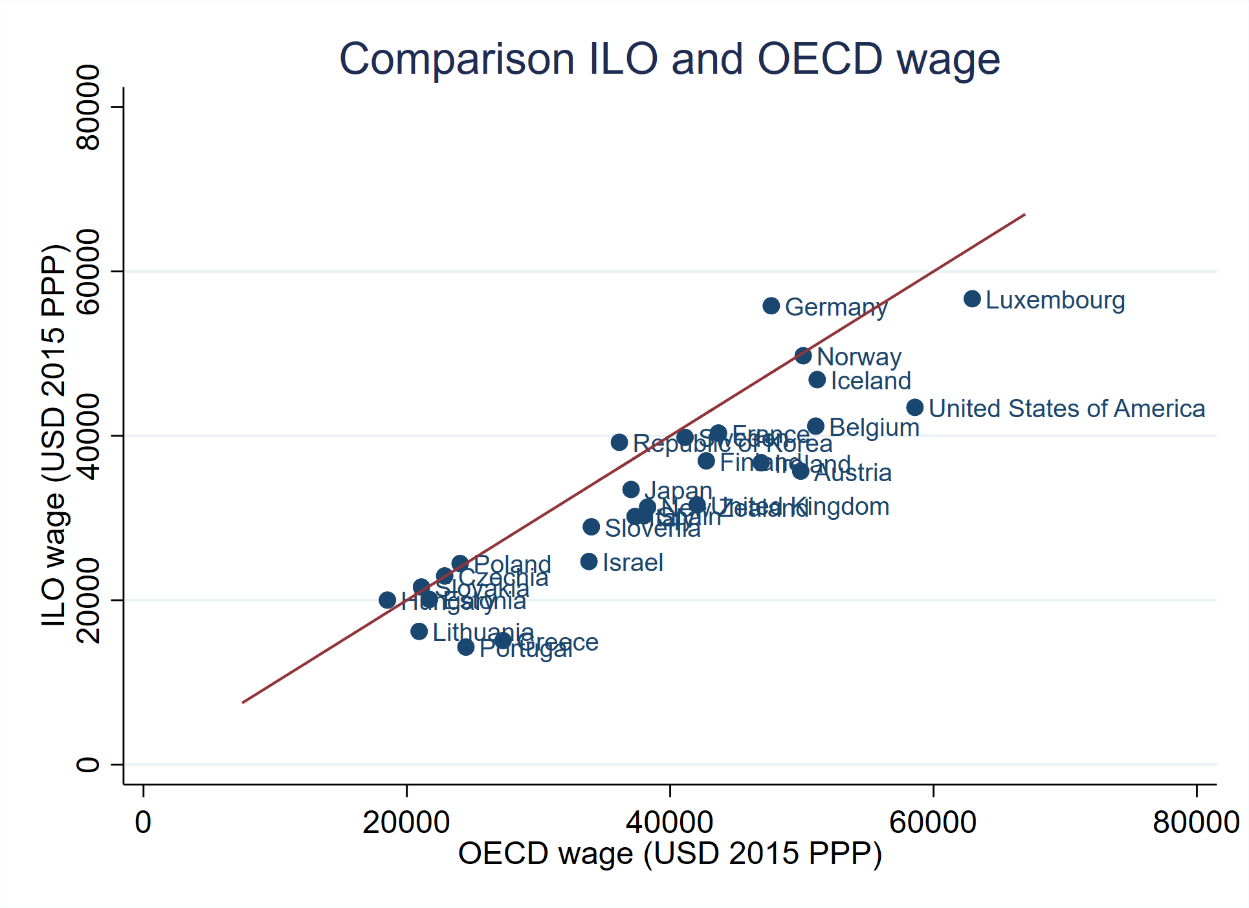


Figure S7. Comparison of ILO labor share wage proxy and OECD wage in 2015


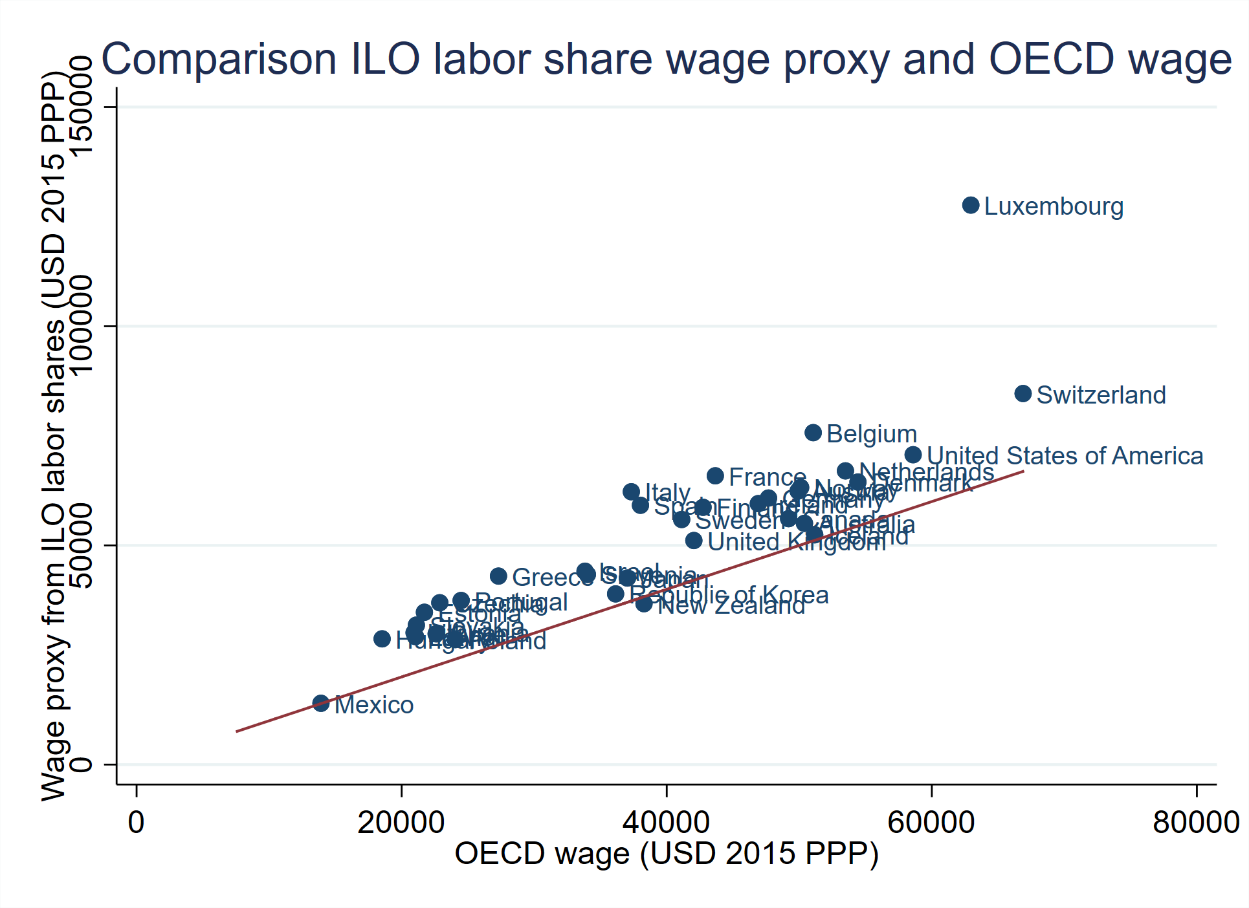


Table S9. Country-wise overview of all inputs and unmet need estimates

| Country | Income group | WHO region | Incidence rate | Surgical volume per 100,000 | Estimated appendectomies | Average annual wage | Data source for wage | GDP in mn USD PPP | Population in thousand | Unmet need in %  (Appr. 1) | Unmet need in %  (Appr. 2) |
| --- | --- | --- | --- | --- | --- | --- | --- | --- | --- | --- | --- |
| Afghanistan | LIC | Eastern Mediterranean | 267 | 692 | 65 | 3,916 | ILO labor share | 71,068 | 34,414 | 86 | 75 |
| Albania | UMIC | Europe | 201 | 1,911 | 89 | 10,592 | ILO wage | 34,052 | 2,881 | 62 | 56 |
| Algeria | UMIC | Africa | 278 | 3,823 | 178 | 18,555 | ILO labor share | 430,121 | 39,728 | 24 | 36 |
| Angola | UMIC | Africa | 280 | 1,349 | 63 | 7,160 | ILO labor share | 150,081 | 27,884 | 73 | 78 |
| Argentina | UMIC | Americas | 283 | 6,527 | 304 | 19,182 | ILO labor share | 580,542 | 43,132 | 0 | 0 |
| Armenia | LMIC | Europe | 222 | 4,343 | 326 | 8,202 | ILO wage | 32,337 | 2,926 | 13 | 0 |
| Australia | HIC | Western Pacific | 255 | 28,823 | 362 | 50,390 | OECD wage | 1,102,934 | 23,816 | 0 | 0 |
| Austria | HIC | Europe | 358 | 13,648 | 172 | 49,913 | OECD wage | 445,632 | 8,643 | 0 | 0 |
| Azerbaijan | UMIC | Europe | 231 | 1,843 | 86 | 12,732 | ILO wage | 107,583 | 9,649 | 63 | 63 |
| Bahamas | HIC | Americas | 284 | 7,386 | 93 | 40,082 | ILO labor share | 12,992 | 374 | 0 | 0 |
| Bahrain | HIC | Eastern Mediterranean | 284 | 3,790 | 48 | 24,627 | ILO labor share | 62,394 | 1,372 | 0 | 0 |
| Bangladesh | LMIC | South-East Asia | 1,454 | 338 | 25 | 3,645 | ILO labor share | 509,716 | 156,256 | 93 | 98 |
| Barbados | HIC | Americas | 258 | 6,763 | 85 | 18,254 | ILO labor share | 4,281 | 285 | 0 | 0 |
| Belarus | UMIC | Europe | 271 | 18,394 | 857 | 15,364 | ILO labor share | 147,625 | 9,490 | 0 | 0 |
| Belgium | HIC | Europe | 300 | 20,888 | 263 | 51,053 | OECD wage | 538,955 | 11,274 | 0 | 0 |
| Belize | UMIC | Americas | 291 | 1,922 | 90 | 10,189 | ILO wage | 2,507 | 361 | 62 | 69 |
| Benin | LIC | Africa | 138 | 332 | 31 | 3,617 | ILO labor share | 31,059 | 10,576 | 93 | 77 |
| Bhutan | LMIC | South-East Asia | 1,396 | 2,597 | 195 | 9,034 | ILO labor share | 6,692 | 728 | 48 | 86 |
| Bolivia | LMIC | Americas | 771 | 2,132 | 160 | 24,713 | ILO wage | 82,952 | 10,870 | 57 | 79 |
| Bosnia and Herzegovina | UMIC | Europe | 177 | 4,550 | 212 | 10,472 | ILO wage | 42,136 | 3,429 | 9 | 0 |
| Botswana | UMIC | Africa | 351 | 4,348 | 203 | 18,010 | ILO labor share | 30,856 | 2,121 | 13 | 42 |
| Brazil | UMIC | Americas | 123 | 13,674 | 637 | 9,438 | ILO wage | 2,748,398 | 204,472 | 0 | 0 |
| Brunei Darussalam | HIC | Western Pacific | 447 | 6,032 | 76 | 69,177 | ILO labor share | 27,484 | 415 | 0 | 0 |
| Bulgaria | UMIC | Europe | 179 | 7,092 | 330 | 13,036 | ILO wage | 132,560 | 7,178 | 0 | 0 |
| Burkina Faso | LIC | Africa | 137 | 300 | 28 | 2,704 | ILO labor share | 33,509 | 18,111 | 94 | 79 |
| Burundi | LIC | Africa | 162 | 249 | 24 | 1,006 | ILO labor share | 7,451 | 10,160 | 95 | 85 |
| Cabo Verde | LMIC | Africa | 159 | 1,881 | 141 | 7,709 | ILO wage | 3,274 | 525 | 62 | 11 |
| Cambodia | LMIC | Western Pacific | 290 | 817 | 61 | 5,085 | ILO wage | 51,404 | 15,521 | 84 | 79 |
| Cameroon | LMIC | Africa | 144 | 738 | 55 | 2,920 | ILO labor share | 78,545 | 23,298 | 85 | 61 |
| Canada | HIC | Americas | 175 | 9,314 | 117 | 49,195 | OECD wage | 1,647,308 | 35,703 | 0 | 0 |
| Central African Republic | LIC | Africa | 260 | 162 | 15 | 1,127 | ILO labor share | 3,495 | 4,493 | 97 | 94 |
| Chad | LIC | Africa | 130 | 47 | 4 | 2,836 | ILO labor share | 26,570 | 14,111 | 99 | 97 |
| Chile | HIC | Americas | 339 | 6,682 | 84 | 22,602 | OECD wage | 402,456 | 17,969 | 0 | 0 |
| China | UMIC | Western Pacific | 173 | 2,691 | 125 | 14,572 | ILO wage | 16,464,849 | 1,371,220 | 46 | 28 |
| Colombia | UMIC | Americas | 422 | 27,385 | 1,276 | 13,947 | ILO labor share | 607,997 | 47,521 | 0 | 0 |
| Comoros | LIC | Africa | 178 | 672 | 64 | 4,109 | ILO labor share | 2,253 | 777 | 87 | 64 |
| Congo | LMIC | Africa | 259 | 676 | 51 | 6,962 | ILO labor share | 23,740 | 4,856 | 86 | 80 |
| Costa Rica | UMIC | Americas | 406 | 3,707 | 173 | 16,926 | ILO wage | 86,247 | 4,848 | 26 | 57 |
| Croatia | HIC | Europe | 224 | 6,162 | 77 | 38,222 | ILO labor share | 102,491 | 4,204 | 0 | 0 |
| Cyprus | HIC | Europe | 197 | 3,037 | 38 | 2,603 | ILO wage | 29,164 | 1,161 | 0 | 0 |
| Czechia | HIC | Europe | 220 | 6,247 | 79 | 22,876 | OECD wage | 373,453 | 10,546 | 0 | 0 |
| Côte d'Ivoire | LMIC | Africa | 134 | 896 | 67 | 4,094 | ILO labor share | 106,745 | 23,226 | 82 | 50 |
| DR Congo | LIC | Africa | 298 | 198 | 19 | 852 | ILO labor share | 54,403 | 76,245 | 96 | 94 |
| Denmark | HIC | Europe | 266 | 9,926 | 125 | 54,427 | OECD wage | 297,287 | 5,683 | 0 | 0 |
| Djibouti | LMIC | Eastern Mediterranean | 168 | 979 | 73 | 4,899 | ILO labor share | 4,074 | 914 | 80 | 56 |
| Dominican Republic | UMIC | Americas | 294 | 4,384 | 204 | 6,157 | ILO wage | 148,319 | 10,282 | 12 | 31 |
| Ecuador | UMIC | Americas | 814 | 1,501 | 70 | 13,404 | ILO labor share | 185,652 | 16,212 | 70 | 91 |
| Egypt | LMIC | Eastern Mediterranean | 260 | 2,039 | 153 | 7,089 | ILO wage | 748,147 | 92,443 | 59 | 41 |
| El Salvador | LMIC | Americas | 398 | 3,773 | 283 | 7,013 | ILO wage | 50,674 | 6,325 | 25 | 29 |
| Equatorial Guinea | UMIC | Africa | 295 | 3,754 | 175 | 38,894 | ILO labor share | 31,315 | 1,169 | 25 | 41 |
| Estonia | HIC | Europe | 263 | 9,703 | 122 | 21,717 | OECD wage | 38,976 | 1,315 | 0 | 0 |
| Eswatini | LMIC | Africa | 309 | 3,186 | 239 | 12,594 | ILO labor share | 8,345 | 1,104 | 36 | 23 |
| Ethiopia | LIC | Africa | 39 | 249 | 24 | 1,500 | ILO labor share | 152,328 | 100,835 | 95 | 39 |
| Fiji | UMIC | Western Pacific | 118 | 2,309 | 108 | 13,740 | ILO labor share | 10,460 | 869 | 54 | 9 |
| Finland | HIC | Europe | 240 | 11,346 | 143 | 42,742 | OECD wage | 245,537 | 5,480 | 0 | 0 |
| France | HIC | Europe | 223 | 9,069 | 114 | 43,664 | OECD wage | 2,863,821 | 66,548 | 0 | 0 |
| Gabon | UMIC | Africa | 270 | 2,654 | 124 | 15,798 | ILO labor share | 30,395 | 1,948 | 47 | 54 |
| Gambia | LIC | Africa | 148 | 339 | 32 | 2,440 | ILO labor share | 3,832 | 2,086 | 93 | 78 |
| Georgia | UMIC | Europe | 197 | 5,097 | 237 | 11,187 | ILO wage | 42,178 | 3,725 | 0 | 0 |
| Germany | HIC | Europe | 304 | 9,354 | 118 | 47,680 | OECD wage | 4,076,146 | 81,687 | 0 | 0 |
| Ghana | LMIC | Africa | 154 | 951 | 71 | 6,225 | ILO wage | 102,270 | 27,849 | 81 | 54 |
| Greece | HIC | Europe | 204 | 7,191 | 90 | 27,306 | OECD wage | 309,238 | 10,821 | 0 | 0 |
| Guatemala | LMIC | Americas | 514 | 1,423 | 107 | 6,985 | ILO wage | 121,046 | 15,567 | 72 | 79 |
| Guinea | LIC | Africa | 136 | 259 | 25 | 3,335 | ILO labor share | 20,469 | 11,432 | 95 | 82 |
| Guinea-Bissau | LIC | Africa | 143 | 431 | 41 | 1,580 | ILO labor share | 2,782 | 1,737 | 91 | 71 |
| Guyana | UMIC | Americas | 293 | 2,453 | 114 | 17,970 | ILO labor share | 8,389 | 767 | 51 | 61 |
| Haiti | LIC | Americas | 218 | 609 | 58 | 3,025 | ILO labor share | 25,315 | 10,696 | 88 | 74 |
| Honduras | LMIC | Americas | 519 | 2,334 | 175 | 6,879 | ILO wage | 44,434 | 9,113 | 53 | 66 |
| Hungary | HIC | Europe | 187 | 14,048 | 177 | 18,524 | OECD wage | 257,643 | 9,843 | 0 | 0 |
| Iceland | HIC | Europe | 279 | 9,249 | 116 | 51,165 | OECD wage | 16,642 | 331 | 0 | 0 |
| India | LMIC | South-East Asia | 121 | 734 | 55 | 7,280 | ILO labor share | 6,669,901 | 1,310,152 | 85 | 55 |
| Indonesia | LMIC | South-East Asia | 69 | 1,394 | 105 | 4,325 | ILO wage | 2,454,679 | 258,383 | 72 | 0 |
| Iran | UMIC | Eastern Mediterranean | 225 | 4,230 | 197 | 14,220 | ILO labor share | 873,893 | 78,492 | 15 | 12 |
| Iraq | UMIC | Eastern Mediterranean | 288 | 2,008 | 94 | 11,858 | ILO labor share | 369,498 | 35,572 | 60 | 68 |
| Ireland | HIC | Europe | 234 | 2,996 | 38 | 46,909 | OECD wage | 331,912 | 4,702 | 0 | 0 |
| Israel | HIC | Europe | 239 | 4,783 | 60 | 33,833 | OECD wage | 311,611 | 8,380 | 0 | 0 |
| Italy | HIC | Europe | 162 | 8,254 | 104 | 37,329 | OECD wage | 2,407,093 | 60,731 | 0 | 0 |
| Jamaica | UMIC | Americas | 289 | 3,839 | 179 | 12,897 | ILO labor share | 24,556 | 2,891 | 23 | 38 |
| Japan | HIC | Western Pacific | 303 | 8,909 | 112 | 37,028 | OECD wage | 5,070,943 | 127,141 | 0 | 0 |
| Jordan | UMIC | Eastern Mediterranean | 296 | 2,473 | 115 | 16,389 | ILO labor share | 91,222 | 9,267 | 51 | 61 |
| Kazakhstan | UMIC | Europe | 233 | 4,459 | 208 | 13,255 | ILO wage | 337,179 | 17,543 | 11 | 11 |
| Kenya | LMIC | Africa | 55 | 824 | 62 | 3,402 | ILO labor share | 156,382 | 47,878 | 84 | 0 |
| Kuwait | HIC | Eastern Mediterranean | 295 | 6,775 | 85 | 26,442 | ILO labor share | 194,233 | 3,836 | 0 | 0 |
| Kyrgyzstan | LMIC | Europe | 243 | 3,045 | 228 | 8,016 | ILO wage | 25,385 | 5,957 | 39 | 6 |
| Lao PDR | LMIC | Western Pacific | 319 | 601 | 45 | 6,041 | ILO labor share | 42,039 | 6,741 | 88 | 86 |
| Latvia | HIC | Europe | 276 | 18,404 | 231 | 21,042 | OECD wage | 50,851 | 1,978 | 0 | 0 |
| Lebanon | UMIC | Eastern Mediterranean | 282 | 5,444 | 254 | 23,436 | ILO labor share | 102,458 | 6,533 | 0 | 10 |
| Lesotho | LMIC | Africa | 294 | 1,104 | 83 | 5,117 | ILO labor share | 5,497 | 2,059 | 78 | 72 |
| Liberia | LIC | Africa | 150 | 813 | 77 | 1,415 | ILO labor share | 6,892 | 4,472 | 84 | 49 |
| Libya | UMIC | Eastern Mediterranean | 290 | 3,943 | 184 | 14,945 | ILO labor share | 63,446 | 6,418 | 21 | 37 |
| Lithuania | HIC | Europe | 291 | 10,189 | 128 | 20,935 | OECD wage | 84,625 | 2,905 | 0 | 0 |
| Luxembourg | HIC | Europe | 302 | 10,054 | 126 | 62,944 | OECD wage | 61,572 | 570 | 0 | 0 |
| Madagascar | LIC | Africa | 161 | 212 | 20 | 2,577 | ILO wage | 32,776 | 24,234 | 96 | 88 |
| Malawi | LIC | Africa | 173 | 367 | 35 | 671 | ILO labor share | 12,682 | 16,745 | 93 | 80 |
| Malaysia | UMIC | Western Pacific | 256 | 4,329 | 202 | 16,028 | ILO wage | 711,234 | 30,271 | 13 | 21 |
| Maldives | UMIC | South-East Asia | 227 | 6,442 | 300 | 13,968 | ILO labor share | 7,738 | 455 | 0 | 0 |
| Mali | LIC | Africa | 142 | 466 | 44 | 3,508 | ILO wage | 36,118 | 17,439 | 91 | 69 |
| Malta | HIC | Europe | 184 | 12,851 | 162 | 42,959 | ILO labor share | 17,101 | 445 | 0 | 0 |
| Mauritania | LMIC | Africa | 151 | 609 | 46 | 8,100 | ILO labor share | 17,996 | 4,046 | 88 | 70 |
| Mauritius | UMIC | Africa | 208 | 2,054 | 96 | 12,738 | ILO wage | 24,280 | 1,263 | 59 | 54 |
| Mexico | UMIC | Americas | 267 | 1,282 | 60 | 13,909 | OECD wage | 2,090,411 | 121,858 | 74 | 78 |
| Mongolia | LMIC | Western Pacific | 363 | 5,168 | 388 | 10,627 | ILO wage | 29,251 | 2,998 | 0 | 0 |
| Montenegro | UMIC | Europe | 190 | 4,312 | 201 | 18,202 | ILO wage | 10,446 | 622 | 14 | 0 |
| Morocco | LMIC | Eastern Mediterranean | 261 | 746 | 56 | 10,168 | ILO labor share | 245,549 | 34,664 | 85 | 79 |
| Mozambique | LIC | Africa | 189 | 296 | 28 | 1,230 | ILO labor share | 27,904 | 27,042 | 94 | 85 |
| Myanmar | LMIC | South-East Asia | 265 | 644 | 48 | 3,925 | ILO wage | 202,381 | 52,681 | 87 | 82 |
| Namibia | UMIC | Africa | 311 | 4,512 | 210 | 14,049 | ILO labor share | 20,683 | 2,315 | 10 | 32 |
| Nepal | LIC | South-East Asia | 874 | 198 | 19 | 1,887 | ILO labor share | 68,198 | 27,015 | 96 | 98 |
| Netherlands | HIC | Europe | 231 | 9,428 | 119 | 53,483 | OECD wage | 885,011 | 16,940 | 0 | 0 |
| New Zealand | HIC | Western Pacific | 263 | 6,099 | 77 | 38,285 | OECD wage | 178,391 | 4,609 | 0 | 0 |
| Nicaragua | LMIC | Americas | 444 | 4,585 | 344 | 7,014 | ILO labor share | 32,175 | 6,223 | 8 | 23 |
| Niger | LIC | Africa | 137 | 245 | 23 | 999 | ILO labor share | 22,155 | 20,002 | 95 | 83 |
| Nigeria | LMIC | Africa | 77 | 1,192 | 89 | 10,406 | ILO labor share | 820,655 | 181,137 | 76 | 0 |
| North Macedonia | UMIC | Europe | 186 | 3,845 | 179 | 18,073 | ILO labor share | 29,559 | 2,079 | 23 | 4 |
| Norway | HIC | Europe | 273 | 10,490 | 132 | 50,104 | OECD wage | 320,119 | 5,189 | 0 | 0 |
| Oman | HIC | Eastern Mediterranean | 301 | 2,452 | 31 | 30,911 | ILO labor share | 131,471 | 4,267 | 0 | 0 |
| Pakistan | LMIC | Eastern Mediterranean | 128 | 413 | 31 | 5,700 | ILO wage | 817,026 | 199,427 | 92 | 76 |
| Palestine | LMIC |  | 291 |  |  | 11,059 | ILO wage | 24,707 | 4,270 |  | 0 |
| Panama | UMIC | Americas | 412 | 6,378 | 297 | 16,355 | ILO wage | 108,733 | 3,968 | 0 | 28 |
| Papua New Guinea | LMIC | Western Pacific | 106 | 920 | 69 | 3,868 | ILO labor share | 30,011 | 8,108 | 82 | 35 |
| Paraguay | UMIC | Americas | 263 | 3,991 | 186 | 12,727 | ILO labor share | 74,271 | 6,689 | 20 | 29 |
| Peru | UMIC | Americas | 876 | 2,850 | 133 | 9,959 | ILO labor share | 345,488 | 30,471 | 43 | 85 |
| Philippines | LMIC | Western Pacific | 89 | 1,608 | 121 | 6,996 | ILO wage | 719,327 | 102,113 | 68 | 0 |
| Poland | HIC | Europe | 152 | 5,980 | 75 | 24,046 | OECD wage | 1,036,711 | 37,986 | 0 | 0 |
| Portugal | HIC | Europe | 141 | 8,602 | 108 | 24,487 | OECD wage | 313,168 | 10,358 | 0 | 0 |
| Puerto Rico | HIC |  | 263 |  |  | 66,598 | ILO labor share | 114,192 | 3,473 | 0 | 0 |
| Qatar | HIC | Eastern Mediterranean | 305 | 7,717 | 97 | 42,668 | ILO wage | 250,957 | 2,566 | 0 | 0 |
| Republic of Korea | HIC | Western Pacific | 362 | 3,351 | 42 | 36,143 | OECD wage | 1,907,146 | 51,015 | 0 | 0 |
| Republic of Moldova | LMIC | Europe | 273 | 4,902 | 368 | 9,350 | ILO wage | 26,156 | 2,835 | 2 | 0 |
| Romania | UMIC | Europe | 176 | 4,602 | 214 | 21,106 | ILO labor share | 442,622 | 19,816 | 8 | 0 |
| Russian Federation | UMIC | Europe | 204 | 6,595 | 307 | 24,873 | ILO labor share | 3,454,720 | 144,097 | 0 | 0 |
| Rwanda | LIC | Africa | 190 | 648 | 61 | 1,202 | ILO labor share | 18,920 | 11,369 | 87 | 68 |
| SVG | UMIC | Americas | 258 | 3,778 | 176 | 13,765 | ILO labor share | 1,282 | 109 | 24 | 32 |
| Saint Lucia | UMIC | Americas | 281 | 4,780 | 223 | 15,360 | ILO labor share | 2,446 | 179 | 4 | 21 |
| Samoa | LMIC | Western Pacific | 114 | 844 | 63 | 10,468 | ILO labor share | 1,148 | 194 | 83 | 45 |
| Sao Tome and Principe | LMIC | Africa | 149 | 2,087 | 157 | 5,012 | ILO labor share | 699 | 199 | 58 | 0 |
| Saudi Arabia | HIC | Eastern Mediterranean | 304 | 3,177 | 40 | 44,059 | ILO wage | 1,487,854 | 31,718 | 0 | 0 |
| Senegal | LIC | Africa | 143 | 390 | 37 | 4,078 | ILO labor share | 42,582 | 14,578 | 92 | 74 |
| Serbia | UMIC | Europe | 201 | 4,822 | 225 | 22,081 | ILO labor share | 106,044 | 7,095 | 4 | 0 |
| Sierra Leone | LIC | Africa | 148 | 334 | 32 | 2,043 | ILO labor share | 9,539 | 7,172 | 93 | 79 |
| Singapore | HIC | Western Pacific | 365 | 7,931 | 100 | 51,797 | ILO wage | 477,939 | 5,535 | 0 | 0 |
| Slovakia | HIC | Europe | 215 | 8,760 | 110 | 21,109 | OECD wage | 154,952 | 5,424 | 0 | 0 |
| Slovenia | HIC | Europe | 232 | 7,524 | 95 | 34,012 | OECD wage | 68,362 | 2,064 | 0 | 0 |
| Solomon Islands | LMIC | Western Pacific | 119 | 1,973 | 148 | 2,472 | ILO labor share | 1,499 | 603 | 61 | 0 |
| South Africa | UMIC | Africa | 130 | 4,732 | 220 | 20,574 | ILO labor share | 630,165 | 55,386 | 5 | 0 |
| Spain | HIC | Europe | 222 | 10,029 | 126 | 38,017 | OECD wage | 1,713,640 | 46,445 | 0 | 0 |
| Sri Lanka | LMIC | South-East Asia | 214 | 1,699 | 127 | 5,125 | ILO wage | 221,717 | 20,970 | 66 | 40 |
| Sudan | LMIC | Eastern Mediterranean | 267 | 1,968 | 148 | 4,752 | ILO labor share | 126,205 | 38,903 | 61 | 45 |
| Suriname | UMIC | Americas | 256 | 5,182 | 241 | 15,549 | ILO labor share | 6,344 | 559 | 0 | 6 |
| Sweden | HIC | Europe | 309 | 15,068 | 189 | 41,128 | OECD wage | 482,824 | 9,799 | 0 | 0 |
| Switzerland | HIC | Europe | 285 | 11,190 | 141 | 66,889 | OECD wage | 552,608 | 8,282 | 0 | 0 |
| Tajikistan | LMIC | Europe | 248 | 1,678 | 126 | 2,969 | ILO labor share | 21,702 | 8,454 | 66 | 49 |
| Thailand | UMIC | South-East Asia | 242 | 2,946 | 137 | 9,805 | ILO wage | 1,069,970 | 68,715 | 41 | 43 |
| Timor-Leste | LMIC | South-East Asia | 311 | 845 | 63 | 3,885 | ILO labor share | 3,899 | 1,196 | 83 | 80 |
| Togo | LIC | Africa | 138 | 396 | 37 | 1,654 | ILO labor share | 10,311 | 7,323 | 92 | 73 |
| Tonga | LMIC | Western Pacific | 113 | 3,012 | 226 | 8,156 | ILO labor share | 551 | 101 | 40 | 0 |
| Trinidad and Tobago | HIC | Americas | 250 | 6,744 | 85 | 36,205 | ILO labor share | 38,400 | 1,370 | 0 | 0 |
| Tunisia | LMIC | Eastern Mediterranean | 269 | 3,579 | 268 | 14,474 | ILO labor share | 106,637 | 11,180 | 28 | 0 |
| Turkey | UMIC | Europe | 274 | 11,006 | 513 | 23,334 | ILO labor share | 1,704,106 | 78,529 | 0 | 0 |
| Turkmenistan | UMIC | Europe | 243 | 2,605 | 121 | 16,549 | ILO labor share | 77,202 | 5,565 | 48 | 50 |
| Uganda | LIC | Africa | 167 | 513 | 49 | 2,010 | ILO labor share | 71,768 | 38,225 | 90 | 71 |
| Ukraine | LMIC | Europe | 217 | 5,402 | 405 | 6,529 | ILO wage | 336,113 | 45,154 | 0 | 0 |
| United Arab Emirates | HIC | Eastern Mediterranean | 279 | 7,072 | 89 | 31,308 | ILO labor share | 591,978 | 9,263 | 0 | 0 |
| United Kingdom | HIC | Europe | 183 | 6,921 | 87 | 42,047 | OECD wage | 2,808,960 | 65,116 | 0 | 0 |
| United Republic of Tanzania | LIC | Africa | 162 | 337 | 32 | 1,984 | ILO labor share | 106,599 | 51,483 | 93 | 80 |
| United States of America | HIC | Americas | 157 | 11,113 | 140 | 58,591 | OECD wage | 18,224,704 | 320,635 | 0 | 0 |
| Uruguay | HIC | Americas | 276 | 6,924 | 87 | 12,372 | ILO wage | 62,499 | 3,412 | 0 | 0 |
| Uzbekistan | LMIC | Europe | 248 | 2,584 | 194 | 9,614 | ILO wage | 146,683 | 31,299 | 48 | 22 |
| Vanuatu | LMIC | Western Pacific | 120 | 1,216 | 91 | 4,721 | ILO labor share | 776 | 271 | 76 | 24 |
| Viet Nam | LMIC | Western Pacific | 278 | 1,463 | 110 | 7,505 | ILO wage | 566,960 | 92,677 | 71 | 61 |
| Zambia | LMIC | Africa | 175 | 585 | 44 | 3,924 | ILO labor share | 43,739 | 15,879 | 88 | 75 |
| Zimbabwe | LIC | Africa | 257 | 1,151 | 109 | 17,206 | ILO wage | 39,048 | 13,815 | 77 | 58 |

*Note:* Approach 1 calculates unmet need as the relative difference of surgical volume to the minimum surgical volume proposed by the Lancet Commission on Global Surgery. Approach 2 calculates unmet need as the relative difference of estimated appendectomies (calculated as World Bank-income-group specific share of surgical volume) to the number of appendicitis cases.

## Appendix S3: Additional results and sensibility checks

Figure S8. Country- and region-wise comparison of unmet need according to approach 1 and 2


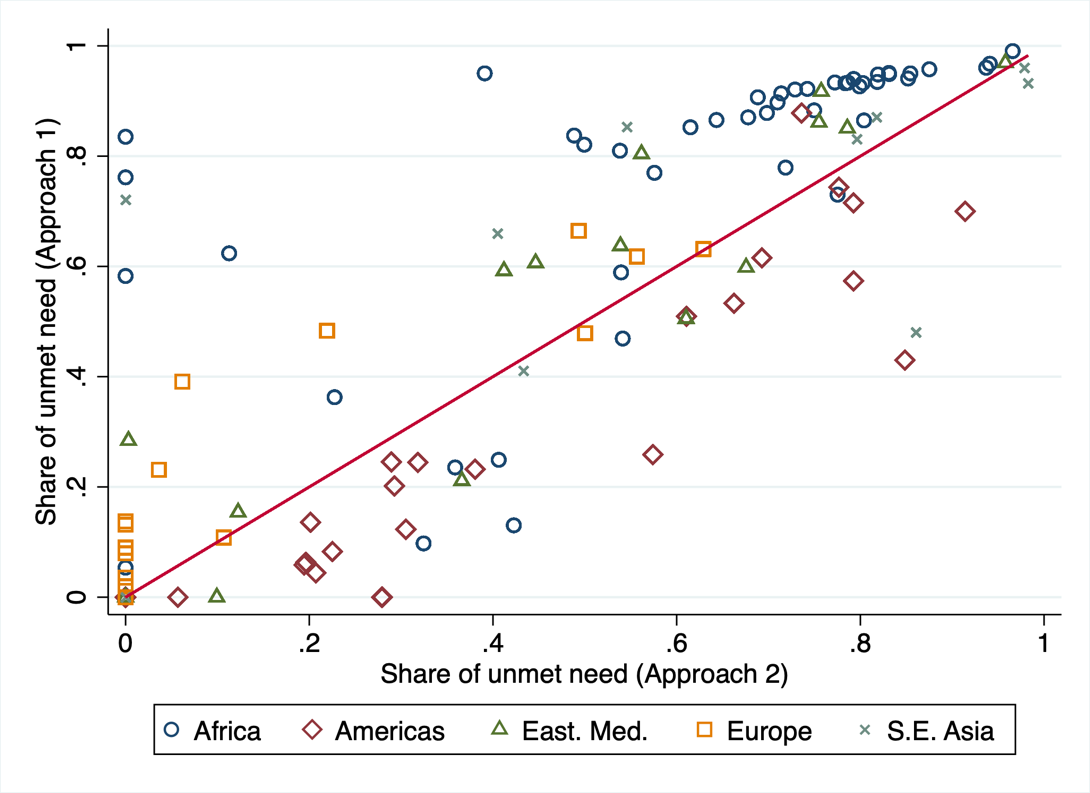


*Note:* Approach 1 calculates unmet need as the relative difference of surgical volume to the minimum surgical volume proposed by the Lancet Commission on Global Surgery. Approach 2 calculates unmet need as the relative difference of estimated appendectomies (calculated as World Bank-income-group specific share of surgical volume) to the number of appendicitis cases.

Figure S9. Unmet need by region and income group


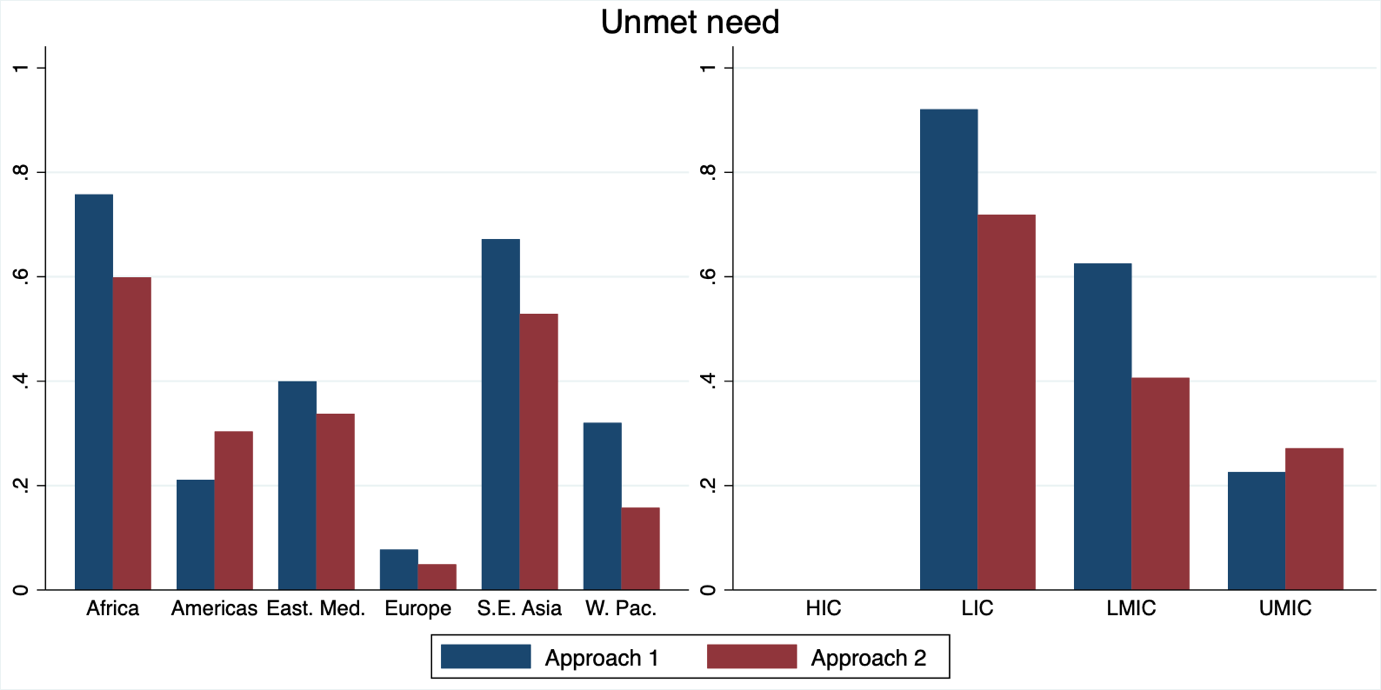


*Note:* Approach 1 calculates unmet need as the relative difference of surgical volume to the minimum surgical volume proposed by the Lancet Commission on Global Surgery. Approach 2 calculates unmet need as the relative difference of estimated appendectomies (calculated as World Bank-income-group specific share of surgical volume) to the number of appendicitis cases.

Figure S10. Expected outcomes per case of appendicitis when not providing care to local standards


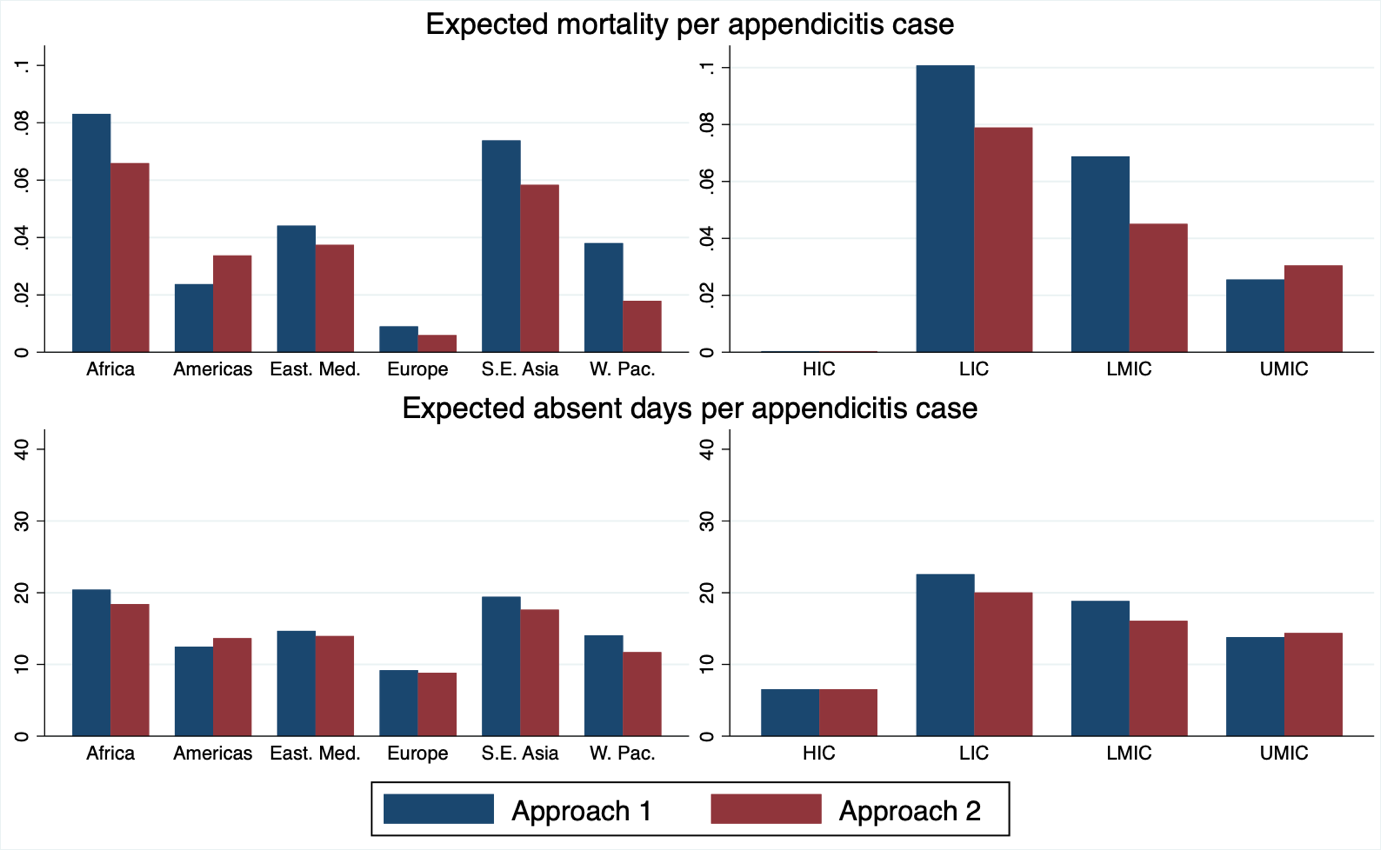


*Note:* Approach 1 calculates unmet need as the relative difference of surgical volume to the minimum surgical volume proposed by the Lancet Commission on Global Surgery. Approach 2 calculates unmet need as the relative difference of estimated appendectomies (calculated as World Bank-income-group specific share of surgical volume) to the number of appendicitis cases.

Table S10. Estimates excess mortality due to unmet surgical need

|  | **Africa** | **Americas** | **Eastern Med.** | **Europe** | **South-East Asia** | **Western Pacific** | **World** |
| --- | --- | --- | --- | --- | --- | --- | --- |
| Countries | 44 | 29 | 18 | 50 | 10 | 19 | 170 |
| No surgery to local standard (1) | 8,299 | 2,663 | 3,841 | 888 | 7,277 | 4,356 | 4,185 |
|  | (2,826) | (3,113) | (3,868) | (1,968) | (3,243) | (3,899) | (4,129) |
| No surgery to local standard (2) | 6,522 | 3,642 | 3,366 | 564 | 6,301 | 2,204 | 3,448 |
|  | (2,943) | (3,532) | (3,418) | (1,643) | (4,001) | (3,071) | (3,697) |
| No surgery to high. standard (1) | 8,372 | 2,718 | 3,889 | 917 | 7,349 | 4,405 | 4,237 |
|  | (2,826) | (3,128) | (3,894) | (1,988) | (3,243) | (3,926) | (4,150) |
| No surgery to high. standard (2) | 6,594 | 3,697 | 3,414 | 593 | 6,373 | 2,254 | 3,500 |
|  | (2,943) | (3,551) | (3,443) | (1,659) | (4,001) | (3,089) | (3,717) |

Mortality per 100,000 cases of appendicitis by region. Depicted as mean (standard deviation). Approach 1 calculates unmet need as the relative difference of surgical volume to the minimum surgical volume proposed by the Lancet Commission on Global Surgery. Approach 2 calculates unmet need as the relative difference of estimated appendectomies (calculated as World Bank-income-group specific share of surgical volume) to the number of appendicitis cases.

Figure S11. Map of mortality-related income losses due to not providing surgery to local standards


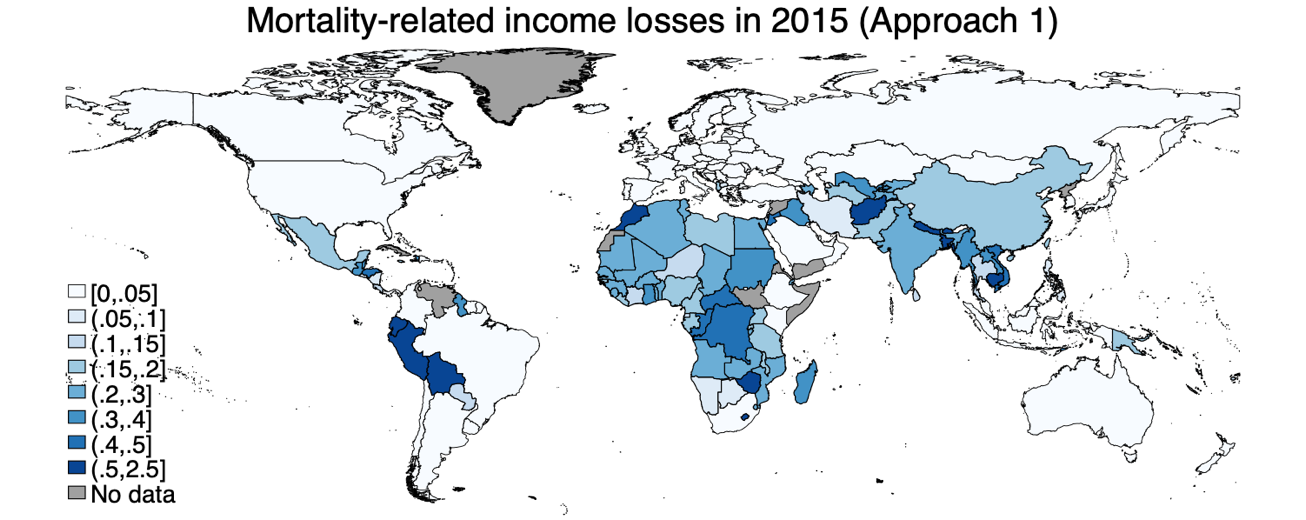


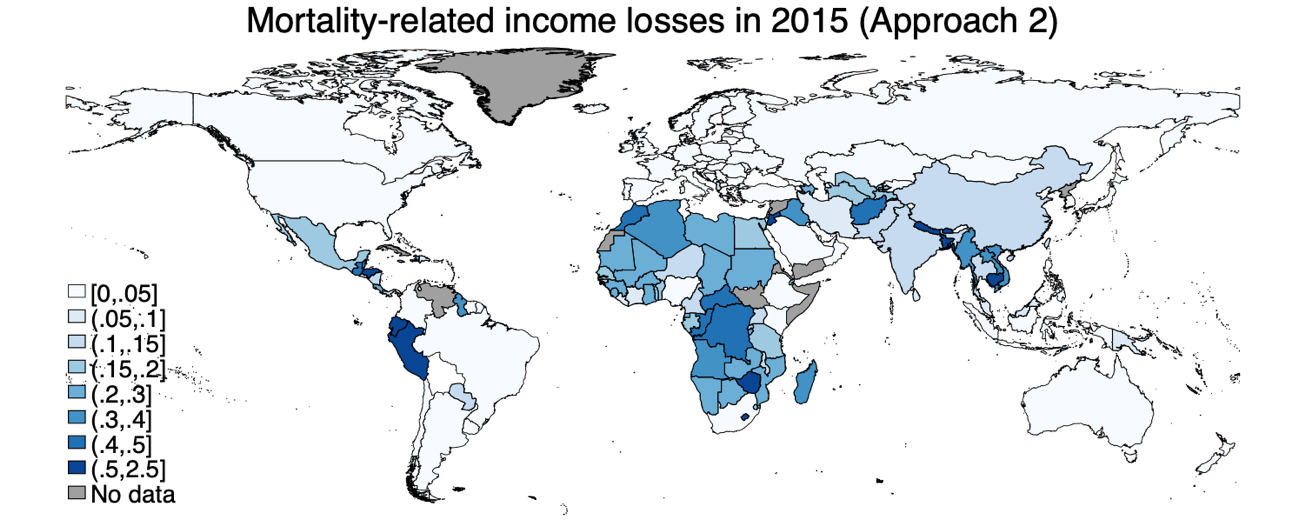


*Note:* Approach 1 calculates unmet need as the relative difference of surgical volume to the minimum surgical volume proposed by the Lancet Commission on Global Surgery. Approach 2 calculates unmet need as the relative difference of estimated appendectomies (calculated as World Bank-income-group specific share of surgical volume) to the number of appendicitis cases.

Figure S12. Map of absenteeism-related income losses due to not providing surgery to local standards


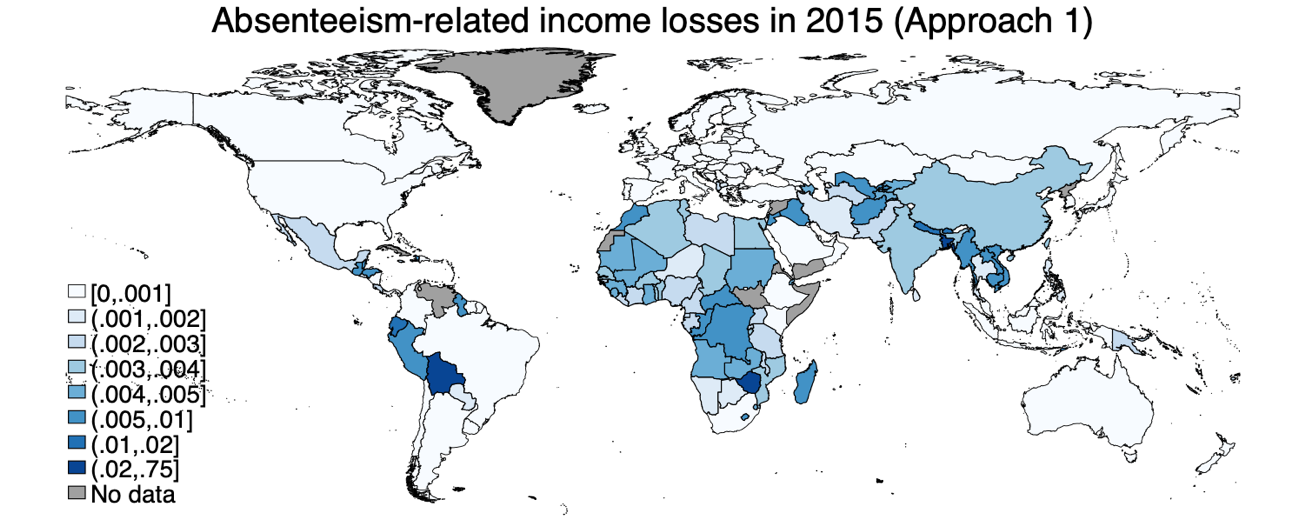


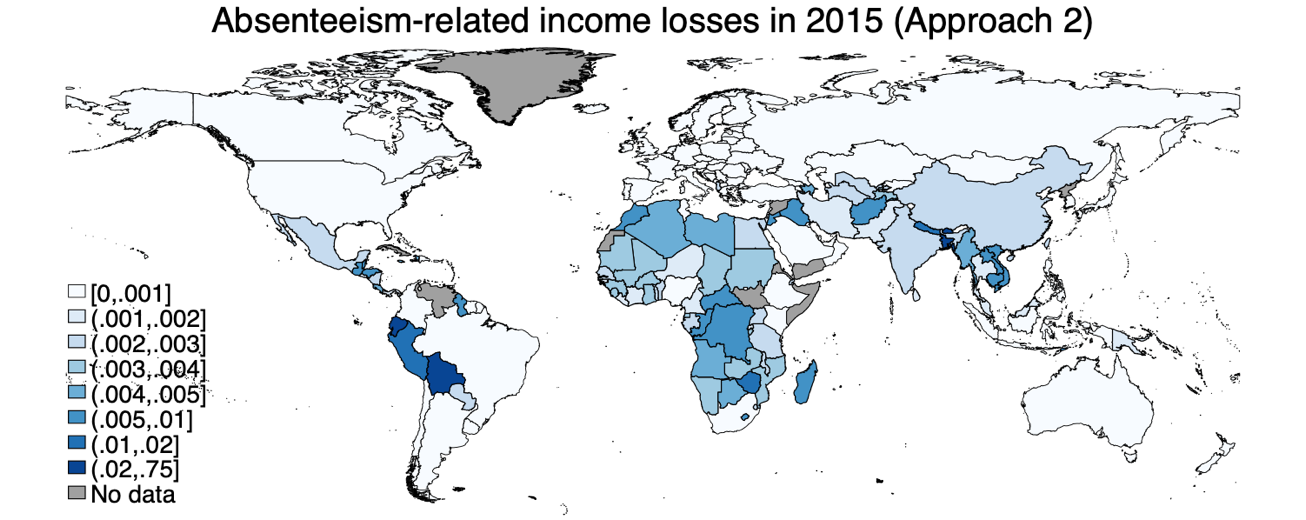


*Note:* Approach 1 calculates unmet need as the relative difference of surgical volume to the minimum surgical volume proposed by the Lancet Commission on Global Surgery. Approach 2 calculates unmet need as the relative difference of estimated appendectomies (calculated as World Bank-income-group specific share of surgical volume) to the number of appendicitis cases.

Figure S13. Total economic burden of not providing surgery to local standard by gender


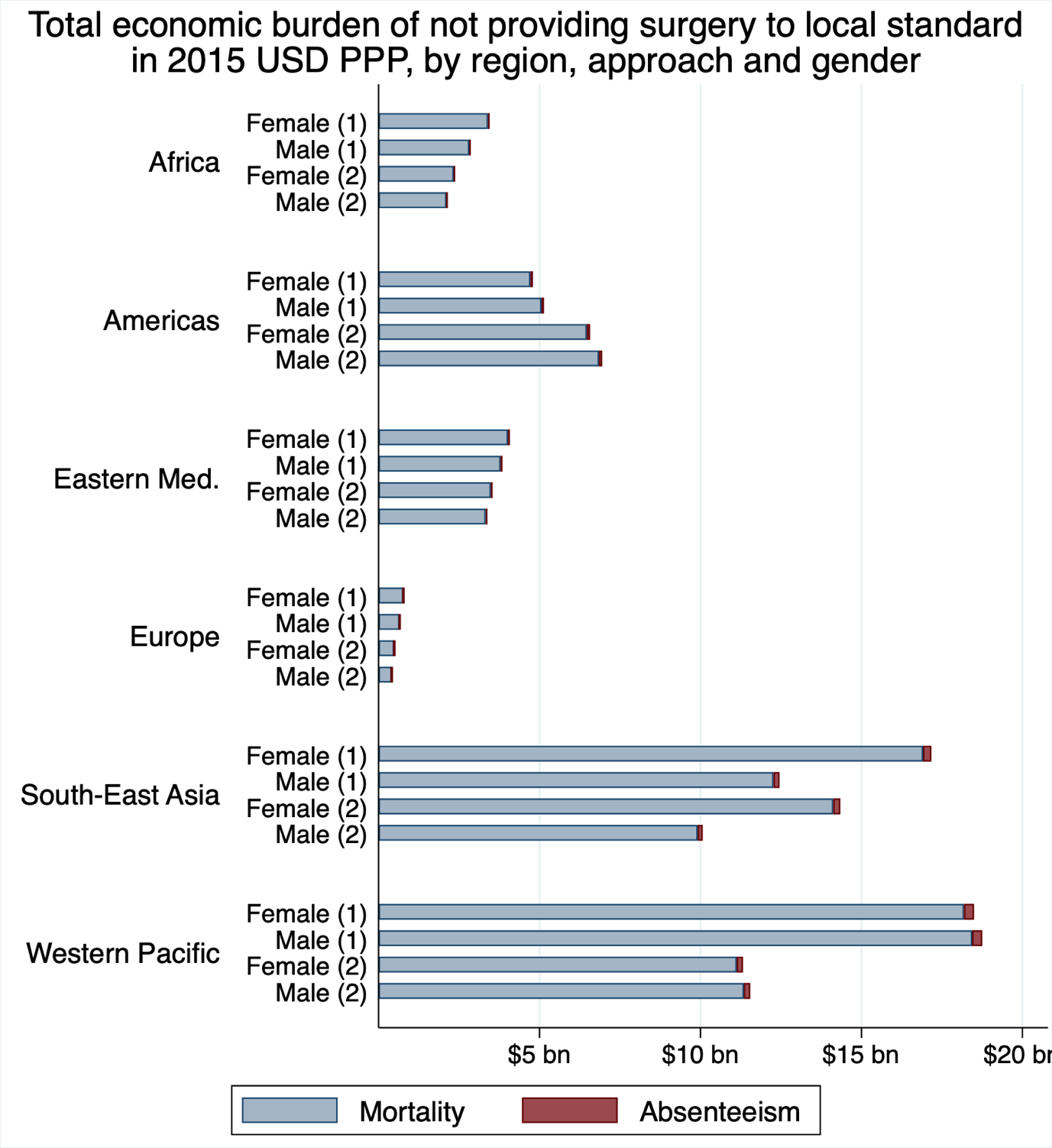


*Note:* All gender differences origin from the gender differences in incidence rates in the GBD estimates. Approach 1 calculates unmet need as the relative difference of surgical volume to the minimum surgical volume proposed by the Lancet Commission on Global Surgery. Approach 2 calculates unmet need as the relative difference of estimated appendectomies (calculated as World Bank-income-group specific share of surgical volume) to the number of appendicitis cases.

Figure S14. Total economic burden of not providing surgery to local standard by gender


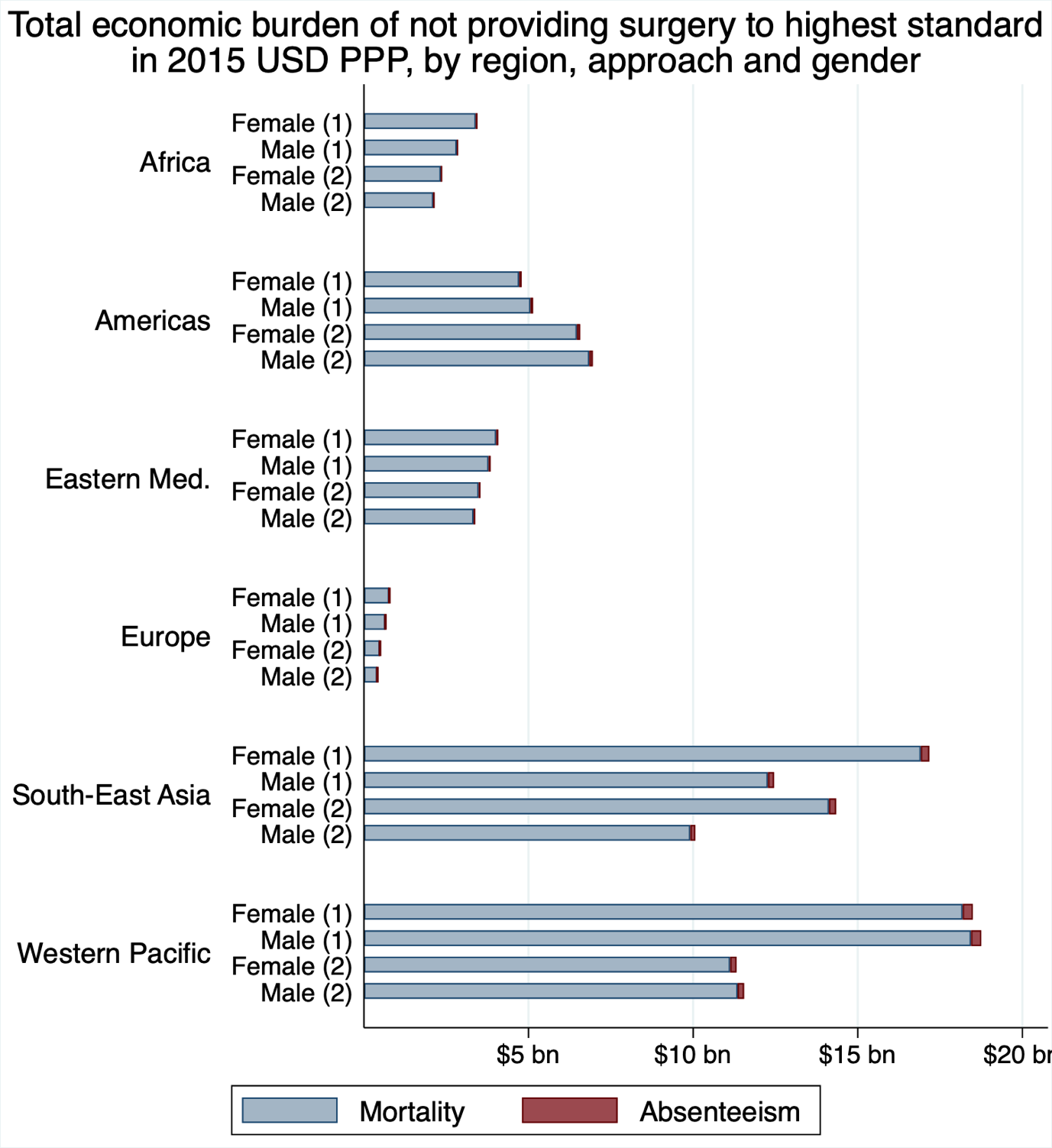


*Note:* All gender differences origin from the gender differences in incidence rates in the GBD estimates. Approach 1 calculates unmet need as the relative difference of surgical volume to the minimum surgical volume proposed by the Lancet Commission on Global Surgery. Approach 2 calculates unmet need as the relative difference of estimated appendectomies (calculated as World Bank-income-group specific share of surgical volume) to the number of appendicitis cases.

Figure S15. Avoidable economic burden in relation to indirect economic burden of appendicitis


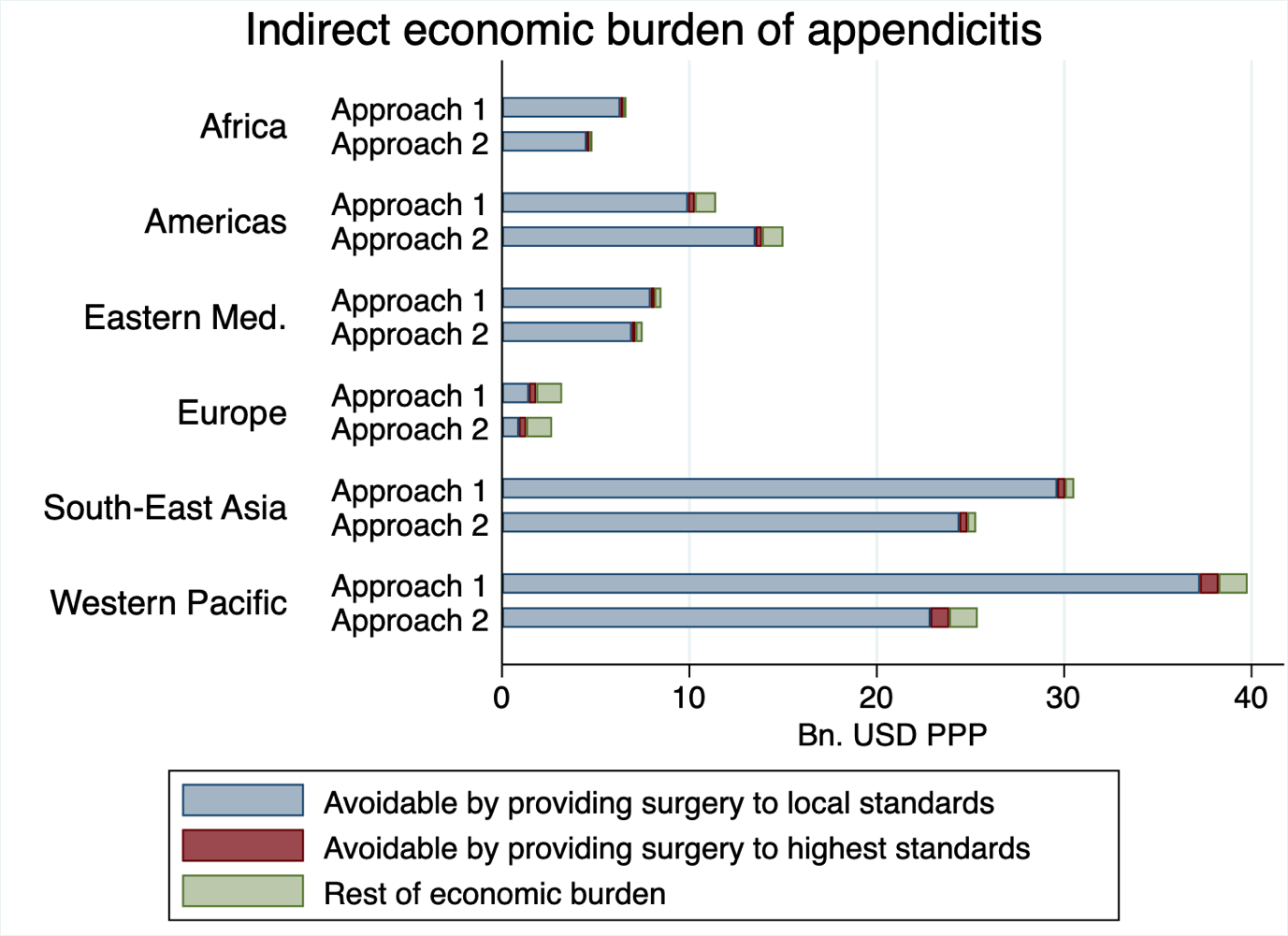


*Note:* The rest of the burden would remain if all people with appendectomies in low- and middle-income countries would retrieve surgery to highest standards. They occur due to adverse events and are modifiable by (global) medical/technological progress in the treatment of appendicitis. Approach 1 calculates unmet need as the relative difference of surgical volume to the minimum surgical volume proposed by the Lancet Commission on Global Surgery. Approach 2 calculates unmet need as the relative difference of estimated appendectomies (calculated as World Bank-income-group specific share of surgical volume) to the number of appendicitis cases.

Figure S16. Total economic burden in % of GDP by approach


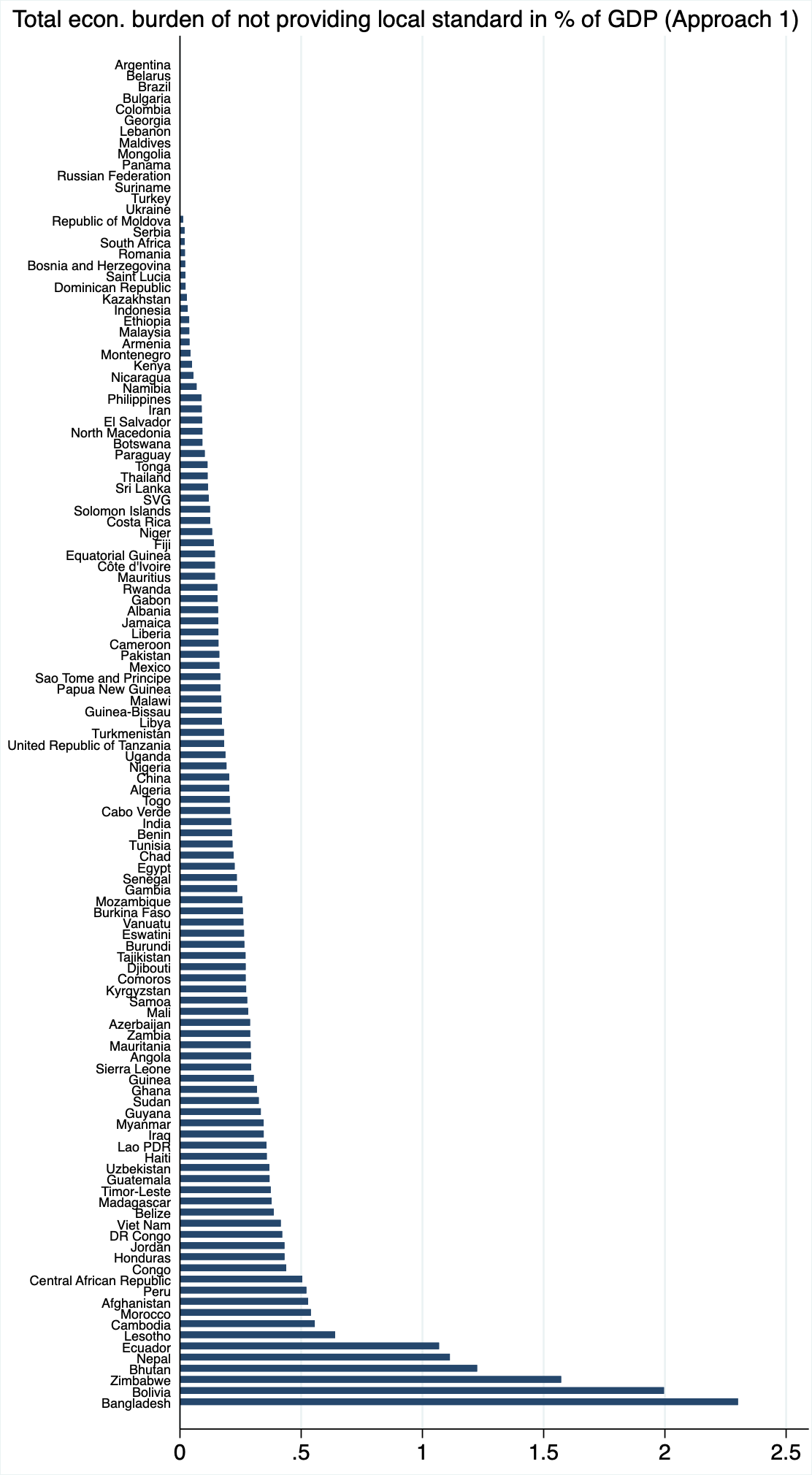


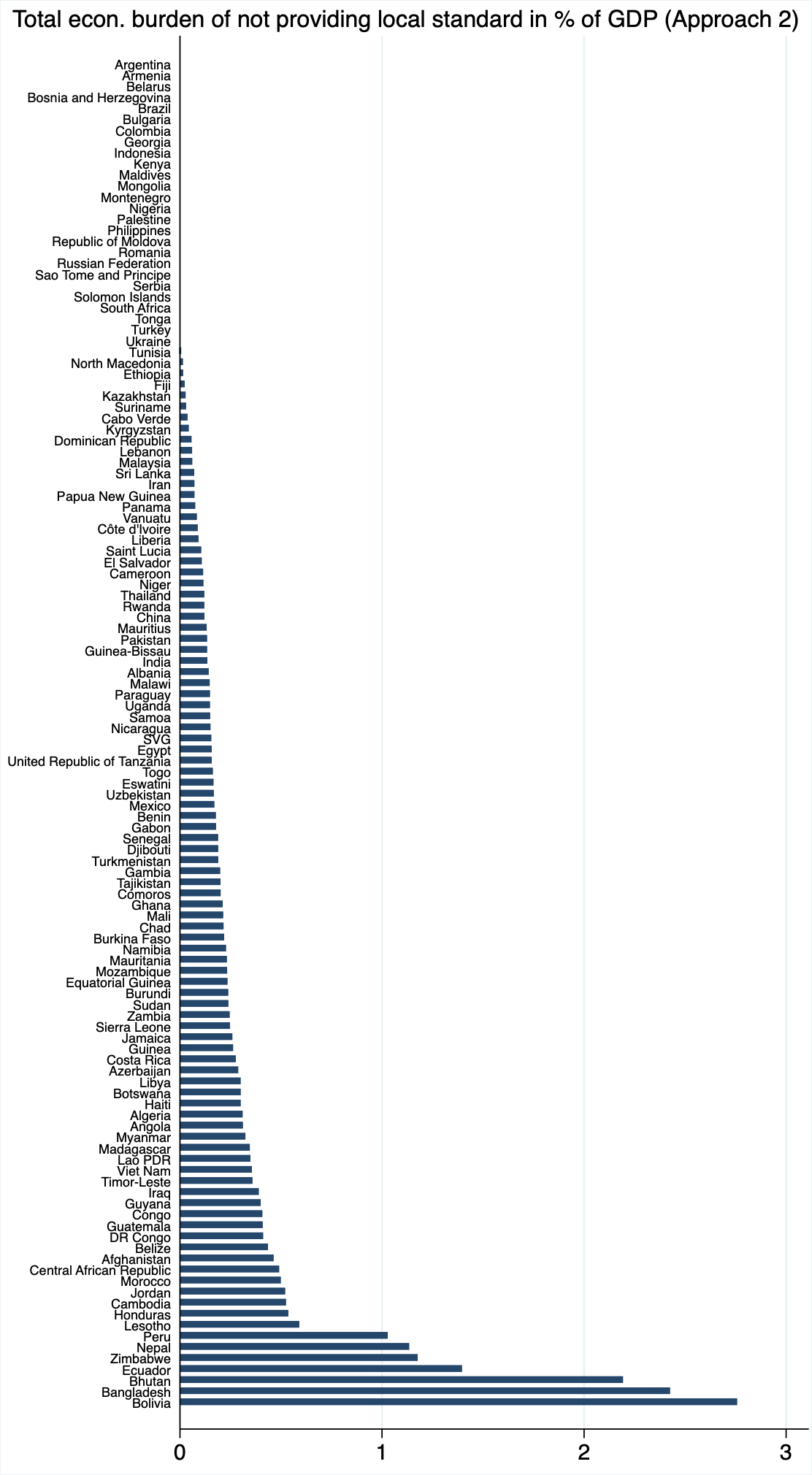


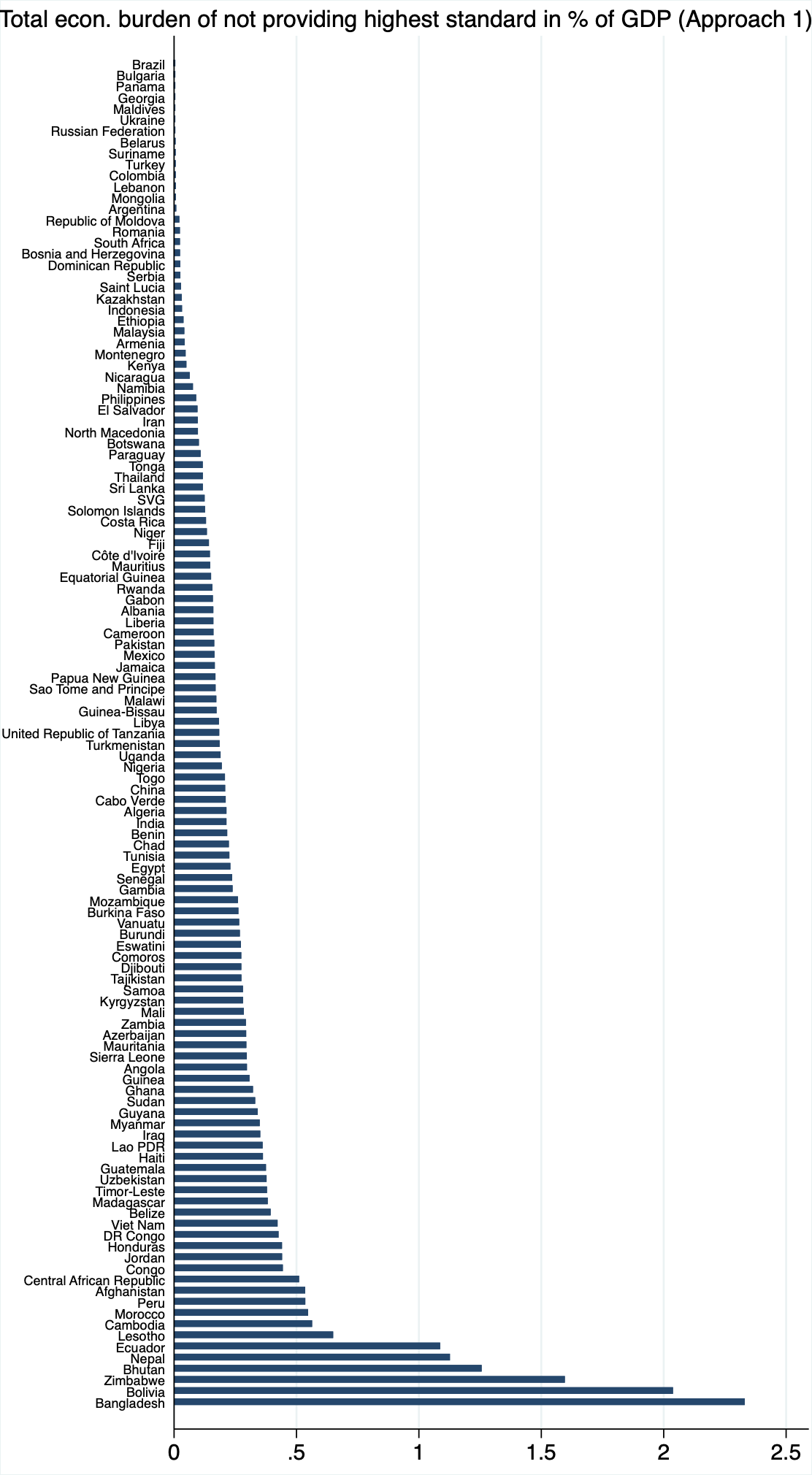


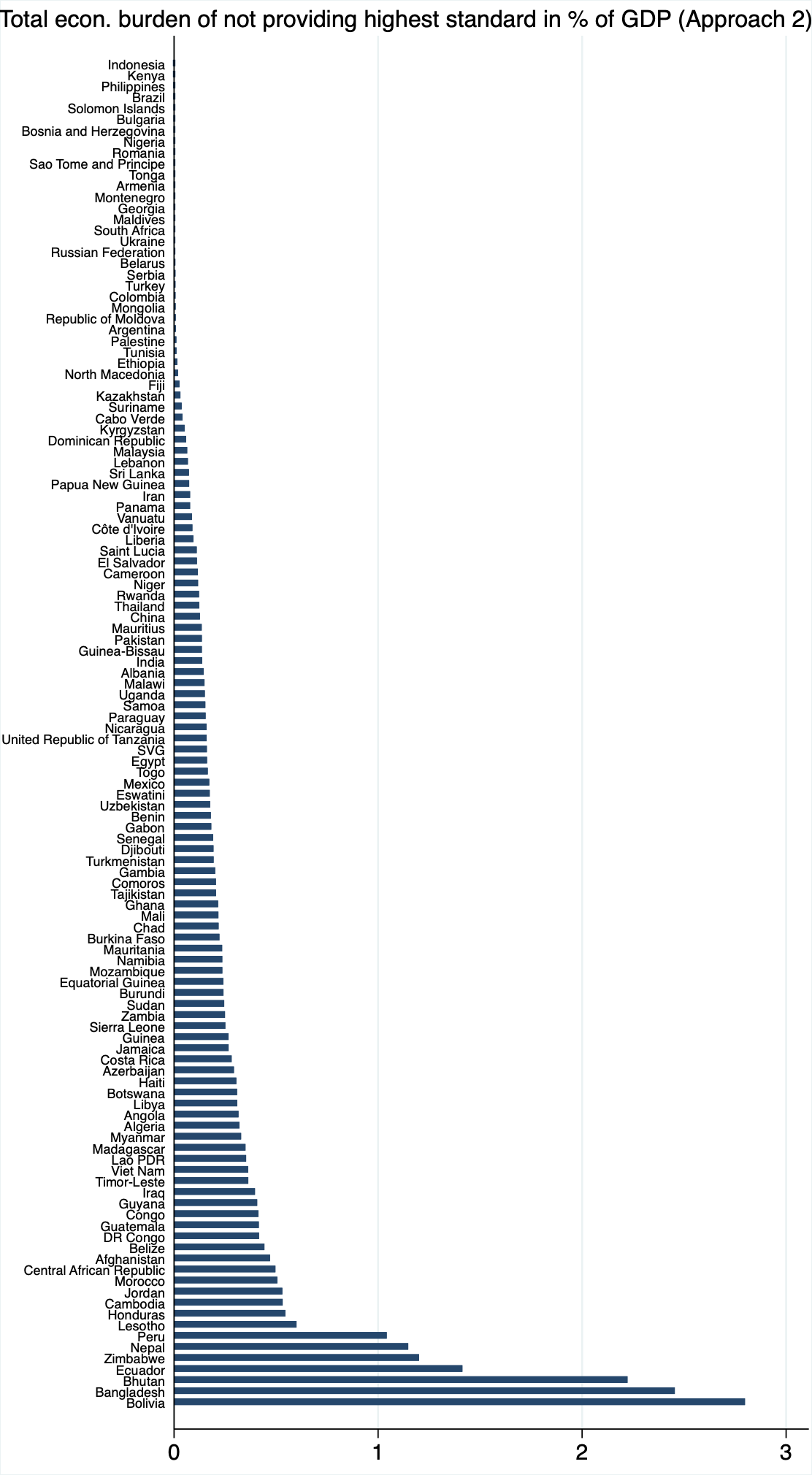


Table S11. Country-wise economic burden as % of GDP

| Country | Income group | WHO region | Mortality-related burden in % of GDP | | | | Absenteeism-related burden in % of GDP | | | | Total economic burden in % of GDP | | | |
| --- | --- | --- | --- | --- | --- | --- | --- | --- | --- | --- | --- | --- | --- | --- |
|  |  |  | Local standard | | Highest standard | | Local standard | | Highest standard | | Local standard | | Highest standard | |
|  |  |  | Appr. 1 | Appr. 2 | Appr. 1 | Appr. 2 | Appr. 1 | Appr. 2 | Appr. 1 | Appr. 2 | Appr. 1 | Appr. 2 | Appr. 1 | Appr. 2 |
| Afghanistan | LIC | Eastern Mediterranean | 0.5208 | 0.4563 | 0.5249 | 0.4604 | 0.0078 | 0.0069 | 0.0109 | 0.0101 | 0.5286 | 0.4632 | 0.5358 | 0.4705 |
| Albania | UMIC | Europe | 0.1549 | 0.1396 | 0.1566 | 0.1413 | 0.0025 | 0.0023 | 0.0040 | 0.0037 | 0.1574 | 0.1419 | 0.1605 | 0.1450 |
| Algeria | UMIC | Africa | 0.2001 | 0.3051 | 0.2058 | 0.3108 | 0.0033 | 0.0050 | 0.0082 | 0.0098 | 0.2034 | 0.3100 | 0.2140 | 0.3205 |
| Angola | UMIC | Africa | 0.2887 | 0.3065 | 0.2913 | 0.3091 | 0.0044 | 0.0046 | 0.0065 | 0.0067 | 0.2931 | 0.3111 | 0.2978 | 0.3158 |
| Argentina | UMIC | Americas | 0.0000 | 0.0000 | 0.0047 | 0.0047 | 0.0000 | 0.0000 | 0.0043 | 0.0043 | 0.0000 | 0.0000 | 0.0090 | 0.0090 |
| Armenia | LMIC | Europe | 0.0388 | 0.0000 | 0.0408 | 0.0020 | 0.0007 | 0.0000 | 0.0024 | 0.0018 | 0.0395 | 0.0000 | 0.0432 | 0.0037 |
| Australia | HIC | Western Pacific | 0.0000 | 0.0000 | 0.0000 | 0.0000 | 0.0000 | 0.0000 | 0.0000 | 0.0000 | 0.0000 | 0.0000 | 0.0000 | 0.0000 |
| Austria | HIC | Europe | 0.0000 | 0.0000 | 0.0000 | 0.0000 | 0.0000 | 0.0000 | 0.0000 | 0.0000 | 0.0000 | 0.0000 | 0.0000 | 0.0000 |
| Azerbaijan | UMIC | Europe | 0.2846 | 0.2836 | 0.2877 | 0.2866 | 0.0045 | 0.0045 | 0.0070 | 0.0069 | 0.2891 | 0.2881 | 0.2946 | 0.2936 |
| Bahamas | HIC | Americas | 0.0000 | 0.0000 | 0.0000 | 0.0000 | 0.0000 | 0.0000 | 0.0000 | 0.0000 | 0.0000 | 0.0000 | 0.0000 | 0.0000 |
| Bahrain | HIC | Eastern Mediterranean | 0.0000 | 0.0000 | 0.0000 | 0.0000 | 0.0000 | 0.0000 | 0.0000 | 0.0000 | 0.0000 | 0.0000 | 0.0000 | 0.0000 |
| Bangladesh | LMIC | South-East Asia | 2.2667 | 2.3887 | 2.2830 | 2.4050 | 0.0348 | 0.0364 | 0.0477 | 0.0493 | 2.3015 | 2.4251 | 2.3308 | 2.4543 |
| Barbados | HIC | Americas | 0.0000 | 0.0000 | 0.0000 | 0.0000 | 0.0000 | 0.0000 | 0.0000 | 0.0000 | 0.0000 | 0.0000 | 0.0000 | 0.0000 |
| Belarus | UMIC | Europe | 0.0000 | 0.0000 | 0.0032 | 0.0032 | 0.0000 | 0.0000 | 0.0030 | 0.0030 | 0.0000 | 0.0000 | 0.0063 | 0.0063 |
| Belgium | HIC | Europe | 0.0000 | 0.0000 | 0.0000 | 0.0000 | 0.0000 | 0.0000 | 0.0000 | 0.0000 | 0.0000 | 0.0000 | 0.0000 | 0.0000 |
| Belize | UMIC | Americas | 0.3811 | 0.4289 | 0.3852 | 0.4330 | 0.0059 | 0.0066 | 0.0093 | 0.0099 | 0.3870 | 0.4355 | 0.3945 | 0.4430 |
| Benin | LIC | Africa | 0.2113 | 0.1748 | 0.2128 | 0.1763 | 0.0031 | 0.0026 | 0.0043 | 0.0038 | 0.2144 | 0.1774 | 0.2171 | 0.1801 |
| Bhutan | LMIC | South-East Asia | 1.2066 | 2.1600 | 1.2235 | 2.1768 | 0.0190 | 0.0326 | 0.0329 | 0.0458 | 1.2257 | 2.1926 | 1.2563 | 2.2226 |
| Bolivia | LMIC | Americas | 1.9653 | 2.7156 | 1.9882 | 2.7386 | 0.0309 | 0.0416 | 0.0497 | 0.0599 | 1.9962 | 2.7572 | 2.0379 | 2.7984 |
| Bosnia and Herzegovina | UMIC | Europe | 0.0211 | 0.0000 | 0.0227 | 0.0016 | 0.0004 | 0.0000 | 0.0018 | 0.0015 | 0.0215 | 0.0000 | 0.0245 | 0.0030 |
| Botswana | UMIC | Africa | 0.0911 | 0.2955 | 0.0958 | 0.3001 | 0.0015 | 0.0047 | 0.0055 | 0.0086 | 0.0927 | 0.3002 | 0.1014 | 0.3088 |
| Brazil | UMIC | Americas | 0.0000 | 0.0000 | 0.0010 | 0.0010 | 0.0000 | 0.0000 | 0.0008 | 0.0008 | 0.0000 | 0.0000 | 0.0018 | 0.0018 |
| Brunei Darussalam | HIC | Western Pacific | 0.0000 | 0.0000 | 0.0000 | 0.0000 | 0.0000 | 0.0000 | 0.0000 | 0.0000 | 0.0000 | 0.0000 | 0.0000 | 0.0000 |
| Bulgaria | UMIC | Europe | 0.0000 | 0.0000 | 0.0013 | 0.0013 | 0.0000 | 0.0000 | 0.0012 | 0.0012 | 0.0000 | 0.0000 | 0.0025 | 0.0025 |
| Burkina Faso | LIC | Africa | 0.2558 | 0.2157 | 0.2577 | 0.2176 | 0.0038 | 0.0032 | 0.0052 | 0.0047 | 0.2596 | 0.2190 | 0.2628 | 0.2222 |
| Burundi | LIC | Africa | 0.2617 | 0.2353 | 0.2635 | 0.2371 | 0.0039 | 0.0035 | 0.0053 | 0.0050 | 0.2655 | 0.2388 | 0.2688 | 0.2421 |
| Cabo Verde | LMIC | Africa | 0.2037 | 0.0368 | 0.2059 | 0.0390 | 0.0032 | 0.0006 | 0.0049 | 0.0025 | 0.2069 | 0.0374 | 0.2108 | 0.0415 |
| Cambodia | LMIC | Western Pacific | 0.5477 | 0.5165 | 0.5521 | 0.5209 | 0.0084 | 0.0080 | 0.0119 | 0.0115 | 0.5561 | 0.5245 | 0.5640 | 0.5324 |
| Cameroon | LMIC | Africa | 0.1565 | 0.1129 | 0.1577 | 0.1141 | 0.0023 | 0.0017 | 0.0033 | 0.0027 | 0.1588 | 0.1146 | 0.1610 | 0.1168 |
| Canada | HIC | Americas | 0.0000 | 0.0000 | 0.0000 | 0.0000 | 0.0000 | 0.0000 | 0.0000 | 0.0000 | 0.0000 | 0.0000 | 0.0000 | 0.0000 |
| Central African Republic | LIC | Africa | 0.4972 | 0.4834 | 0.5006 | 0.4869 | 0.0074 | 0.0073 | 0.0101 | 0.0099 | 0.5046 | 0.4907 | 0.5107 | 0.4968 |
| Chad | LIC | Africa | 0.2181 | 0.2126 | 0.2196 | 0.2141 | 0.0032 | 0.0031 | 0.0043 | 0.0042 | 0.2213 | 0.2158 | 0.2239 | 0.2184 |
| Chile | HIC | Americas | 0.0000 | 0.0000 | 0.0000 | 0.0000 | 0.0000 | 0.0000 | 0.0000 | 0.0000 | 0.0000 | 0.0000 | 0.0000 | 0.0000 |
| China | UMIC | Western Pacific | 0.1999 | 0.1192 | 0.2028 | 0.1221 | 0.0034 | 0.0021 | 0.0060 | 0.0047 | 0.2033 | 0.1213 | 0.2088 | 0.1268 |
| Colombia | UMIC | Americas | 0.0000 | 0.0000 | 0.0036 | 0.0036 | 0.0000 | 0.0000 | 0.0033 | 0.0033 | 0.0000 | 0.0000 | 0.0069 | 0.0069 |
| Comoros | LIC | Africa | 0.2668 | 0.1983 | 0.2689 | 0.2004 | 0.0041 | 0.0031 | 0.0057 | 0.0048 | 0.2709 | 0.2014 | 0.2746 | 0.2052 |
| Congo | LMIC | Africa | 0.4318 | 0.4014 | 0.4351 | 0.4047 | 0.0065 | 0.0061 | 0.0092 | 0.0088 | 0.4383 | 0.4075 | 0.4443 | 0.4135 |
| Costa Rica | UMIC | Americas | 0.1225 | 0.2720 | 0.1257 | 0.2752 | 0.0021 | 0.0044 | 0.0049 | 0.0071 | 0.1246 | 0.2764 | 0.1305 | 0.2823 |
| Croatia | HIC | Europe | 0.0000 | 0.0000 | 0.0000 | 0.0000 | 0.0000 | 0.0000 | 0.0000 | 0.0000 | 0.0000 | 0.0000 | 0.0000 | 0.0000 |
| Cyprus | HIC | Europe | 0.0000 | 0.0000 | 0.0000 | 0.0000 | 0.0000 | 0.0000 | 0.0000 | 0.0000 | 0.0000 | 0.0000 | 0.0000 | 0.0000 |
| Czechia | HIC | Europe | 0.0000 | 0.0000 | 0.0000 | 0.0000 | 0.0000 | 0.0000 | 0.0000 | 0.0000 | 0.0000 | 0.0000 | 0.0000 | 0.0000 |
| Côte d'Ivoire | LMIC | Africa | 0.1421 | 0.0865 | 0.1433 | 0.0876 | 0.0021 | 0.0013 | 0.0030 | 0.0023 | 0.1443 | 0.0878 | 0.1463 | 0.0899 |
| DR Congo | LIC | Africa | 0.4155 | 0.4054 | 0.4184 | 0.4083 | 0.0062 | 0.0060 | 0.0084 | 0.0083 | 0.4217 | 0.4114 | 0.4268 | 0.4166 |
| Denmark | HIC | Europe | 0.0000 | 0.0000 | 0.0000 | 0.0000 | 0.0000 | 0.0000 | 0.0000 | 0.0000 | 0.0000 | 0.0000 | 0.0000 | 0.0000 |
| Djibouti | LMIC | Eastern Mediterranean | 0.2668 | 0.1863 | 0.2690 | 0.1886 | 0.0041 | 0.0029 | 0.0058 | 0.0047 | 0.2709 | 0.1893 | 0.2748 | 0.1933 |
| Dominican Republic | UMIC | Americas | 0.0227 | 0.0563 | 0.0239 | 0.0575 | 0.0004 | 0.0009 | 0.0014 | 0.0020 | 0.0231 | 0.0572 | 0.0254 | 0.0594 |
| Ecuador | UMIC | Americas | 1.0521 | 1.3742 | 1.0622 | 1.3843 | 0.0165 | 0.0211 | 0.0248 | 0.0291 | 1.0687 | 1.3953 | 1.0870 | 1.4134 |
| Egypt | LMIC | Eastern Mediterranean | 0.2221 | 0.1545 | 0.2246 | 0.1570 | 0.0035 | 0.0025 | 0.0055 | 0.0046 | 0.2256 | 0.1570 | 0.2301 | 0.1616 |
| El Salvador | LMIC | Americas | 0.0898 | 0.1059 | 0.0922 | 0.1083 | 0.0015 | 0.0017 | 0.0036 | 0.0038 | 0.0913 | 0.1076 | 0.0958 | 0.1122 |
| Equatorial Guinea | UMIC | Africa | 0.1420 | 0.2315 | 0.1458 | 0.2353 | 0.0023 | 0.0036 | 0.0054 | 0.0068 | 0.1442 | 0.2351 | 0.1512 | 0.2420 |
| Estonia | HIC | Europe | 0.0000 | 0.0000 | 0.0000 | 0.0000 | 0.0000 | 0.0000 | 0.0000 | 0.0000 | 0.0000 | 0.0000 | 0.0000 | 0.0000 |
| Eswatini | LMIC | Africa | 0.2602 | 0.1632 | 0.2650 | 0.1680 | 0.0041 | 0.0026 | 0.0081 | 0.0067 | 0.2643 | 0.1658 | 0.2731 | 0.1747 |
| Ethiopia | LIC | Africa | 0.0375 | 0.0154 | 0.0378 | 0.0157 | 0.0005 | 0.0002 | 0.0007 | 0.0005 | 0.0381 | 0.0157 | 0.0385 | 0.0162 |
| Fiji | UMIC | Western Pacific | 0.1370 | 0.0228 | 0.1387 | 0.0245 | 0.0022 | 0.0004 | 0.0037 | 0.0019 | 0.1393 | 0.0232 | 0.1424 | 0.0264 |
| Finland | HIC | Europe | 0.0000 | 0.0000 | 0.0000 | 0.0000 | 0.0000 | 0.0000 | 0.0000 | 0.0000 | 0.0000 | 0.0000 | 0.0000 | 0.0000 |
| France | HIC | Europe | 0.0000 | 0.0000 | 0.0000 | 0.0000 | 0.0000 | 0.0000 | 0.0000 | 0.0000 | 0.0000 | 0.0000 | 0.0000 | 0.0000 |
| Gabon | UMIC | Africa | 0.1523 | 0.1757 | 0.1544 | 0.1778 | 0.0024 | 0.0028 | 0.0042 | 0.0045 | 0.1547 | 0.1784 | 0.1586 | 0.1824 |
| Gambia | LIC | Africa | 0.2327 | 0.1956 | 0.2344 | 0.1973 | 0.0034 | 0.0029 | 0.0047 | 0.0042 | 0.2361 | 0.1986 | 0.2391 | 0.2016 |
| Georgia | UMIC | Europe | 0.0000 | 0.0000 | 0.0022 | 0.0022 | 0.0000 | 0.0000 | 0.0020 | 0.0020 | 0.0000 | 0.0000 | 0.0042 | 0.0042 |
| Germany | HIC | Europe | 0.0000 | 0.0000 | 0.0000 | 0.0000 | 0.0000 | 0.0000 | 0.0000 | 0.0000 | 0.0000 | 0.0000 | 0.0000 | 0.0000 |
| Ghana | LMIC | Africa | 0.3130 | 0.2081 | 0.3156 | 0.2106 | 0.0047 | 0.0032 | 0.0067 | 0.0053 | 0.3177 | 0.2113 | 0.3223 | 0.2160 |
| Greece | HIC | Europe | 0.0000 | 0.0000 | 0.0000 | 0.0000 | 0.0000 | 0.0000 | 0.0000 | 0.0000 | 0.0000 | 0.0000 | 0.0000 | 0.0000 |
| Guatemala | LMIC | Americas | 0.3638 | 0.4030 | 0.3672 | 0.4064 | 0.0056 | 0.0061 | 0.0083 | 0.0088 | 0.3694 | 0.4091 | 0.3755 | 0.4152 |
| Guinea | LIC | Africa | 0.2999 | 0.2591 | 0.3020 | 0.2612 | 0.0044 | 0.0039 | 0.0060 | 0.0055 | 0.3043 | 0.2630 | 0.3081 | 0.2667 |
| Guinea-Bissau | LIC | Africa | 0.1690 | 0.1320 | 0.1702 | 0.1333 | 0.0025 | 0.0020 | 0.0034 | 0.0030 | 0.1715 | 0.1340 | 0.1737 | 0.1362 |
| Guyana | UMIC | Americas | 0.3282 | 0.3934 | 0.3325 | 0.3977 | 0.0052 | 0.0062 | 0.0088 | 0.0098 | 0.3335 | 0.3996 | 0.3414 | 0.4075 |
| Haiti | LIC | Americas | 0.3529 | 0.2957 | 0.3556 | 0.2984 | 0.0053 | 0.0045 | 0.0074 | 0.0067 | 0.3582 | 0.3003 | 0.3630 | 0.3051 |
| Honduras | LMIC | Americas | 0.4245 | 0.5275 | 0.4299 | 0.5328 | 0.0067 | 0.0082 | 0.0111 | 0.0126 | 0.4313 | 0.5357 | 0.4410 | 0.5454 |
| Hungary | HIC | Europe | 0.0000 | 0.0000 | 0.0000 | 0.0000 | 0.0000 | 0.0000 | 0.0000 | 0.0000 | 0.0000 | 0.0000 | 0.0000 | 0.0000 |
| Iceland | HIC | Europe | 0.0000 | 0.0000 | 0.0000 | 0.0000 | 0.0000 | 0.0000 | 0.0000 | 0.0000 | 0.0000 | 0.0000 | 0.0000 | 0.0000 |
| India | LMIC | South-East Asia | 0.2081 | 0.1331 | 0.2097 | 0.1347 | 0.0031 | 0.0021 | 0.0044 | 0.0034 | 0.2112 | 0.1352 | 0.2141 | 0.1381 |
| Indonesia | LMIC | South-East Asia | 0.0315 | 0.0000 | 0.0317 | 0.0003 | 0.0005 | 0.0000 | 0.0007 | 0.0003 | 0.0319 | 0.0000 | 0.0325 | 0.0005 |
| Iran | UMIC | Eastern Mediterranean | 0.0882 | 0.0702 | 0.0921 | 0.0740 | 0.0015 | 0.0012 | 0.0048 | 0.0045 | 0.0897 | 0.0714 | 0.0968 | 0.0785 |
| Iraq | UMIC | Eastern Mediterranean | 0.3400 | 0.3838 | 0.3438 | 0.3876 | 0.0053 | 0.0059 | 0.0084 | 0.0090 | 0.3453 | 0.3898 | 0.3522 | 0.3966 |
| Ireland | HIC | Europe | 0.0000 | 0.0000 | 0.0000 | 0.0000 | 0.0000 | 0.0000 | 0.0000 | 0.0000 | 0.0000 | 0.0000 | 0.0000 | 0.0000 |
| Israel | HIC | Europe | 0.0000 | 0.0000 | 0.0000 | 0.0000 | 0.0000 | 0.0000 | 0.0000 | 0.0000 | 0.0000 | 0.0000 | 0.0000 | 0.0000 |
| Italy | HIC | Europe | 0.0000 | 0.0000 | 0.0000 | 0.0000 | 0.0000 | 0.0000 | 0.0000 | 0.0000 | 0.0000 | 0.0000 | 0.0000 | 0.0000 |
| Jamaica | UMIC | Americas | 0.1556 | 0.2550 | 0.1601 | 0.2595 | 0.0026 | 0.0041 | 0.0064 | 0.0079 | 0.1582 | 0.2591 | 0.1665 | 0.2674 |
| Japan | HIC | Western Pacific | 0.0000 | 0.0000 | 0.0000 | 0.0000 | 0.0000 | 0.0000 | 0.0000 | 0.0000 | 0.0000 | 0.0000 | 0.0000 | 0.0000 |
| Jordan | UMIC | Eastern Mediterranean | 0.4244 | 0.5124 | 0.4300 | 0.5180 | 0.0067 | 0.0080 | 0.0114 | 0.0126 | 0.4311 | 0.5205 | 0.4414 | 0.5307 |
| Kazakhstan | UMIC | Europe | 0.0279 | 0.0275 | 0.0296 | 0.0292 | 0.0005 | 0.0005 | 0.0020 | 0.0020 | 0.0283 | 0.0280 | 0.0316 | 0.0312 |
| Kenya | LMIC | Africa | 0.0490 | 0.0000 | 0.0494 | 0.0004 | 0.0007 | 0.0000 | 0.0010 | 0.0003 | 0.0498 | 0.0000 | 0.0505 | 0.0007 |
| Kuwait | HIC | Eastern Mediterranean | 0.0000 | 0.0000 | 0.0000 | 0.0000 | 0.0000 | 0.0000 | 0.0000 | 0.0000 | 0.0000 | 0.0000 | 0.0000 | 0.0000 |
| Kyrgyzstan | LMIC | Europe | 0.2688 | 0.0424 | 0.2734 | 0.0471 | 0.0043 | 0.0007 | 0.0081 | 0.0047 | 0.2731 | 0.0432 | 0.2815 | 0.0517 |
| Lao PDR | LMIC | Western Pacific | 0.3517 | 0.3433 | 0.3543 | 0.3460 | 0.0053 | 0.0052 | 0.0074 | 0.0073 | 0.3570 | 0.3485 | 0.3617 | 0.3533 |
| Latvia | HIC | Europe | 0.0000 | 0.0000 | 0.0000 | 0.0000 | 0.0000 | 0.0000 | 0.0000 | 0.0000 | 0.0000 | 0.0000 | 0.0000 | 0.0000 |
| Lebanon | UMIC | Eastern Mediterranean | 0.0000 | 0.0592 | 0.0040 | 0.0631 | 0.0000 | 0.0010 | 0.0035 | 0.0045 | 0.0000 | 0.0601 | 0.0075 | 0.0676 |
| Lesotho | LMIC | Africa | 0.6305 | 0.5815 | 0.6359 | 0.5869 | 0.0096 | 0.0089 | 0.0139 | 0.0133 | 0.6401 | 0.5904 | 0.6499 | 0.6002 |
| Liberia | LIC | Africa | 0.1561 | 0.0911 | 0.1574 | 0.0923 | 0.0023 | 0.0014 | 0.0033 | 0.0024 | 0.1585 | 0.0925 | 0.1607 | 0.0947 |
| Libya | UMIC | Eastern Mediterranean | 0.1705 | 0.2952 | 0.1759 | 0.3006 | 0.0028 | 0.0048 | 0.0075 | 0.0094 | 0.1733 | 0.3000 | 0.1833 | 0.3100 |
| Lithuania | HIC | Europe | 0.0000 | 0.0000 | 0.0000 | 0.0000 | 0.0000 | 0.0000 | 0.0000 | 0.0000 | 0.0000 | 0.0000 | 0.0000 | 0.0000 |
| Luxembourg | HIC | Europe | 0.0000 | 0.0000 | 0.0000 | 0.0000 | 0.0000 | 0.0000 | 0.0000 | 0.0000 | 0.0000 | 0.0000 | 0.0000 | 0.0000 |
| Madagascar | LIC | Africa | 0.3725 | 0.3404 | 0.3751 | 0.3430 | 0.0055 | 0.0051 | 0.0075 | 0.0071 | 0.3781 | 0.3455 | 0.3827 | 0.3502 |
| Malawi | LIC | Africa | 0.1680 | 0.1449 | 0.1692 | 0.1461 | 0.0025 | 0.0022 | 0.0034 | 0.0031 | 0.1705 | 0.1471 | 0.1727 | 0.1492 |
| Malaysia | UMIC | Western Pacific | 0.0378 | 0.0598 | 0.0397 | 0.0617 | 0.0006 | 0.0010 | 0.0023 | 0.0027 | 0.0384 | 0.0608 | 0.0420 | 0.0643 |
| Maldives | UMIC | South-East Asia | 0.0000 | 0.0000 | 0.0023 | 0.0023 | 0.0000 | 0.0000 | 0.0020 | 0.0020 | 0.0000 | 0.0000 | 0.0043 | 0.0043 |
| Mali | LIC | Africa | 0.2774 | 0.2105 | 0.2794 | 0.2126 | 0.0041 | 0.0032 | 0.0057 | 0.0048 | 0.2815 | 0.2137 | 0.2851 | 0.2174 |
| Malta | HIC | Europe | 0.0000 | 0.0000 | 0.0000 | 0.0000 | 0.0000 | 0.0000 | 0.0000 | 0.0000 | 0.0000 | 0.0000 | 0.0000 | 0.0000 |
| Mauritania | LMIC | Africa | 0.2872 | 0.2284 | 0.2894 | 0.2306 | 0.0043 | 0.0035 | 0.0060 | 0.0052 | 0.2915 | 0.2318 | 0.2954 | 0.2358 |
| Mauritius | UMIC | Africa | 0.1422 | 0.1302 | 0.1438 | 0.1318 | 0.0024 | 0.0022 | 0.0037 | 0.0036 | 0.1445 | 0.1323 | 0.1475 | 0.1354 |
| Mexico | UMIC | Americas | 0.1602 | 0.1672 | 0.1616 | 0.1687 | 0.0025 | 0.0026 | 0.0037 | 0.0037 | 0.1626 | 0.1698 | 0.1653 | 0.1724 |
| Mongolia | LMIC | Western Pacific | 0.0000 | 0.0000 | 0.0041 | 0.0041 | 0.0000 | 0.0000 | 0.0036 | 0.0036 | 0.0000 | 0.0000 | 0.0077 | 0.0077 |
| Montenegro | UMIC | Europe | 0.0426 | 0.0000 | 0.0446 | 0.0021 | 0.0007 | 0.0000 | 0.0026 | 0.0019 | 0.0433 | 0.0000 | 0.0473 | 0.0040 |
| Morocco | LMIC | Eastern Mediterranean | 0.5317 | 0.4911 | 0.5358 | 0.4953 | 0.0082 | 0.0076 | 0.0115 | 0.0110 | 0.5398 | 0.4987 | 0.5474 | 0.5063 |
| Mozambique | LIC | Africa | 0.2539 | 0.2300 | 0.2557 | 0.2318 | 0.0038 | 0.0035 | 0.0052 | 0.0049 | 0.2577 | 0.2334 | 0.2609 | 0.2367 |
| Myanmar | LMIC | South-East Asia | 0.3399 | 0.3190 | 0.3425 | 0.3216 | 0.0053 | 0.0050 | 0.0074 | 0.0071 | 0.3452 | 0.3240 | 0.3499 | 0.3287 |
| Namibia | UMIC | Africa | 0.0674 | 0.2242 | 0.0721 | 0.2289 | 0.0011 | 0.0036 | 0.0051 | 0.0075 | 0.0686 | 0.2279 | 0.0772 | 0.2364 |
| Nepal | LIC | South-East Asia | 1.0965 | 1.1173 | 1.1042 | 1.1249 | 0.0167 | 0.0170 | 0.0228 | 0.0230 | 1.1132 | 1.1342 | 1.1269 | 1.1479 |
| Netherlands | HIC | Europe | 0.0000 | 0.0000 | 0.0000 | 0.0000 | 0.0000 | 0.0000 | 0.0000 | 0.0000 | 0.0000 | 0.0000 | 0.0000 | 0.0000 |
| New Zealand | HIC | Western Pacific | 0.0000 | 0.0000 | 0.0000 | 0.0000 | 0.0000 | 0.0000 | 0.0000 | 0.0000 | 0.0000 | 0.0000 | 0.0000 | 0.0000 |
| Nicaragua | LMIC | Americas | 0.0547 | 0.1485 | 0.0591 | 0.1529 | 0.0009 | 0.0025 | 0.0048 | 0.0063 | 0.0556 | 0.1510 | 0.0639 | 0.1592 |
| Niger | LIC | Africa | 0.1307 | 0.1142 | 0.1316 | 0.1151 | 0.0019 | 0.0017 | 0.0026 | 0.0024 | 0.1326 | 0.1159 | 0.1342 | 0.1175 |
| Nigeria | LMIC | Africa | 0.1891 | 0.0000 | 0.1908 | 0.0017 | 0.0029 | 0.0000 | 0.0042 | 0.0014 | 0.1920 | 0.0000 | 0.1949 | 0.0031 |
| North Macedonia | UMIC | Europe | 0.0910 | 0.0142 | 0.0936 | 0.0169 | 0.0016 | 0.0002 | 0.0039 | 0.0026 | 0.0925 | 0.0145 | 0.0975 | 0.0195 |
| Norway | HIC | Europe | 0.0000 | 0.0000 | 0.0000 | 0.0000 | 0.0000 | 0.0000 | 0.0000 | 0.0000 | 0.0000 | 0.0000 | 0.0000 | 0.0000 |
| Oman | HIC | Eastern Mediterranean | 0.0000 | 0.0000 | 0.0000 | 0.0000 | 0.0000 | 0.0000 | 0.0000 | 0.0000 | 0.0000 | 0.0000 | 0.0000 | 0.0000 |
| Pakistan | LMIC | Eastern Mediterranean | 0.1598 | 0.1319 | 0.1610 | 0.1331 | 0.0024 | 0.0020 | 0.0033 | 0.0029 | 0.1622 | 0.1339 | 0.1643 | 0.1360 |
| Panama | UMIC | Americas | 0.0000 | 0.0741 | 0.0018 | 0.0759 | 0.0000 | 0.0012 | 0.0016 | 0.0028 | 0.0000 | 0.0753 | 0.0034 | 0.0787 |
| Papua New Guinea | LMIC | Western Pacific | 0.1641 | 0.0705 | 0.1655 | 0.0719 | 0.0025 | 0.0011 | 0.0036 | 0.0023 | 0.1667 | 0.0717 | 0.1691 | 0.0741 |
| Paraguay | UMIC | Americas | 0.1006 | 0.1459 | 0.1039 | 0.1492 | 0.0017 | 0.0024 | 0.0046 | 0.0053 | 0.1023 | 0.1483 | 0.1085 | 0.1546 |
| Peru | UMIC | Americas | 0.5131 | 1.0124 | 0.5211 | 1.0204 | 0.0083 | 0.0156 | 0.0150 | 0.0220 | 0.5214 | 1.0280 | 0.5361 | 1.0424 |
| Philippines | LMIC | Western Pacific | 0.0874 | 0.0000 | 0.0883 | 0.0009 | 0.0013 | 0.0000 | 0.0020 | 0.0007 | 0.0888 | 0.0000 | 0.0903 | 0.0016 |
| Poland | HIC | Europe | 0.0000 | 0.0000 | 0.0000 | 0.0000 | 0.0000 | 0.0000 | 0.0000 | 0.0000 | 0.0000 | 0.0000 | 0.0000 | 0.0000 |
| Portugal | HIC | Europe | 0.0000 | 0.0000 | 0.0000 | 0.0000 | 0.0000 | 0.0000 | 0.0000 | 0.0000 | 0.0000 | 0.0000 | 0.0000 | 0.0000 |
| Puerto Rico | HIC |  | 0.0000 | 0.0000 | 0.0000 | 0.0000 | 0.0000 | 0.0000 | 0.0000 | 0.0000 | 0.0000 | 0.0000 | 0.0000 | 0.0000 |
| Qatar | HIC | Eastern Mediterranean | 0.0000 | 0.0000 | 0.0000 | 0.0000 | 0.0000 | 0.0000 | 0.0000 | 0.0000 | 0.0000 | 0.0000 | 0.0000 | 0.0000 |
| Republic of Korea | HIC | Western Pacific | 0.0000 | 0.0000 | 0.0000 | 0.0000 | 0.0000 | 0.0000 | 0.0000 | 0.0000 | 0.0000 | 0.0000 | 0.0000 | 0.0000 |
| Republic of Moldova | LMIC | Europe | 0.0130 | 0.0000 | 0.0174 | 0.0044 | 0.0002 | 0.0000 | 0.0043 | 0.0040 | 0.0132 | 0.0000 | 0.0217 | 0.0085 |
| Romania | UMIC | Europe | 0.0202 | 0.0000 | 0.0219 | 0.0017 | 0.0004 | 0.0000 | 0.0019 | 0.0016 | 0.0206 | 0.0000 | 0.0239 | 0.0033 |
| Russian Federation | UMIC | Europe | 0.0000 | 0.0000 | 0.0027 | 0.0027 | 0.0000 | 0.0000 | 0.0024 | 0.0024 | 0.0000 | 0.0000 | 0.0051 | 0.0051 |
| Rwanda | LIC | Africa | 0.1521 | 0.1184 | 0.1532 | 0.1196 | 0.0023 | 0.0018 | 0.0032 | 0.0028 | 0.1544 | 0.1203 | 0.1564 | 0.1224 |
| SVG | UMIC | Americas | 0.1169 | 0.1523 | 0.1201 | 0.1555 | 0.0019 | 0.0025 | 0.0047 | 0.0052 | 0.1189 | 0.1548 | 0.1248 | 0.1607 |
| Saint Lucia | UMIC | Americas | 0.0219 | 0.1031 | 0.0252 | 0.1065 | 0.0004 | 0.0017 | 0.0033 | 0.0046 | 0.0223 | 0.1048 | 0.0286 | 0.1111 |
| Samoa | LMIC | Western Pacific | 0.2731 | 0.1467 | 0.2753 | 0.1489 | 0.0043 | 0.0024 | 0.0060 | 0.0043 | 0.2774 | 0.1491 | 0.2814 | 0.1532 |
| Sao Tome and Principe | LMIC | Africa | 0.1638 | 0.0000 | 0.1657 | 0.0019 | 0.0025 | 0.0000 | 0.0041 | 0.0016 | 0.1664 | 0.0000 | 0.1698 | 0.0035 |
| Saudi Arabia | HIC | Eastern Mediterranean | 0.0000 | 0.0000 | 0.0000 | 0.0000 | 0.0000 | 0.0000 | 0.0000 | 0.0000 | 0.0000 | 0.0000 | 0.0000 | 0.0000 |
| Senegal | LIC | Africa | 0.2310 | 0.1860 | 0.2327 | 0.1876 | 0.0034 | 0.0028 | 0.0047 | 0.0041 | 0.2344 | 0.1888 | 0.2374 | 0.1918 |
| Serbia | UMIC | Europe | 0.0187 | 0.0000 | 0.0222 | 0.0035 | 0.0003 | 0.0000 | 0.0036 | 0.0032 | 0.0190 | 0.0000 | 0.0258 | 0.0068 |
| Sierra Leone | LIC | Africa | 0.2893 | 0.2438 | 0.2914 | 0.2459 | 0.0043 | 0.0037 | 0.0059 | 0.0053 | 0.2935 | 0.2475 | 0.2972 | 0.2512 |
| Singapore | HIC | Western Pacific | 0.0000 | 0.0000 | 0.0000 | 0.0000 | 0.0000 | 0.0000 | 0.0000 | 0.0000 | 0.0000 | 0.0000 | 0.0000 | 0.0000 |
| Slovakia | HIC | Europe | 0.0000 | 0.0000 | 0.0000 | 0.0000 | 0.0000 | 0.0000 | 0.0000 | 0.0000 | 0.0000 | 0.0000 | 0.0000 | 0.0000 |
| Slovenia | HIC | Europe | 0.0000 | 0.0000 | 0.0000 | 0.0000 | 0.0000 | 0.0000 | 0.0000 | 0.0000 | 0.0000 | 0.0000 | 0.0000 | 0.0000 |
| Solomon Islands | LMIC | Western Pacific | 0.1220 | 0.0000 | 0.1234 | 0.0013 | 0.0019 | 0.0000 | 0.0030 | 0.0012 | 0.1240 | 0.0000 | 0.1264 | 0.0025 |
| South Africa | UMIC | Africa | 0.0193 | 0.0000 | 0.0217 | 0.0024 | 0.0003 | 0.0000 | 0.0024 | 0.0021 | 0.0196 | 0.0000 | 0.0241 | 0.0045 |
| Spain | HIC | Europe | 0.0000 | 0.0000 | 0.0000 | 0.0000 | 0.0000 | 0.0000 | 0.0000 | 0.0000 | 0.0000 | 0.0000 | 0.0000 | 0.0000 |
| Sri Lanka | LMIC | South-East Asia | 0.1136 | 0.0697 | 0.1148 | 0.0708 | 0.0019 | 0.0012 | 0.0028 | 0.0022 | 0.1155 | 0.0709 | 0.1176 | 0.0730 |
| Sudan | LMIC | Eastern Mediterranean | 0.3201 | 0.2357 | 0.3237 | 0.2392 | 0.0050 | 0.0037 | 0.0078 | 0.0066 | 0.3251 | 0.2394 | 0.3315 | 0.2458 |
| Suriname | UMIC | Americas | 0.0000 | 0.0302 | 0.0035 | 0.0337 | 0.0000 | 0.0005 | 0.0031 | 0.0036 | 0.0000 | 0.0307 | 0.0067 | 0.0373 |
| Sweden | HIC | Europe | 0.0000 | 0.0000 | 0.0000 | 0.0000 | 0.0000 | 0.0000 | 0.0000 | 0.0000 | 0.0000 | 0.0000 | 0.0000 | 0.0000 |
| Switzerland | HIC | Europe | 0.0000 | 0.0000 | 0.0000 | 0.0000 | 0.0000 | 0.0000 | 0.0000 | 0.0000 | 0.0000 | 0.0000 | 0.0000 | 0.0000 |
| Tajikistan | LMIC | Europe | 0.2663 | 0.1978 | 0.2690 | 0.2004 | 0.0041 | 0.0031 | 0.0063 | 0.0053 | 0.2704 | 0.2009 | 0.2753 | 0.2057 |
| Thailand | UMIC | South-East Asia | 0.1121 | 0.1183 | 0.1140 | 0.1201 | 0.0019 | 0.0020 | 0.0035 | 0.0036 | 0.1140 | 0.1203 | 0.1175 | 0.1237 |
| Timor-Leste | LMIC | South-East Asia | 0.3686 | 0.3532 | 0.3716 | 0.3561 | 0.0056 | 0.0054 | 0.0080 | 0.0078 | 0.3742 | 0.3586 | 0.3796 | 0.3639 |
| Togo | LIC | Africa | 0.2022 | 0.1601 | 0.2037 | 0.1616 | 0.0030 | 0.0024 | 0.0041 | 0.0036 | 0.2052 | 0.1625 | 0.2078 | 0.1652 |
| Tonga | LMIC | Western Pacific | 0.1119 | 0.0000 | 0.1138 | 0.0019 | 0.0018 | 0.0000 | 0.0034 | 0.0017 | 0.1137 | 0.0000 | 0.1172 | 0.0036 |
| Trinidad and Tobago | HIC | Americas | 0.0000 | 0.0000 | 0.0000 | 0.0000 | 0.0000 | 0.0000 | 0.0000 | 0.0000 | 0.0000 | 0.0000 | 0.0000 | 0.0000 |
| Tunisia | LMIC | Eastern Mediterranean | 0.2131 | 0.0023 | 0.2181 | 0.0074 | 0.0035 | 0.0000 | 0.0079 | 0.0045 | 0.2166 | 0.0024 | 0.2260 | 0.0119 |
| Turkey | UMIC | Europe | 0.0000 | 0.0000 | 0.0036 | 0.0036 | 0.0000 | 0.0000 | 0.0032 | 0.0032 | 0.0000 | 0.0000 | 0.0068 | 0.0068 |
| Turkmenistan | UMIC | Europe | 0.1786 | 0.1865 | 0.1811 | 0.1890 | 0.0028 | 0.0030 | 0.0049 | 0.0050 | 0.1814 | 0.1894 | 0.1860 | 0.1940 |
| Uganda | LIC | Africa | 0.1847 | 0.1461 | 0.1861 | 0.1475 | 0.0027 | 0.0022 | 0.0038 | 0.0033 | 0.1875 | 0.1483 | 0.1899 | 0.1508 |
| Ukraine | LMIC | Europe | 0.0000 | 0.0000 | 0.0024 | 0.0024 | 0.0000 | 0.0000 | 0.0022 | 0.0022 | 0.0000 | 0.0000 | 0.0046 | 0.0046 |
| United Arab Emirates | HIC | Eastern Mediterranean | 0.0000 | 0.0000 | 0.0000 | 0.0000 | 0.0000 | 0.0000 | 0.0000 | 0.0000 | 0.0000 | 0.0000 | 0.0000 | 0.0000 |
| United Kingdom | HIC | Europe | 0.0000 | 0.0000 | 0.0000 | 0.0000 | 0.0000 | 0.0000 | 0.0000 | 0.0000 | 0.0000 | 0.0000 | 0.0000 | 0.0000 |
| United Republic of Tanzania | LIC | Africa | 0.1797 | 0.1547 | 0.1810 | 0.1559 | 0.0027 | 0.0023 | 0.0037 | 0.0033 | 0.1823 | 0.1570 | 0.1846 | 0.1593 |
| United States of America | HIC | Americas | 0.0000 | 0.0000 | 0.0000 | 0.0000 | 0.0000 | 0.0000 | 0.0000 | 0.0000 | 0.0000 | 0.0000 | 0.0000 | 0.0000 |
| Uruguay | HIC | Americas | 0.0000 | 0.0000 | 0.0000 | 0.0000 | 0.0000 | 0.0000 | 0.0000 | 0.0000 | 0.0000 | 0.0000 | 0.0000 | 0.0000 |
| Uzbekistan | LMIC | Europe | 0.3629 | 0.1647 | 0.3679 | 0.1698 | 0.0058 | 0.0027 | 0.0099 | 0.0070 | 0.3686 | 0.1674 | 0.3778 | 0.1767 |
| Vanuatu | LMIC | Western Pacific | 0.2578 | 0.0819 | 0.2600 | 0.0842 | 0.0040 | 0.0014 | 0.0059 | 0.0033 | 0.2618 | 0.0833 | 0.2659 | 0.0875 |
| Viet Nam | LMIC | Western Pacific | 0.4092 | 0.3504 | 0.4131 | 0.3543 | 0.0065 | 0.0057 | 0.0098 | 0.0089 | 0.4158 | 0.3561 | 0.4229 | 0.3632 |
| Zambia | LMIC | Africa | 0.2853 | 0.2422 | 0.2875 | 0.2444 | 0.0042 | 0.0037 | 0.0059 | 0.0054 | 0.2895 | 0.2459 | 0.2934 | 0.2497 |
| Zimbabwe | LIC | Africa | 1.5486 | 1.1584 | 1.5621 | 1.1719 | 0.0235 | 0.0180 | 0.0341 | 0.0288 | 1.5721 | 1.1764 | 1.5962 | 1.2007 |

Approach 1 calculates unmet need as the relative difference of surgical volume to the minimum surgical volume proposed by the Lancet Commission on Global Surgery. Approach 2 calculates unmet need as the relative difference of estimated appendectomies (calculated as World Bank-income-group specific share of surgical volume) to the number of appendicitis cases.

1. As noted in the main document, this can be confirmed using OECD data on hospitalization in 2015. [↑](#footnote-ref-1)
2. This is different to the probability to receive surgical care or not, i.e., to be operated or not, which is derived from country-level surgical volume as explained above. [↑](#footnote-ref-2)
3. 4 studies for Northern America (Canada: 2, USA: 2), 2 studies for Northern Europe (Denmark, UK), 3 studies for Southern Europe (Albania, Italy, Spain), 2 studies for the Middle East (Iran, Turkey), 5 studies for Asia (South Korea, Taiwan: 4), 3 studies for Africa (South Africa, Nigeria: 2), and none for Eastern Europe, Western Europe, Oceania, or Southern and Central America. [↑](#footnote-ref-3)
